# Discovering gene re-ranking efficiency and conserved gene-gene relationships derived from gene co-expression network analysis on breast cancer data

**DOI:** 10.1038/srep20518

**Published:** 2016-02-19

**Authors:** Marilena M. Bourdakou, Emmanouil I. Athanasiadis, George M. Spyrou

**Affiliations:** 1 Center of Systems Biology, Biomedical Research Foundation, Academy of Athens, Soranou Ephessiou 4, 115 27 Athens, Greece; 2 Department of Informatics and Telecommunications, University of Athens, 15784 Ilissia Athens, Greece

## Abstract

Systemic approaches are essential in the discovery of disease-specific genes, offering a different perspective and new tools on the analysis of several types of molecular relationships, such as gene co-expression or protein-protein interactions. However, due to lack of experimental information, this analysis is not fully applicable. The aim of this study is to reveal the multi-potent contribution of statistical network inference methods in highlighting significant genes and interactions. We have investigated the ability of statistical co-expression networks to highlight and prioritize genes for breast cancer subtypes and stages in terms of: (i) classification efficiency, (ii) gene network pattern conservation, (iii) indication of involved molecular mechanisms and (iv) systems level momentum to drug repurposing pipelines. We have found that statistical network inference methods are advantageous in gene prioritization, are capable to contribute to meaningful network signature discovery, give insights regarding the disease-related mechanisms and boost drug discovery pipelines from a systems point of view.

Breast cancer is a major public health problem, since it remains the most frequently diagnosed cancer and ranked second as a cause of death in women population. Outbreaks are increasing in most countries, despite current efforts have been made to avoid the disease^1^. This happens because breast cancer is a complex disease with many contributing factors affecting the progress of the disease. Despite the fact that many studies have been conducted, neither the exact etiology of the breast cancer, nor the mechanisms behind the heterogeneity from patient to patient are known. For this, the diagnosis and the treatment of breast cancer remain a both challenging and fascinating task^2^.

With the rapid development of genome-wide gene expression profiling methodologies, many bioinformatics data analysis pipelines have been developed to identify breast cancer related genes and discover gene signatures for prognosis and treatment prediction. However, since breast cancer is a complex disease, it should be determined not only by individual genes, but also by the coordinated effect of numerous genes^3^. The information behind gene interaction networks is of great importance due to the fact that all cellular functions are regulated by gene patterns, where the presence or absence of an interaction may cause the emergence of a disease.

Network analysis and graph theory support the study of interactions among relatively large number of genes in order to conclude to large lists of statistically significant genes^4^,^5^,^6^. Several bioinformatics tools, like PINTA^7^, prioritize genes by combining gene expression data with the protein-protein interaction (PPI) network through a random walk approach to enrich the candidate genes and finally re-rank them. The majority of these methods necessitate prior knowledge to re-rank genes accordingly. However, due to the absence of functional characterizations for a significant number of genes, these approaches are not fully applicable^8^. Genome-wide association studies (GWAS) have recognized DNA variants that are related to common complex diseases but for many of these studies, functional associations between genes and diseases are unknown^9^. In order to overcome this hurdle, several network inference methods have been adopted to construct statistical co-expression networks, based on gene expression data. These network inference approaches identify groups of genes that are highly correlated in expression levels to multiple samples according to a variety of correlation functions and algorithms^10^,^11^,^12^,^13^,^14^.

In this study, we investigate the ability of statistical co-expression networks to highlight and prioritize significant genes at four different breast cancer molecular subtypes, including Luminal A, Luminal B, HER2 and Triple Negative as well as at four different disease stages (I-IV) in terms of: (i) classification efficiency, (ii) gene subnetwork conservation, (iii) involved molecular mechanisms investigation and (iv) potential boost to drug repurposing pipelines.

Specifically, we have used mRNA gene expression microarray data concerning Breast Invasive Carcinoma, retrieved from The Cancer Genome Atlas – TCGA (http://gdac.broadinstitute.org/runs/stddata__latest/samples_report/BRCA.html), to reconstruct 17 different networks (twelve based on mathematical correlation and six based on the literature) of the top differentially expressed genes. Using a mathematical function that combines gene expression data with custom networks, we prioritized genes based on each network. Furthermore, in order to investigate the quality of each prioritized gene list, we elucidated the impact of each one over sample discrimination, by applying a hold out validation scheme using the TCGA data as training set and a number of Breast cancer datasets from the transcriptional data repository Gene Expression Omnibus GEO (http://www.ncbi.nlm.nih.gov/geo/)^15^ as test sets. Using the network inference method that performed the highest classification score, we constructed co-expression networks for all datasets (train and test sets) to find the most significant gene-gene links that recur in all networks. With the proposed pipeline, we concluded to breast cancer specific network patterns per subtype and stage. Analyzing each pattern we concluded in specific mechanisms per subtype and stage related to cellular community (cell communication, focal adhesion), signaling (in terms of extracellular matrix and cytokine receptor interactions), cell growth and death (cell cycle), immune system (including complement and coagulation cascades and toll like receptor signaling pathway), endocrine system (ppar and adipocytokine signaling pathway), carbohydrate, lipid and amino acid metabolism (glycolysis/gluconeogenesis, fatty acid and glycerolipid metabolism, bile acid biosynthesis, as well as tyrosine, phenylalanine, glycine, serine, threonine metabolism) and xenobiotics biodegradation and metabolism (3 chloroacylic acid and 1,2 methylnaphthalene degradation, metabolism of xenobiotics by cytochrome p450). Interestingly, all the derived network patterns include genes found in breast cancer specific regions of significant somatic copy number alterations (SCNA)^16^. Finally, the genes from the conserved network patterns were used in a drug repurposing pipeline, revealing drugs that have the potential to suppress breast cancer specifically for each molecular subtype and stage of the disease. Figure 1 illustrates the conceptual pipeline of our method.

## Results

### Evaluation of gene re-ranking through a classification scheme

**Figure 2:**
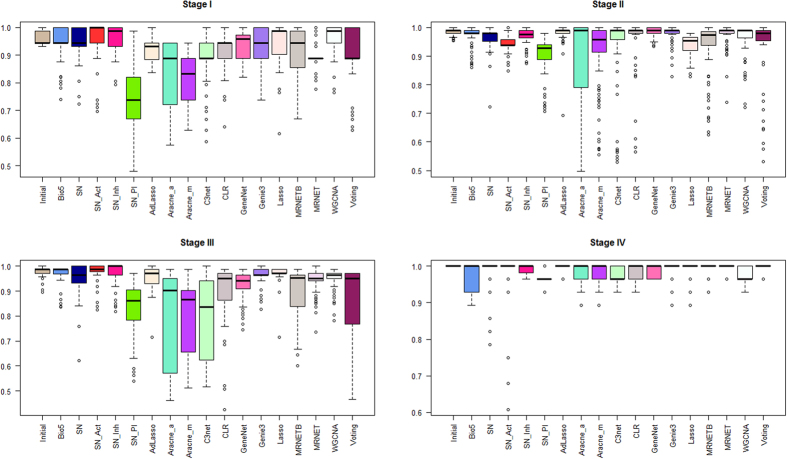
Box plots of the mean accuracy rates of the top 100 sequential genes from all ranked and re-ranked gene lists in combination with PageRank reconciling method, using hold out validation with train set the TCGA expression values and test set the expression values from GEO independent datasets for breast cancer stages.

**Figure 3:**
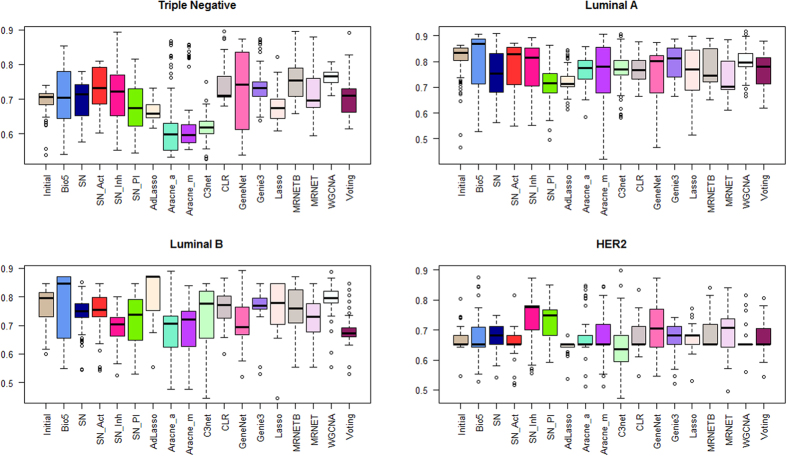
Box plots of the mean accuracy rates of the top 100 sequential genes from all ranked and re-ranked gene lists in combination with PageRank reconciling method, using hold out validation with train set the TCGA expression values and test set the expression values from GEO independent datasets for breast cancer subtypes.

The top 1000 re-ranked gene lists for each subtype and stage, along with the initially ranked list, gave us a total number of 18 ranked gene lists. In order to evaluate each list, we elucidated the impact of the top 100 genes from each list over sample discrimination, by applying a hold out validation scheme. More precisely, we employed a Support Vector Machine (SVM) – based classification scheme using the e1071 R package^17^ through sequential gene selection of the first 100 genes, using as Train set the expression values of each top 100 gene list from the reference set (TCGA) and as Test sets the expression values of the same top 100 genes from a number of independent GEO datasets (discovery sets) available for each subtype and stage. We followed the same procedure for each top 100 gene lists and we calculated the mean classification accuracy from the discovery datasets in a sequential gene selection manner. Figures 2 and 3 show the box plots of the mean classification accuracies of the top 100 sequential genes for each network approach using the Page Rank reconciling method for each stage and subtype. We observe that the median accuracy values of all methods are greater than 70% in Stage I, 90% in Stage II, 80% in Stage III and 95% in Stage IV. Regarding subtypes, the median accuracy values of all methods are greater than 58% in Triple Negative, 70% in Luminal A, 65% in Luminal B and 65% in HER2. Furthermore, in most cases the median classification performances of the top 100 gene lists from network inference methods are either better or equivalent compared to the median performance of the initial gene list. The mean accuracy plots for each ranked and re-ranked lists are available at Supplementary Figs 1–45.

Each ranking method is scored according to the maximum achieved mean classification accuracy across datasets, modified by two multiplicative weights: wn that is related to the number of genes required for the maximum accuracy and wcv that is related to the coefficient of variation (CV) of the classification accuracy along the first 100 genes (see **Methods**).

**Figure 4:**
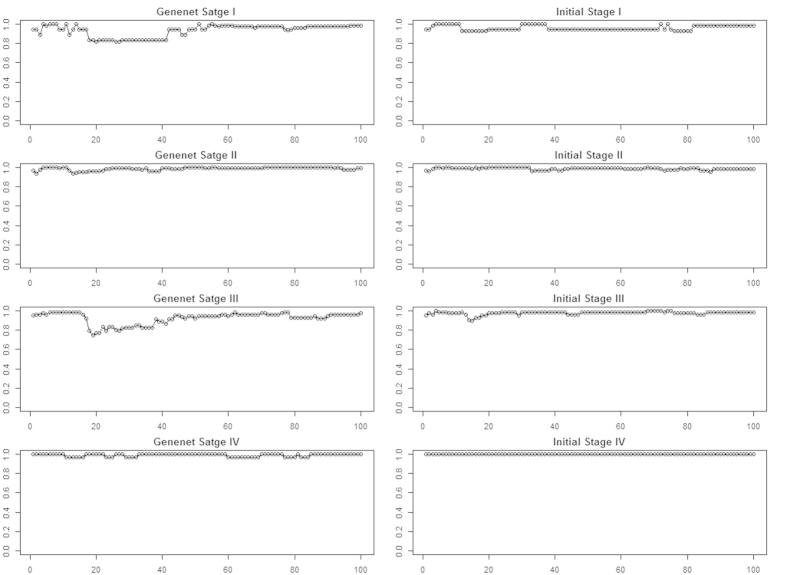
Mean accuracy rates of the top 100 sequential genes from the Genenet network inference method and the Initial for each breast cancer stage.

**Figure 5:**
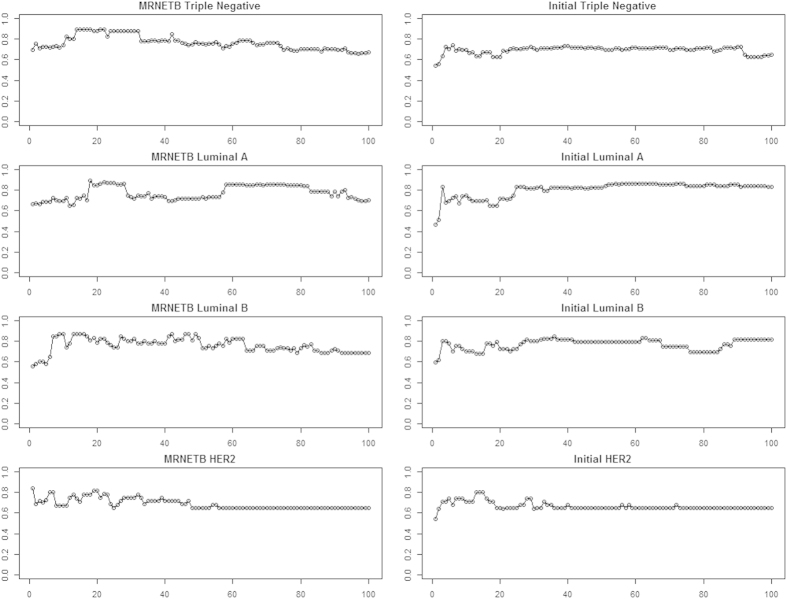
Mean accuracy rates of the top 100 sequential genes from the MRNETB network inference method and the Initial for each breast cancer subtype.

The maximum average score for breast cancer stages (Table 1) and subtypes (Table 2) was achieved by Genenet network inference method and Maximum Relevance Minimum Redundancy Backward (MRNETB), respectively. For this reason we adopted them for the rest of our analysis. It is worth mentioning that the selected statistical network inference methods achieved a higher or equivalent score compared to the initial ranking in most cases (Figs 4, 5).

### Deriving a common Network Pattern

**Figure 6:**
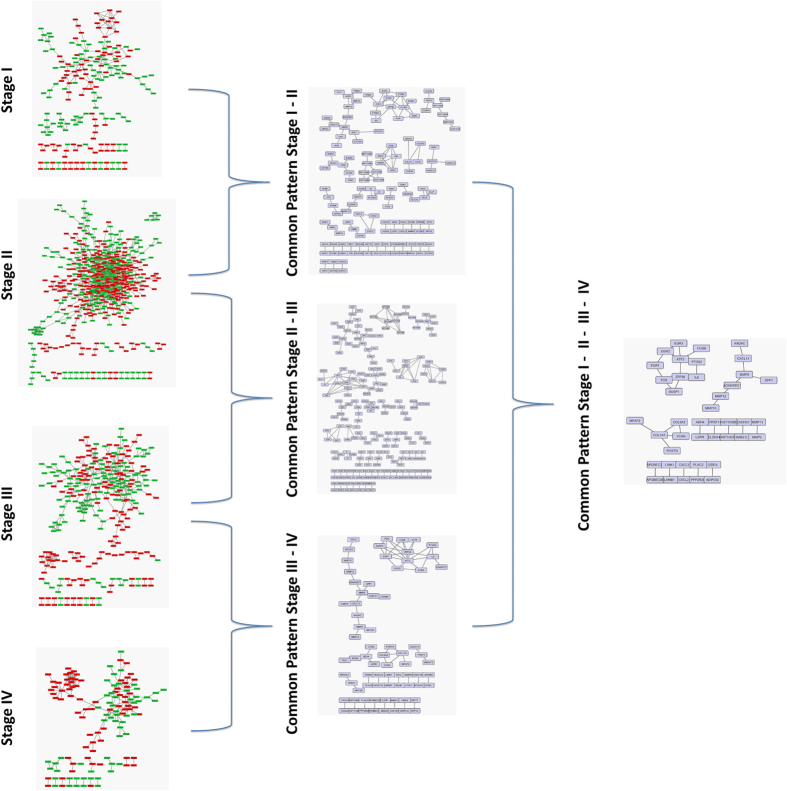
Network pattern for each breast cancer stage and the common edges across them.

**Figure 7:**
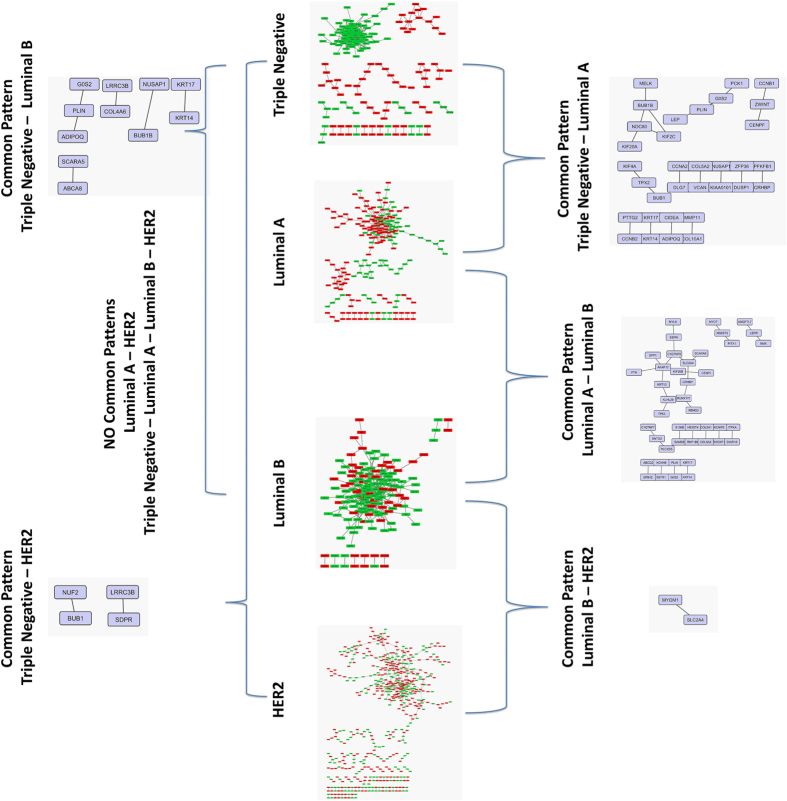
Network pattern for each breast cancer subtype and the common interactions across Luminal A and Luminal B.

We applied the Genenet and MRNETB network inference methods to reconstruct gene co-expression networks for each of the available dataset for each stage and subtype. In order to highlight any common gene network pattern, we found the common edges across all datasets. We performed a dynamic filtering to keep only the highly weighted gene - gene links, by removing the weakest edges from the common network until we concluded to the maximum fully connected cluster (clique), satisfying two criteria: i) it is not identical with the initial network, (ii) the number of its nodes is more than 10% of the number of nodes of the initial network. Finally, we came up with 205 genes-nodes and 216 edges for Stage I, 561 genes-nodes and 896 edges for Stage II, 289 nodes and 380 edges for Stage III and 132genes-nodes and 169 edges for Stage IV. As far as subtypes are concerned, we came up with 196 genes-nodes and 872 edges for Triple Negative, 201 genes-nodes and 272 edges for Luminal A, 155 genes-nodes and 305 edges for Luminal B and 544 genes-nodes and 573 edges for HER2. From these patterns we highlighted the top 100 interactions for each stage and subtype based on their weights (Supplementary Figs 46–53). Furthermore, we found the common edges among the gene network patterns of the successive pairs of disease staging (I–II, II–III, III–IV). Finally we concluded in the common pattern across all the breast cancer stages (Fig. 6). We repeated the same procedure for the breast cancer subtypes for all possible pair combinations (Fig. 7).

### Network inference, underlying mechanisms

We used the Enrichr web-based software application (http://amp.pharm.mssm.edu/Enrichr/)^18^ in order to find the underlying significant biological pathways derived from genes of each network pattern. Common and exclusive mechanisms of each stage and subtype were further investigated (Tables 3, 4).

Following pathway analysis of our findings for the case of Staging, we have found four exclusive stage-related pathways including *phenylalanine metabolism* for Stage II, *peroxisome proliferator-activated* (PPAR) *signaling pathway* and *glycolysis and gluconeogenesis* for Stage III and *toll like receptor signaling pathway* for Stage IV. For the cases of *phenylalanine metabolism* and *glycolysis/gluconeogenesis* pathways, it has been reported that ALDH1A3 involved in both pathways is expressed at significantly higher levels in tumors that lacked expression of the ER. In addition, expression of ALDH1A3 was positively associated with grade in ER-positive tumors, as well as positively correlated with tumor staging, rendering ALDH1A3 a candidate biomarker for metastasis in invasive breast cancers. Activation of peroxisome proliferator-activated receptor α (PPARα) has been reported to inhibit tumor growth and angiogenesis in cancer cells^19^, while suggesting the development of PPAR agonists as anticancer agents. Nevertheless, on the latter analysis, no evidence regarding the staging was performed. IL-6 (IL6) cytokine found in *toll like receptor signaling pathway* has been involved in acute and chronic inflammation and has been associated with cancer progression^20^. It also plays an etiologic role in the development of cognitive difficulties in breast cancer patients. For the case of SPP1 (Stage IV), metastasis-associated protein *Osteopontin* has been tightly correlated with a poor prognosis, almost certainly caused by metastatic spread from the primary tumor in human breast cancer^21^. We have also revealed three common pathways found in all four Stages including *cell communication*, *cytokine receptor interaction* and *ecm receptor interaction* pathways. Collagen alpha-1(I) chain Protein (COL1A1) found in all the aforementioned pathways was recently proposed as a potential biomarker of breast cancer^22^.

For the case of Luminal A, Luminal B, HER2 and TN subtypes, we have found seven exclusive subtype-related pathways, including *glycine serine and threonine metabolism pathway* for Luminal B, *glycerolipid metabolism*, *fatty acid metabolism*, *complement and coagulation cascades* and *bladder cancer* for HER2 and *small cell lung cancer* and *metabolism of xenobiotics by cytochrome p450* for TN. For the Luminal B case, it was found that estrogen-related receptors α and γ (ERRα and ERRγ) up-regulate *MAOB* gene activity, whereas estrogen receptors α and β (ERα and ERβ) decrease stimulation in both a ligand-dependent and -independent manner^23^. High glycerol-3-phosphate acyltransferase (GPAM *glycerolipid metabolism pathway*) protein expression levels have been associated with hormone receptor negative status and with a better overall survival rates^24^. Moreover, *ACADL* gene has been reported to be related with ER positive, as well as with Luminal A and TN tumors^25^. Concerning *CDKN2A*, it has been indicated to be overexpressed in the majority of TN breast and HER2-enriched cancer carcinomas, while in cases of Luminal A and B type tumors was less frequently expressed^26^. Reduced gene expression of AKR1C1 appears to be unrelated to PR or ER status in breast tissue samples, as described in the literature^27^. Finally, two pathways were found common in all subtypes, including *cell communication* and *ecm receptor* interaction. Collagen family genes^22^ were found important, not only in the previous staging analysis, but also in the subtyping analysis too.

### Network inference and drug repurposing

The network patterns were further processed in order to investigate their contribution regarding the discovery of potential drugs for breast cancer subtypes and stages. Actually, genes that constitute the common network patterns from each subtype and stage were divided into up and down regulated, based on their Fold Change from the initial statistical analysis of the TCGA reference sets. The up and down regulated genes formed disease signatures that were queried in a well-established drug repurposing pipeline. Namely, LINCS-L1000 (http://www.lincscloud.org/) is the advanced version of cMap^28^ with significantly increased number of drug treatments, cell types and gene signatures based on L1000 high throughput technology. We used the LINCS-L1000 detailed report and we collected the top 20 drugs for each gene list with the most negative enrichment scores. The negative score suggests that the drugs are considered to be inhibitors. We then derived a list of 80 drugs (Table 5) regarding the stages (20 drugs per stage) and 80 drugs (Table 6) regarding the subtypes (20 drugs per subtype). DrugBank database^29^ (http://www.drugbank.ca/), as well as ChemSpider^30^ (www.chemspider.com) tool were used to find their chemical structures. The resulted drug lists (names and structures) were further evaluated via ChemBioServer^31^, a web application for searching, filtering and comparing drug structures. More specifically, we compared each top 20 drug list from LINCS with 25 known FDA-approved Breast cancer therapeutic drugs (http://www.cancer.gov/about-cancer/treatment/drugs/breast - Drugs Used to Treat Breast Cancer). This list includes Anastrozole, Capecitabine, Cyclophosphamide, Docetaxel, Doxorubicin, Epirubicin, Eribulin, Everolimus, Exemestane, Fluorouracil, Fulvestrant, Gemcitabine, Goserelin, Ixabepilone, Lapatinib, Letrozole, Megestrol, Methotrexate, Paclitaxel, Palbociclib, Pamidronate, Tamoxifen, Thiotepa, Toremifene and Vinblastine. Hierarchical clustering using tanimoto similarity (Soergel distance) was applied to each of the top 20 drug list from LINCS and the 25 known FDA-approved Breast cancer therapeutic drugs (Supplementary Figs 54–61). LINCS Drug Names were transformed into ChemSpider IDs (see Supplementary Table 1)

In synopsis, the unique drugs for the breast cancer stages were 63 and for the breast cancer subtypes 58, as we have located common drugs across them. Taking their union and removing the duplicates we conclude to a total of 105 repurposed drugs. Two of them (Gemcitabine and Palbociclib) are included in the list of the 25 known FDA-approved Breast cancer therapeutic drugs. We performed a Hypergeometric distribution test in order to find the statistical significance of this drug overlapping. More precisely, LINCS_L1000 database is comprised from 20,413 chemical reagents. Twenty two out of twenty five breast cancer drugs are also included in LINCS database. Finally, from the 105 drugs that were found from our analysis, the probability of finding two drugs to overlap with the Breast Cancer drugs in LINCS is 0.005471157, pointing out that there is statistical significance in their selection.

Interestingly, there have been found enough exclusive repurposed drugs for each stage: 12 for Stage I, 15 for Stage II, 13 for Stage III and 11 for Stage IV. Also, one repurposed drug (idarubicin) resulted in all Stages. Similar findings can be described for the subtype analysis. There have been found exclusively repurposed drugs: 7 for Luminal A, 12 for Luminal B, 14 for HER2 and 12 for TN. Accordingly, two repurposed drugs (etoposide and wortmannin) resulted in all Subtypes.

**Figure 8:**
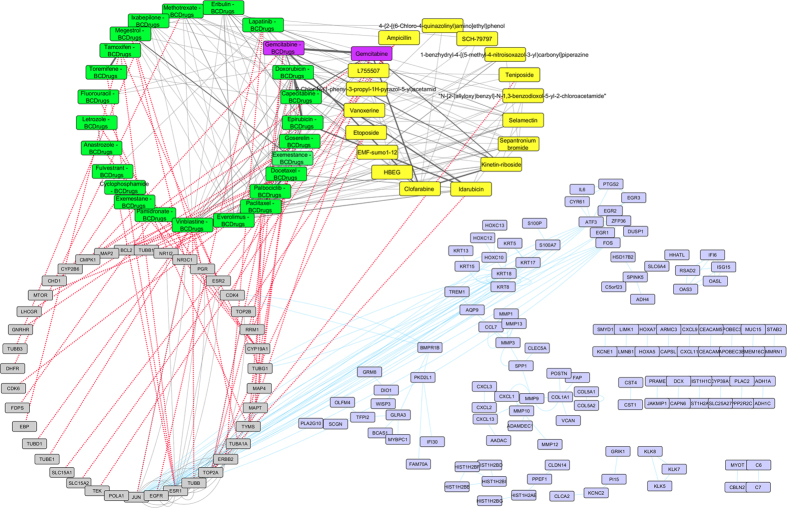
Super Network for breast cancer Stage I- consists of 4 sub-networks: 1) two drug – drug networks: with yellow cycle are represented the 20 drugs from LINCS and with green cycle the 25 therapeutic breast cancer drugs 2) drug – target network: grey round rectangles represent the target genes of all drugs (red dots edges) and 3) target - pattern genes network: physical interactions (blue edges) between target genes and genes from the network pattern (purple round rectangles). One out of the 25 FDA approved Breast cancer drugs (Gemcitabine), was found in the top 20 drug list from LINCS from breast cancer stage I (dark magenta).

To further examine the resulted drugs, we constructed a super network that combines each of the top 20 drugs extracted from our analysis with the 25 FDA approved breast cancer drugs, with their target genes and finally with the respective common network pattern. We used the DrugBank database (http://www.drugbank.ca/)^29^ in order to find the target genes of all drugs from LINCS and the 25 FDA approved Breast Cancer drugs. GeneMANIA^32^ plug-in of Cytoscape^33^ was applied to identify which genes from each pattern were physically interacting with the target genes. Our goal was to understand the correlations between drugs, drug targets and conserved co-expressed genes from a network-based view, in order to outline small paths that are of great importance in breast cancer stages and subtypes. Each network consists of four sub-networks, two drug – drug similarity networks, a drug – target network and a drug target – common pattern genes co-expression network, as shown in Figure 8 and the subsequent figures:

- **Drug – Drug networks:** In Figure 8 and the subsequent figures, the yellow cycles represent each top 20 drug list from LINCS and the green cycles the 25 FDA Breast cancer Drugs. Edges between the two cycles represent their structural similarity. As much thicker is the edge, the greater the similarity between the drugs. Only edges with similarity greater than 0.5 are presented.
- **Drug – Target network:** Grey cycles Figure 8 and the subsequent figures depict the target genes. As we described above, we found the corresponding target genes of the total drugs by means of the DrugBank database. Drug- target associations are represented with red dots.
- **Target – Pattern Genes:** Purple ellipses typify top 100 genes from each common network pattern. Blue edges represent physical interactions between target genes and genes from each common network pattern.

**Figure 9:**
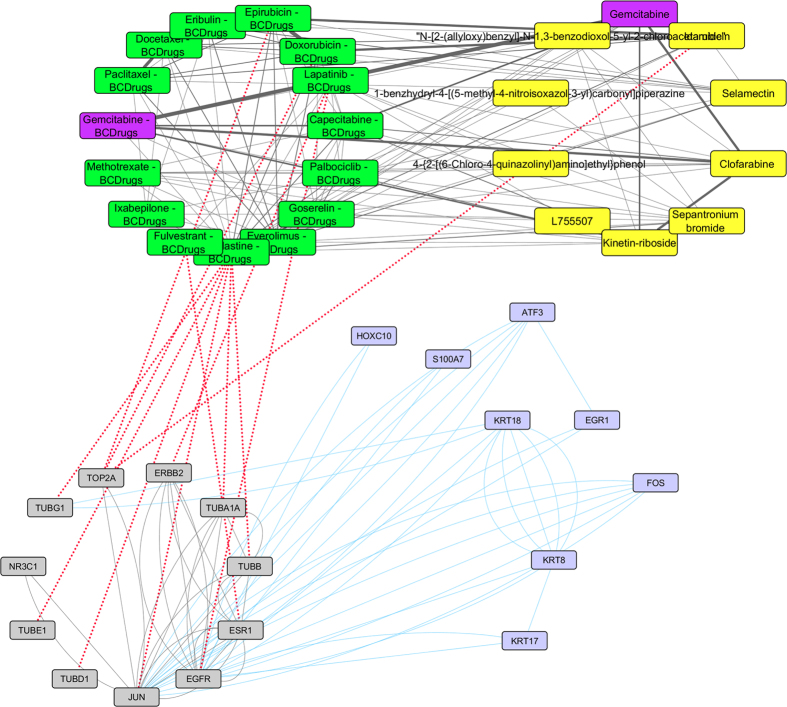
Highlighted target genes that physically interact with genes from the breast cancer stage I common network pattern and their corresponding repurposed drugs from LINCS, along with their structurally similar Breast cancer drugs.

As shown in Fig. 8, one drug out of 25 FDA approved Breast cancer drugs, Gemcitabine, was proposed as repurposed drug by the LINCS for breast cancer stage I. Furthermore, Gemcitabine is quite similar (tanimoto^31^ similarity greater than 80%) with Clofarabine and Kinetin-riboside (repurposed drugs from LINCS). Clofarabine is also an anti-cancer, antineoplastic chemotherapy drug and is classified as an antimetabolite. Kinetin riboside, a cytokinin riboside plant hormone with anticancer activity, has been used to study differentiation and apoptosis processes in myeloid leukemia cells, plant tumor cells (crown-gall) and other cancers. Moreover, Vinblastine – Breast Cancer drug was found to be greater than 60% structurally similar with Sepantronium bromide (repurposed drug from LINCS), which is a small-molecule proapoptotic agent with potential antineoplastic activity. Vinblastine has three target genes TUBA1A, TUBB and JUN. The latter was found to physically interact with three genes (ATF3, FOS and EGR1) of the breast cancer stage I network pattern (Fig. 9). As shown in Fig. 9, Idarubicin (repurposed drug from LINCS) was also found to be 85% structurally similar with Doxorubicin and Epirubicin and they are all topoisomerase 2 inhibitors (TOP2A).

**Figure 10:**
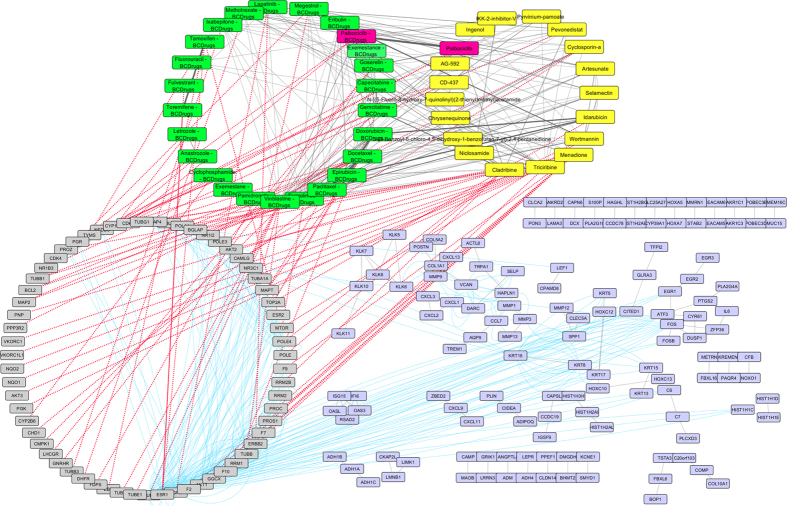
Super Network for breast cancer Stage II- consists of 4 sub-networks: 1) two drug – drug networks: with yellow cycle are represented the 20 drugs from LINCS and with green cycle the 25 therapeutic breast cancer drugs 2) drug – target network: grey round rectangles represent the target genes of all drugs (red dots edges) and 3) target - pattern genes network: physical interactions (blue edges) between target genes and genes from the network pattern (purple round rectangles). One out of the 25 FDA approved Breast cancer drugs (Palbociclib), was found in the top 20 drug list from LINCS from breast cancer stage II (deep pink).

**Figure 11:**
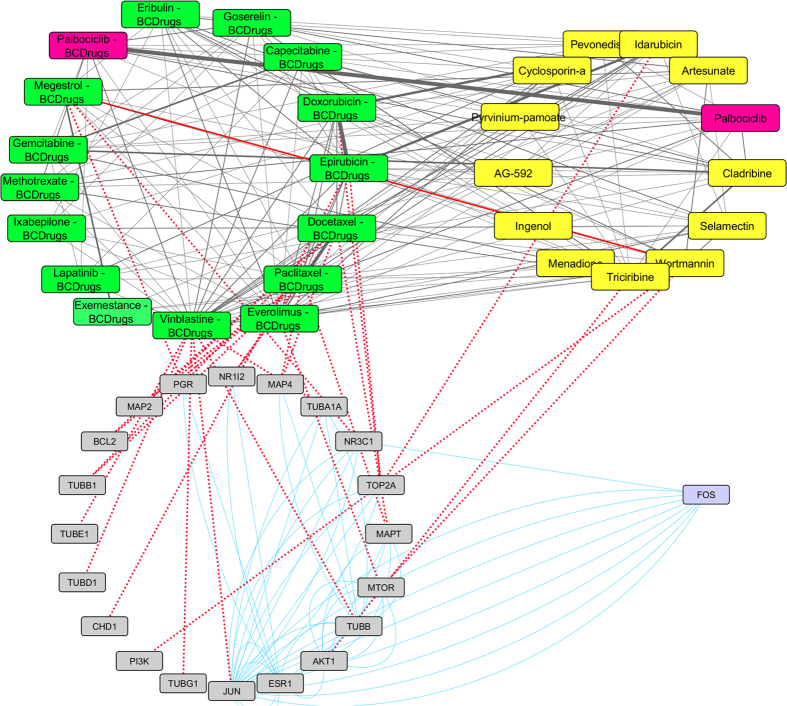
Highlighted target genes that physically interact with genes from the breast cancer stage II common network pattern and their corresponding repurposed drugs from LINCS, along with their structurally similar Breast cancer drugs.

As shown in Fig. 10, one drug out of 25 FDA approved Breast cancer drugs, Palbociclib, was found as repurposed drug from LINCS for breast cancer stage II. Gemcitabine (Breast cancer drug) has quite similar structure (greater than 70%) with Capecitabine (Breast cancer drug) and Cladribine (repurposed drug from LINCS) which is greater than 70% structurally similar with Triciribine (repurposed drug from LINCS) (Fig. 11). Cladribine is a chemotherapy drug used mainly to treat hairy cell leukaemia and occasionally other types of leukaemia and lymphoma. Moreover, Triciribine has a potential antineoplastic activity and inhibits the phosphorylation, activation, and signaling of Akt-1, -2, and -3, which may result to the inhibition of Akt-expressing tumor cell proliferation. As shown in Fig. 11, Megestrol (Breast cancer drug) has quite similar structure (greater than 70%) with Wortmannin (repurposed drug from LINCS). Worthmannin is a steroid metabolite of the fungi Penicillium funiculosum, Talaromyces wortmannii, which is a non-specific, covalent inhibitor of phosphoinositide 3-kinases (PI3Ks). It can also inhibit PI3K-related enzymes such as mTOR which is also target gene of Everolimus Breast cancer drug. Finally, the gene (FOS) from the breast cancer stage II pattern, physically interacts with *JUN*, a target gene of Vinblastine Breast cancer drug and with NR3C1, a target gene of Megestrol Breast cancer drug (Fig. 11).

**Figure 12:**
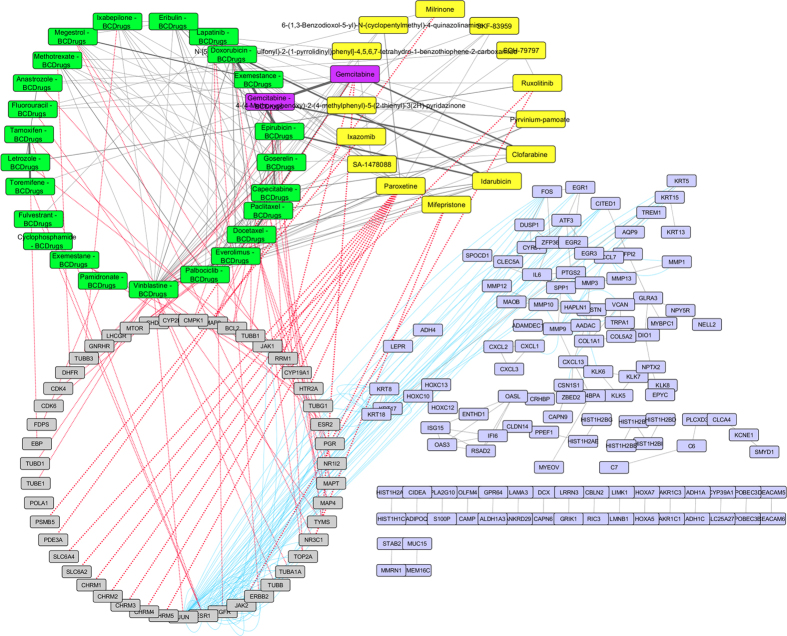
Super Network for breast cancer Stage III- consists of 4 sub-networks: 1) two drug – drug networks: with yellow cycle are represented the 20 drugs from LINCS and with green cycle the 25 therapeutic breast cancer drugs 2) drug – target network: grey round rectangles represent the target genes of all drugs (red dots edges) and 3) target - pattern genes network: physical interactions (blue edges) between target genes and genes from the network pattern (purple round rectangles). One out of the 25 FDA approved Breast cancer drugs (Gemcitabine), was found in the top 20 drug list from LINCS from breast cancer stage III (dark magenta).

**Figure 13:**
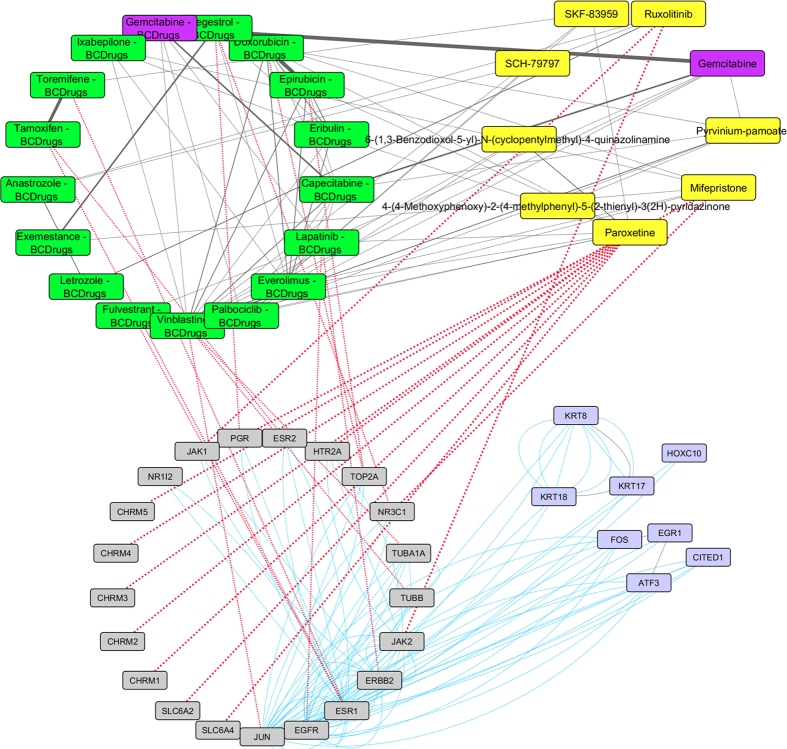
Highlighted target genes that physically interact with genes from the breast cancer stage III common network pattern and their corresponding repurposed drugs from LINCS, along with their structurally similar Breast cancer drugs.

As shown in Fig. 12, one drug out of 25 FDA approved Breast cancer drugs, Gemcitabine, was found as repurposed drug from LINCS for breast cancer stage III. Letrozole (Breast cancer drug) has similar structure (greater than 60%) with Ruxolitinib (repurposed drug from LINCS) a drug for the treatment of intermediate or high-risk myelofibrosis (Fig. 13). Furthermore, Pyrvinium-pamoate (repurposed drug from LINCS) was found to be greater than 60% structurally similar with Vinblastine (Breast cancer drug). Pyrvinium-pamoate (PP) is an FDA-approved antihelmintic drug that inhibits WNT signaling. Four genes from breast cancer stage III network pattern (KRT8, KRT17, KRT18 and HOXC10) physically interact with *EGFR*, a target gene of Lapatinib Breast cancer drug which is quite similar (greater than 50%) to Paroxetine (repurposed drug from LINCS). Paroxetine is an antidepressant drug of the selective serotonin reuptake inhibitor (SSRI) type and as shown in Fig. 13, is also structurally similar (greater than 60%) with 6-(1,3-Benzodioxol-5-yl)-N-(cyclopentylmethyl)-4-quinazolinamine (repurposed small molecule from LINCS).

**Figure 14:**
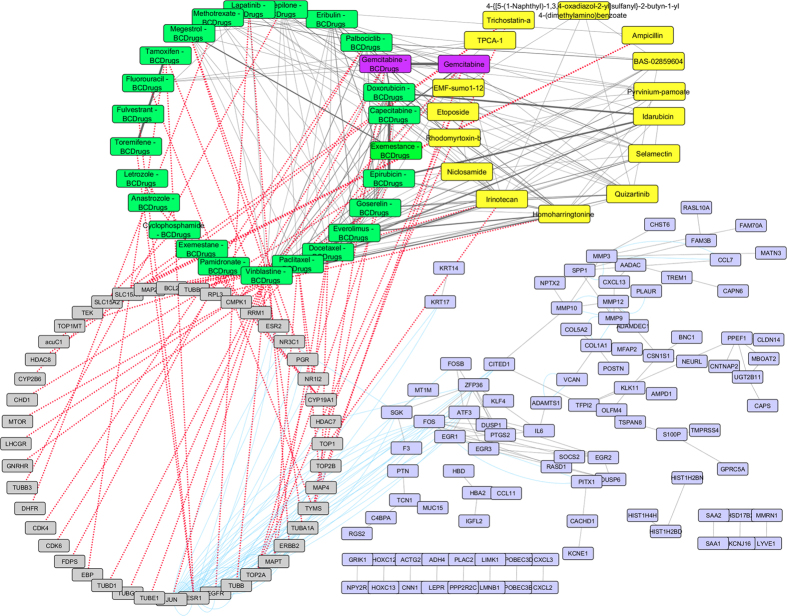
Super Network for breast cancer Stage IV- consists of 4 sub-networks: 1) two drug – drug networks: with yellow cycle are represented the 20 drugs from LINCS and with green cycle the 25 therapeutic breast cancer drugs 2) drug – target network: grey round rectangles represent the target genes of all drugs (red dots edges) and 3) target – pattern genes network: physical interactions (blue edges) between target genes and genes from the network pattern (purple round rectangles). One from the 25 FDA approved Breast cancer drugs (Gemcitabine), was found in the top 20 drug list from LINCS from breast cancer stage IV (dark magenta).

**Figure 15:**
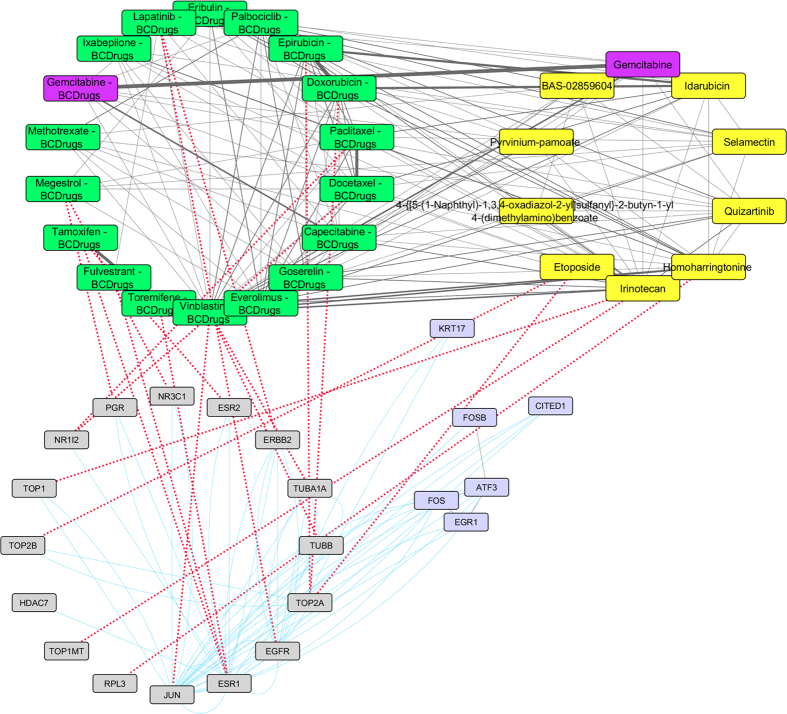
Highlighted target genes that physical interact with genes from the breast cancer stage IV common network pattern and their corresponding repurposed drugs from LINCS with the structurally similar Breast cancer drugs.

As in breast cancer stages I and III one drug out of 25 FDA approved Breast cancer drugs – Gemcitabine – was found as repurposed drug from LINCS for breast cancer stage IV (Fig. 14). A repurposed drug from LINCS – Homoharringtonine was found to be structurally similar with Everolimus and Vinblastine Breast cancer drugs (greater than 70%). On the other hand, as shown in Fig. 15, Vinblastine has similar structure (greater than 70%) with Irinotecan (repurposed drug from LINCS) which is 63% structurally similar with Quizartinib. Irinotecan is a chemotherapy drug and it is used to treat bowel cancer and it is also topoisomerase I inhibitor (Fig. 15). Quizartinib is a small molecule receptor tyrosine kinase inhibitor and it is used to treat acute myeloid leukaemia. Moreover, Selamectin (repurposed drug from LINCS) has greater than 60% similar structure with Eribulin Breast cancer drug. Selamectin is a topical parasiticide and antihelminthic used on dogs and cats.

**Figure 16:**
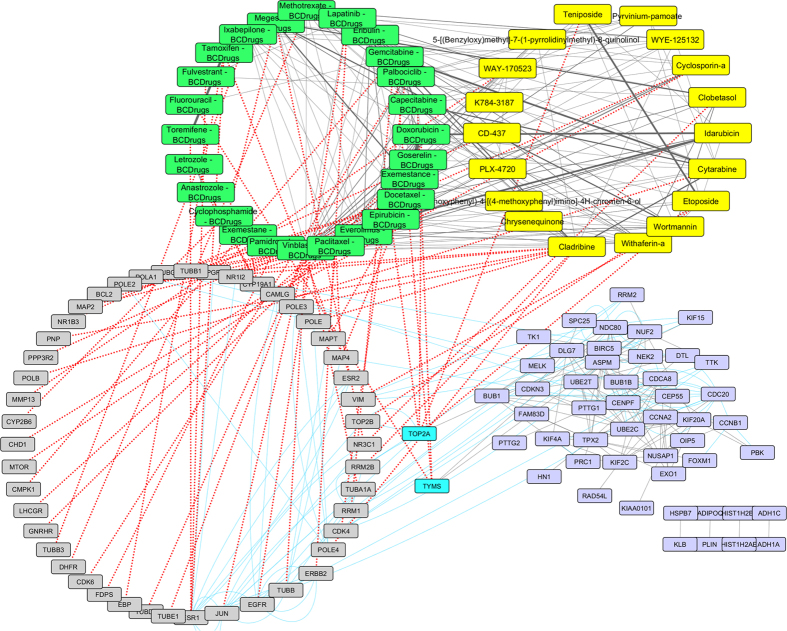
Super Network for Triple Negative breast cancer - consists of 4 sub-networks: 1) two drug – drug networks: with yellow cycle are represented the 20 drugs from LINCS and with green cycle the 25 therapeutic breast cancer drugs 2) drug – target network: grey round rectangles represent the target genes of all drugs (red dots edges) and 3) target – pattern genes network: physical interactions (blue edges) between target genes and genes from the network pattern (purple round rectangles). Two target genes are also in the Triple Negative common network pattern (turquoise).

**Figure 17:**
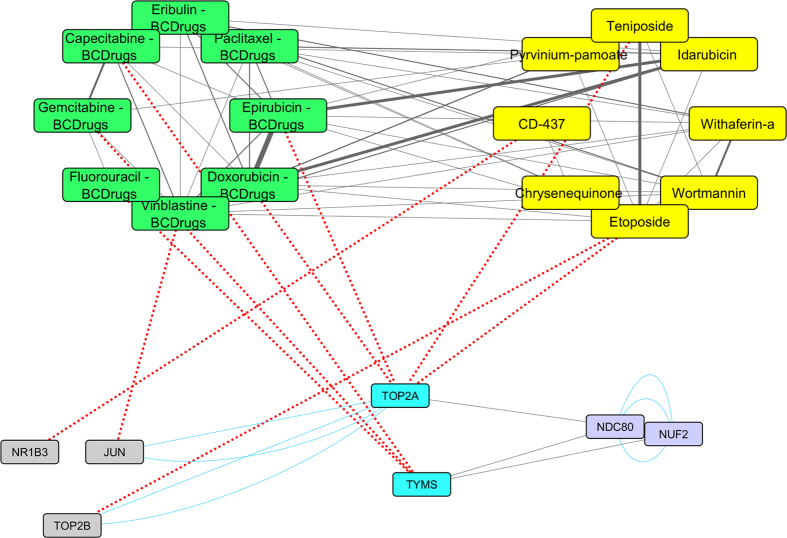
Highlighted target genes that physical interact with genes from the Triple Negative breast cancer subtype common network pattern and their corresponding repurposed drugs from LINCS with the structurally similar Breast cancer drugs.

As shown in Figs 16, 17 two target genes (TOP2A and TYMS) are also involved in the Triple Negative pattern. TOP2A is a target gene of two Breast cancer drugs (Epirubicin and Doxorubicin) and of two repurposed drugs from LINCS (Etoposide and Teniposide) which are greater than 80% structurally similar. TOP2A physically interacts with two other target genes – JUN and TOP2B (Fig. 17). TYMS is also a target gene of three Breast cancer drugs (Fluorouracil, Gemcitabine and Capecitabine) and physically interacts with two genes from the Triple Negative pattern -NUF2 and NDC80 (Fig. 17).

**Figure 18:**
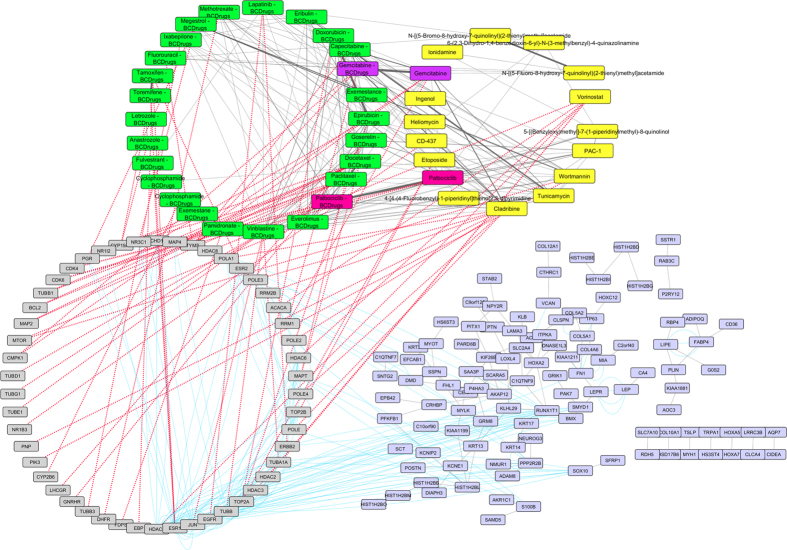
Super Network for Luminal A breast cancer subtype- consists of 4 sub-networks: 1) two drug – drug networks: with yellow cycle are represented the 20 drugs from LINCS and with green cycle the 25 therapeutic breast cancer drugs 2) drug – target network: grey round rectangles represent the target genes of all drugs (red dots edges) and 3) target – pattern genes network: physical interactions (blue edges) between target genes and genes from the network pattern (purple round rectangles). Two from the 25 FDA approved Breast cancer drugs (Gemcitabine and Palbociclib), was found in the top 20 drug list from LINCS from Luminal A breast cancer (dark magenta and deep pink respectively).

**Figure 19:**
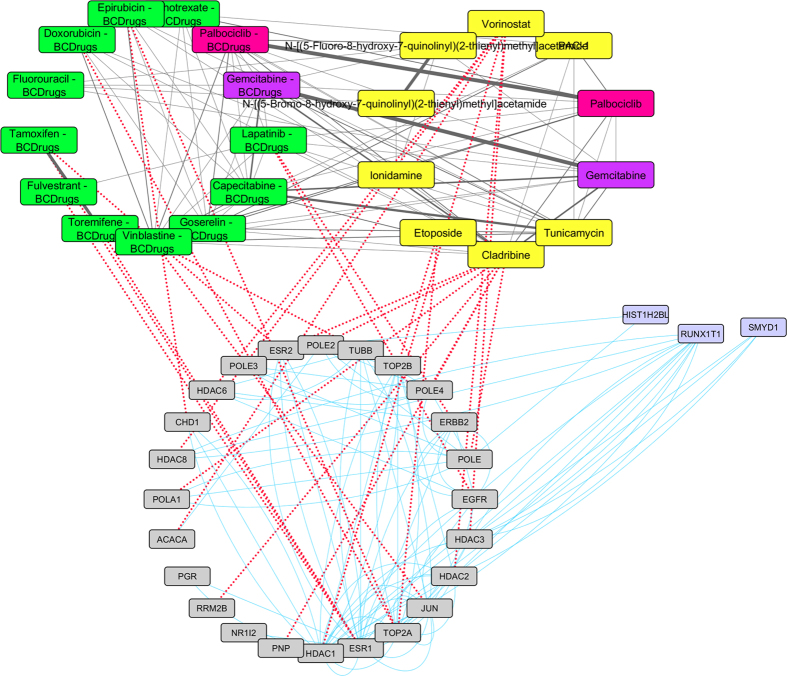
Highlighted target genes that physical interact with genes from the Luminal A breast cancer subtype common network pattern and their corresponding repurposed drugs from LINCS with the structurally similar Breast cancer drugs.

As shown in Fig. 18 two drugs out of 25 FDA approved Breast cancer drugs – Gemcitabine and Palbociclib – were also found as repurposed drugs from LINCS for breast cancer Luminal A (Fig. 18). Two genes from the Luminal A network pattern physically interact with four genes that involved in Histone deacetylases class (HDAC1, HDAC2, HDAC3 and HDAC8), which are target genes of Vorinostat (repurposed drug from LINCS). Vorinostat is a member of a larger class of compounds that inhibit histone deacetylases (HDAC) and it is used to treat cutaneous T cell lymphoma (CTCL). Furthermore, HIST1H2BL from the Luminal A pattern physically interacts with POLE and POLE2, which are target genes of Cladribine (repurposed drug from LINCS). Cladribine was quite structurally similar (greater than 70%) to Gemcitabine Breast cancer drug and Tunicamycin (greater than 60%), which is a repurposed drug from LINCS (Fig. 19).

**Figure 20:**
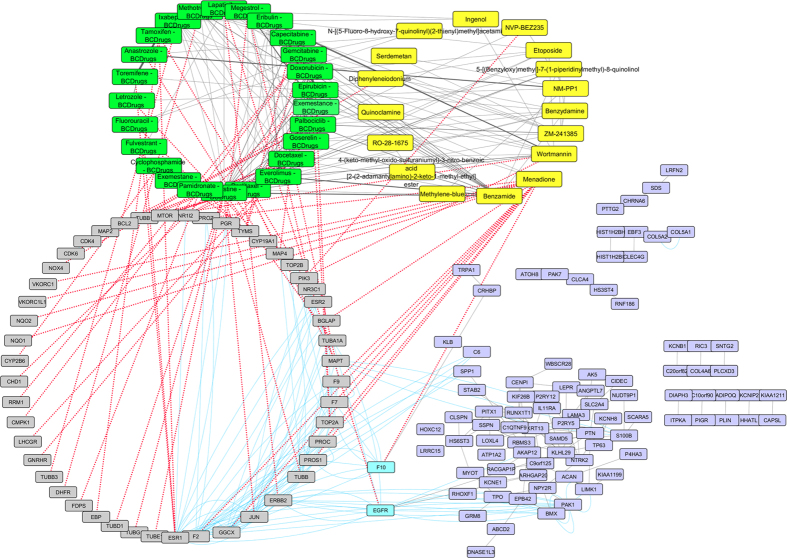
Super Network for Luminal B breast cancer subtype- consists of 4 sub-networks: 1) two drug – drug networks: with yellow cycle are represented the 20 drugs from LINCS and with green cycle the 25 therapeutic breast cancer drugs 2) drug – target network: grey round rectangles represent the target genes of all drugs (red dots edges) and 3) target – pattern genes network: physical interactions (blue edges) between target genes and genes from the network pattern (purple round rectangles). Two target genes are also in the Luminal B common network pattern (turquoise).

**Figure 21:**
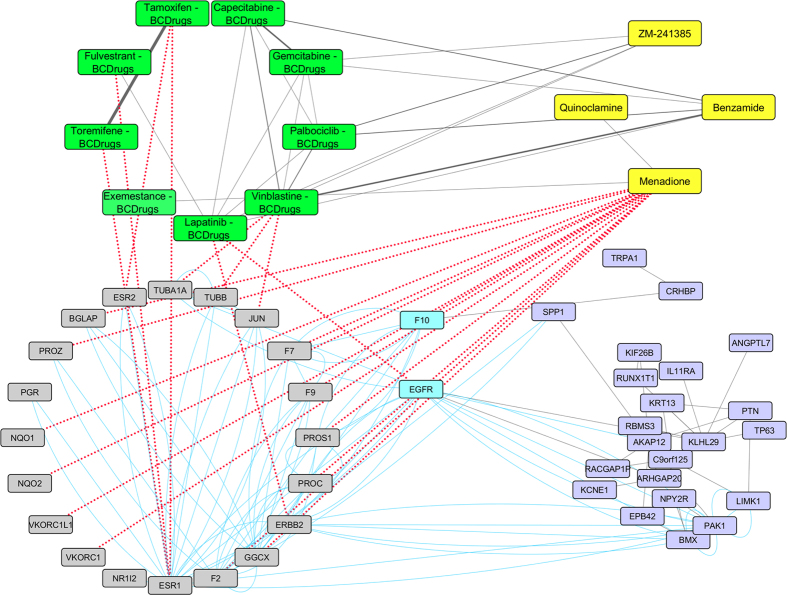
Highlighted target genes that physical interact with genes from the Luminal B breast cancer subtype common network pattern and their corresponding repurposed drugs from LINCS with the structurally similar Breast cancer drugs.

As shown in Figs 20, 21 two target genes (F10 and EGFR) are also involved in the Luminal B pattern. F10 is one out of 13 target genes of Menadione (repurposed drug from LINCS). Menadione is a synthetic chemical compound that used as a nutritional supplement because of its vitamin K activity. Furthermore, EGFR with ERBB2 are target genes of Lapatinib - Breast cancer drug (Fig. 21). Moreover, Benzamide (repurposed drug from LINCS) was found to be structurally similar (greater than 70%) to Vinblastine (Breast cancer drug). Benzamide is an off-white solid and it is used in a wide range of therapeutics including analgesics, antiemetics, antipsychotics and other agents. Finally, ZM-241385 (repurposed drug from LINCS) has similar structure (more than 60%) with Palbociclib Breast cancer drug (Fig. 21). ZM-241385 is an antagonist ligand and may be useful as a treatment for Alzheimer’s and Parkinson’s disease.

**Figure 22:**
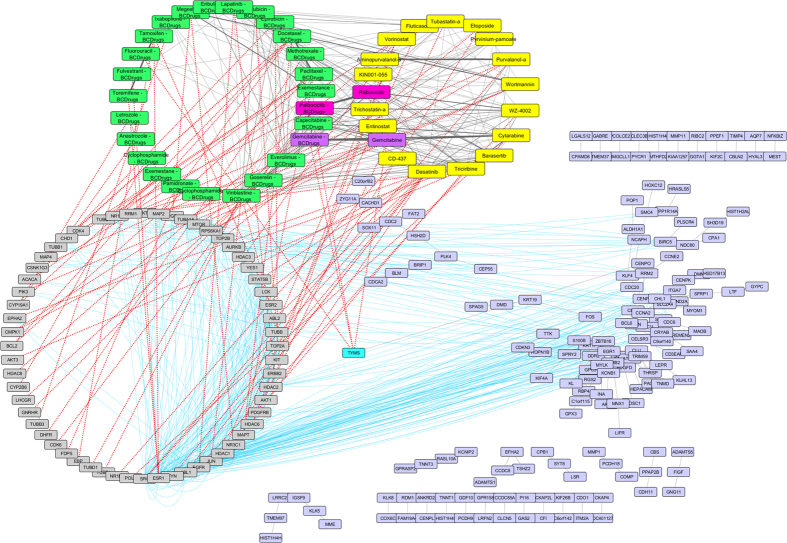
Super Network for HER2 breast cancer subtype- consists of 4 sub-networks: 1) two drug – drug networks: with yellow cycle are represented the 20 drugs from LINCS and with green cycle the 25 therapeutic breast cancer drugs 2) drug – target network: grey round rectangles represent the target genes of all drugs (red dots edges) and 3) target – pattern genes network: physical interactions (blue edges) between target genes and genes from the network pattern (purple round rectangles). Two from the 25 FDA approved Breast cancer drugs (Gemcitabine and Palbociclib), were found in the top 20 drug list from LINCS from HER2 breast cancer (dark magenta and deep pink respectively). One target gene is also in the HER2 common network pattern (turquoise).

**Figure 23:**
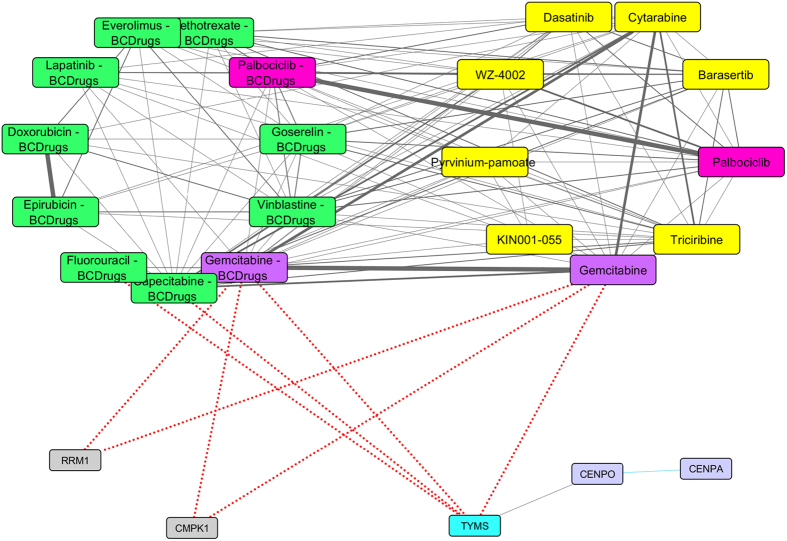
Highlighted target genes that physical interact with genes from the HER2 breast cancer subtype common network pattern and their corresponding repurposed drugs from LINCS with the structurally similar Breast cancer drugs.

As shown in Figs 22, 23, target gene (TYMS) is also involved in the HER2 pattern. TYMS physically interacts with two genes from the HER2 pattern -CENPO and CENPA and is a target gene of three Breast cancer drugs (Fluorouracil, Capecitabine and Gemcitabine). Gemcitabine, as previously described, is a Breast cancer drug that was also found as a repurposed drug from LINCS for HER2 pattern. It is more than 80% structurally similar to the repurposed drug Cytarabine, which is a chemotherapy agent that used mainly in the treatment of cancers of white blood cells. Furthermore, Palbociclib is also a Breast cancer drug that was found from the drug repurposing analysis of HER2 pattern. It has similar structure - 75% with WZ-4002 repurposed drug, which is a novel mutant-selective inhibitor of EGFR. Finally, both Palbociclib and WZ-4002 are structurally similar to Dasatinib (more than 60%), which is a cancer drug used to treat acute lymphoblastic leukemia.

## Discussion

In the present work, we used eleven network inference methods and one ensemble scheme to reconstruct gene co-expression networks, in order to examine their contribution in identifying significant genes and gene-gene links related to different breast cancer stages and subtypes. During this assessment, we demonstrated that in most cases of breast cancer stages and subtypes, the statistical co-expression networks produce either similar or more enriched lists with significant genes (in terms of maximum classification accuracy achieved) for each breast cancer stage and subtype than the conventional statistical approach or the networks based solely on the biological information extracted from the literature. Actually, the dominance of statistical networks is profound in the analysis of breast cancer subtypes, whereas in the case of stage analysis, the simple statistical method (Initial) and the signaling network based on inhibition (SN_I) give slightly better (almost equivalent) scores than statistical networks.

Furthermore, our analysis concluded to eight network patterns, four for the stages (I, II, III and IV) and four for the subtypes (Triple Negative, Luminal A, Luminal B and HER2). Additionally, we further analyzed the gene patterns, in order to investigate potential mechanisms and drugs for breast carcinomas staging and subtypes. As described in the previous section, we have found four exclusive stage-related pathways including *phenylalanine metabolism* for Stage II, *peroxisome proliferator-activated* (PPAR) *signaling pathway* and *glycolysis and gluconeogenesis* for Stage III and *toll like receptor signaling pathway* for Stage IV. PPAR *signaling pathway* has been implicated in the pathology of numerous diseases, including obesity, diabetes, atherosclerosis, and cancer. More specifically, PPAR *signaling pathway* has been reported as a possible important predictor of breast cancer response to neoadjuvant chemotherapy^34^. Five dehydrogenase (ADH) isoenzymes and aldehyde dehydrogenases (ALDH) genes from the breast cancer Stage III network pattern were involved in the *glycolysis and gluconeogenesis pathway*. It has been reported that patients with advanced breast cancer had changes in the activity of ADH isoenzymes and ALDH^35^. Furthermore, from the breast cancer Stage IV pattern, we have found an exclusive pathway - *toll like receptor signaling pathway*, for which it is well known that supports *in vitro* and *in vivo* tumor cell growth^36^. For the case of breast cancer subtypes, we have found seven exclusive subtype-related pathways, including *glycine serine and threonine metabolism pathway* for Luminal B, *glycerolipid metabolism*, *fatty acid metabolism*, *complement and coagulation cascades* and *bladder cancer* for HER2 and *small cell lung cancer* and *metabolism of xenobiotics* by cytochrome p450 for Triple Negative. *Hyperactivation Glycine serine and threonine metabolism pathway* drives to oncogenesis and recent developments support that this pathway may provide novel opportunities for drug development and biomarker identification of human cancers^37^. It has been found that HER2 overexpression increases translation of fatty acid synthase (FASN) and FASN overexpression markedly increases EGFR and HER2 signaling, which results to enhanced cell growth. The overexpression of FASN has been associated with poor prognosis and may be a novel therapeutic target in HER2-overexpressing breast cancer cells^38^. Moreover, from the Triple Negative pattern we found the metabolism of *xenobiotics by cytochrome p450 pathway*. Cytochromes P450 (CYPs) play a pivotal role in cancer formation and cancer treatment as they participate in the inactivation and activation of anticancer drugs^39^.

Most of the specific mechanisms per subtype and stage are related to cellular community, signaling, cell growth and death, immune and endocrine systems, carbohydrate, lipid and amino acid metabolism, as well as xenobiotics biodegradation and metabolism. Furthermore, all the derived network patterns include genes found in breast cancer specific regions of significant somatic copy number alterations (SCNA)^16^. These results are fully aligned to the up-to-date recognized cancer hallmarks related to cell growth, metabolism, immune system, inflammation and genome duplication^40^.

The resulted network patterns were also analyzed by means of LINCS drug reposition pipeline, so as to propose potential anticancer drugs for breast cancer stages and subtypes. Based on this analysis, we have concluded to 63 potential unique drugs for breast cancer stages and 58 for breast cancer subtypes. In order to elucidate potential anti-breast cancer properties of these drugs, we compared their molecular structure similarity against 25 drugs of clinical use. Two out of these 25 drugs (Gemcitabine and Palbociclib) were also found as repurposed drugs from LINCS. In Stage I, two repurposed drugs Clofarabine and Kinetin-riboside were found to be structurally similar to Gemcitabine. Clofarabine seems to have potential efficacy in epigenetic therapy of solid tumours, especially at early stages of carcinogenesis^41^. Furthermore, Kinetin-riboside is an anti-proliferative agent which induces apoptosis in certain cell lines. Mechanistic studies show that Kinetin riboside may cause a cell cycle arrest at the G2/M phase. Coconut milk contains kinetin riboside and is thought to have the potential to inhibit the progression of many cancers, including prostate, colon and breast cancer. One study found that carcinogen-induced mammary tumors in mice were reduced by coconut oil too (http://foodforbreastcancer.com/). Moreover, in Stage I, Sepantronium bromide (repurposed drug from LINCS) has been found similar with Vinblastine Breast cancer drug and Idarubicin with Doxorubicin and Epirubicin respectively. Sepantronium bromide (survivin inhibitor YM155) has been investigated as potential drug of breast cancer subtypes^42^. Finally, Idarubicin was also investigated for its mechanism of action in breast cancer and it has been reported that is effective in elderly breast cancer patients^43^. For Stage II, Cladribine (repurposed drug) was found to be structurally similar with Triciribine (repurposed drug) and Gemcitabine and Capecitabine Breast cancer drugs. In clinical trial (June, 2015) triciribine phosphate, combined with paclitaxel, doxorubicin hydrochloride, and cyclophosphamide, used as a treatment to patients with stage IIB-IV breast cancer (https://clinicaltrials.gov).

Moreover, Wortmannin (repurposed drug) was found structurally similar to Megestrol. It has been reported that Worthmannin induces MCF-7 cell death^44^,^45^. In Stage III Ruxolitinib and Pyrvinium-pamoate repurposed drugs from LINCS have been found structurally similar with Letrozole and Vinblastine Breast cancer drugs respectively. An ongoing clinical trial (October, 2015) has compared the overall survival of women with advanced (Stage III) or metastatic (Stage IV) HER2-negative breast cancer who received treatment with Capecitabine in combination with Ruxolitinib versus those who received treatment with Capecitabine, solely (https://clinicaltrials.gov). Additionally, Pyrvinium-pamoate is reported to be a potential drug for aggressive breast cancer^46^. Finally, in Stage IV, Homoharringtonine (repurposed drug) was found to be structurally similar with Everolimus and Vinblastine Breast cancer drugs, and Irinotecan (repurposed drug) with Vinblastine Breast cancer drug and Quizartinib repurposed small molecule. Irinotecan has been examined in a clinical trial in Phase II in order to find its objective response rate in patients with metastatic breast cancer (Stage IV) (https://clinicaltrials.gov).

In case of repurposed drugs for breast cancer subtypes, we have found that Etoposide and Teniposide (repurposed drugs) as structurally similar with two Breast cancer drugs Epirubicin and Doxorubicin in Triple Negative subtype. The latter four drugs are topoisomerase ii inhibitors (TOP2A), while Etoposide has been found as effective drug in Chinese women with heavily pretreated metastatic breast cancer^47^. TOP2A is also an up-regulated gene in the Triple Negative pattern. As TOP2A, TYMS is also a gene from the Triple Negative pattern which is a target gene of three Breast cancer drugs (Fluorouracil, Gemcitabine and Capecitabine). TOP2A and TYMS were found significant up-regulated genes in Triple Negative breast cancer cells, as compared to normal cells^48^. In Luminal A, the target genes of Vorinostat, physically interact with two genes (RUNX1T1 and SMYD1) from the Luminal A pattern. It has been reported that Vorinostat in combination with Tamoxifen, may treats patients with hormone therapy-resistant breast cancer^49^. In Luminal B, F10 and EGFR genes from Luminal B pattern are also target genes of Menadione (repurposed drug from LINCS) and Lapatinib Breast cancer drug. Menadione has been examined on its antiproliferative action on breast cancer cells^50^. Finally in HER2 subtype, Palbociclib is also a Breast cancer drug that was found from the drug repurposing analysis of HER2 pattern. It has quite similar structure with WZ-4002 repurposed drug, which is a novel mutant inhibitor of EGFR. Both Palbociclib and WZ-4002, are structurally similar to Dasatinib – a repurposed drug from LINCS for the HER2 subtype. In a recent study, Dasatinib (Src inhibitor) has been reported to have anti-tumor effect in HER2 positive breast cancer with Trastuzumab resistance^51^.

Finally, the action of the remaining mechanisms and drugs found from LINCS may be further investigated, since they have been derived from significantly relevant genes related to breast cancer stages and subtypes.

## Methods

### Datasets and preprocessing

#### Reference Set

TCGA mRNA (microarray) gene expression data for Breast Invasive Carcinoma cases are obtained from Firehose (http://gdac.broadinstitute.org/). From a total 587 samples (526 primary solid tumor samples and 61 primary solid normal samples - 17.814 genes), we have selected a subset of tumor data containing information regarding breast cancer staging, HER2, ER and PR status with their corresponding normal samples (Table 7). Concerning staging, selection of stages *I*, *II*, *III* and *IV* was performed based on the clinical records accompanying each sample, while for the case of subtyping, the selection was performed as followed: (i) Luminal A for ER+ and/or PR+, HER2-, (ii) Luminal B for ER+ and/or PR+, HER2+, (iii) HER2 for ER-, PR-, HER2+ and (iv) Triple Negative for ER-, PR-, HER2-. The eight distinct TCGA dataset were statistically analyzed with the *LIMMA* R package in order to select the Differentially Expressed Genes (DEGs) in breast cancer samples compared with the normal ones^52^. The top 1000 genes of each sub-dataset with p-value < 0.01 and q-value < 0.01, sorted based on their log Fold Change absolute value, were used as the reference sets in our analysis.

#### Validation Sets

We searched in Gene Expression Omnibus (http://www.ncbi.nlm.nih.gov/geo/) accessed on 19 November 2015 using the following query:

[Title] “Breast cancer” OR “breast tumor” OR “breast carcinomas” AND [Organism] “Homo Sapiens” AND [Filter] “Expression profiling by array” AND [All Fields] “Normal” NOT [All Fields] “Therapy” NOT [All Fields] “Treatment” NOT [All Fields] “Drug” AND *

where * was set as “Triple Negative”[All Fields] OR “ Basal like”[All Fields] for the case of Triple Negative, “Luminal A”[All Fields] for the case of Luminal A, “Luminal B”[All Fields] for the case of Luminal B, “HER2”[All Fields] OR “ERBB2”[All Fields] for the case of HER2 and “Stage”[All Fields] OR “TNM”[All Fields] for the case of staging.

We concluded to 7 independent GEO datasets after excluding the ones containing samples either generated using treated cells or taken from peripheral blood or containing siRNAs, as shown in Table 8.

To be able to analyze together all datasets (reference and validation sets) we normalized the expression values on a scale from 0 to 1 and we imputed the missing values using the *impute* R package^53^.

### Network Reconstruction

We have examined 3 major categories of statistical network inference methods: (i) Mutual Information-based methods, (ii) Correlation-based methods and (iii) Tree-based methods. Also, we utilized Biological information-based network methods and one ensemble scheme using all statistical network inference methods.

#### Mutual Information-based methods

Mutual Information (MI) is a nonlinear measure used to measure equally linear and nonlinear correlations. Mutual information represents a general information-theoretic approach to determine the statistical dependence between variables^54^. MI between two discreet random variables *X*, *Y* jointly distributed according to *p(x, y)* is given by:


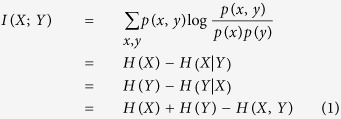


where *H(X)*, *H(Y)* is the entropy of the discreet variable *X* and *Y* and *H(X,Y)* the joint entropy.

The basic idea is to calculate the mutual information values of all pairs for a given gene expression profile and declare mutual information values as relevant if their corresponding value is larger than a given threshold. The resulting network is constructed based on this threshold by including an edge between two genes and a score as the weight of this edge^55^. Weights can be calculated using various algorithms. In this work we applied 6 mutual information based algorithms:

The first two algorithms Aracne.a and Aracne.m (Algorithm for the Reconstruction of Accurate Cellular Networks)^56^ are functions that implement ARACNE algorithm to reconstruct gene interaction networks. This algorithm examines each triplet of nodes with corresponding edges, independently, and removes the weakest:

For Aracne.a:


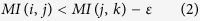


and


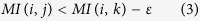


For Aracne.m (multiplicative model):


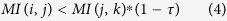


and


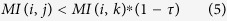


where MI is the matrix of the mutual information and *ε*, *τ* additive tolerances which are used for the impact of the MI estimation. We used the default values *e* = 0.05 and *τ* = 0.15 as suggested in^57^.

The third algorithm, called CLR (Context Likelihood or Relatedness Network)^58^, derives a score for each gene pair after the calculation of the mutual information. More specifically, for *X*_*i*_ and *X*_*j*_ it calculates the value:


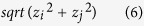


for each pair of variables *i*, *j* where:


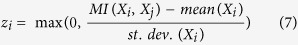


An adaptive background correction step is used in order to eliminate false correlations and indirect influences as described in^58^.

The fourth algorithm is the MRNET (Maximum Relevance Minimum Redundancy)^59^. This algorithm infers a network of interactions between genes by using a forward selection strategy to identify a maximally independent set of neighbors for every variable. MRNET starts by choosing the variable *Xi* with the largest shared information with the objective of *Y*. Then, it repeats the investigation of all selected variables by taking the *Xk* that maximizes the difference:


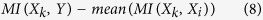


The process stops when the value becomes negative.

MRNETB (Maximum Relevance Minimum Redundancy Backward) is an improved version of the previous network inference algorithm MRNET. As stated above, MRNET applies a forward selection strategy to identify a set of neighbors for every variable. However, forward selection methods suffer in performance if the first neighbor is chosen incorrectly. On the other hand, MRNETB implements a combination of backward elimination and a sequential replacement procedure keeping the same computational cost^59^.

The final algorithm used in this category is C3NET, which focuses in the detection of a significant maximum mutual information network in a way that two genes are only connected with each other if their shared significant mutual information value is maximal at least for one of these two genes with respect to all other genes.

The C3net algorithm is divided into two main steps. In the first step, C3net eliminates the non – significant mutual information values and in the second step it keeps the maximum mutual information value for each pair of genes^60^.

#### Correlation based methods

Four different algorithms were used in order to construct gene interaction networks based on the correlation/partial correlation of gene pairs.

Sparse undirected graphical models can be estimated by the use of L1 (Lasso- Least absolute shrinkage and selection operator) regularization^61^. It is assumed that gene expressions have a multivariate Gaussian distribution with mean μ and covariance matrix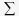. It is shown that if the component (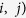 of the inverse matrix 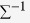
is zero, then variables *i* and *j* are conditionally independent, given the other variables. Therefore, co-expression networks can be constructed by estimating the inverse of covariance matrix through L1. Adaptive Lasso considers the Lasso with penalty weights. It is considered that adaptive Lasso procedure is consistent for high-dimensional model selection in graphical Gaussian models under rather general and less restrictive conditions^62^.

GeneNet is a statistical learning algorithm based on the method of Schaefer and Strimmer^63^ which allows the assessment of Graphical Gaussian Models (GGMs). GeneNet is an extension of GGMs and is implemented in two stages: In the first stage the network converts the correlation (correlation network) to a partial correlation (partial correlation network) which is a non-directional graph showing the linear compounds. In the second stage it converts the undirected graph in partially directed assessing the log ratio pairs of individual variability (partial variances).

The second algorithm is WGCNA (Weighted correlation network analysis)^64^. This algorithm is used to find groups of genes with high correlation. It computes an adjacency matrix using the Spearman correlation:


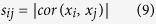


We calculated correlations across each pair *(xi, xj)* of genes.

#### Tree based method

In the third category, GENIE3 algorithm splits the problem of network construction between k genes into k regression sub-problems. GENIE3 applies tree based methods Random Forest^65^ or Extra Trees^66^ in each of the regression problems in order to find the expression pattern of one of the genes from the expression patterns of all the other genes^67^.

Summarizing, we have used 11 network inference methods (Table 9) to reconstruct gene co-expression networks for each dataset including the top 1000 DEGs from the TCGA dataset. All the selected methods are implemented in R packages. Specifically, Aracne.a, Aracne.m, CLR, MRNET are implemented in the PARMIGENE (PARallel Mutual Information calculation for GEne NEtwork reconstruction) R-package which provides a parallel estimation of the mutual information based on entropy estimates from k-nearest neighbors distances^57^. MRNETB is implemented in MINET (Mutual Information NETworks) R-package^68^. C3net is included in the homonym R-package C3NET^69^. Lasso and Adaptive lasso regression methods are included in PARCOR R-package which estimates the matrix of partial correlations based on different regularized regression methods^62^. GeneNet and WGCNA are included in ENA (Ensemble network aggregation) R-package^70^ while GENIE3 is implemented through the homonym R-package GENIE3^67^.

#### Biological Information-based Networks

We have used the Cytoscape^33^ platform and more specifically the GeneMania plug-in^32^ to reconstruct a gene network using biological information (Table 9). The GeneMANIA algorithm inside the homonymous plugin obtains information from a combination of potentially heterogeneous sources. This plug-in uses a large data set unifying functional networks comprising approximately 800 networks for 6 organisms including Homo sapiens. Using the Homo sapiens network we constructed a sub – network for the top 1000 DEGs from the TCGA dataset merging 5 Network types:

1. **Co-expression**: Two genes interact if their expression levels are similar across conditions in a gene expression study. Most of these data are collected from the Gene Expression Omnibus (GEO) and are associated with a publication.
2. **Physical Interaction**: Protein-protein interactions- two gene products interact if they were found to interact in a protein-protein interaction study.
3. **Genetic interaction**: Two genes functionally interact if the effects of perturbing one gene were found to be modified by perturbations to a second gene.
4. **Co-localization:** Two genes interact if they are expressed in the same tissue, or if their gene products are both identified in the same cellular location.
5. **Pathways:** Two gene products interact if they participate in the same reaction within a pathway.

We also used the manually curated human signaling network^71^ (http://www.cancer-systemsbiology.org/dataandsoftware.htm) based on the literature since 2005 (Version 6). The signaling network contains more than 6,000 proteins and 63,000 relations from different data sources including BioCarta, CST Signaling pathways, Pathway Interaction database (PID), iHOP, and many review papers on cell signaling. The signaling network comprised of three different relations (activation, inhibition and physical interactions). This network was used not only as a whole network (all relations), but was further divided into three sub-networks based on the different relation types.

#### Ensemble Scheme based on Statistical Network Inference Methods - Voting

We have created a union unique gene list based on the different top 100 re-ranked gene lists from the eleven statistical network inference methods. Based on the highest frequency of the appearance, the minimum mean rank and the minimum coefficient of variation across all statistical network inference methods we selected the top 100 genes.

### Gene re-ranking using underlying networks

In order to investigate the influence of the reconstructed 17 gene networks (12 statistically and 5 biologically inferred) on gene prioritization, we applied a method that allows for a custom network selection combining the log fold change absolute values with the selected underlying network in order to re-rank the initial DEGs^72^. The basic idea of the method is the reconciliation of the gene expression values taking into account an underlying gene network. This approach is available as part of the Biorithm software in the Network Reconciliation package^72^.

More specifically, considering the underlying network as a graph *G* with a set of nodes *V* and a set of edges *E*, the relation of genes *u*, *v* to G is annotated as *(u, v)* and the weight of each edge is annotated as W. The number of all neighboring nodes of v in the graph *G* is *Nv* and the total weight of the neighboring nodes of *v* in *G* is *dv*. The degree of perturbation *S(v)* (initially the value of the node *v* in *G*) is computed as the absolute value of the gene’s log Fold Change.

So, if two genes *u* and *v* are connected by an interaction in G, then *S(u)* and *S(v)* should maintain similar values. Then a re-calculation of the value *p(v)* between 0 and 1 for every node 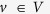
is performed, taking into account two restrictions:

1. *p(v)* remains close to v’ s initial value *S(v)*
2. *p(v)* is similar to *p(u)* for every neighbor *u* 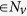

We used the PageRank energy function as recommended in^72^:


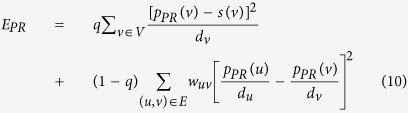


In equation (10) the parameter *q* ranges in [0, 1] and it weighs the contribution of the first and second sum. The first sum gives emphasis on differential gene expression values and the second one in the network topology. In this work we used the *q* value of 0.5 as recommended in^72^.

### Scoring the ranked gene lists

Each method is scored according to the maximum achieved mean classification accuracy across datasets, modified by two multiplicative weights: w_n_ (eq. 11) that is related to the number of genes required for the maximum accuracy and w_cv_ (eq. 12) that is related to the coefficient of variation (CV) of the classification accuracy along the first 100 genes (see Supplementary Tables 2–9).

Specifically,


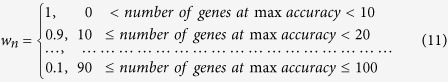



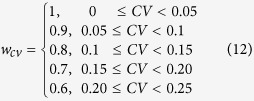


Finally, we calculated the average score of each method across stages and subtypes.

## Additional Information

**How to cite this article**: Bourdakou, M. M. *et al.* Discovering gene re-ranking efficiency and conserved gene-gene relationships derived from gene co-expression network analysis on breast cancer data. *Sci. Rep.* **6**, 20518; doi: 10.1038/srep20518 (2016).

## Supplementary Material

Supplementary Information

## Figures and Tables

**Figure 1:**
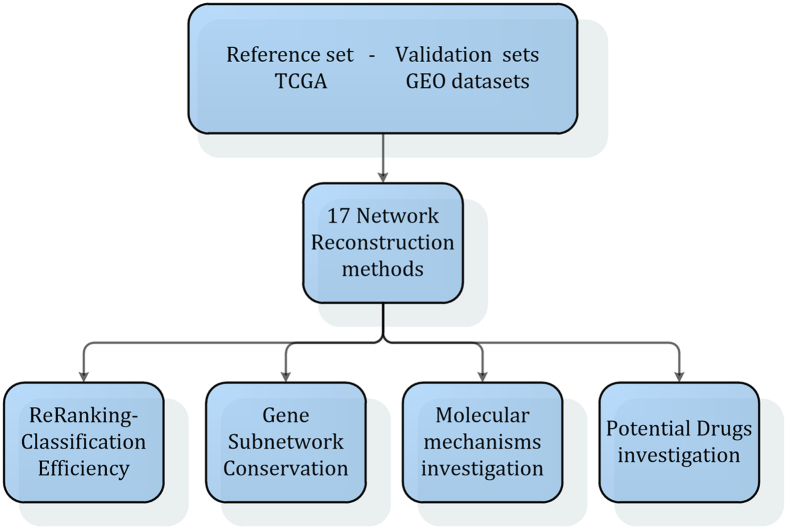
Analysis workflow was followed eight times for each of the four breast cancer subtypes and stages – initially TCGA mRNA Breast cancer gene expression datasets were statistically analyzed by means of LIMMA statistical R package in order to find the top 1000 differentially expressed genes, for each case. Derived gene lists were used as input for co-expression network reconstruction using 11 different network inference methods, one ensemble scheme and six biological. PageRank algorithm was applied to re-rank gene lists based on each network topology along with the existing expression profiles. For the re-ranked lists, we applied an SVM-based classification scheme using as training set the TCGA datasets, tested on a number of breast cancer GEO datasets available for each subtype and stage. Using the most efficient network inference method for each category, we derived to common subnetwork patterns across all datasets. In the sequel, we further investigated the nodes of each common subnetwork pattern regarding their capacity to reveal basic mechanisms and boost certain drug repurposing pipelines for each subtype and stage.

**Table 1:** Mean Score of each re-ranking method for the case of breast cancer stages.

Re-rankingMethods	Score @Stage I	Score @Stage II	Score @Stage III	Score @Stage IV	MEANScore
Initial	1.000	1.000	1.000	1.000	1.000
SN_I	1.000	0.900	0.900	1.000	0.950
Genenet	0.900	1.000	0.887	1.000	0.947
Lasso	0.900	0.980	0.986	0.900	0.942
AdLasso	0.900	1.000	0.900	0.900	0.925
WGCNA	0.900	0.802	0.986	1.000	0.922
SN	0.900	0.800	0.810	1.000	0.878
SN_A	0.810	0.900	0.900	0.900	0.878
mrnet	0.800	0.800	0.800	1.000	0.850
Bio5	0.630	0.700	0.986	1.000	0.829
CLR	0.810	0.720	0.473	1.000	0.751
Genie3	0.810	0.900	0.200	1.000	0.728
Voting	0.810	0.640	0.311	1.000	0.690
C3net	0.720	0.480	0.240	1.000	0.610
Aracnem	0.302	0.640	0.276	1.000	0.555
mrnetb	0.450	0.240	0.394	1.000	0.521
Aracnea	0.302	0.420	0.177	0.900	0.450
SN_PI	0.207	0.265	0.156	0.400	0.257

**Table 2:** Mean Score of each re-ranking method for the case of breast cancer subtypes.

Re-rankingMethods	Score @ TripleNegative	Score @Luminal A	Score @Luminal B	Score @HER2	MEANScore
MRNETB	0.645	0.722	0.784	0.756	0.727
Voting	0.802	0.712	0.762	0.580	0.714
WGCNA	0.633	0.717	0.685	0.756	0.698
MRNET	0.728	0.660	0.559	0.816	0.691
CLR	0.725	0.474	0.624	0.751	0.644
Genie3	0.708	0.639	0.685	0.401	0.608
AdLasso	0.440	0.760	0.470	0.682	0.588
Initial	0.666	0.388	0.533	0.651	0.560
C3net	0.674	0.572	0.355	0.575	0.544
Aracnea	0.625	0.077	0.641	0.764	0.527
SN_PI	0.587	0.623	0.271	0.612	0.523
Bio5	0.410	0.726	0.418	0.420	0.494
Aracnem	0.687	0.191	0.529	0.533	0.485
SN_I	0.429	0.428	0.504	0.314	0.419
Lasso	0.296	0.215	0.304	0.695	0.378
SN_A	0.292	0.070	0.228	0.734	0.331
SN	0.211	0.146	0.077	0.472	0.226
Genenet	0.280	0.070	0.071	0.140	0.140

**Table 3:** Common and exclusive significant pathways for the case of breast cancer stages.

Stage	Pathways	P-value	Genes
Stage I	*cell communication*	6.42E-07	LAMB3;KRT13;KRT8;LAMC2;KRT5;LMNB1;COL1A1;KRT18;COL5A1;KRT17;KRT15;COL5A2;SPP1
	*cytokine receptor interaction*	0.000903	CXCL11;CXCL9;IL6;CCL11;CCL7;IL20RA;LEPR;CXCL1;BMPR1B;CXCL13;CXCL3;CXCL2
	*metabolism of xenobiotics by cytochrome p450*	0.001439	ADH4;ADH1C;ADH1A;AKR1C1;AKR1C3;CYP3A5
	*3 chloroacrylic acid degradation*	0.002961	ADH4;ADH1C;ADH1A
	*ecm receptor interaction*	0.00403	COL1A1;COL5A1;LAMB3;COL5A2;SPP1;LAMC2
Stage II	*cell communication*	1.48E-07	COL17A1;LAMB3;COL11A1;LAMA3;KRT13;KRT8;LAMC2;KRT5;LMNB1;COL1A1;COMP;GJB2;KRT19;KRT18;IBSP;KRT17;KRT15;KRT37;COL5A2;KRT14;COL4A6;SPP1;DSG3;DSC1
	*3 chloroacrylic acid degradation*	0.000249	ADH4;ALDH1A3;ADH1C;ALDH2;ADH1B;ADH1A
	*cytokine receptor interaction*	0.001038	CXCL9;CCL11;TNFRSF18;IL20RA;CXCL1;CXCL13;CXCL3;CXCL2;PRLR;CX3CL1;EGFR;GHR;BMP2;CXCL11;IL6;TPO;CCL7;LEP;TNFSF4;KIT;IL21R;LEPR;CCL28;IL17B
	*ecm receptor interaction*	0.004466	COL1A1;IBSP;LAMB3;SV2B;COL11A1;COL5A2;LAMA3;COL4A6;SPP1;SDC1;LAMC2
	*tyrosine metabolism*	0.005824	ADH4;ALDH1A3;TPO;ADH1C;MAOB;ADH1B;MAOA;ADH1A
	*fatty acid metabolism*	0.008441	ADH4;ALDH1A3;ACADL;ADH1C;ALDH2;ADH1B;ADH1A
	*bile acid biosynthesis*	0.0136	ADH4;ALDH1A3;ADH1C;ALDH2;ADH1B;ADH1A
	*glycerolipid metabolism*	0.021223	ADH4;ALDH1A3;ADH1C;ALDH2;GPAM;ADH1B;ADH1A
	*1 and 2 methylnaphthalene degradation*	0.021512	ADH4;ADH1C;ADH1B;ADH1A
	*complement and coagulation cascades*	0.022486	C6;C7;F12;CFI;PLAUR;C4BPA;F3;CFB
	*phenylalanine metabolism	0.036024	ALDH1A3;TPO;MAOB;MAOA
Stage III	*cell communication*	2.58E-09	LAMB3;LAMA3;KRT13;KRT8;LAMC2;KRT5;LMNB1;COL1A1;COMP;KRT19;KRT18;IBSP;KRT17;KRT15;KRT37;COL5A2;KRT14;SPP1;DSG3
	*3 chloroacrylic acid degradation*	8.57E-05	ADH4;ALDH1A3;ADH1C;ADH1B;ADH1A
	*fatty acid metabolism*	0.001264	ADH4;ALDH1A3;ACADL;ADH1C;ADH1B;ADH1A
	*metabolism of xenobiotics by cytochrome p450*	0.002315	ADH4;ALDH1A3;ADH1C;ADH1B;ADH1A;AKR1C1;AKR1C3
	*1 and 2 methylnaphthalene degradation*	0.002336	ADH4;ADH1C;ADH1B;ADH1A
	*tyrosine metabolism*	0.002709	ADH4;ALDH1A3;ADH1C;MAOB;ADH1B;ADH1A
	*glycerolipid metabolism*	0.003212	ADH4;ALDH1A3;ADH1C;GPAM;ADH1B;ADH1A
	*bile acid biosynthesis*	0.003433	ADH4;ALDH1A3;ADH1C;ADH1B;ADH1A
	*ecm receptor interaction*	0.007067	COL1A1;IBSP;LAMB3;COL5A2;LAMA3;SPP1;LAMC2
	*cytokine cytokine receptor interaction*	0.008847	CCL11;IL20RA;CXCL1;CXCL13;CXCL3;CXCL2;CXCL11;IL6;CCL7;LEP;IL21R;LEPR;CCL28
	^*^glycolysis and gluconeogenesis	0.023202	ADH4;ALDH1A3;ADH1C;ADH1B;ADH1A
	^*^PPAR signaling pathway	0.028896	ACADL;MMP1;ADIPOQ;OLR1;ANGPTL4
	*complement and coagulation cascades*	0.032049	C6;C7;PLAUR;C4BPA;CFB
Stage IV	*cytokine receptor interaction*	0.002012	CXCL11;IL6;CCL11;CCL7;IL21R;LEPR;CXCL13;CXCL3;CXCL2
	*cell communication*	0.005242	COL1A1;KRT17;COL5A2;KRT14;SPP1;LMNB1
	*toll like receptor signaling pathway	0.029052	CXCL11;IL6;SPP1;FOS
	*ecm receptor interaction*	0.082661	COL1A1;COL5A2;SPP1
	*complement and coagulation cascades*	0.048285	PLAUR;C4BPA;F3

^*^Exclusive mechanisms for the specific Breast Cancer Stage.

**Table 4:** Common and exclusive significant pathways for the case of breast cancer subtypes.

Subtype	Term	P-value	Genes
Luminal A	*cell communication*	1.05E-05	GJB2;COL5A1;KRT17;KRT37;COL5A2;KRT14;LAMA3;COL4A6;FN1;KRT13;SPP1;KRT5
	*ecm receptor interaction*	0.001371	COL5A1;COL5A2;LAMA3;COL4A6;FN1;SPP1;CD36
	*adipocytokine signaling pathway*	0.002432	LEP;ADIPOQ;LEPR;CD36;SLC2A4;PCK1
	*ppar signaling pathway*	0.001848	FABP4;ADIPOQ;AQP7;LPL;CD36;PCK1
	*cell cycle*	0.003567	CCNA2;CCNB2;CCNB1;PTTG2;BUB1B;CDC25C;BUB1
Luminal B	*cell communication*	0.000115	COL5A1;KRT17;KRT37;COL5A2;KRT14;LAMA3;COL4A6;KRT13;SPP1
	*focal adhesion*	0.001244	PAK1;COL5A1;COL5A2;LAMA3;COL4A6;PAK7;SPP1;EGFR;MYLK
	*tyrosine metabolism*	0.006883	TPO;ADH1C;MAOB;ADH1A
	*ecm receptor interaction*	0.007472	COL5A1;COL5A2;LAMA3;COL4A6;SPP1
	* **glycine serine and threonine metabolism**	**0.024788**	**DMGDH;SDS;MAOB**
	*3 chloroacrylic acid degradation*	0.021358	ADH1C;ADH1A
HER2	*cell communication*	0.00018	COL17A1;LAMB3;COL11A1;FN1;KRT5;LMNB1;COL1A1;COMP;KRT19;IBSP;KRT17;KRT15;COL5A2;COL4A6;DSC1;INA
	*ppar signaling pathway*	0.000568	ACADL;ACSL1;MMP1;ADIPOQ;AQP7;OLR1;SLC27A6;CD36;SORBS1;PCK1
	*cell cycle*	0.001311	CCNA2;CDC20;CCNB2;CCNB1;CCNE2;CDKN2A;PTTG2;E2F1;CDC6;BUB1;CDC25A;MCM2
	* **glycerolipid metabolism**	**0.002357**	**ADH4;DGAT2;ADH1C;ALDH2;GPAM;ADH1A;PPAP2B;MGLL**
	*adipocytokine signaling pathway*	0.009519	ACSL1;ADIPOQ;LEPR;IRS2;CD36;SLC2A4;PCK1;ACACB
	*ecm receptor interaction*	0.009967	COL1A1;IBSP;LAMB3;COL11A1;COL5A2;COL4A6;FN1;ITGA7;CD36
	* **fatty acid metabolism**	**0.011914**	**ADH4;ACADL;ADH1C;ALDH2;ACSL1;ADH1A**
	*3 chloroacrylic acid degradation*	0.004958	ADH4;ADH1C;ALDH2;ADH1A
	*focal adhesion*	0.023258	FIGF;LAMB3;CAV1;COL11A1;FN1;MYLK;COL1A1;COMP;IBSP;PDGFD;COL5A2;COL4A6;ITGA7;PAK3
	*tyrosine metabolism*	0.02328	AOC3;ADH4;TPO;ADH1C;MAOB;ADH1A
	* **complement and coagulation cascades**	**0.024164**	**C7;F10;F12;PROS1;CFI;PLAUR;C4BPA**
	* **bladder cancer**	**0.026558**	**FIGF;CDKN2A;MMP1;E2F1;MMP9**
Triple Negative	*cell cycle*	1.14E-10	PLK1;BUB1B;CDC25C;PKMYT1;CCNA2;CDC20;CCNB2;CCNB1;CCNE2;PTTG1;CCNE1;PTTG2;CHEK1;BUB1;MAD2L1
	*cell communication*	1.65E-06	COL17A1;COL1A1;KRT17;LAMA2;COL11A1;COL5A2;KRT14;LAMA3;COL4A6;FN1;KRT5;LMNB1
	*ecm receptor interaction*	7.92E-05	COL1A1;LAMA2;COL11A1;COL5A2;LAMA3;COL4A6;FN1;HMMR
	*focal adhesion*	0.003057	COL1A1;LAMA2;CAV2;CAV1;COL11A1;COL5A2;LAMA3;COL4A6;FN1
	* **small cell lung cancer**	**0.002403**	**CCNE2;LAMA2;CCNE1;LAMA3;COL4A6;FN1**
	* **metabolism of xenobiotics by cytochrome p450**	**0.02563**	**ADH1C;ADH1A;AKR1C1;AKR1C3**
	*tyrosine metabolism*	0.053153461	TPO;ADH1C;ADH1A

*Exclusive mechanisms for the specific Breast Cancer Subtype.

**Table 5:** Drug List for all breast cancer stages – X represents the appearance of the drug in the specific stage.

LINCS Drugs	Stage I	Stage II	Stage III	Stage IV
1-benzhydryl-4-[(5-methyl-4-nitroisoxazol-3-yl)carbonyl]piperazine	X			
2-Chlor-N-(1-phenyl-3-propyl-1H-pyrazol-5-yl)acetamid	X			
4-{2-[(6-Chloro-4-quinazolinyl)amino]ethyl}phenol	X			
Ampicillin	X			X
clofarabine	X		X	
EMF-sumo1-12	X		X	X
etoposide	X			X
gemcitabine	X		X	X
HBEG	X			
idarubicin	X	X	X	X
INCA-6	X		X	
vanoxerine	X			
kinetin-riboside	X			
L755507	X			
N-[2-(allyloxy)benzyl]-N-1,3-benzodioxol-5-yl-2-chloroacetamide	X			
SA-792541	X			
SCH 79797 dihydrochloride	X			
Selamectin	X	X		X
Sepantronium	X			
teniposide	X			
3-(3-Benzoyl-6-chloro-4,5-dihydroxy-1-benzofuran-7-yl)-2,4-pentanedione		X		
AG-592		X		
Artesunate		X		
CD-437		X		
chrysenequinone		X		
cladribine		X		
cyclosporin-a		X		
IKK-2-inhibitor-V		X		
ingenol		X		
menadione		X		
N-[(5-Fluoro-8-hydroxy-7-quinolinyl)(2-thienyl)methyl]acetamide		X		
niclosamide		X		X
palbociclib		X		
Pevonedistat		X		
pyrvinium-pamoate		X	X	X
RO-28-1675		X		
triciribine		X		
wortmannin		X		
4-(4-Methoxyphenoxy)-2-(4-methylphenyl)-5-(2-thienyl)-3(2H)-pyridazinone			X	
6-(1,3-Benzodioxol-5-yl)-N-(cyclopentylmethyl)-4-quinazolinamine			X	
BIBR-1532			X	
ixazomib			X	
methyl-2,5-dihydroxycinnamate			X	X
mifepristone			X	
milrinone			X	
N’-[(E)-(2,3-Dihydroxyphenyl)methylene]-2-hydroxybenzohydrazide			X	
N-[5-(4-Morpholinylsulfonyl)-2-(1-pyrrolidinyl)phenyl]-4,5,6,7-tetrahydro-1-benzothiophene-2-carboxamide			X	
paroxetine			X	
ruxolitinib			X	
SA-1478088			X	
SCH-79797			X	
SKF-83959			X	
2-Dichloromethyl-4-ethylsulfanyl-6-phenyl-[1,3,5]triazine				X
4-{[5-(1-Naphthyl)-1,3,4-oxadiazol-2-yl]sulfanyl}-2-butyn-1-yl				X
BAS-02859604				X
calmidazolium				X
homoharringtonine				X
irinotecan				X
KM-03949SC				X
quizartinib				X
rhodomyrtoxin-b				X
TPCA-1				X
trichostatin-a				X

**Table 6:** Drug List for all breast cancer subtypes – X represents the appearance of the drug in the specific subtype.

LINCS Drugs	LuminalA	LuminalB	HER2	TripleNegative
4-[4-(4-Fluorobenzyl)-1-piperidinyl]thieno[2,3-d]pyrimidine	X			
5-[(Benzyloxy)methyl]-7-(1-piperidinylmethyl)-8-quinolinol	X	X		
6-(2,3-Dihydro-1,4-benzodioxin-6-yl)-N-(3-methylbenzyl)-4-quinazolinamine	X			
CD-437	X		X	X
chlorambucil	X	X		
cladribine	X			X
etoposide	X	X	X	X
gemcitabine	X		X	
heliomycin	X			
ingenol	X	X		
L-690488	X	X		
lonidamine	X			
methylene-blue	X	X		
N-[(5-Bromo-8-hydroxy-7-quinolinyl)(2-thienyl)methyl]acetamide	X			
N-[(5-Fluoro-8-hydroxy-7-quinolinyl)(2-thienyl)methyl]acetamide	X	X		
PAC-1	X			
palbociclib	X		X	
tunicamycin	X			
vorinostat	X		X	
wortmannin	X	X	X	X
4-(keto-methyl-oxido-sulfuraniumyl)-3-nitro-benzoic		X		
benzamide		X		
benzydamine		X		
diphenyleneiodonium		X		
menadione		X		
NM-PP1		X		
NVP-BEZ235		X		
obatoclax		X		
quinoclamine		X		
RO-28-1675		X		
serdemetan		X		
ZM-241385		X		
aminopurvalanol-a			X	
barasertib			X	
cytarabine			X	X
dasatinib			X	
entinostat			X	
fluticasone			X	
KIN001-055			X	
purvalanol-a			X	
pyrvinium-pamoate			X	X
SIB-1893			X	X
trichostatin-a			X	
triciribine			X	
tubastatin-a			X	X
WZ-4002			X	
(4E)-2-(4-Methoxyphenyl)-4-[(4-methoxyphenyl)imino]-4H-chromen-6-ol				X
5-[(Benzyloxy)methyl]-7-(1-pyrrolidinylmethyl)-8-quinolinol				X
chrysenequinone				X
clobetasol				X
cyclosporin-a				X
idarubicin				X
K784-3187				X
PLX-4720				X
teniposide				X
WAY-170523				X
withaferin-a				X
WYE-125132				X

**Table 7:** TCGA Breast Cancer sub-datasets with normal and tumor samples.

	Categories	Number ofNormal TCGASamples	Number ofTumor TCGASamples
Stages	Triple Negative	61	55
	Luminal A	61	218
	Luminal B	61	69
	HER2	61	23
Subtypes	Stage I	61	89
	Stage II	61	296
	Stage III	61	111
	Stage IV	61	13

**Table 8:** GEO Breast Cancer sub-datasets with normal and tumor samples.

**Stages**	**GSE ID**	**Stage I**	**Stage II**	**Stage III**	**Stage IV**	**Normal**
	GSE53752	12	25	11	3	25
	GSE61304	5	33	18	1*	4
**Subtypes**	**GSE ID**	**Triple Negative**	**Luminal A**	**Luminal B**	**HER2**	**Normal**
	GSE65194	4	30	30	30	11
	GSE57297	3	19	3	0	7
	GSE36295	11	12	7	6	5
	GSE53752	51	0	0	0	25
	GSE38959	30	0	0	0	13
	GSE50428	6	5	5	5	5

^*^This dataset was excluded for Stage IV due to insufficient number of samples (GSE comprised of 1 sample with Stage IV).

**Table 9:** Network reconstruction methods.

Name	Category	Package
Aracne.a^31^	Mutual Information	PARMIGENE
Aracne.m^32^	Mutual Information	PARMIGENE
CLR^33^	Mutual Information	PARMIGENE
MRNET^34^	Mutual Information	PARMIGENE
MRNETB^34^	Mutual Information	MINET
C3NET^35^	Mutual Information	C3NET
Lasso^36^	Correlation	PARCOR
Adaptive Lasso^37^	Correlation	PARCOR
Genenet^38^	Correlation	ENA
WGCNA^39^	Correlation	ENA
Genie3^42^	Tree –Based	Genie3
Bio5^46^,^47^	Biological Information	Cytoscape-GeneMANIA
Signaling Network^71^	Biological Information	X
Signaling Network_Activation^71^	Biological Information	X
Signaling Network_Inhibition^71^	Biological Information	X
Signaling Network_Physical Interactions^71^	Biological Information	X
Voting	Mutual Information, Correlation, Tree –Based	X
