# Supplementary material for: Discovering gene re-ranking efficiency and conserved gene-gene relationships derived from gene co-expression network analysis on breast cancer data
Source: Sci Rep. 2016 Feb 19;6:20518. doi: 10.1038/srep20518 (PMC4759568; doi:10.1038/srep20518)
Supplement: Supplementary Information [file srep20518-s1.doc]

**Supplementary Information**

**Discovering gene re-ranking efficiency and conserved gene-gene relationships derived from gene co-expression network analysis on breast cancer data**

Marilena M. Bourdakou^1, 2^, Emmanouil I. Athanasiadis^1^ and George M. Spyrou^1*^

^1^ Center of Systems Biology, Biomedical Research Foundation, Academy of Athens, Soranou Ephessiou 4, 115 27 Athens, Greece.

^2^ Department of Informatics and Telecommunications, University of Athens, 15784 Ilissia Athens, Greece.

^*^ Corresponding author. George M. Spyrou, Tel.: +30 210 6597151; Fax: +30 210 6597505; E-mail: gspyrou@bioacademy.gr


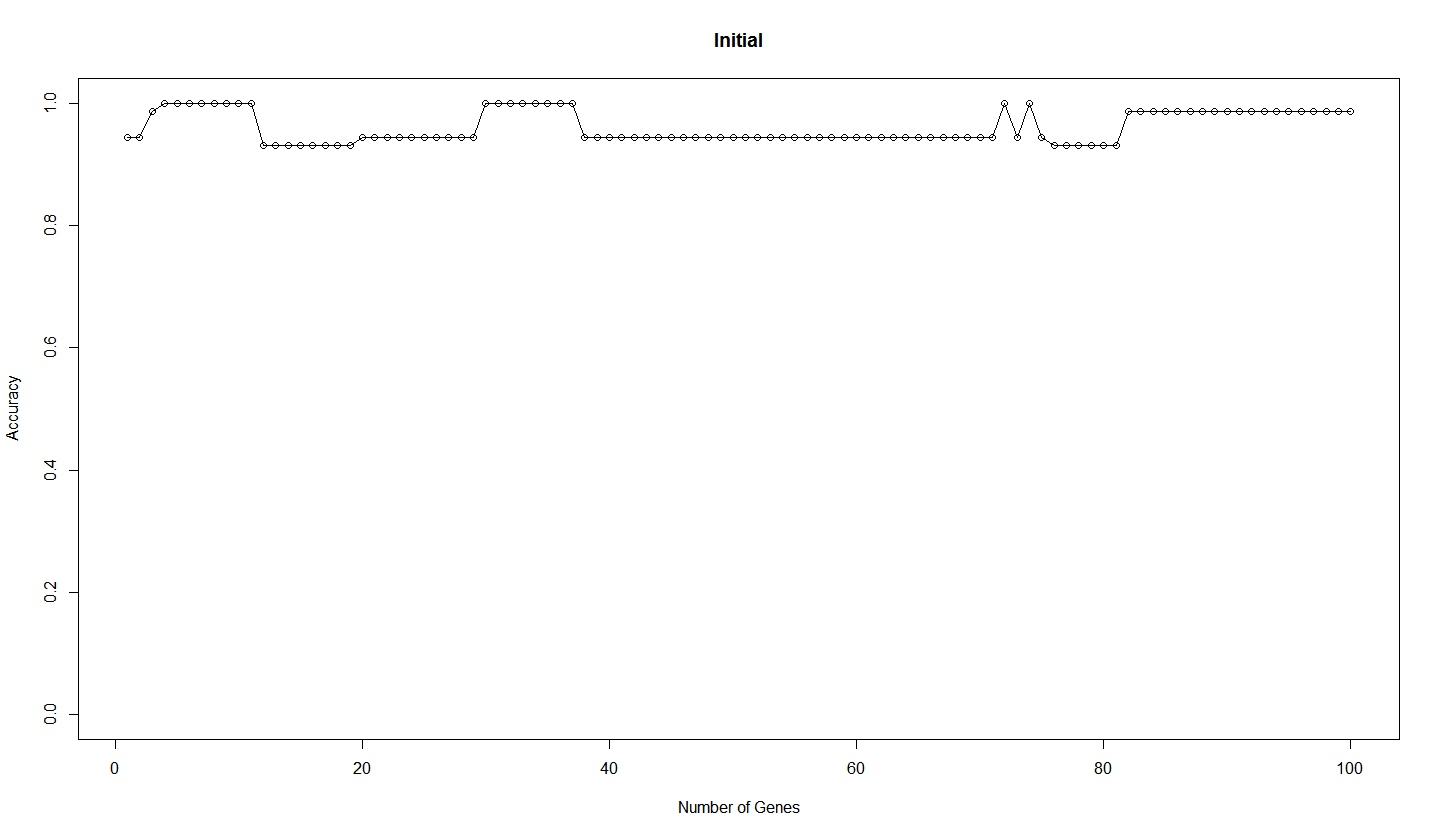


**Supplementary Figure 1:** Mean accuracy rate of the sequential gene selection from the top 100 Initial ranked genes (from Limma), using hold out validation with train set the TCGA expression values and test set the expression values from 2 GEO independent datasets for breast cancer Stage I.


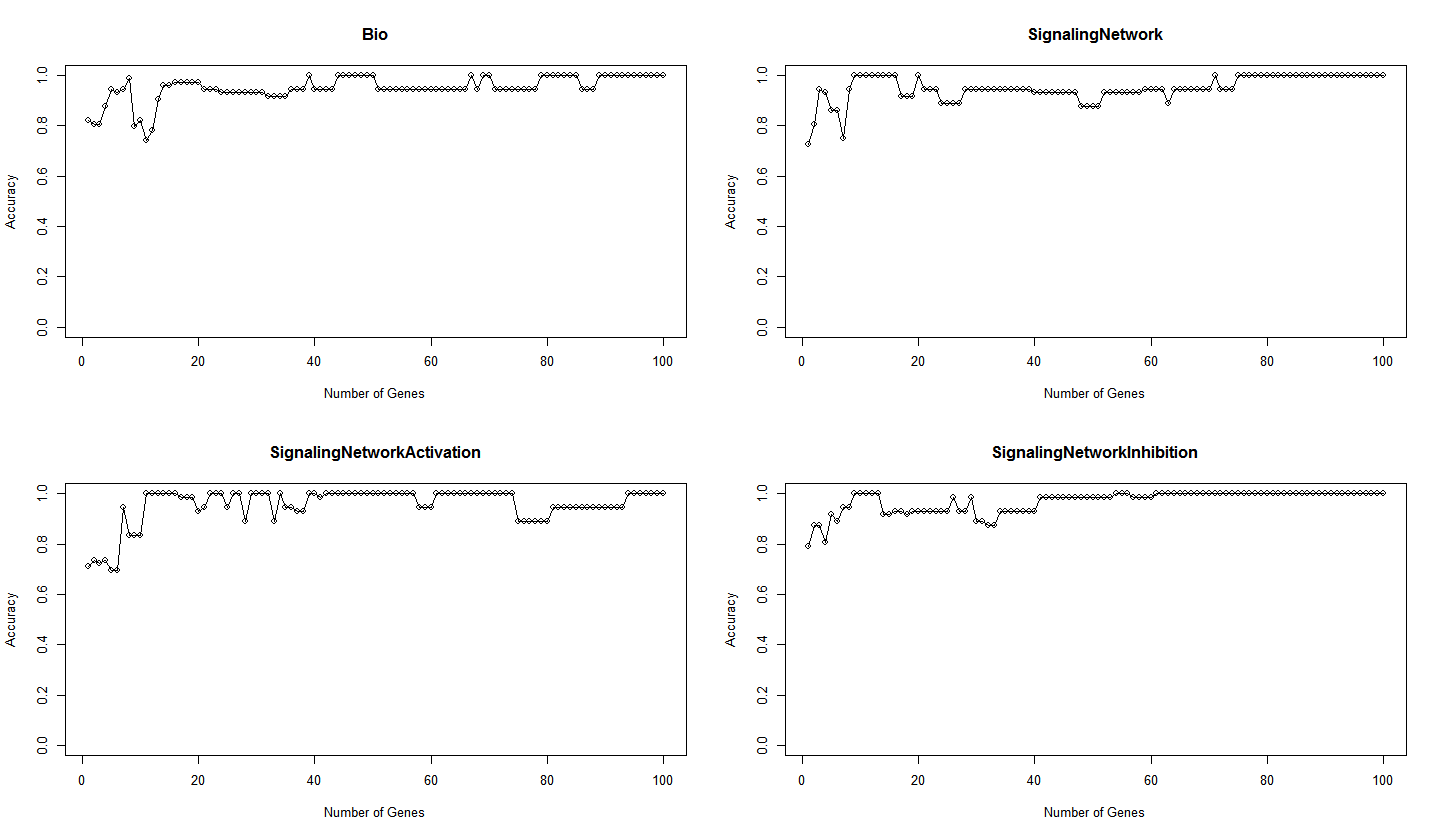


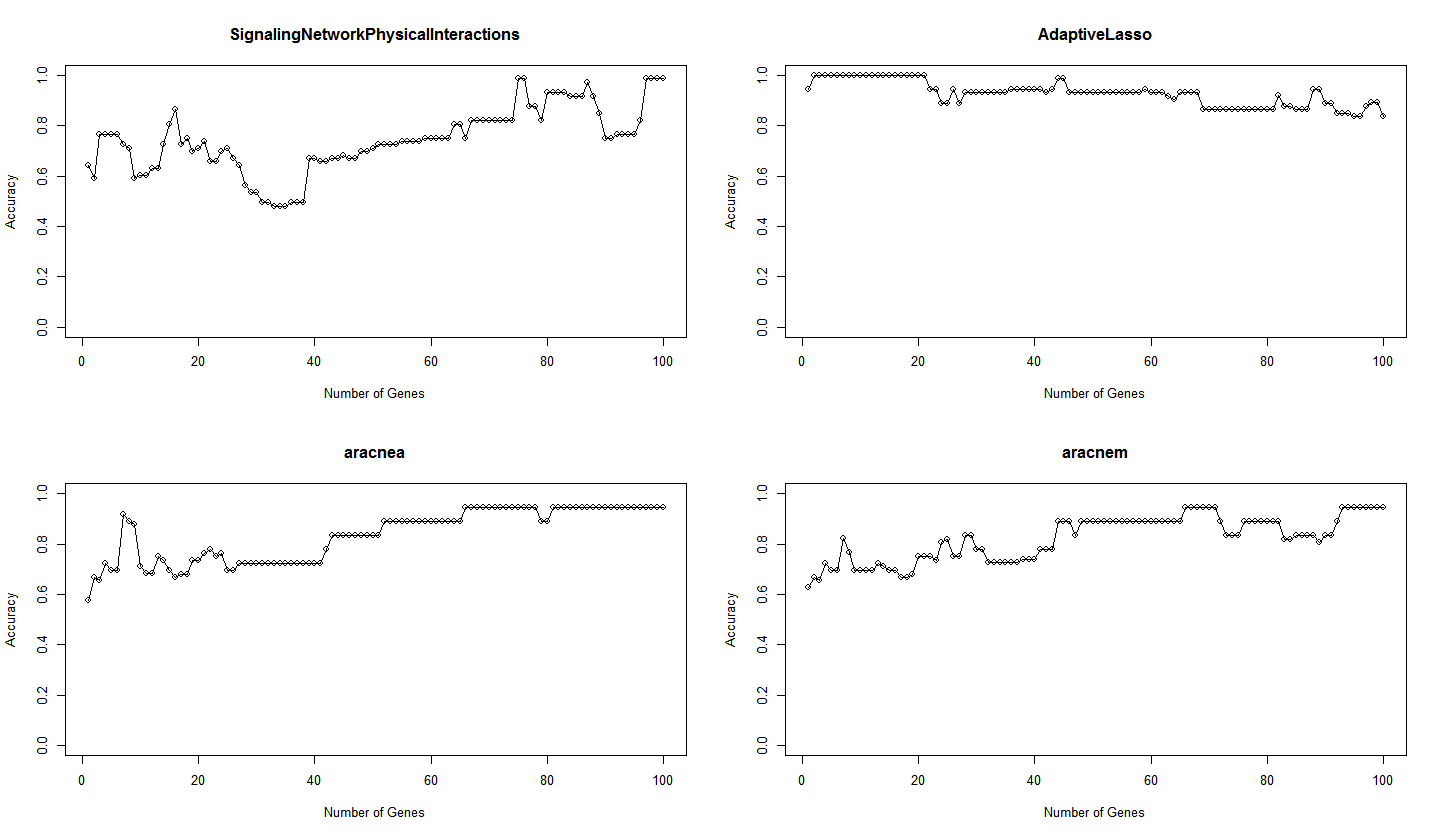


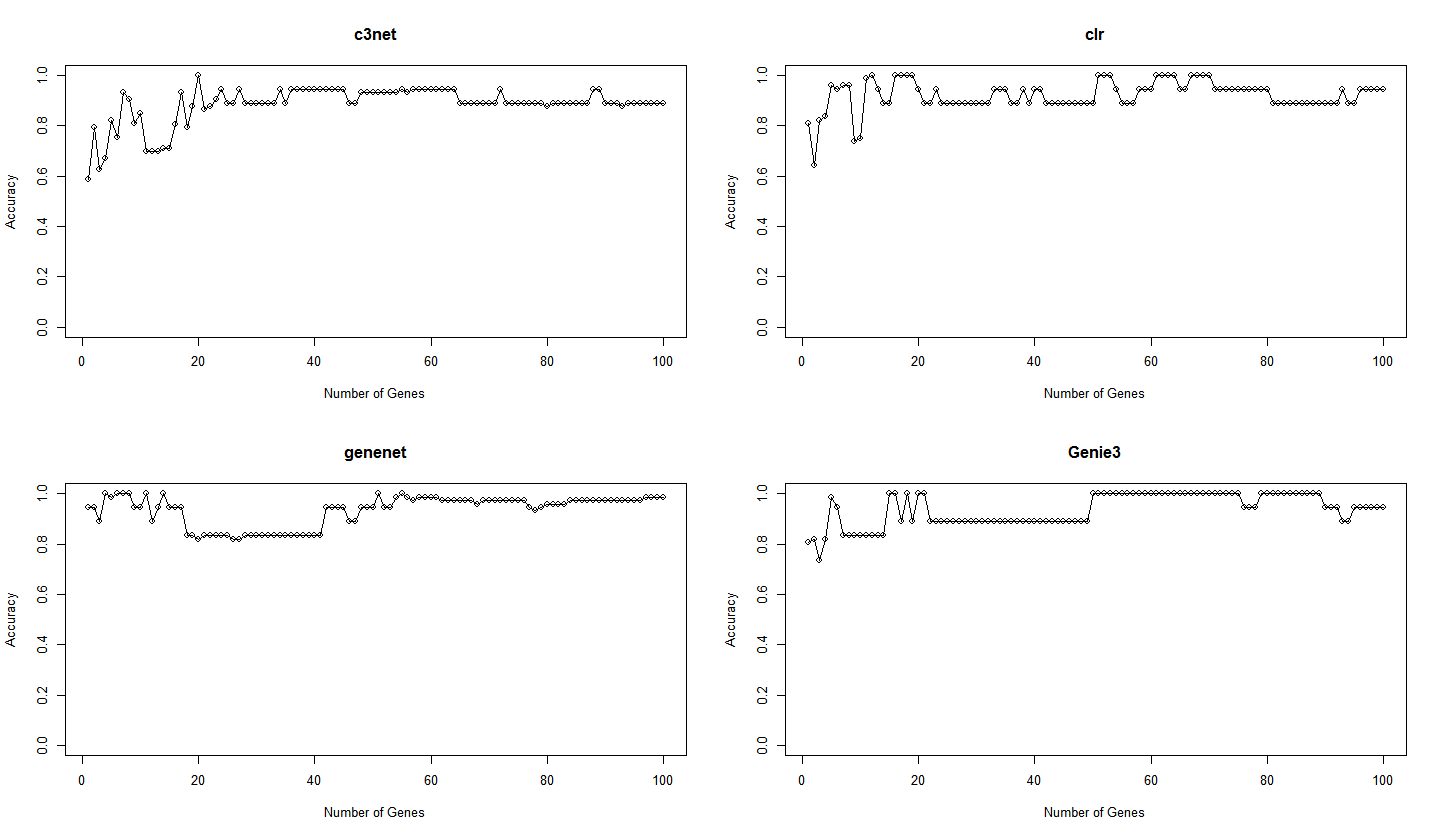


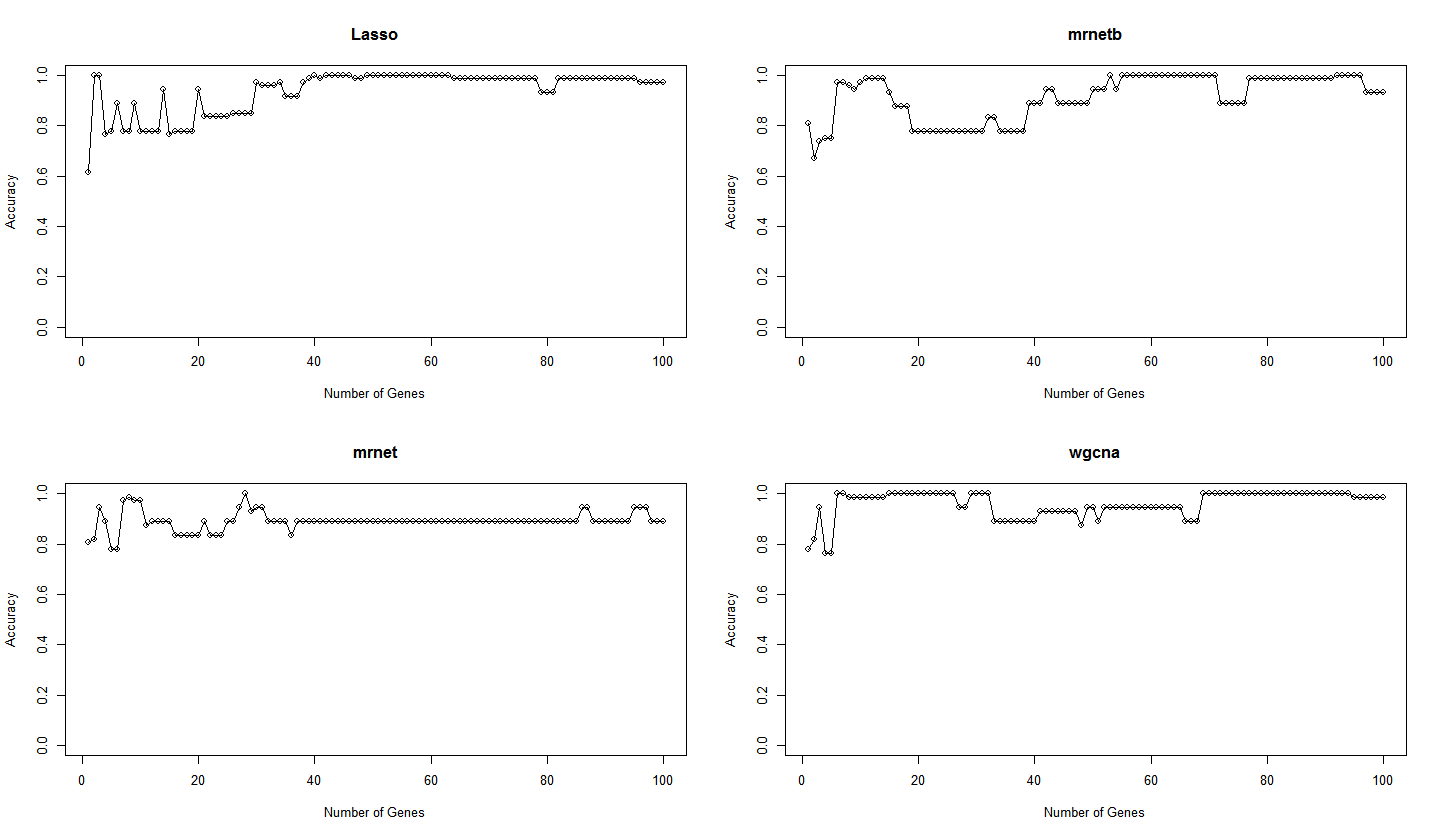


**Supplementary Figure 2:** Mean accuracy rates of the top 100 sequential genes from all ranked and re-ranked gene lists from each method in combination with PageRank reconciling method, using hold out validation with train set the TCGA expression values and test set the expression values from 2 GEO independent datasets for breast cancer Stage I.


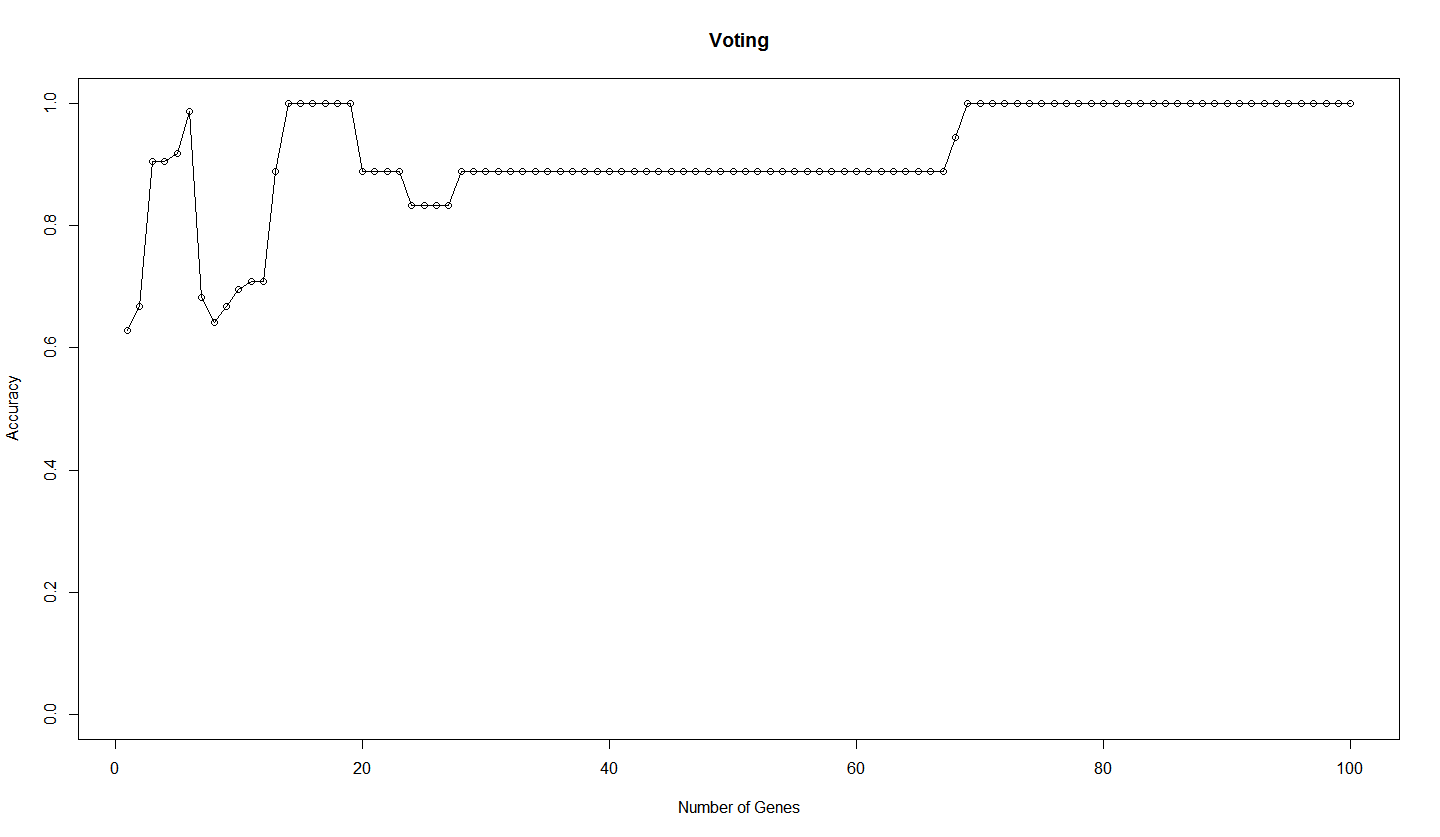


**Supplementary Figure 3:** Mean accuracy rate of the sequential gene selection from the top 100 re-ranked genes with the ensemble Voting method, using hold out validation with train set the TCGA expression values and test set the expression values from 2 GEO independent datasets for breast cancer Stage I.


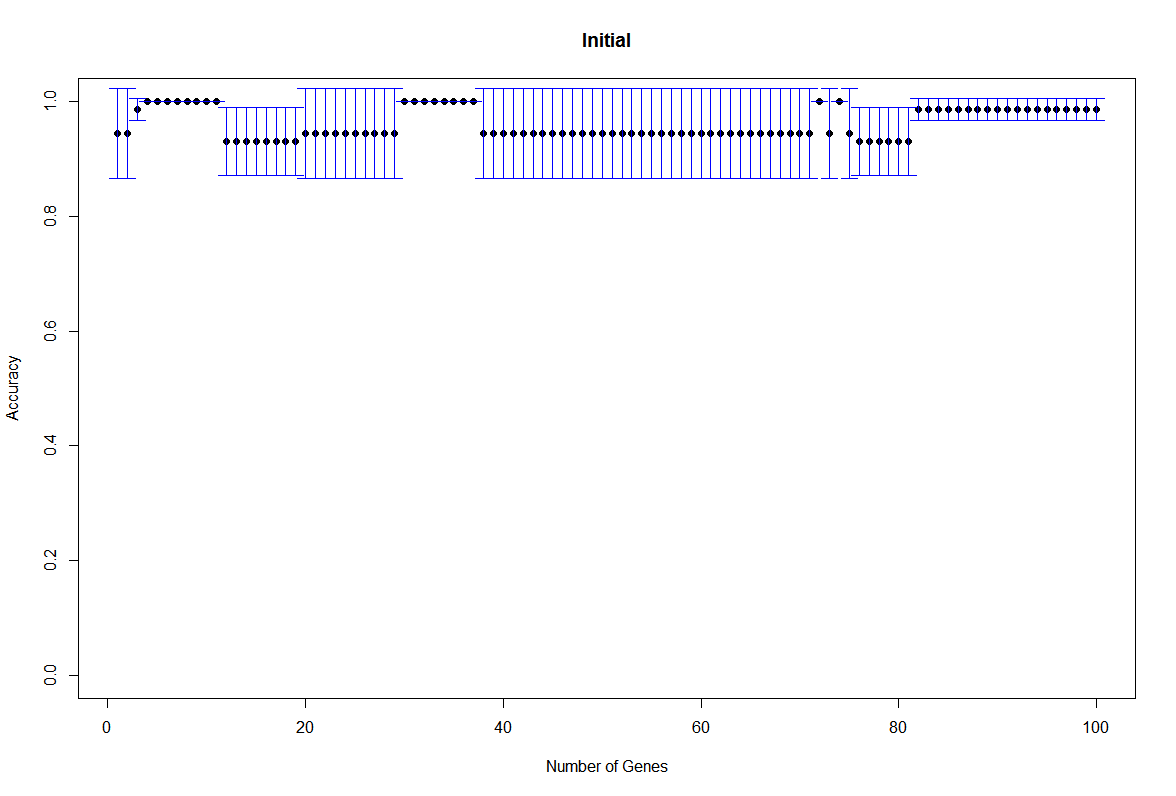


**Supplementary Figure 4:** Mean accuracy rate, with error bars, of the sequential gene selection from the top 100 Initial ranked genes (from Limma), using hold out validation with train set the TCGA expression values and test set the expression values from 2 GEO independent datasets for breast cancer Stage I.


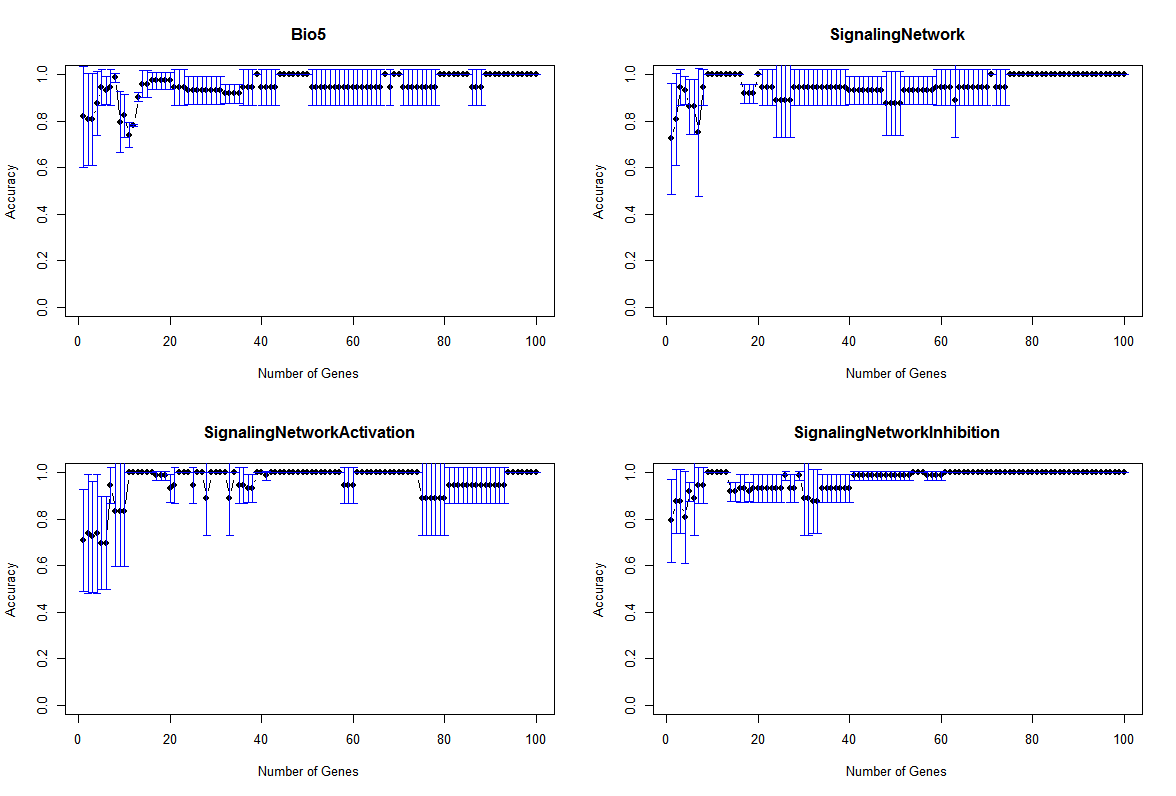


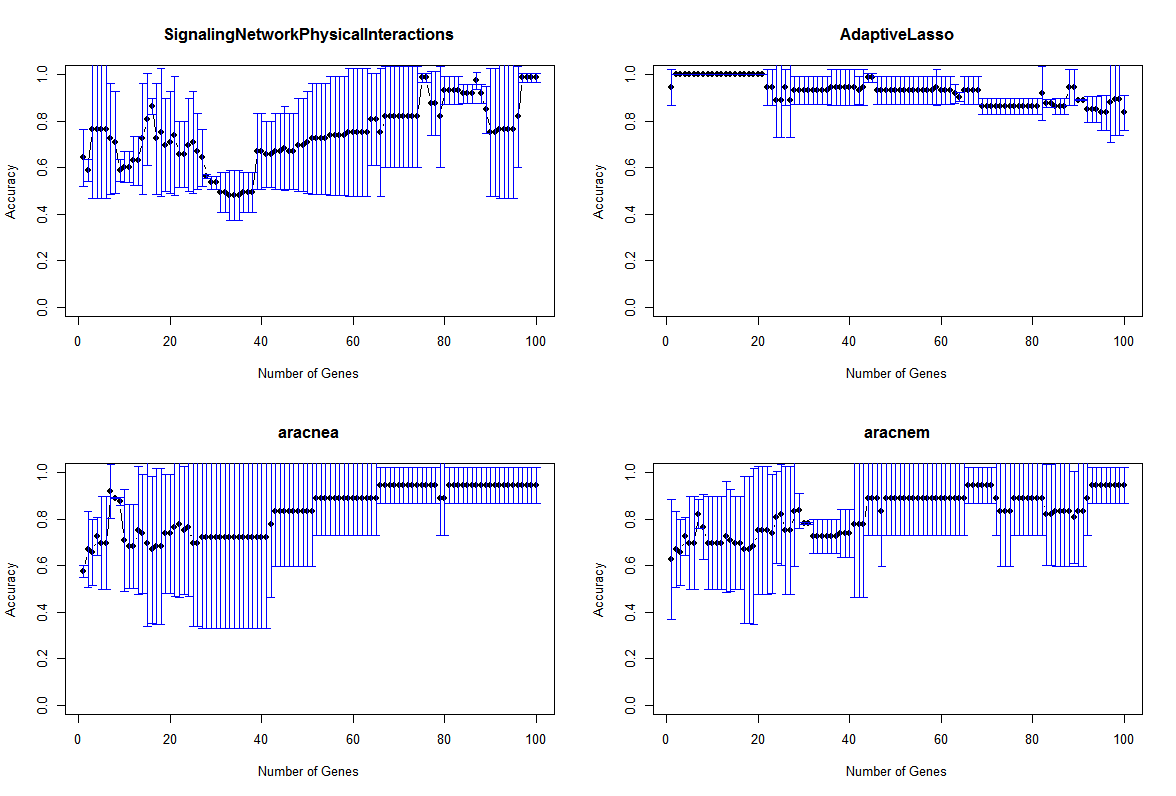


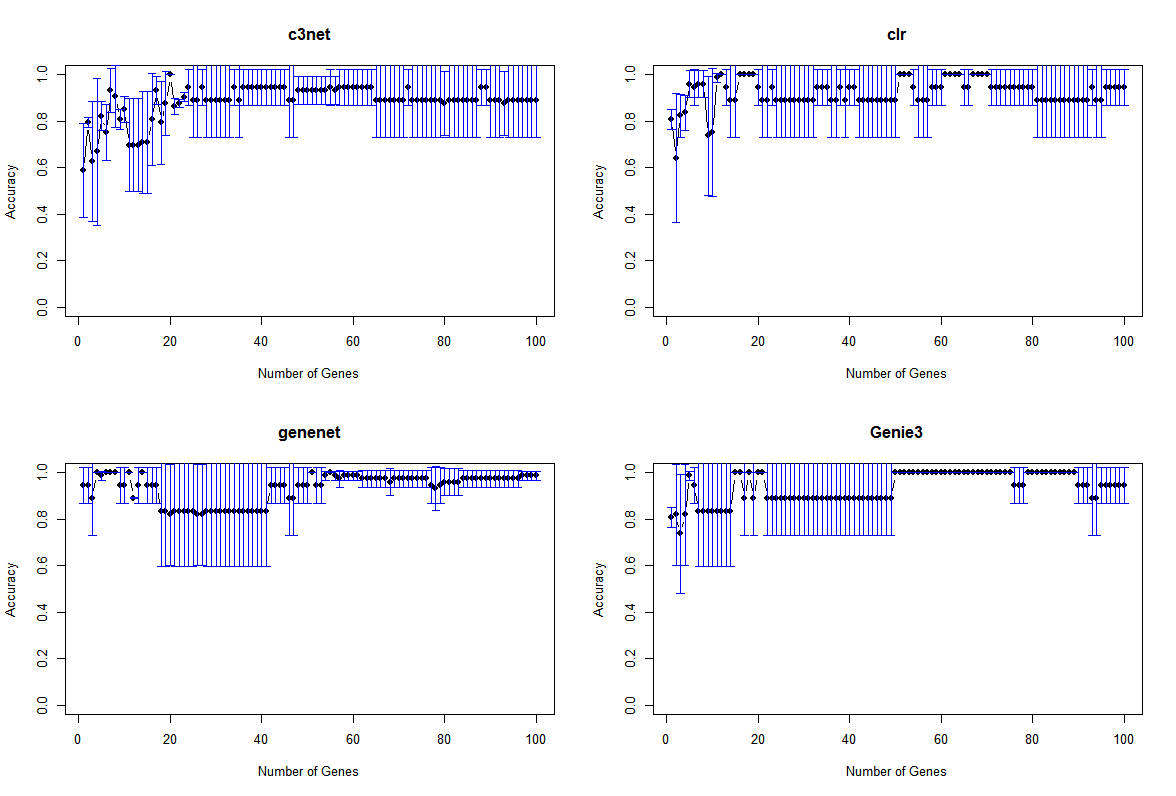


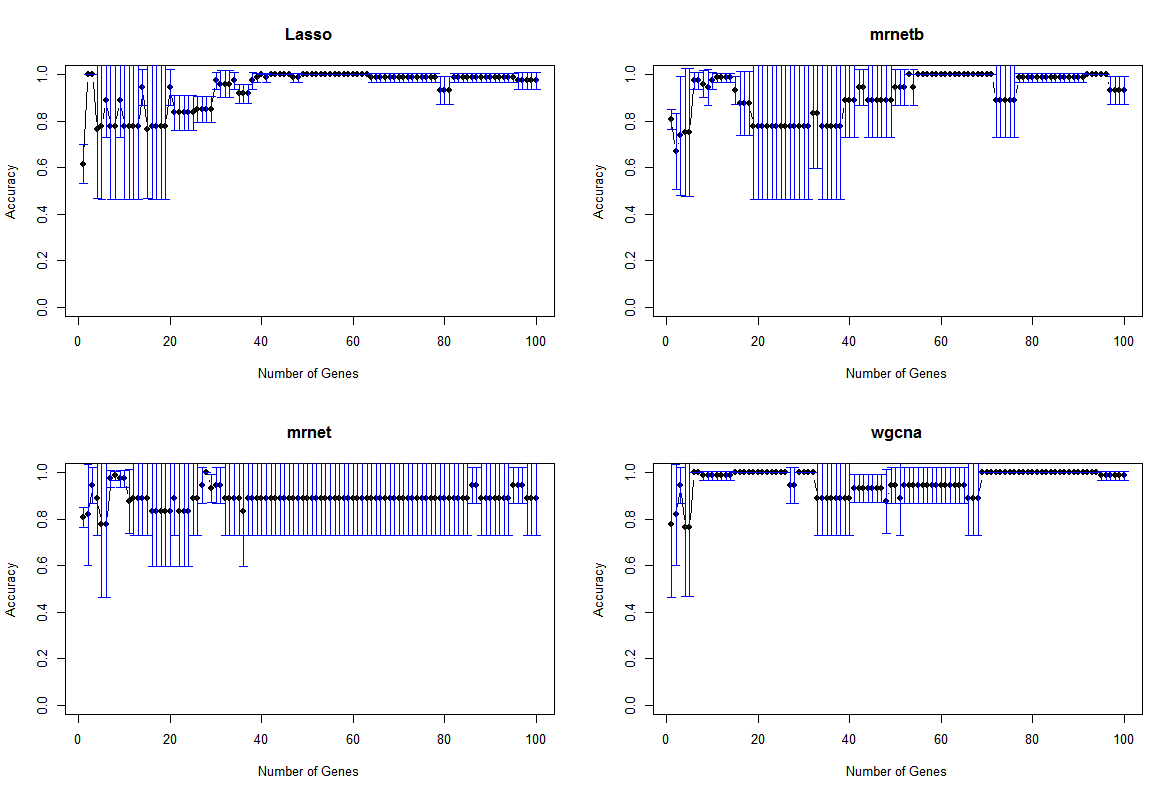


**Supplementary Figure 5:** Mean accuracy rates, with error bars, of the top 100 sequential genes from all ranked and re-ranked gene lists from each method in combination with PageRank reconciling method, using hold out validation with train set the TCGA expression values and test set the expression values from 2 GEO independent datasets breast cancer Stage I.


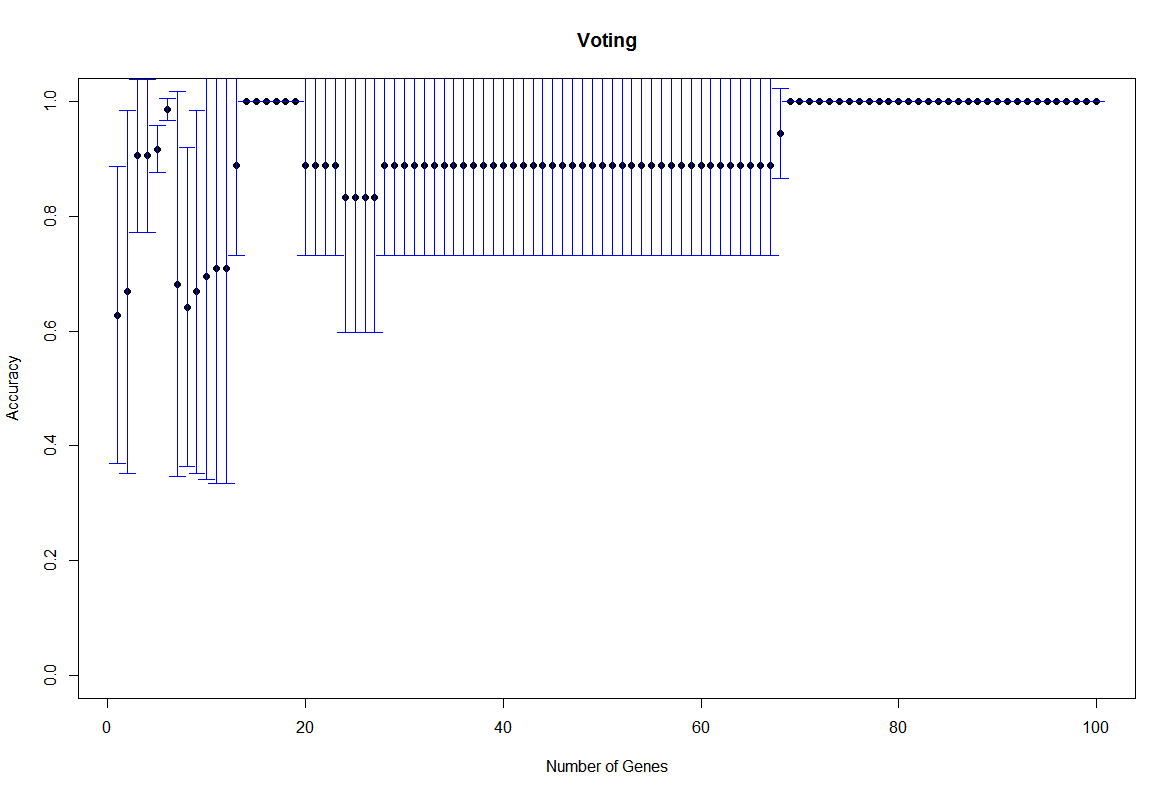


**Supplementary Figure 6:** Mean accuracy rate, with error bars, of the sequential gene selection the top 100 re-ranked genes with the ensemble Voting method, using hold out validation with train set the TCGA expression values and test set the expression values from 2 GEO independent datasets for breast cancer Stage I.


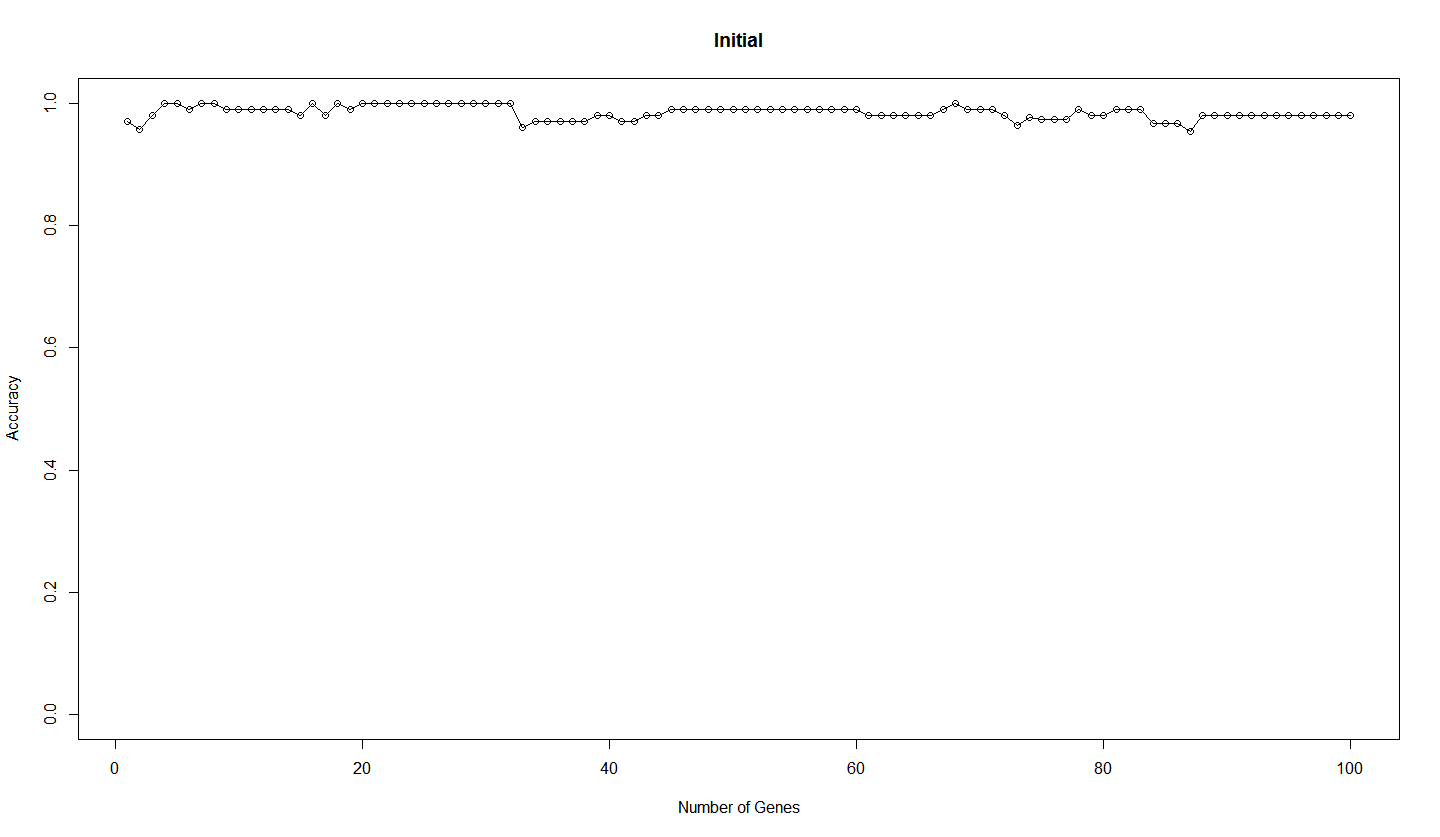


**Supplementary Figure 7:** Mean accuracy rate of the sequential gene selection from the top 100 Initial ranked genes (from Limma), using hold out validation with train set the TCGA expression values and test set the expression values from 2 GEO independent datasets for breast cancer Stage II.


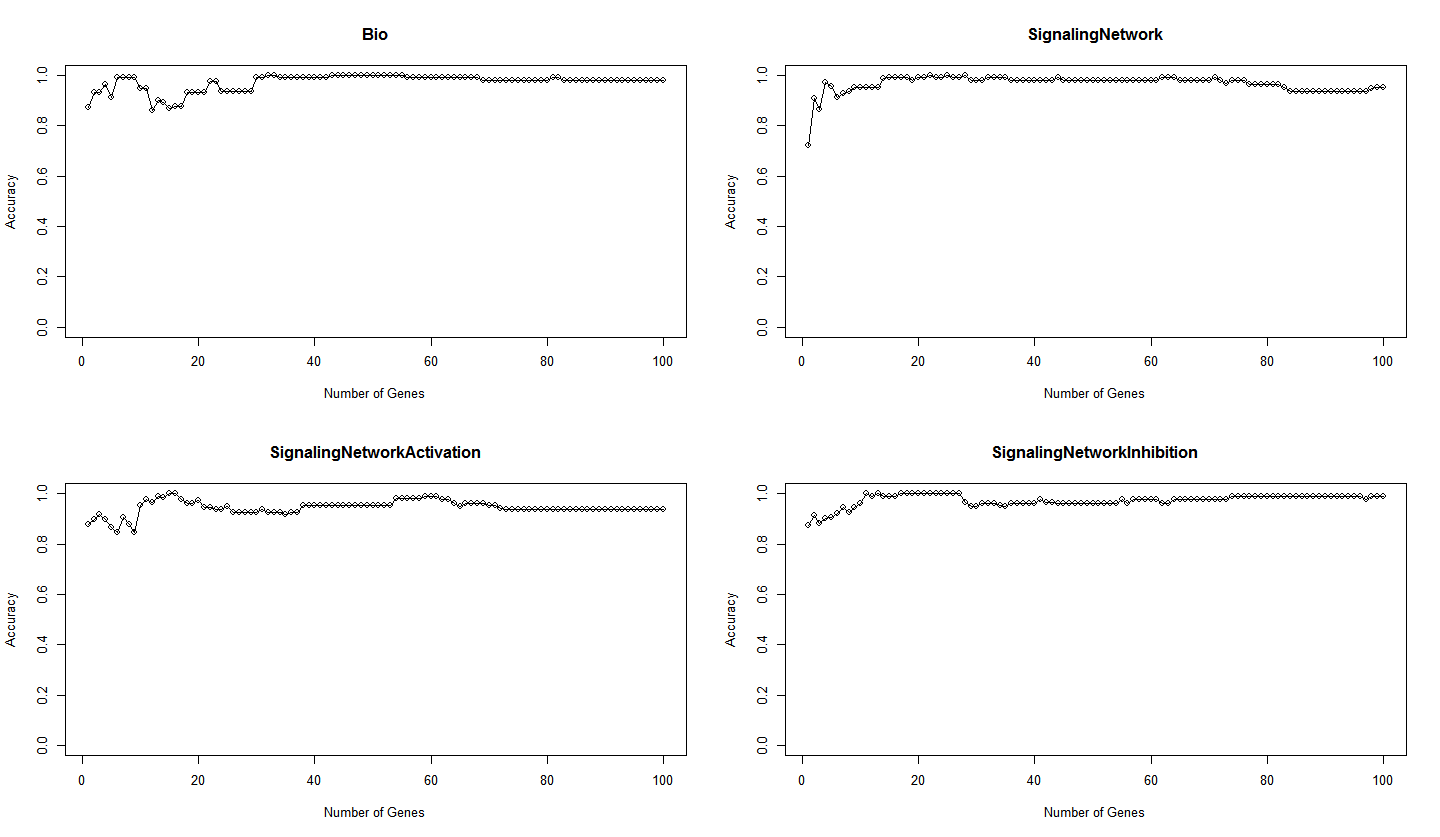


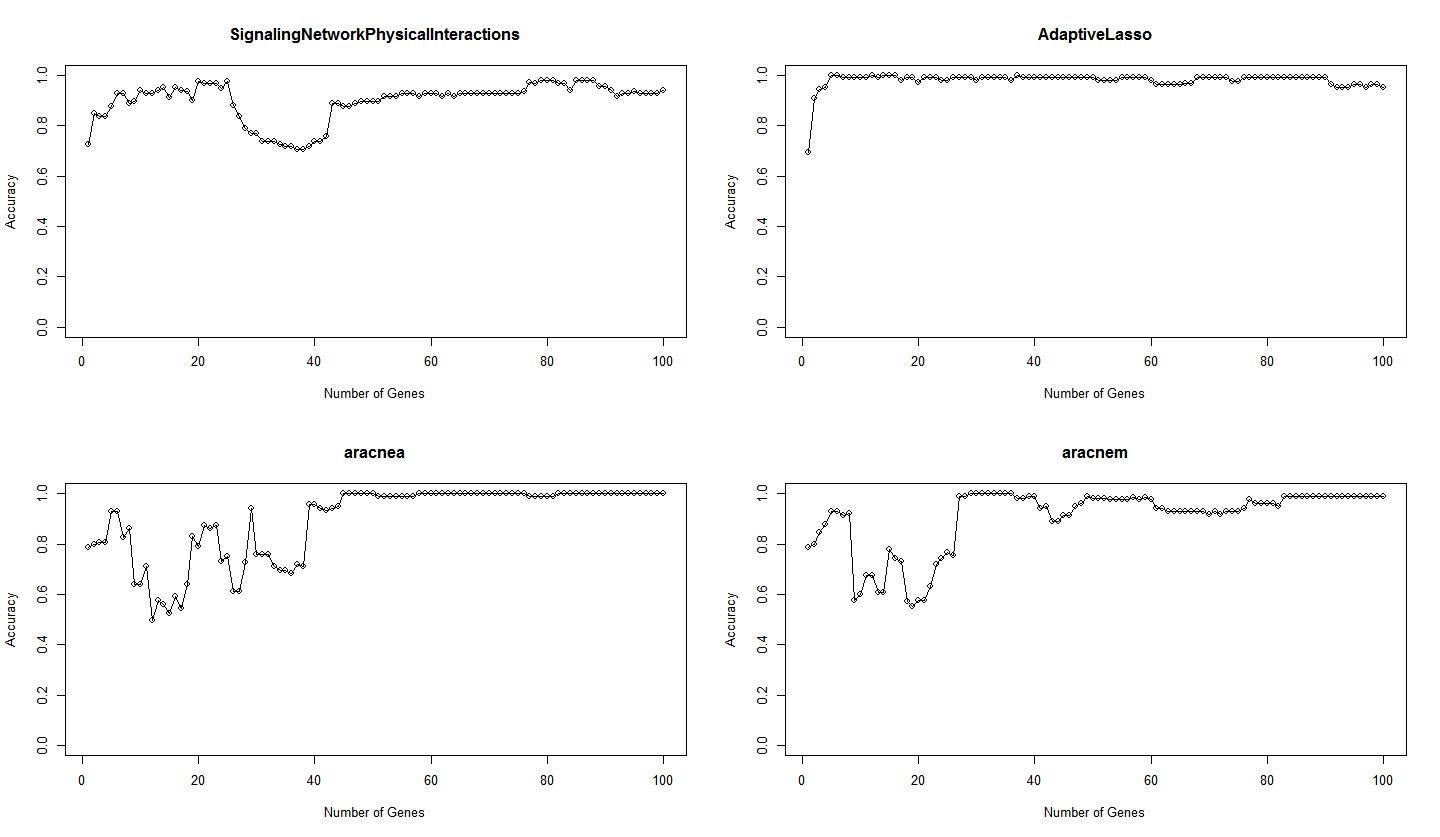


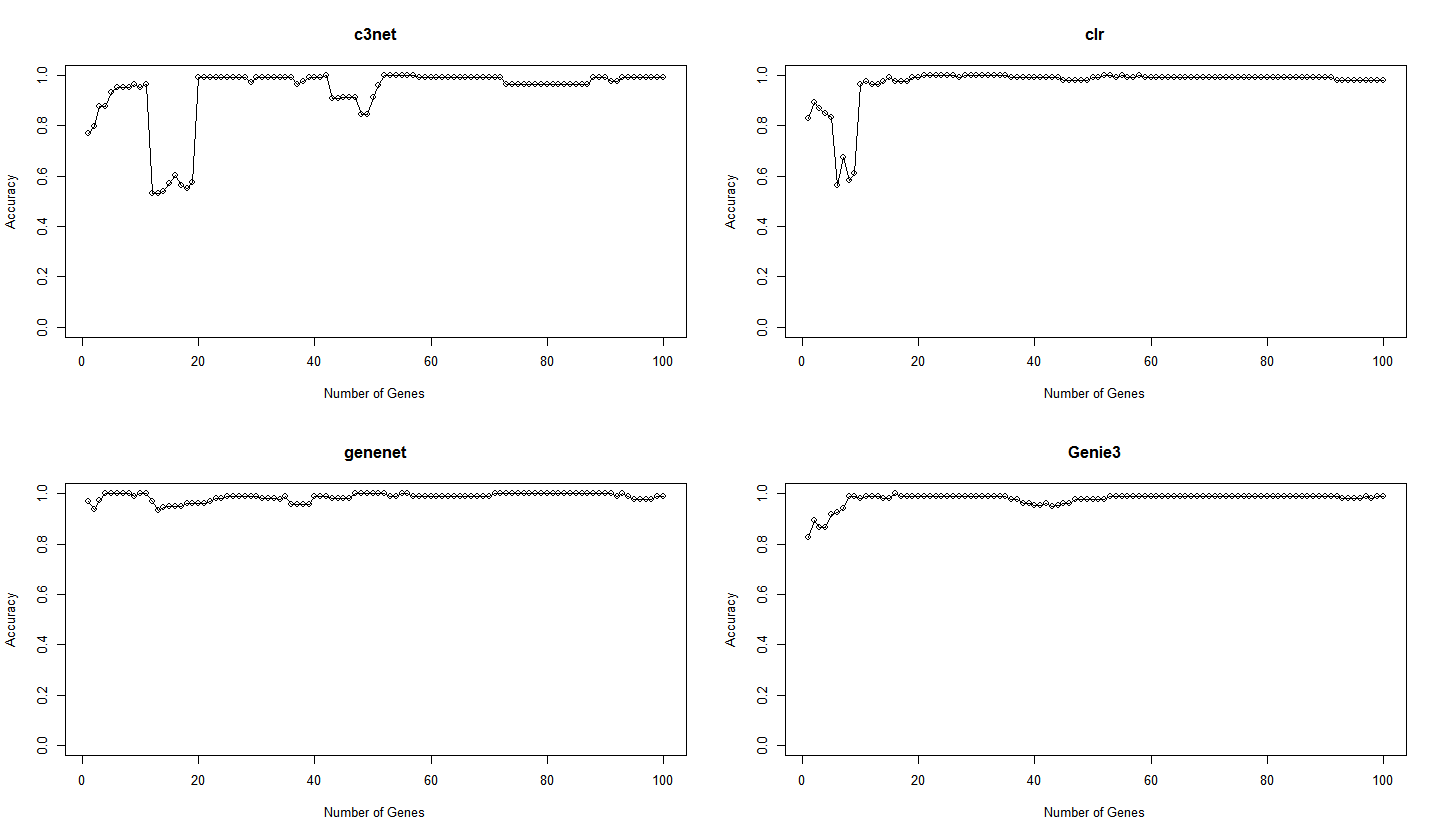


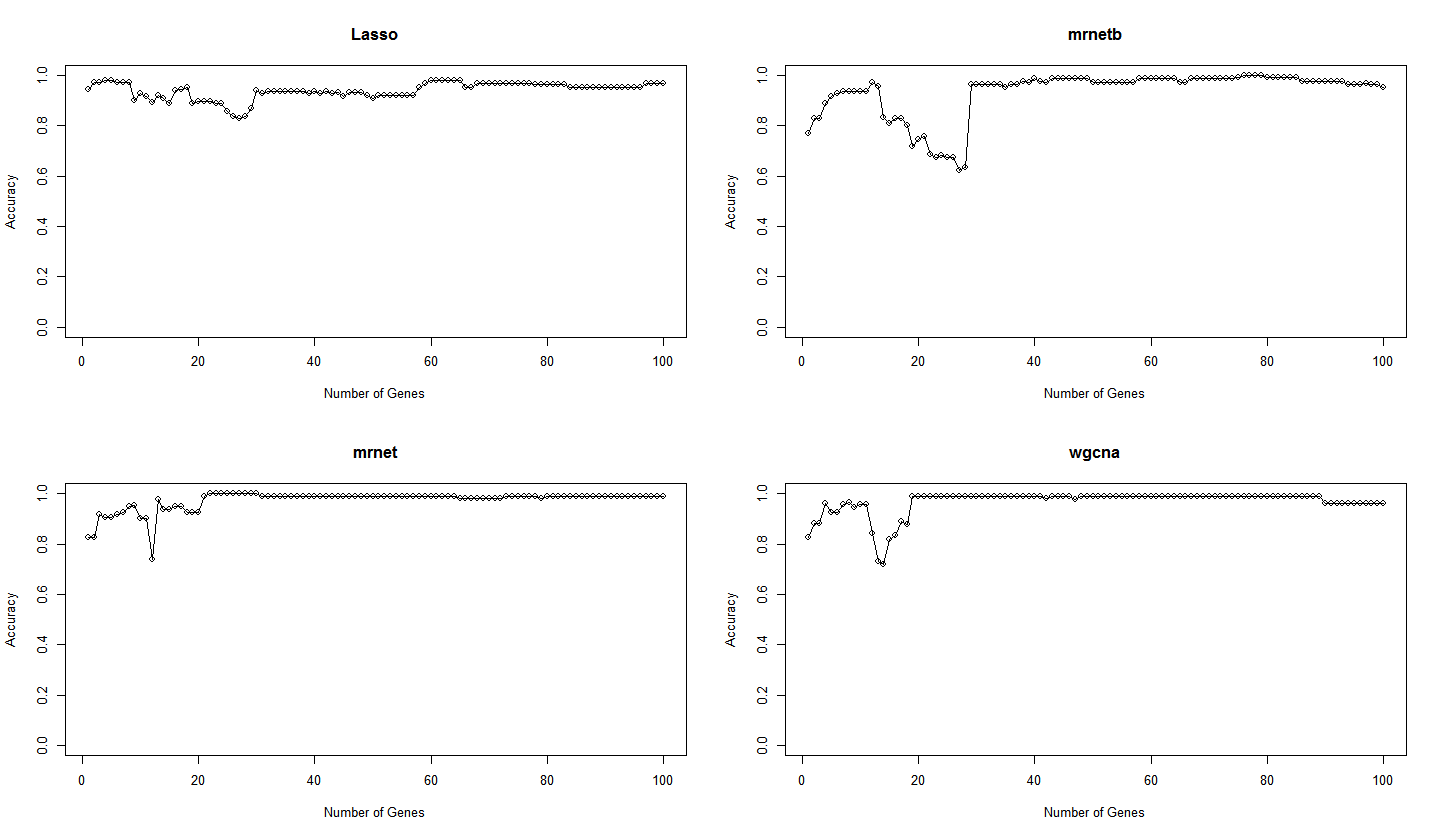


**Supplementary Figure 8:** Mean accuracy rates of the top 100 sequential genes from all ranked and re-ranked gene lists from each method in combination with PageRank reconciling method, using hold out validation with train set the TCGA expression values and test set the expression values from 2 GEO independent datasets for breast cancer Stage II.


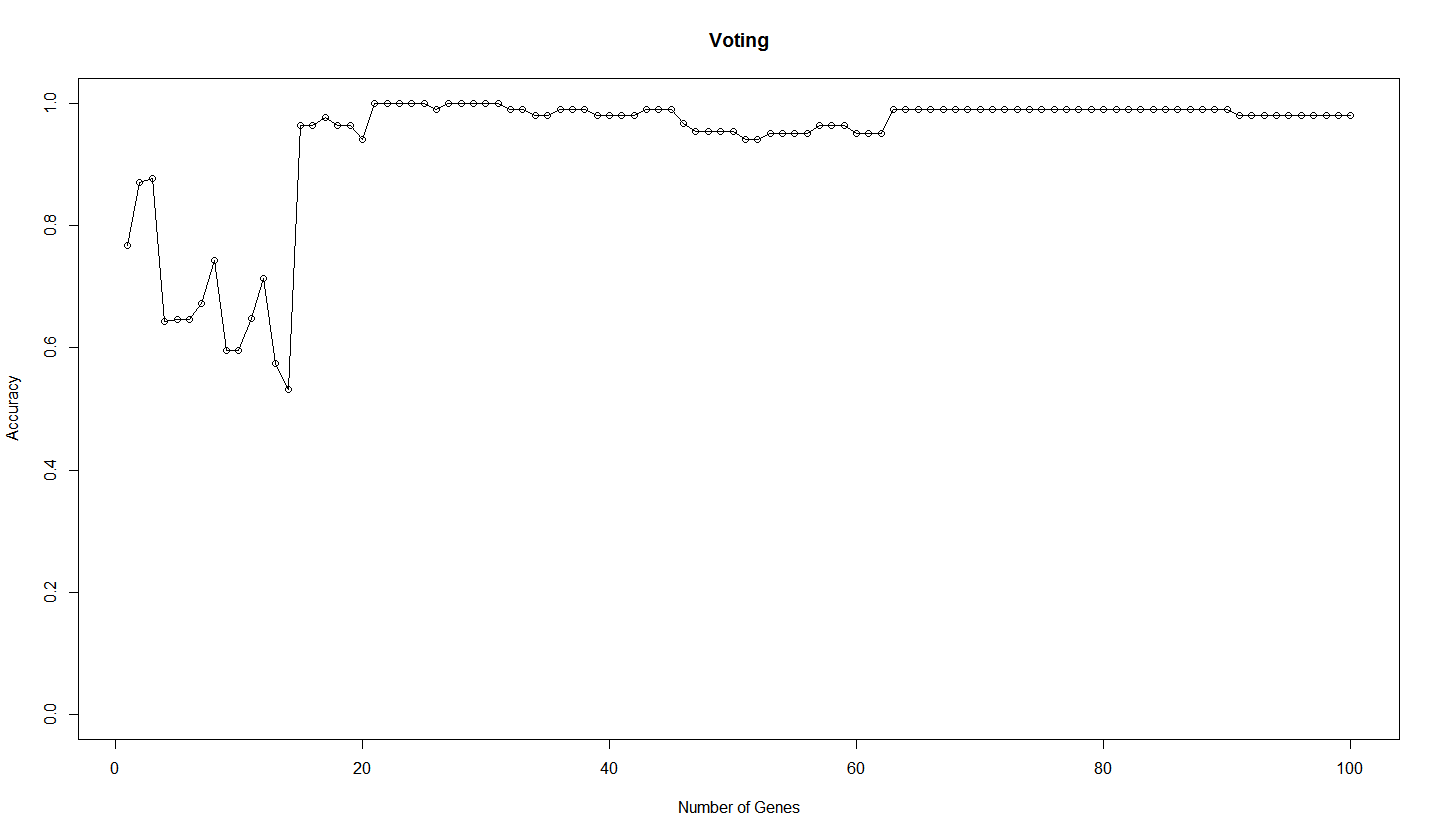


**Supplementary Figure 9:** Mean accuracy rate of the sequential gene selection from the top 100 re-ranked genes with the ensemble Voting method, using hold out validation with train set the TCGA expression values and test set the expression values from 2 GEO independent datasets for breast cancer Stage II.


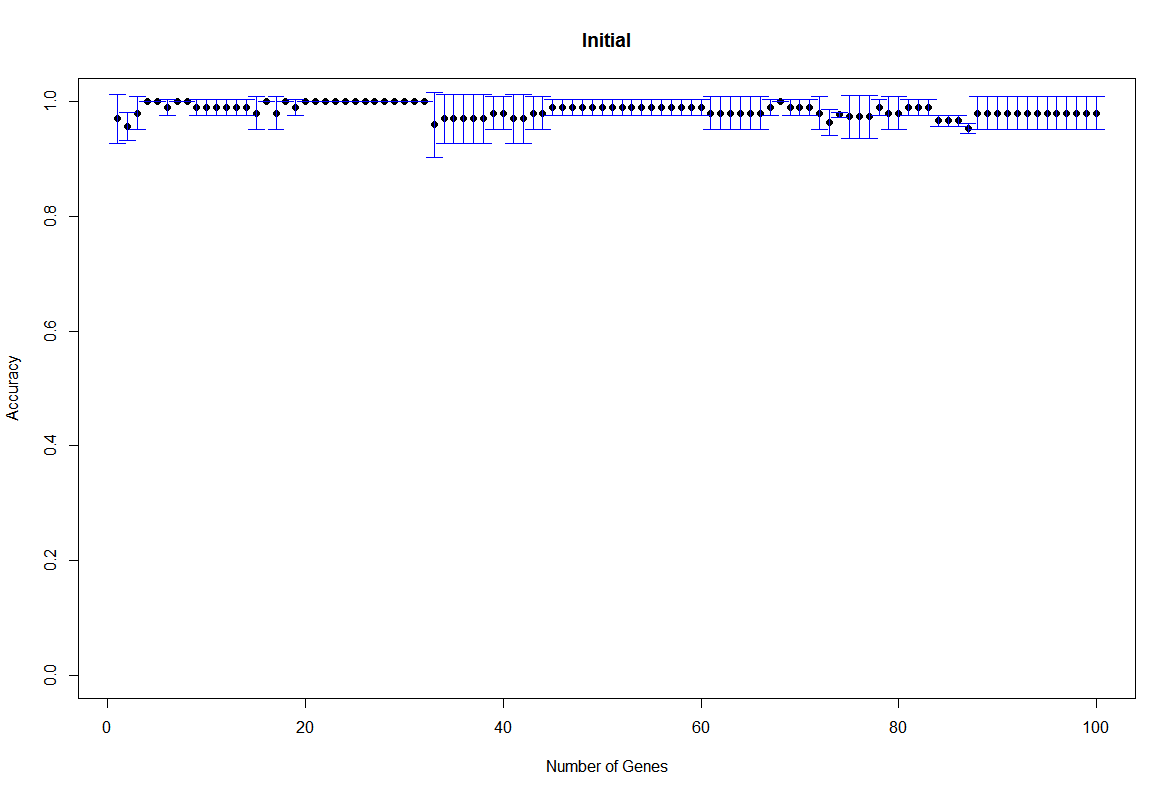


**Supplementary Figure 10:** Mean accuracy rate, with error bars, of the sequential gene selection from the top 100 Initial ranked genes (from Limma), using hold out validation with train set the TCGA expression values and test set the expression values from 2 GEO independent datasets for breast cancer Stage II.


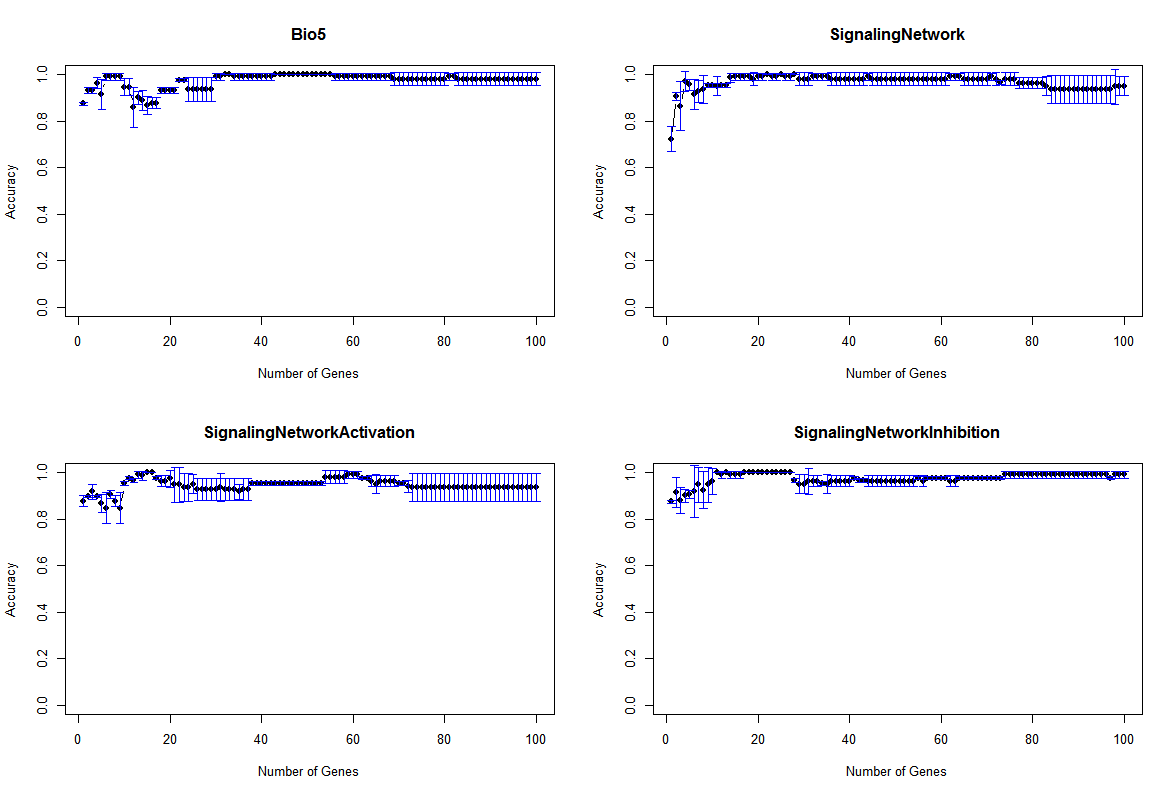


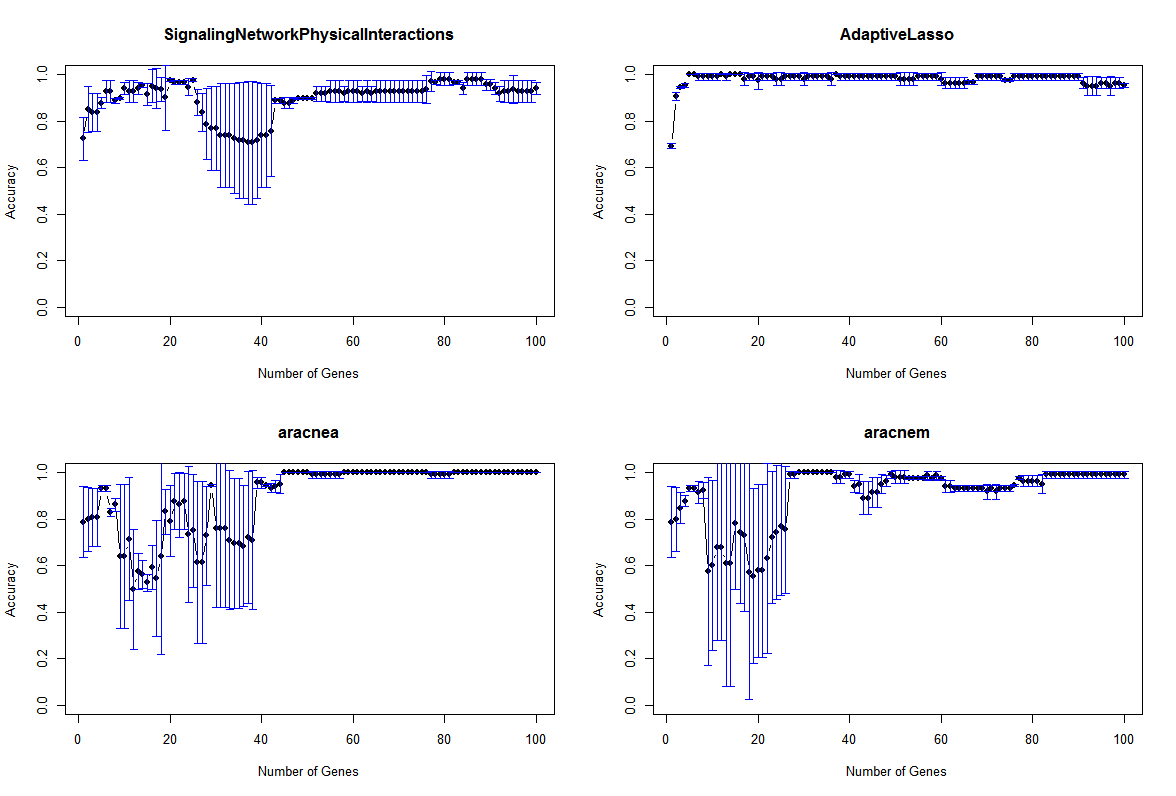


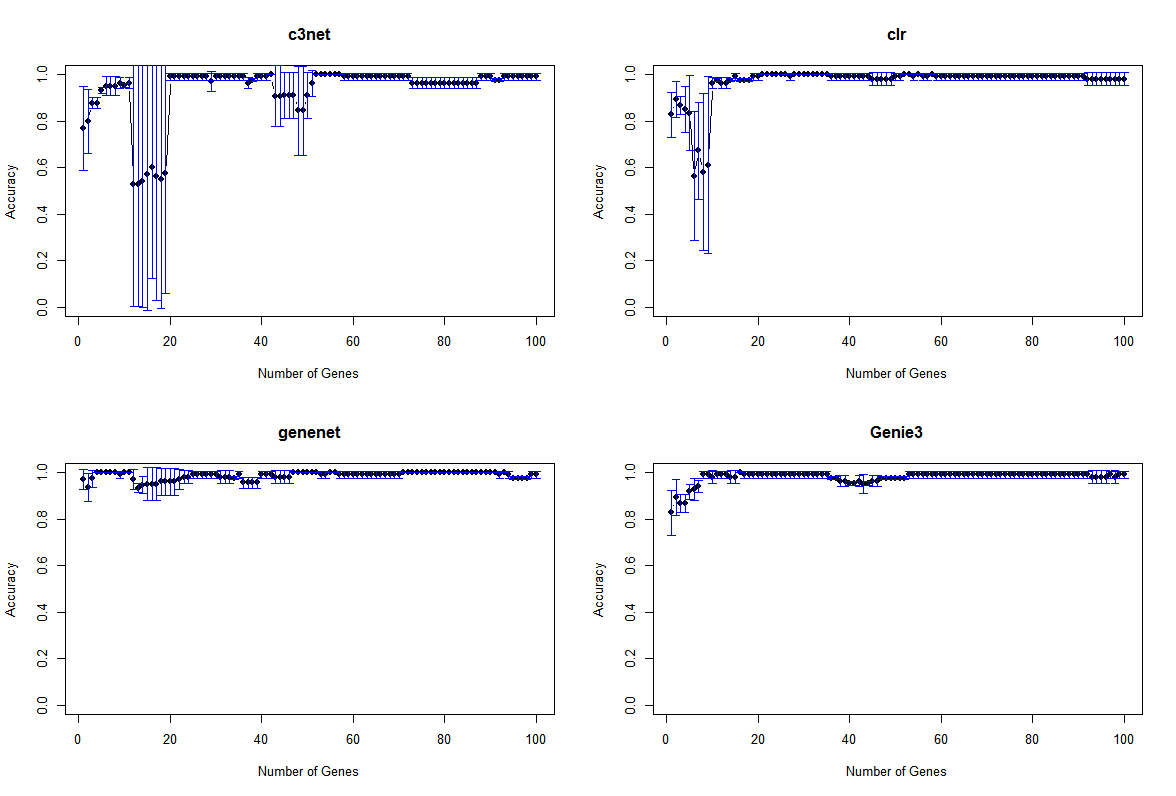


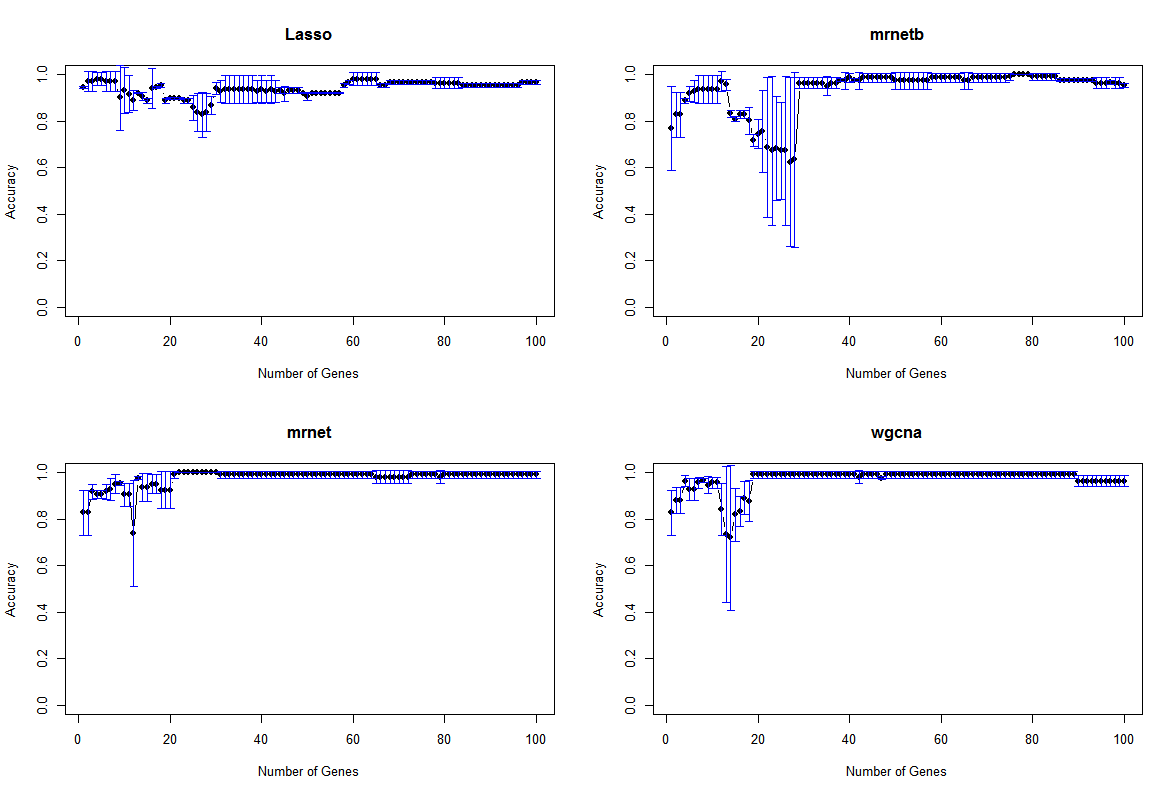


**Supplementary Figure 11:** Mean accuracy rates, with error bars, of the top 100 sequential genes from all ranked and re-ranked gene lists from each method in combination with PageRank reconciling method, using hold out validation with train set the TCGA expression values and test set the expression values from 2 GEO independent datasets breast cancer Stage II.


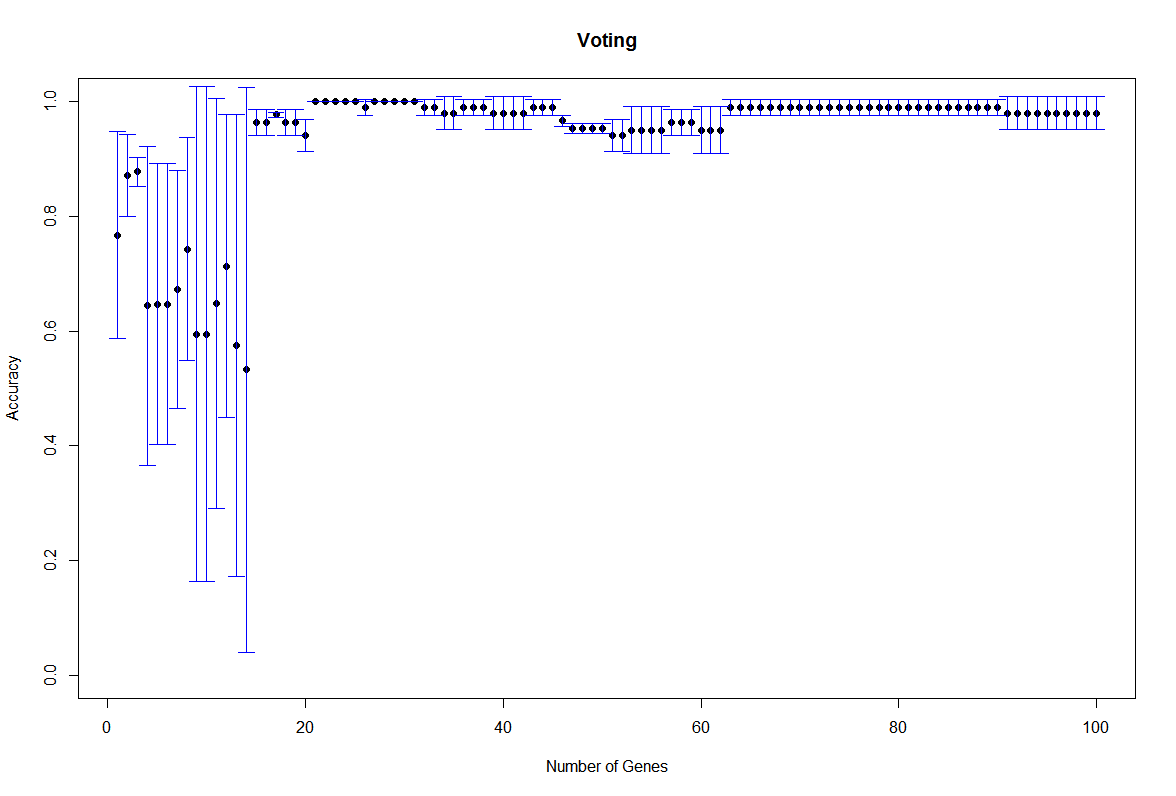


**Supplementary Figure 12:** Mean accuracy rate, with error bars, of the sequential gene selection the top 100 re-ranked genes with the ensemble Voting method, using hold out validation with train set the TCGA expression values and test set the expression values from 2 GEO independent datasets for breast cancer Stage II.


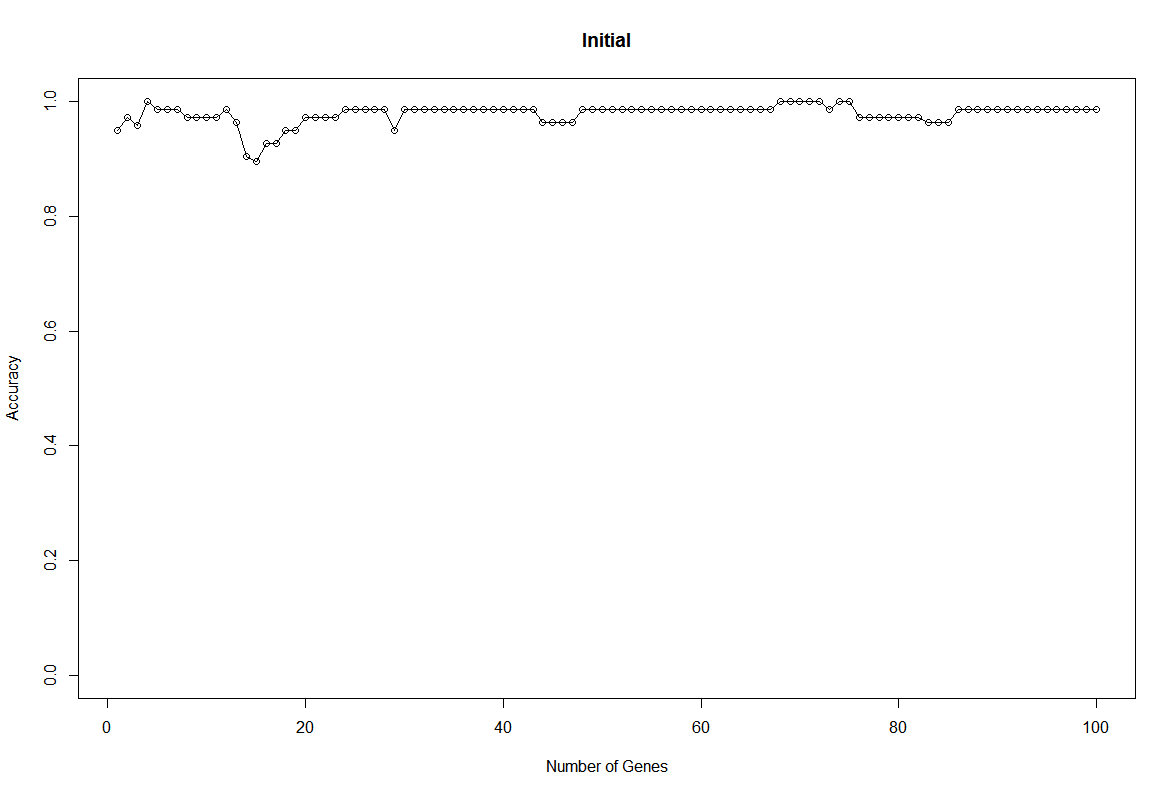


**Supplementary Figure 13:** Mean accuracy rate of the sequential gene selection from the top 100 Initial ranked genes (from Limma), using hold out validation with train set the TCGA expression values and test set the expression values from 2 GEO independent datasets for breast cancer Stage III.


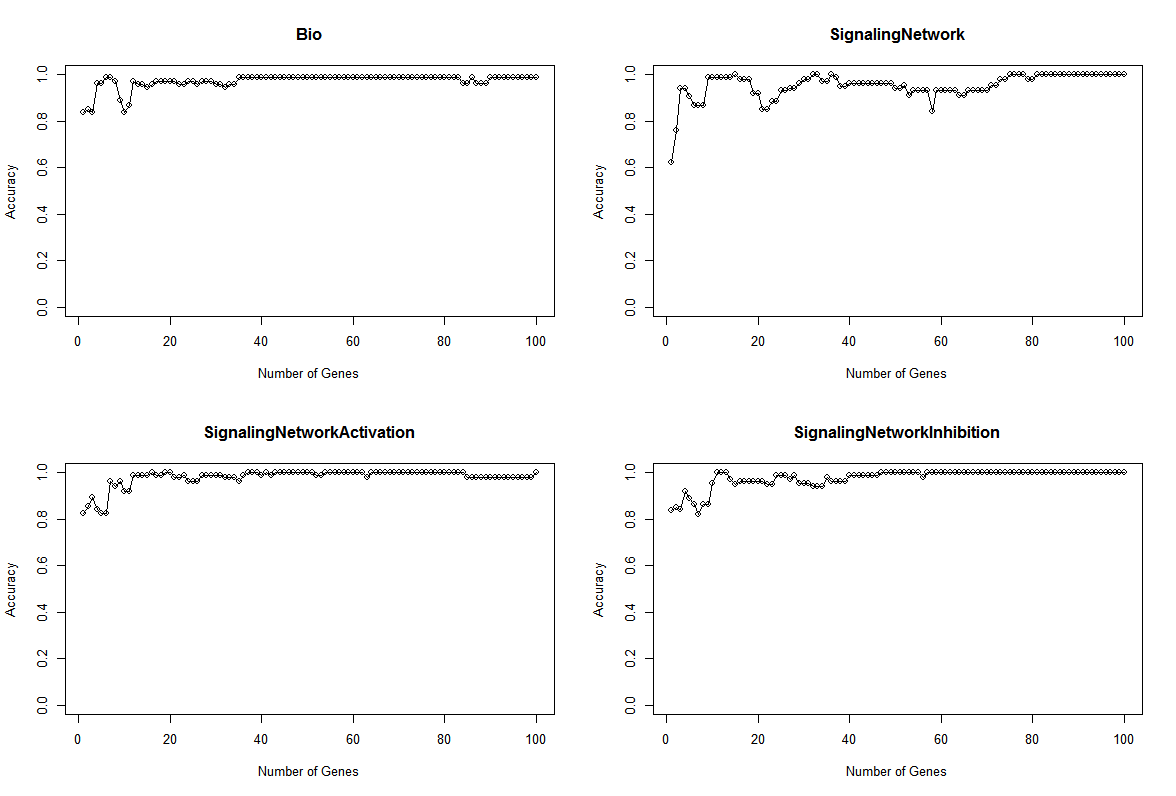


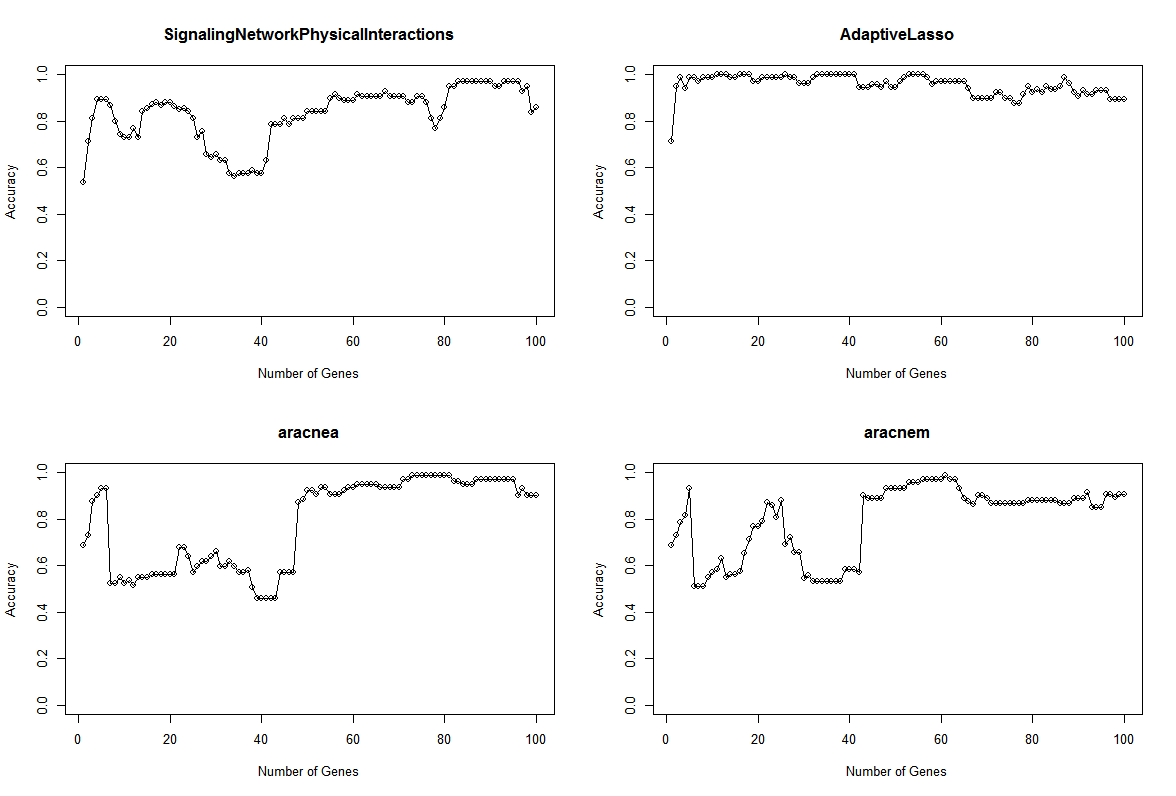


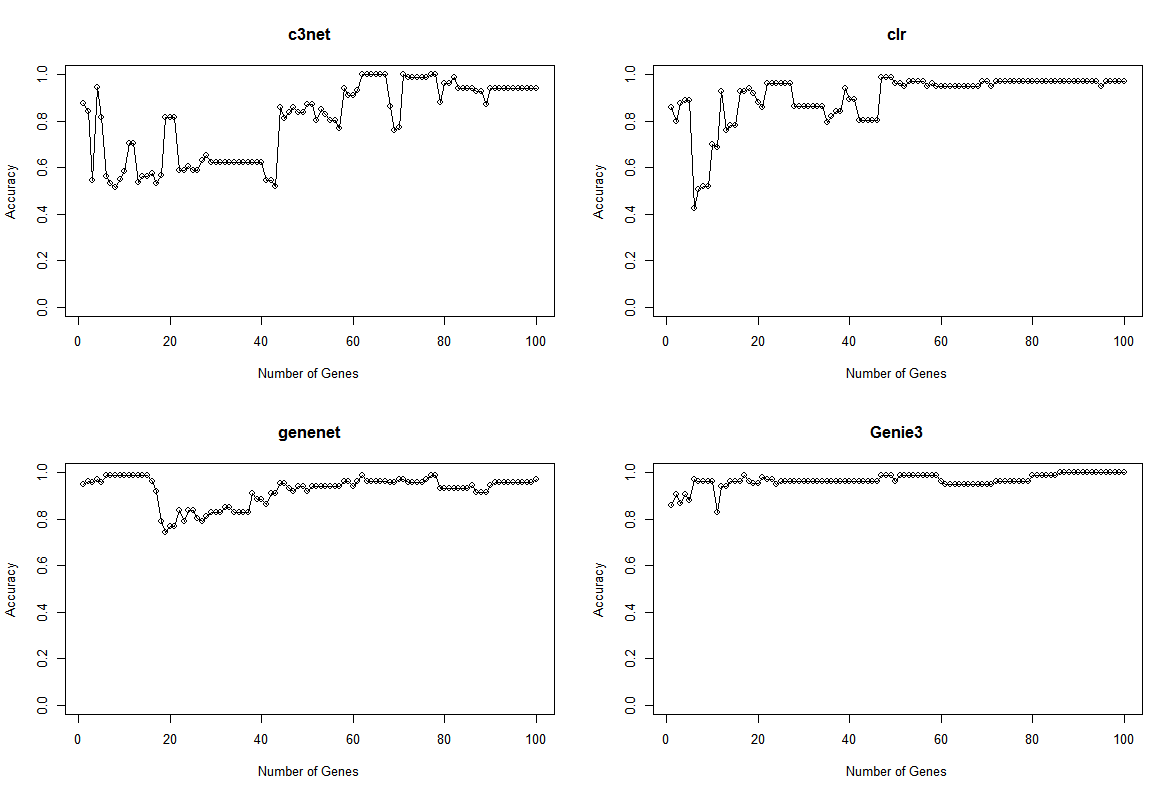


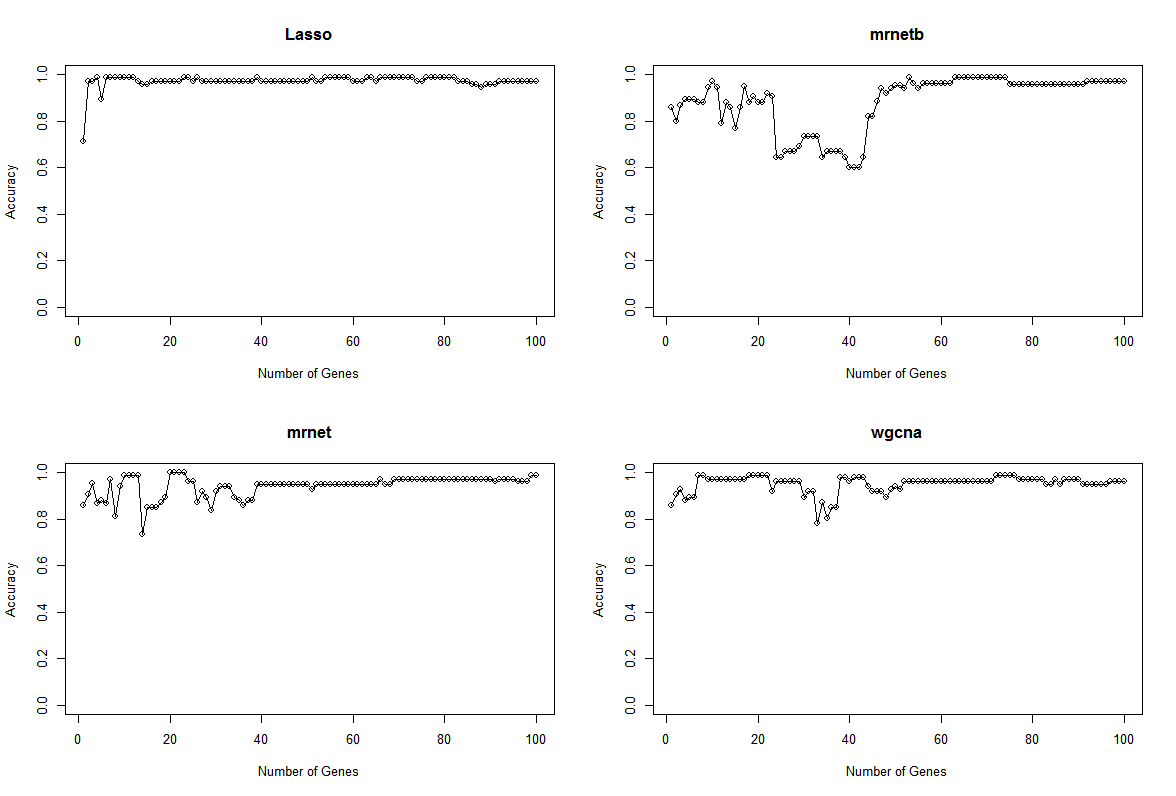


**Supplementary Figure 14:** Mean accuracy rates of the top 100 sequential genes from all ranked and re-ranked gene lists from each method in combination with PageRank reconciling method, using hold out validation with train set the TCGA expression values and test set the expression values from 2 GEO independent datasets for breast cancer Stage III.


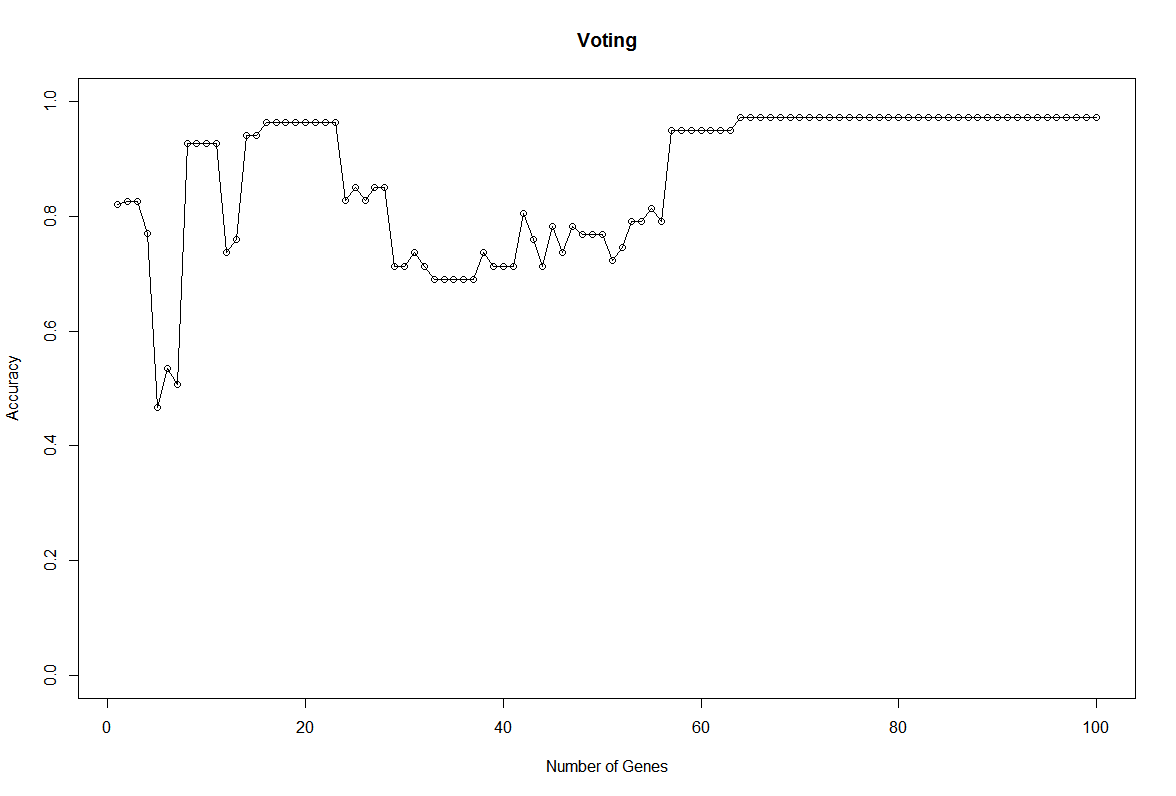


**Supplementary Figure 15:** Mean accuracy rate of the sequential gene selection from the top 100 re-ranked genes with the ensemble Voting method, using hold out validation with train set the TCGA expression values and test set the expression values from 2 GEO independent datasets for breast cancer Stage III.


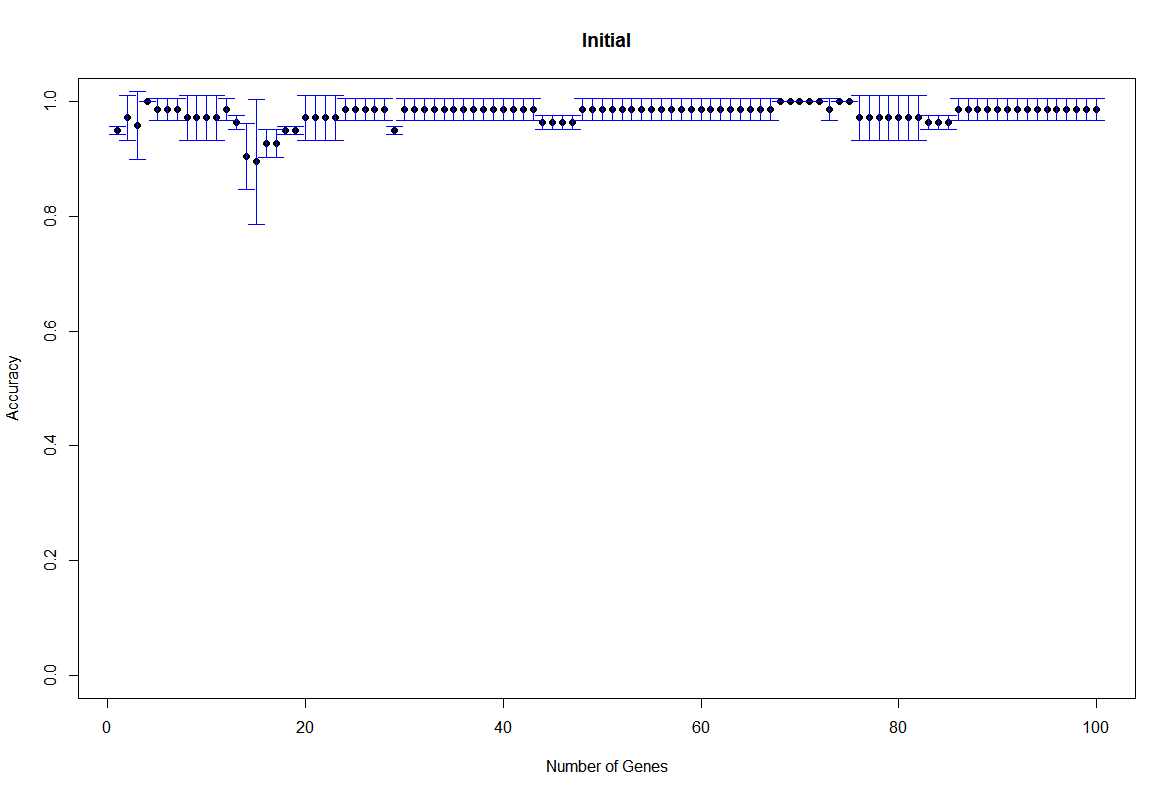


**Supplementary Figure 16:** Mean accuracy rate, with error bars, of the sequential gene selection from the top 100 Initial ranked genes (from Limma), using hold out validation with train set the TCGA expression values and test set the expression values from 2 GEO independent datasets for breast cancer Stage III.


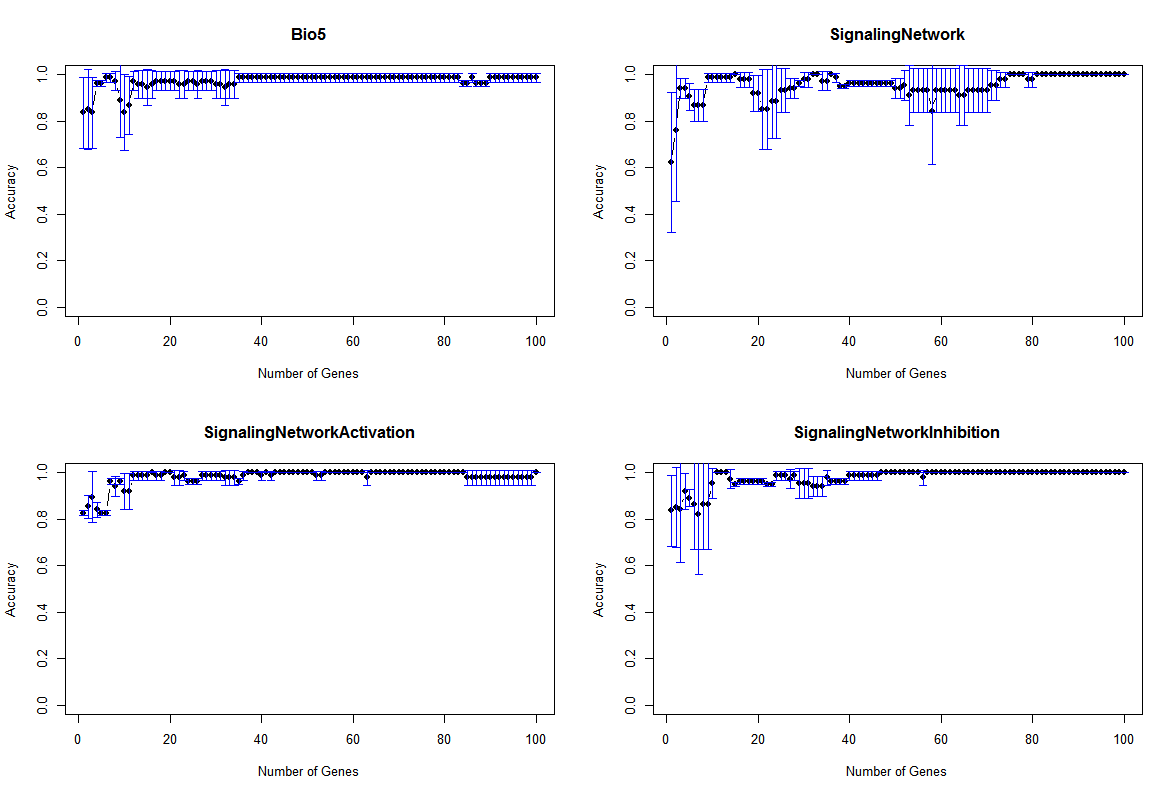


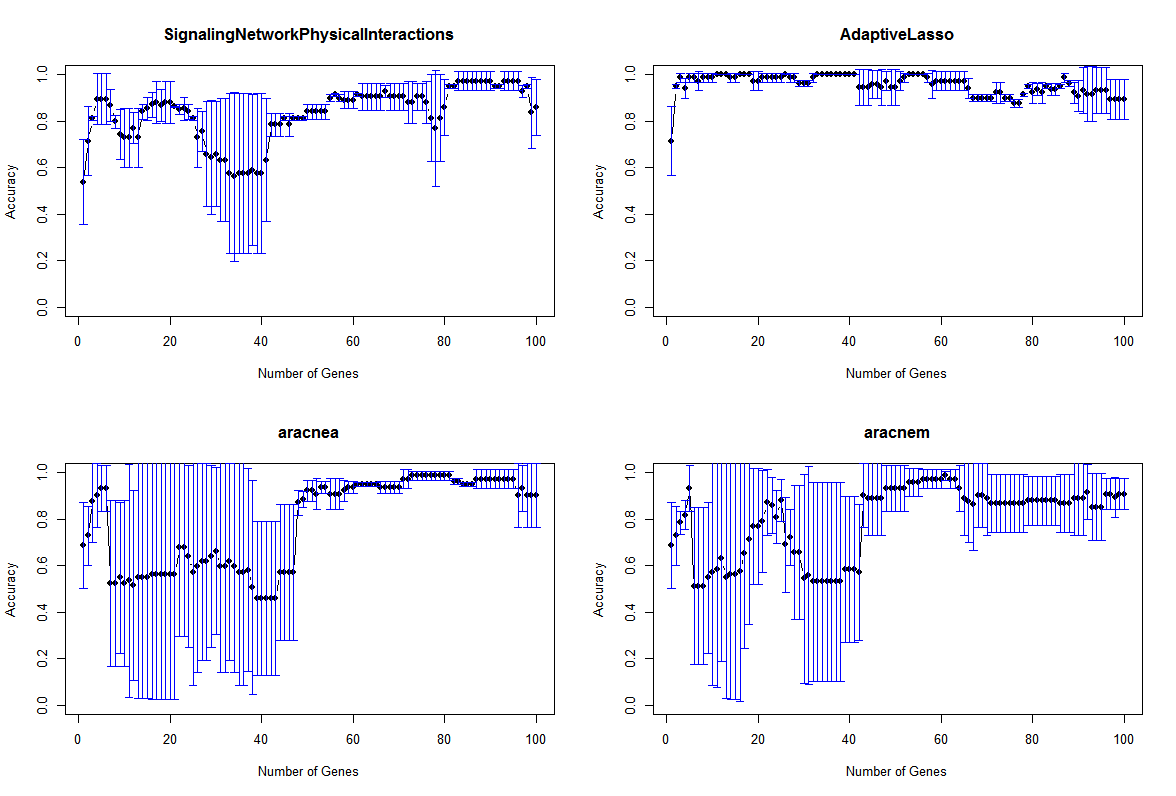


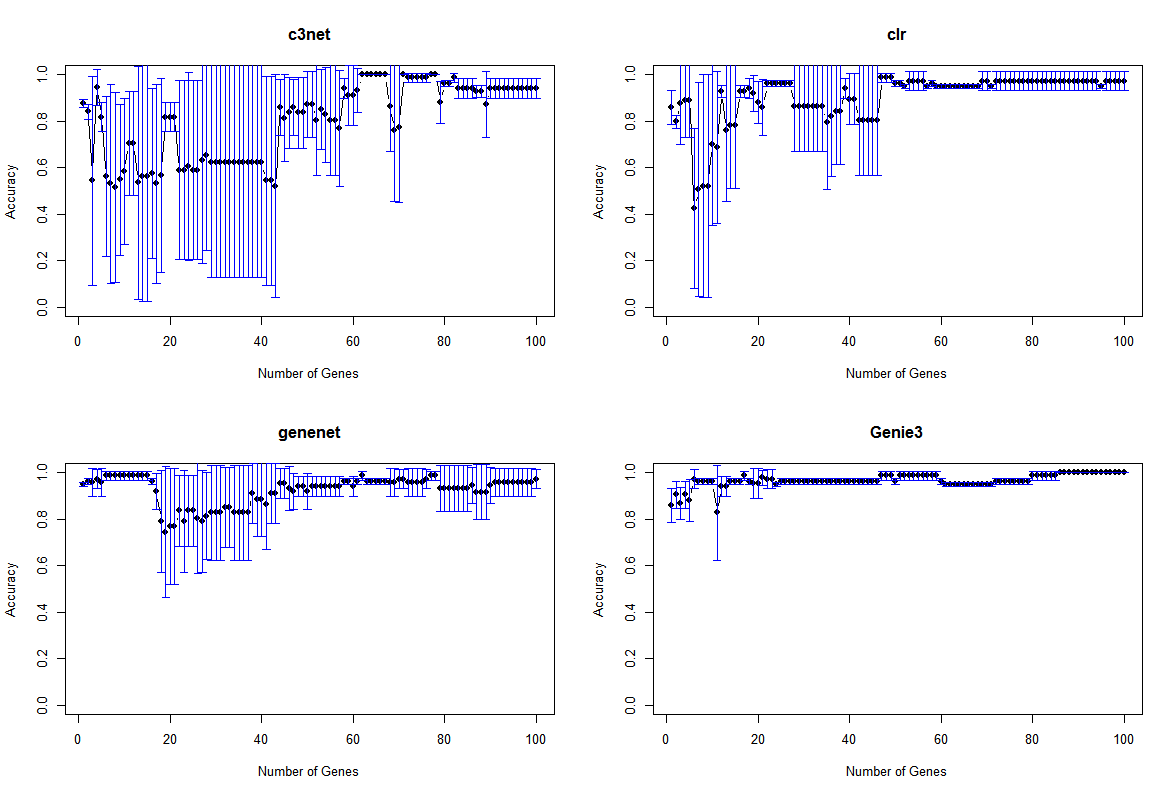


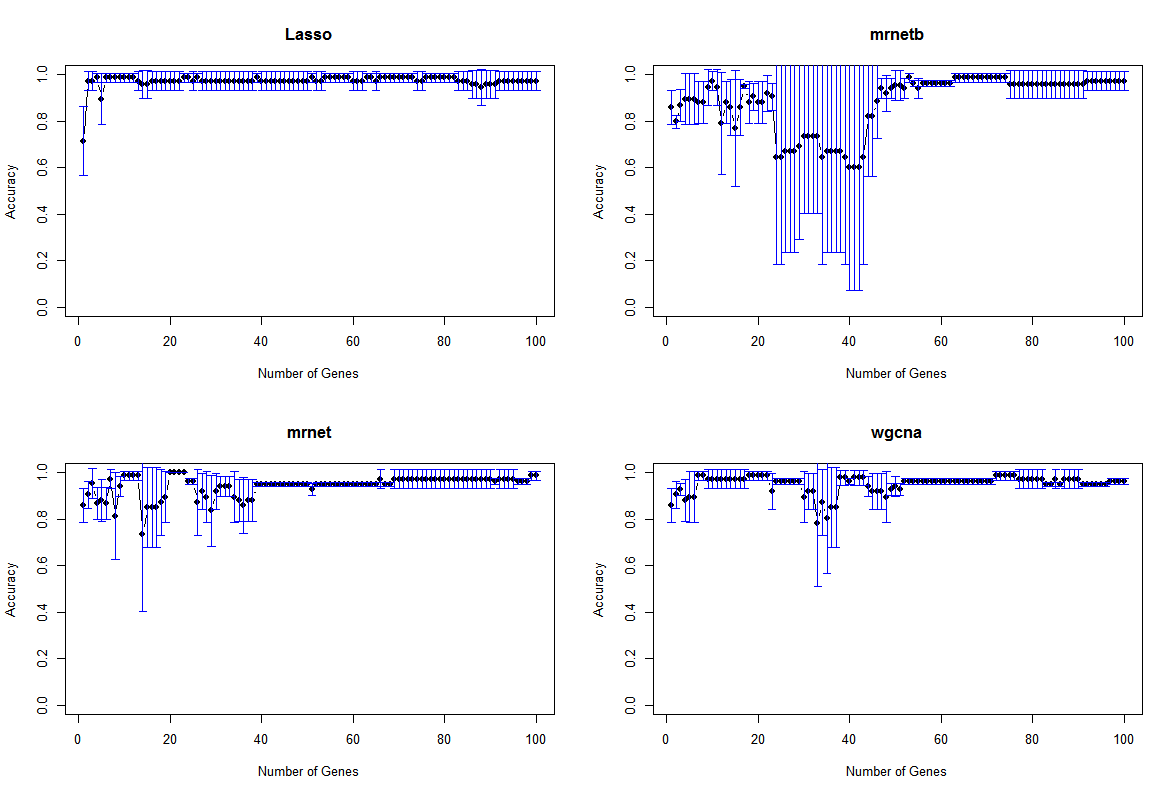


**Supplementary Figure 17:** Mean accuracy rates, with error bars, of the top 100 sequential genes from all ranked and re-ranked gene lists from each method in combination with PageRank reconciling method, using hold out validation with train set the TCGA expression values and test set the expression values from 2 GEO independent datasets breast cancer Stage III.


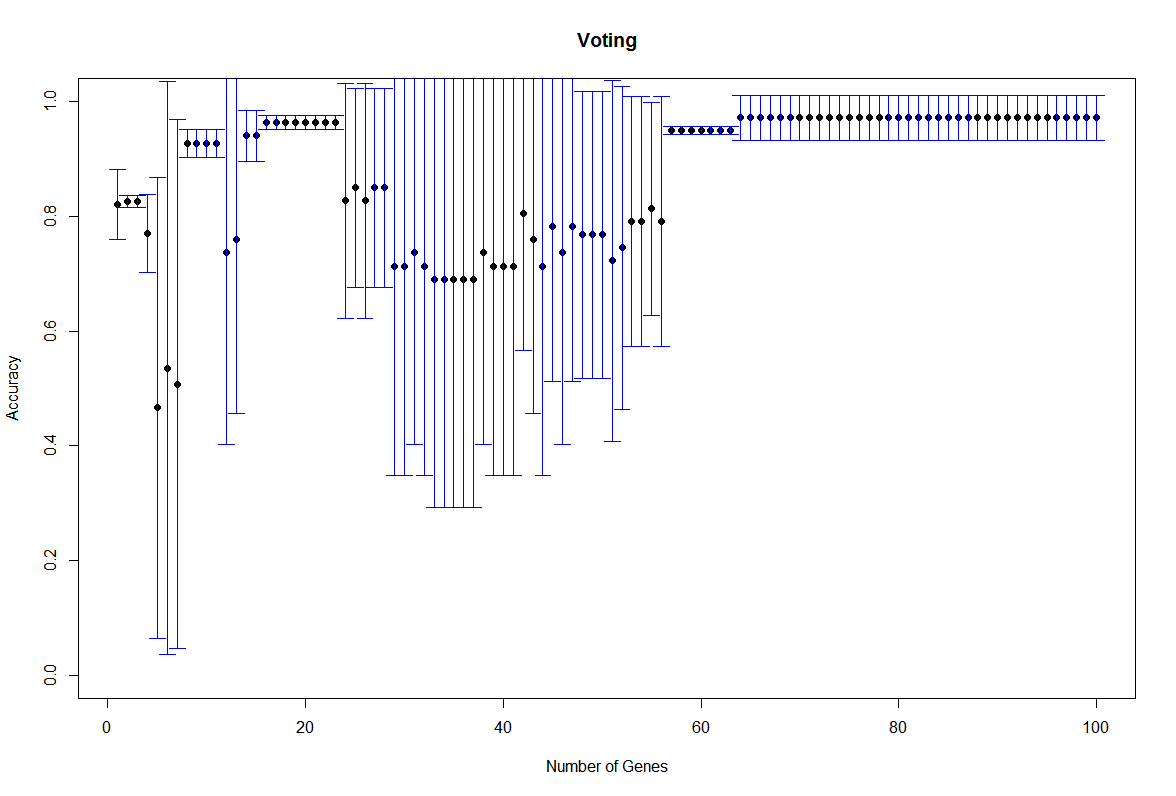


**Supplementary Figure 18:** Mean accuracy rate, with error bars, of the sequential gene selection the top 100 re-ranked genes with the ensemble Voting method, using hold out validation with train set the TCGA expression values and test set the expression values from 2 GEO independent datasets for breast cancer Stage III.

**
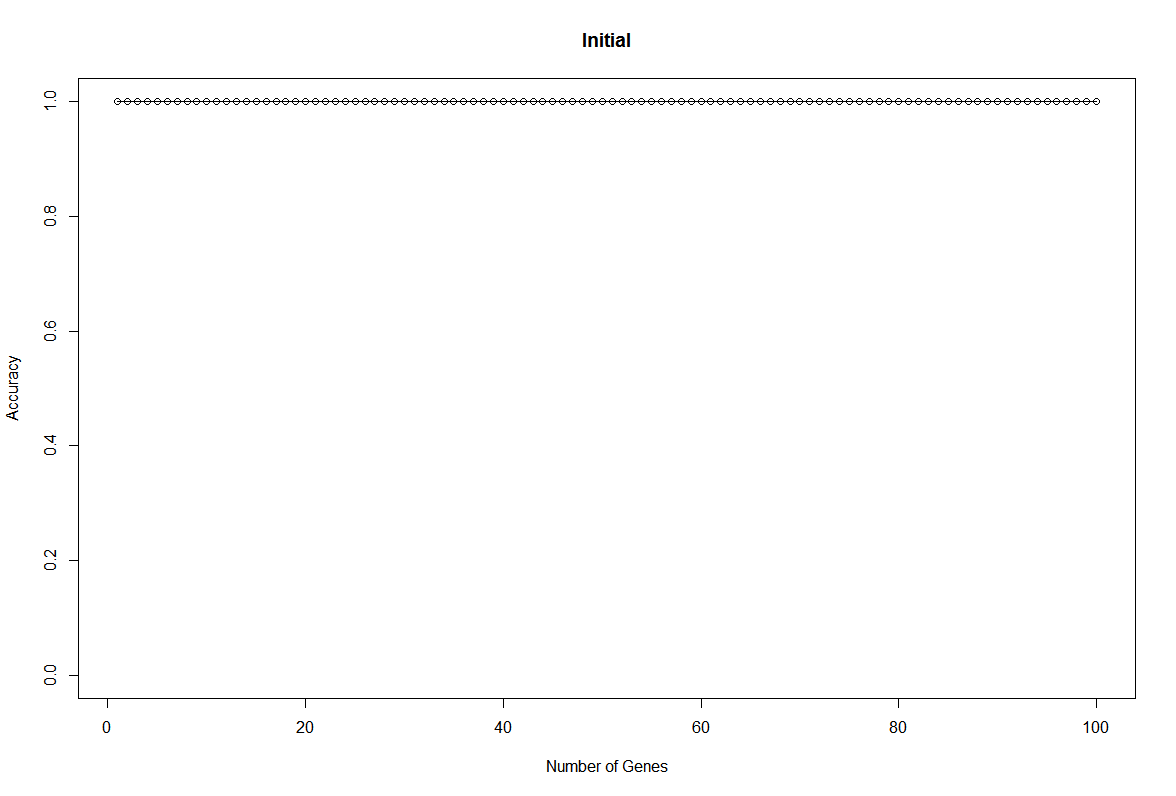
**

**Supplementary Figure 19:** Mean accuracy rate of the sequential gene selection from the top 100 Initial ranked genes (from Limma), using hold out validation with train set the TCGA expression values and test set the expression values from 1 GEO independent datasets for breast cancer Stage IV.

**
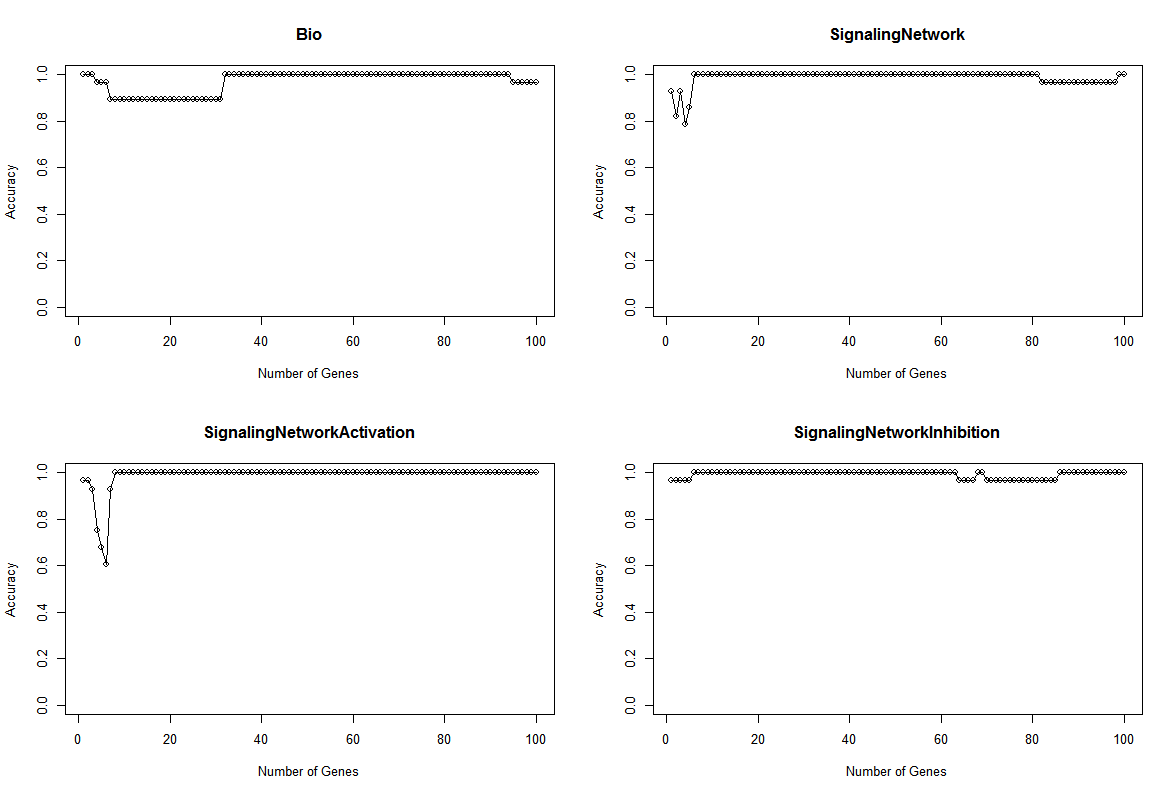
**

**
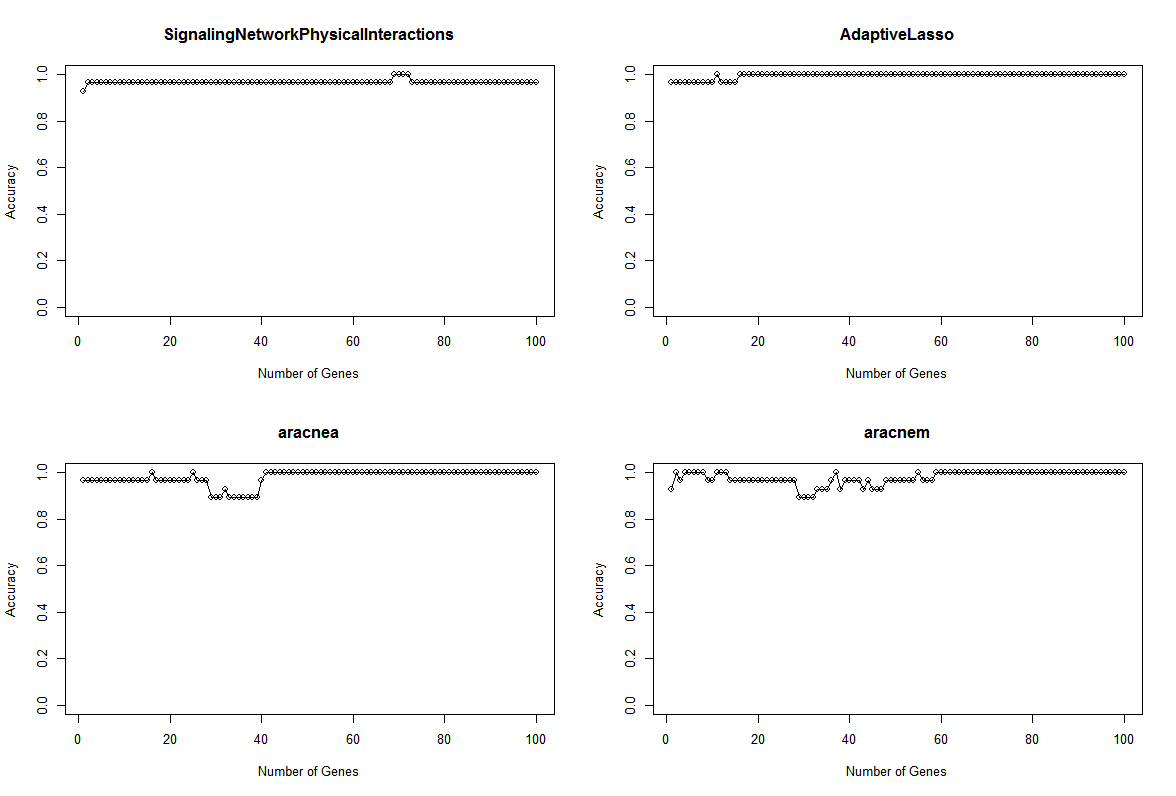
**

**
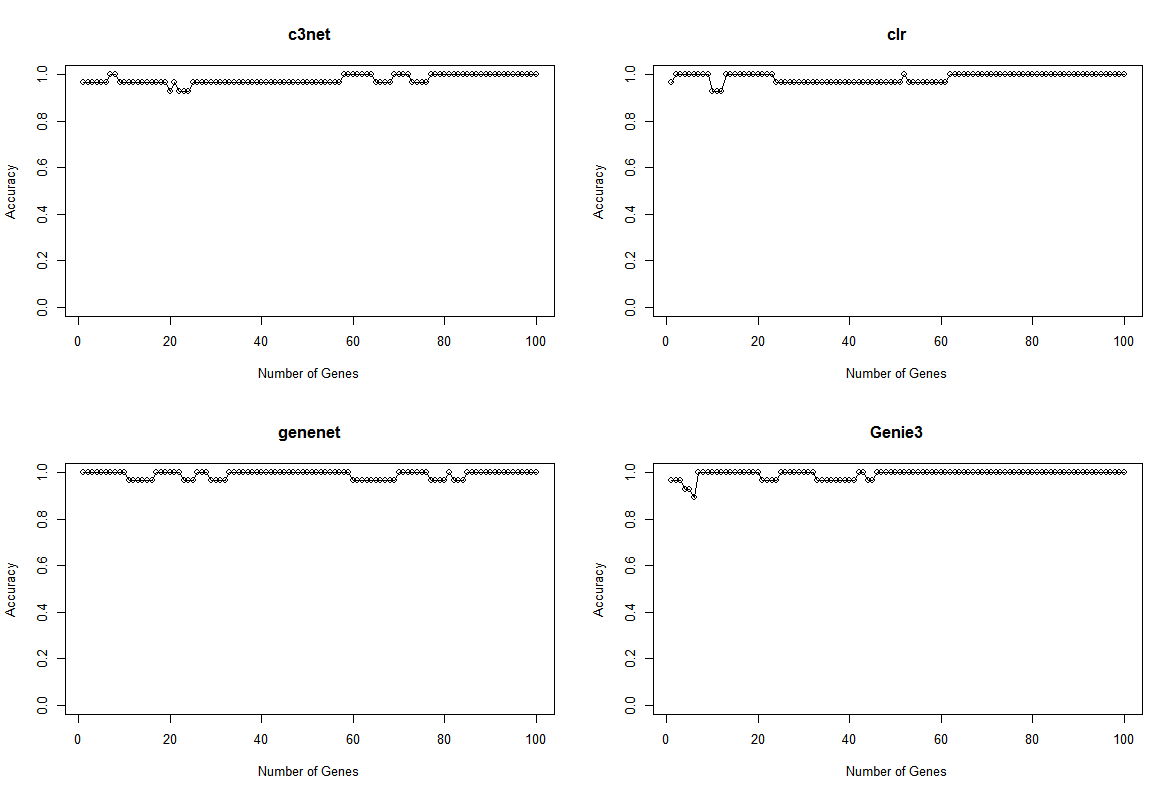
**

**
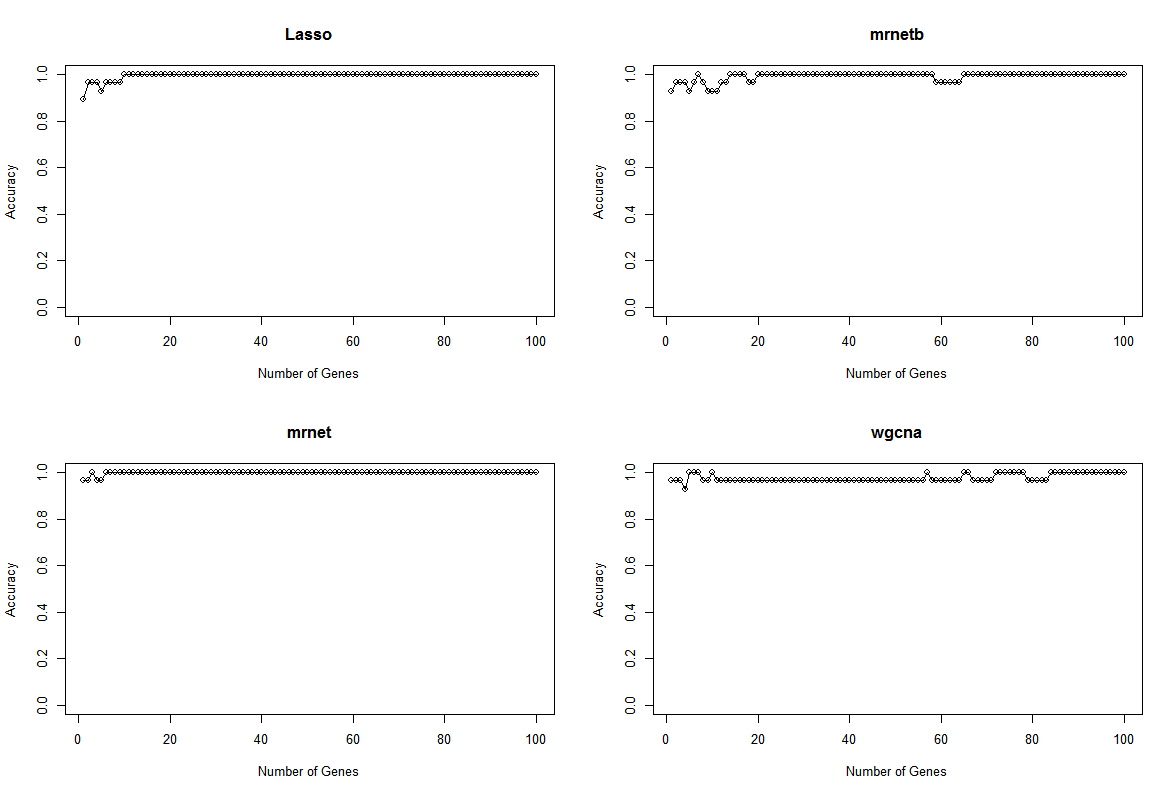
**

**Supplementary Figure 20:** Mean accuracy rates of the top 100 sequential genes from all ranked and re-ranked gene lists from each method in combination with PageRank reconciling method, using hold out validation with train set the TCGA expression values and test set the expression values from 1 GEO independent datasets for breast cancer Stage IV.

**
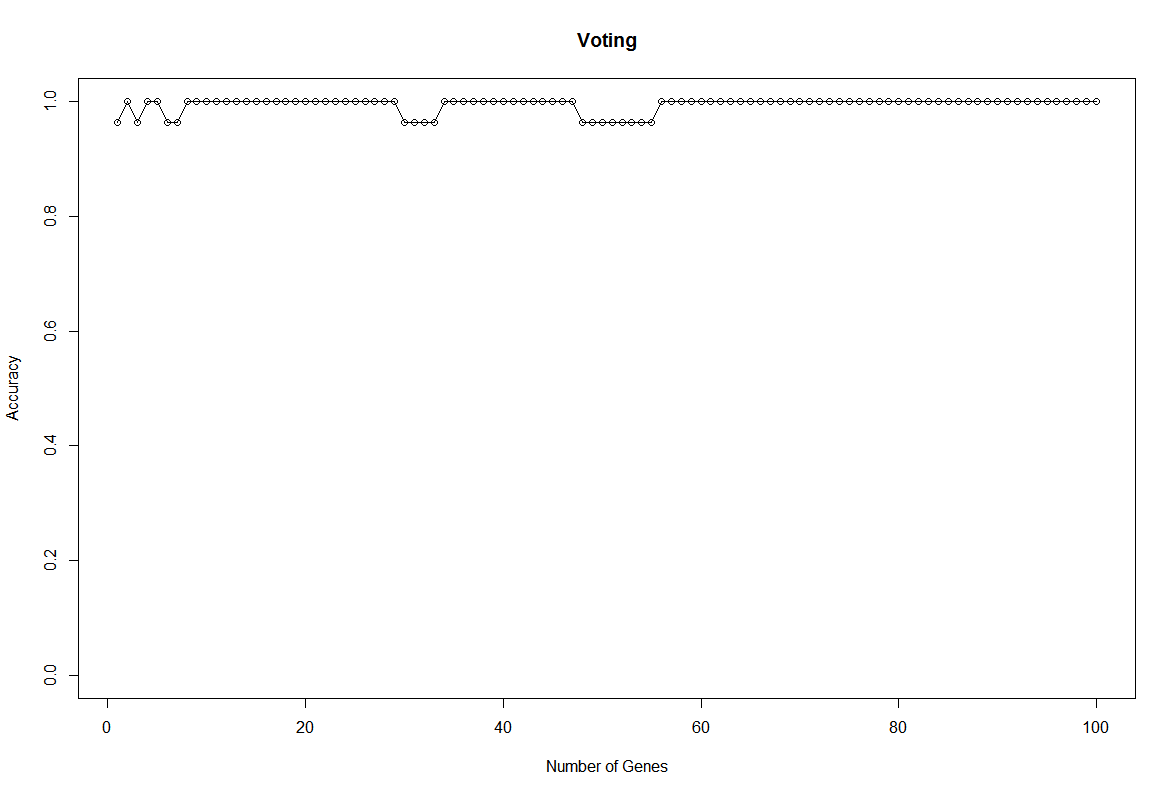
**

**Supplementary Figure 21:** Mean accuracy rate of the sequential gene selection from the top 100 re-ranked genes with the ensemble Voting method, using hold out validation with train set the TCGA expression values and test set the expression values from 1 GEO independent datasets for breast cancer Stage IV.


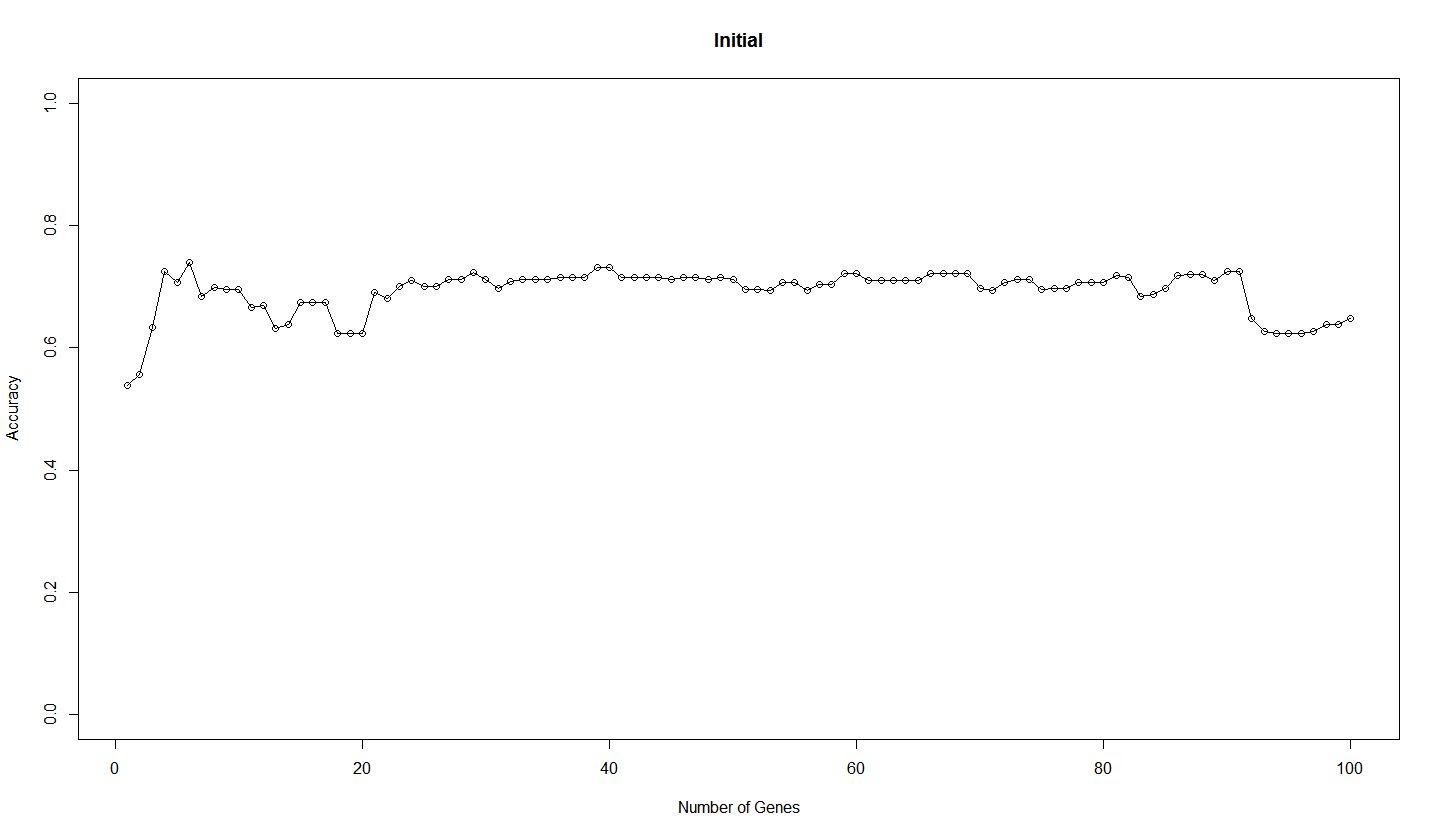


**Supplementary Figure 22:** Mean accuracy rate of the sequential gene selection from the top 100 Initial ranked genes (from Limma), using hold out validation with train set the TCGA expression values and test set the expression values from 6 GEO independent datasets for Triple Negative subtype of breast cancer.


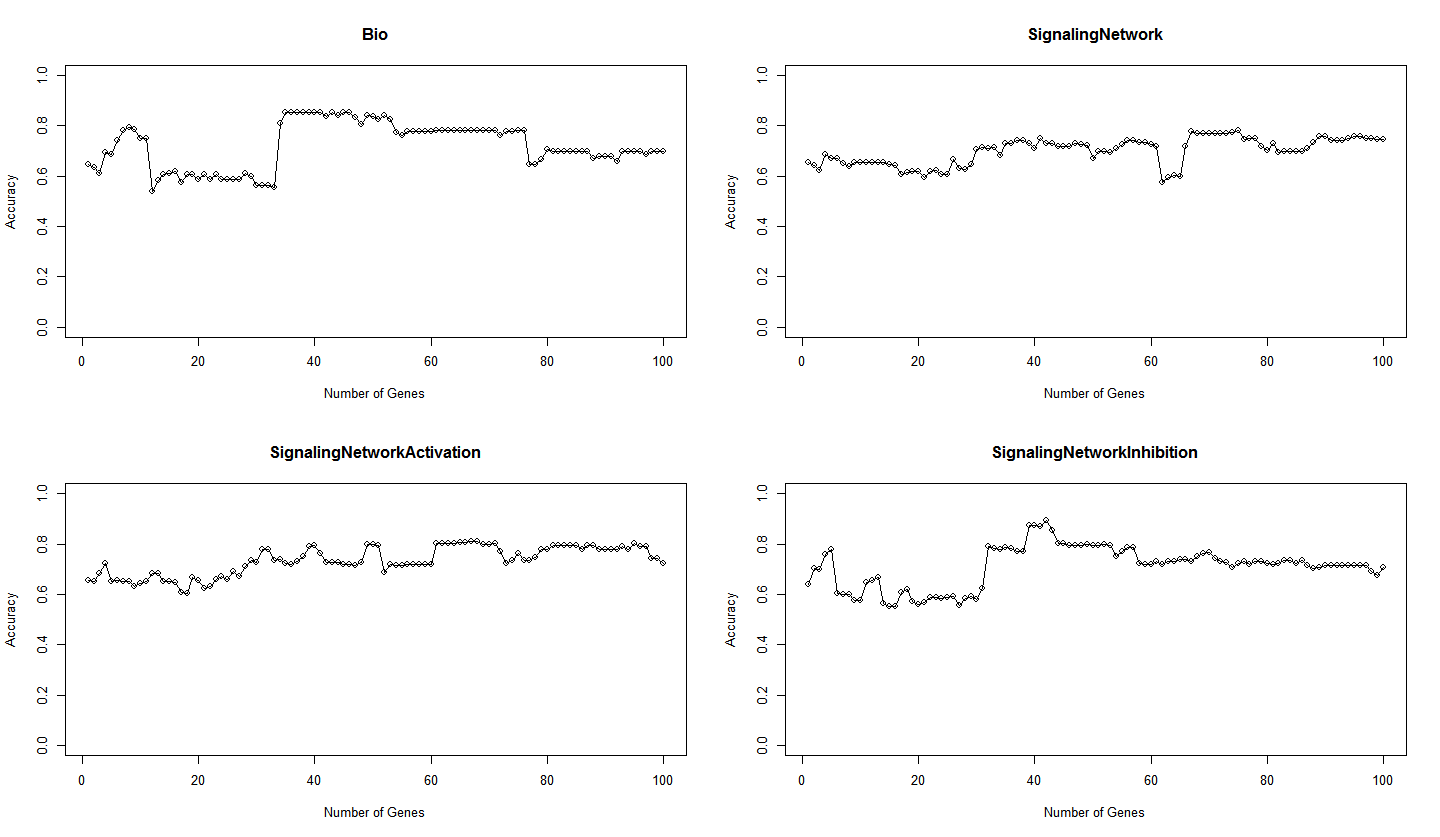


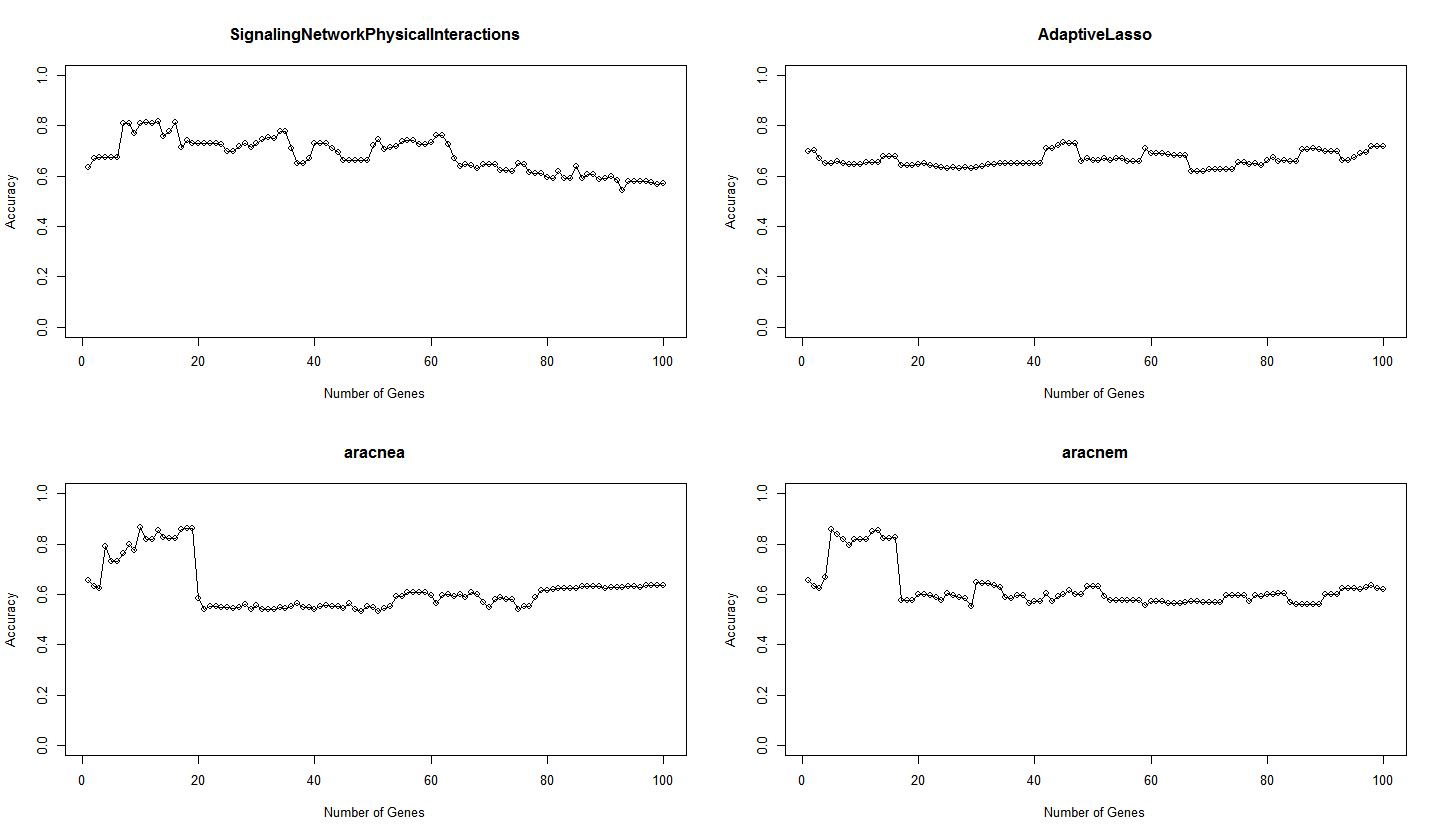


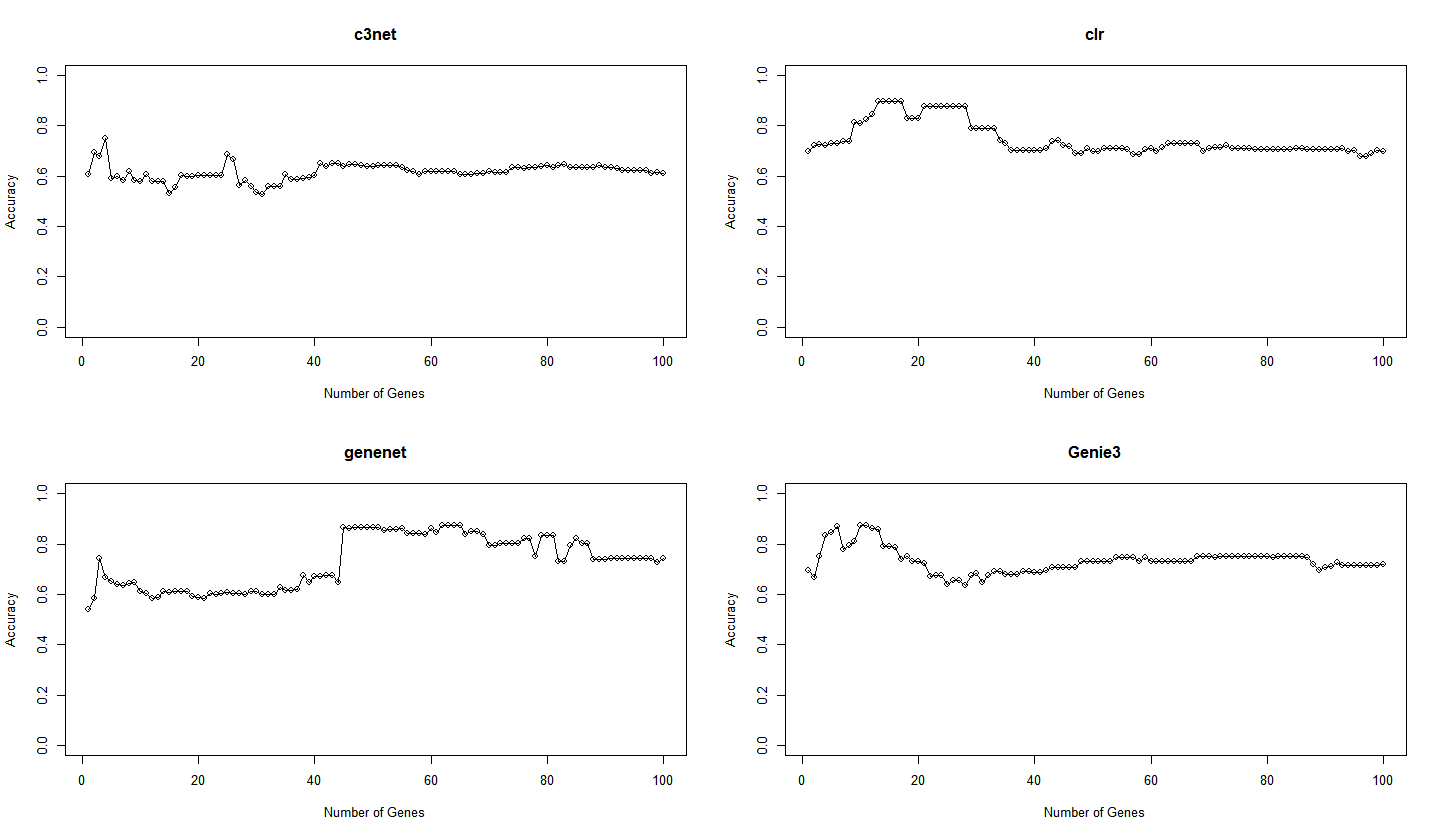

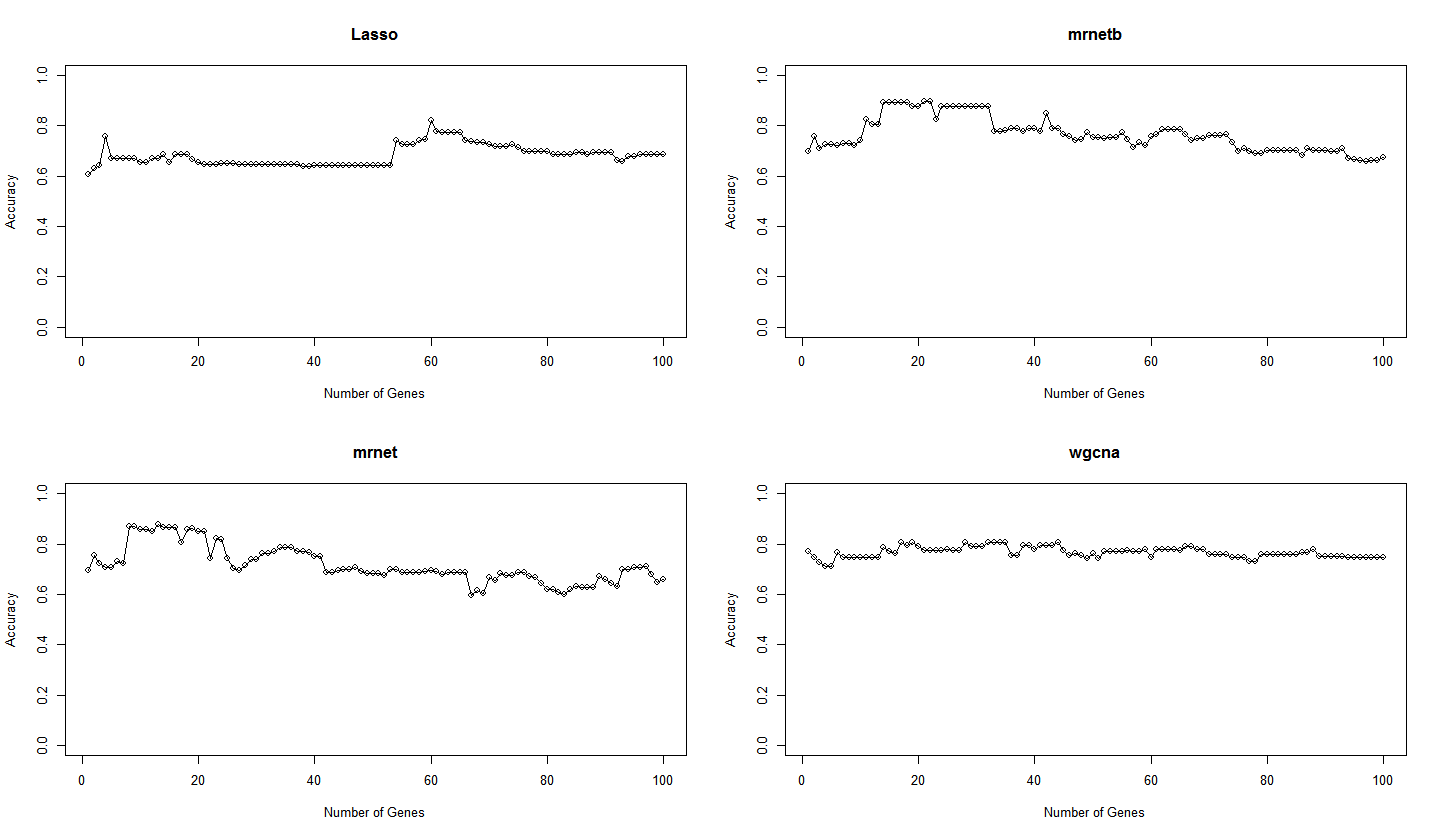


**Supplementary Figure 23:** Mean accuracy rates of the top 100 sequential genes from all ranked and re-ranked gene lists from each method in combination with PageRank reconciling method, using hold out validation with train set the TCGA expression values and test set the expression values from 6 GEO independent datasets for Triple Negative subtype of breast cancer.


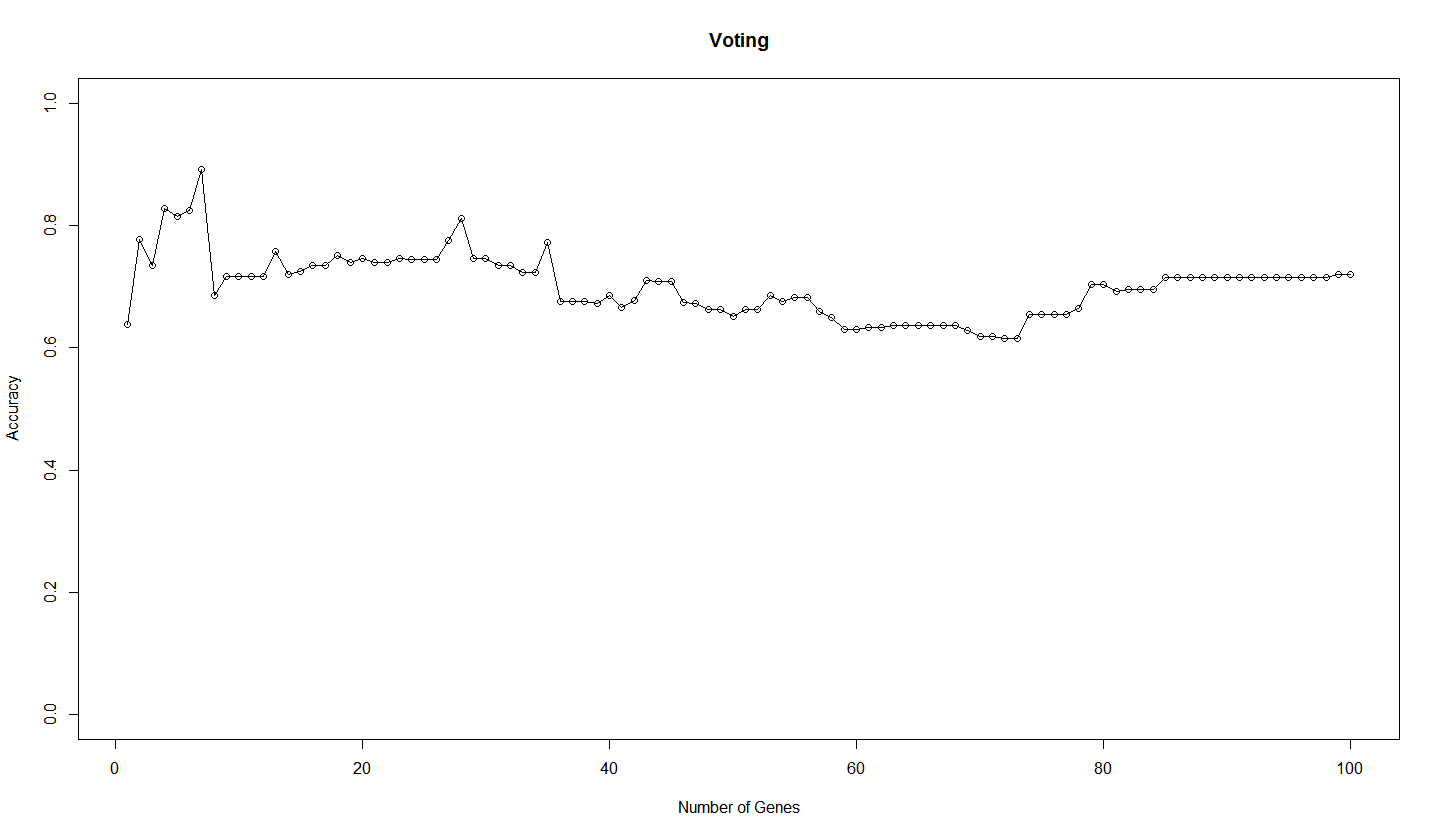


**Supplementary Figure 24:** Mean accuracy rate of the sequential gene selection from the top 100 re-ranked genes with the ensemble Voting method, using hold out validation with train set the TCGA expression values and test set the expression values from 6 GEO independent datasets for Triple Negative subtype of breast cancer.


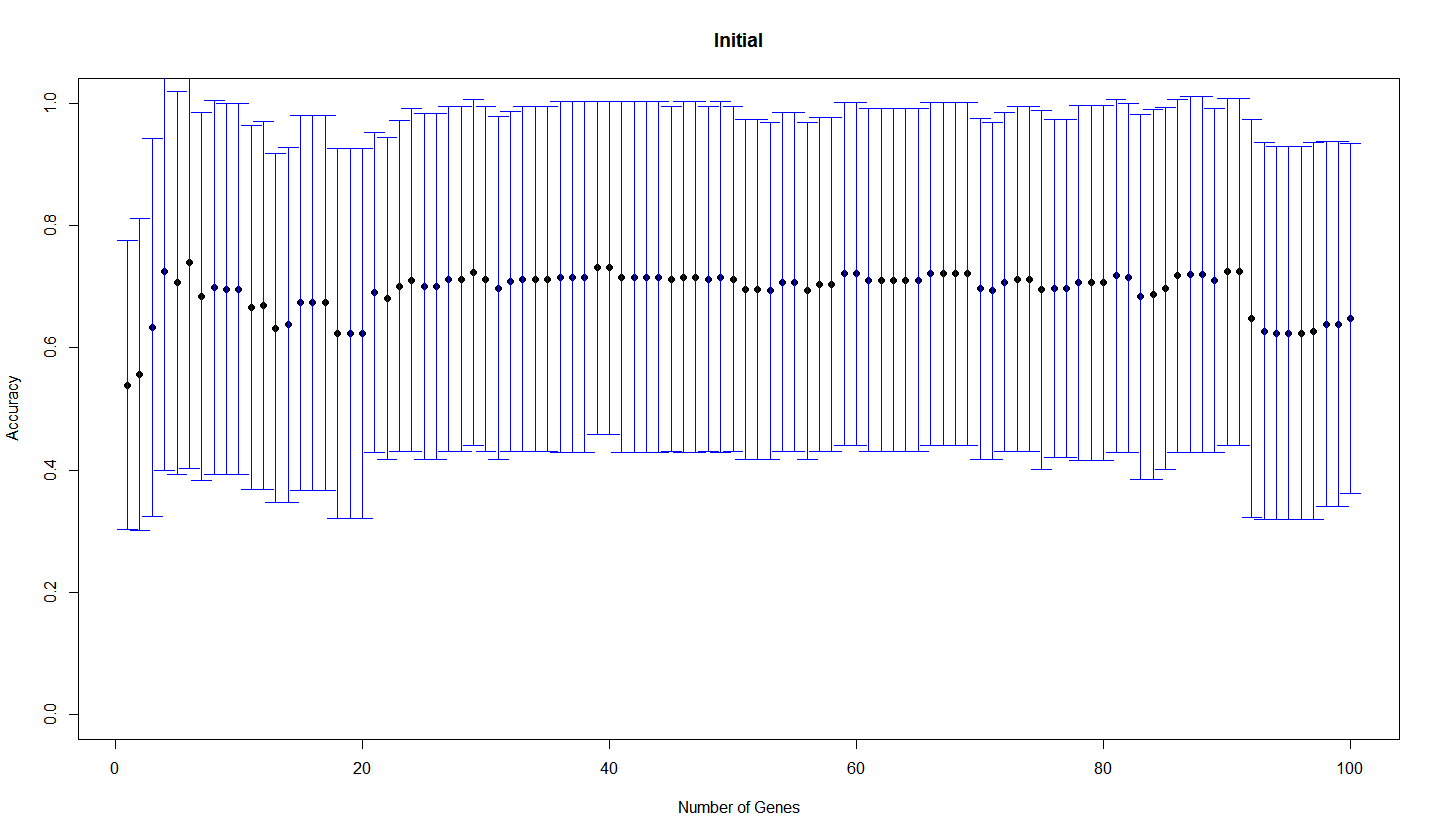


**Supplementary Figure 25:** Mean accuracy rate, with error bars, of the sequential gene selection from the top 100 Initial ranked genes (from Limma), using hold out validation with train set the TCGA expression values and test set the expression values from 6 GEO independent datasets for Triple Negative subtype of breast cancer.


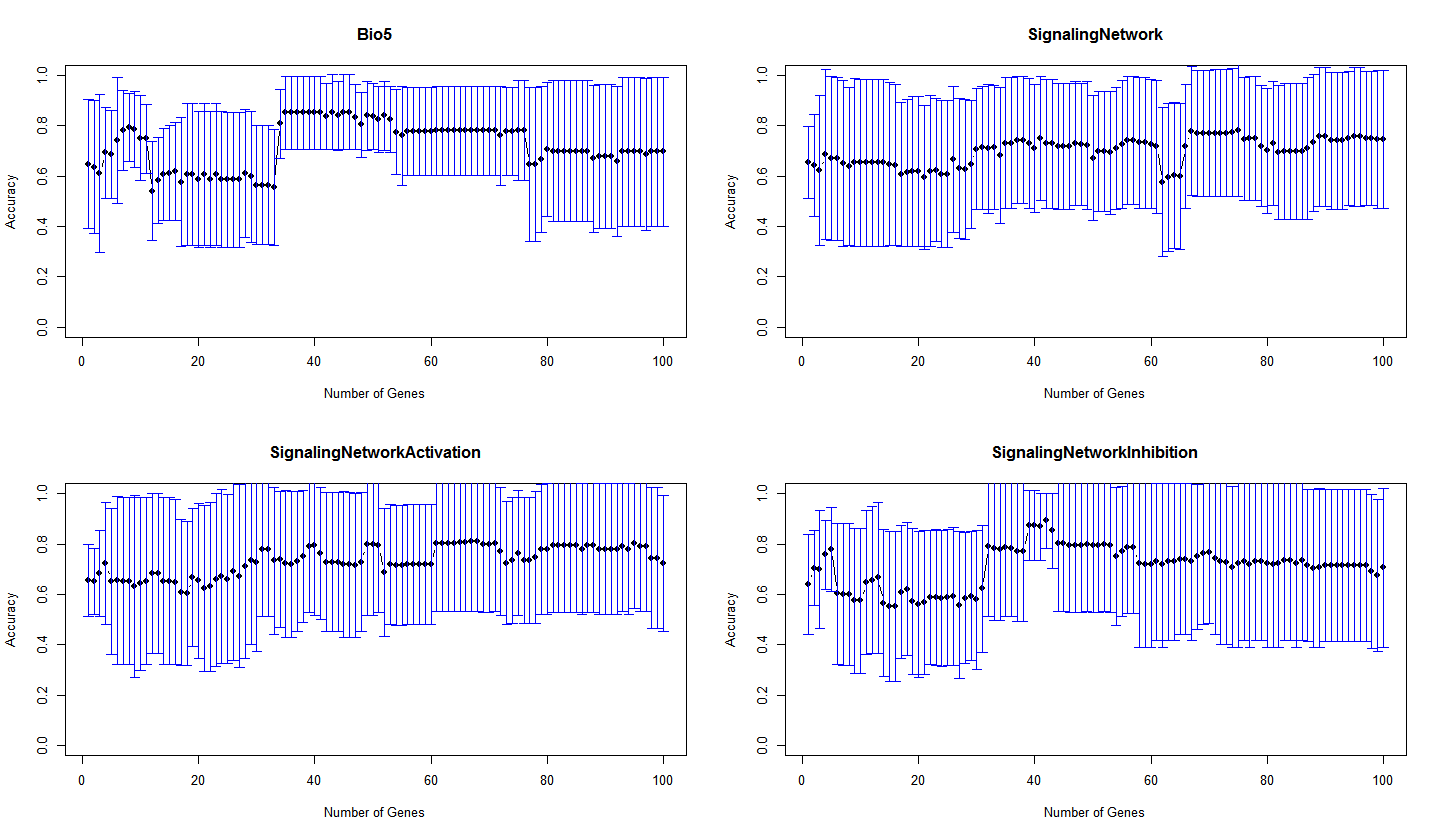


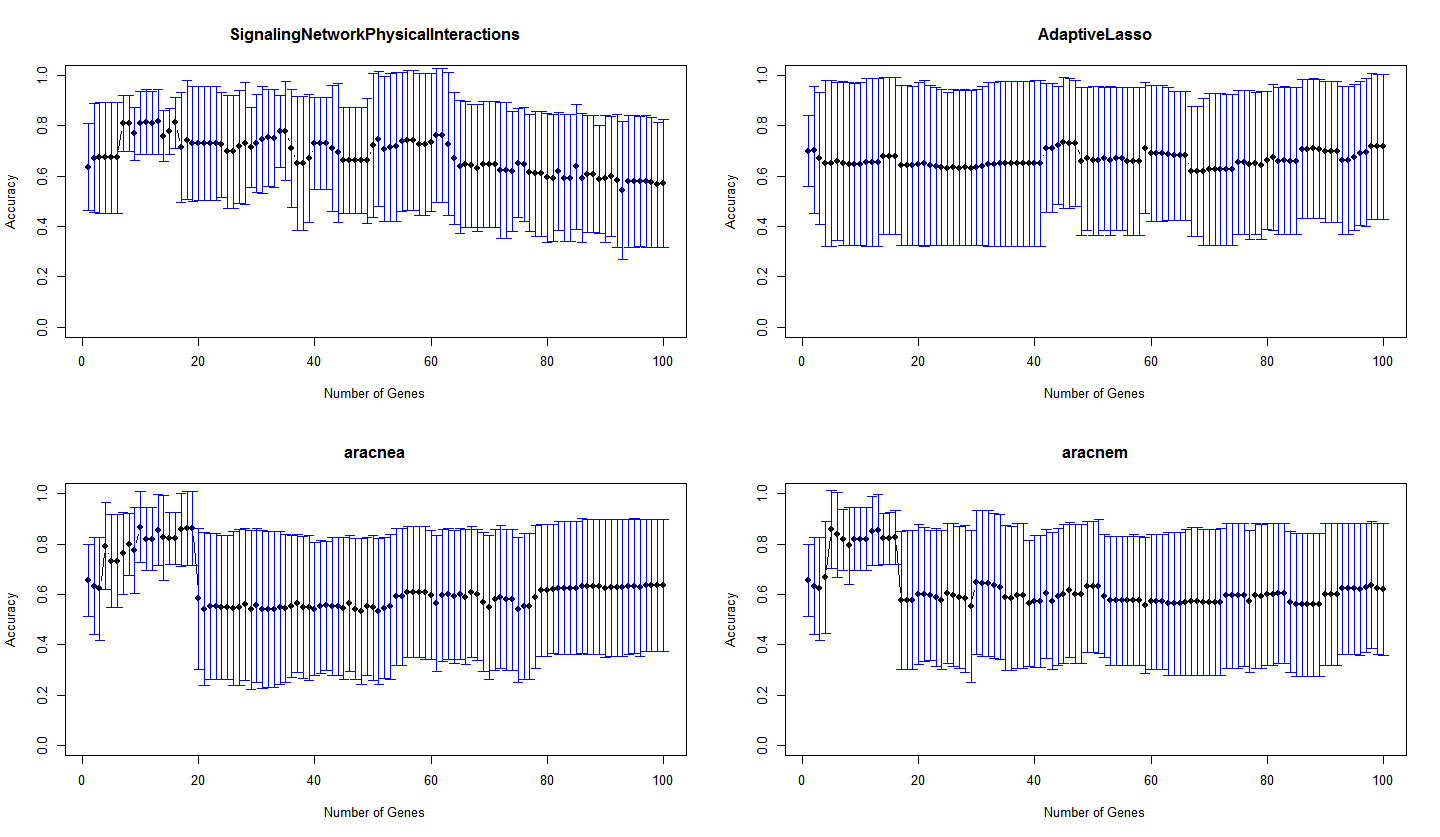


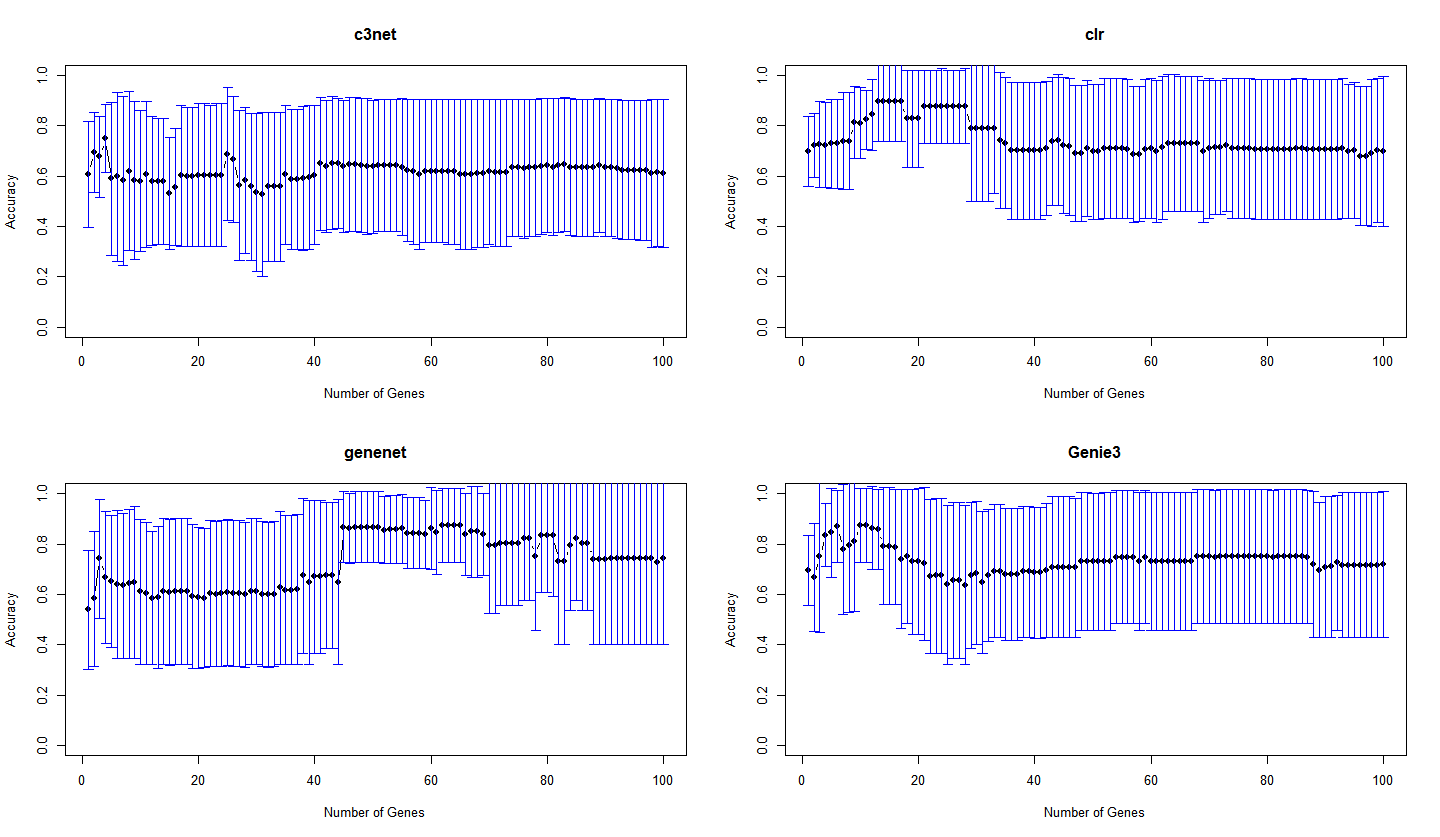


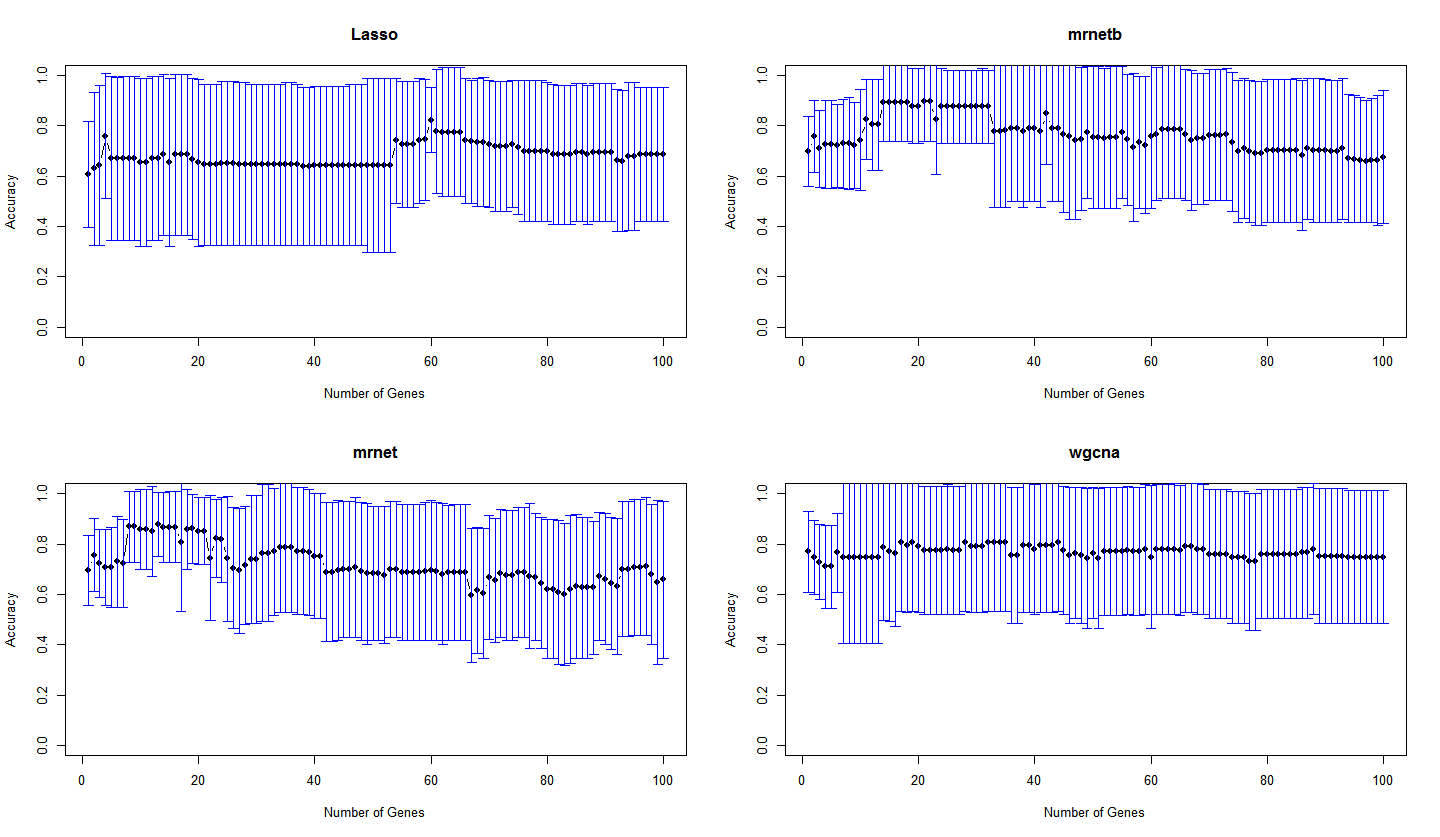


**Supplementary Figure 26:** Mean accuracy rates, with error bars, of the top 100 sequential genes from all ranked and re-ranked gene lists from each method in combination with PageRank reconciling method, using hold out validation with train set the TCGA expression values and test set the expression values from 6 GEO independent datasets Triple Negative subtype of breast cancer.


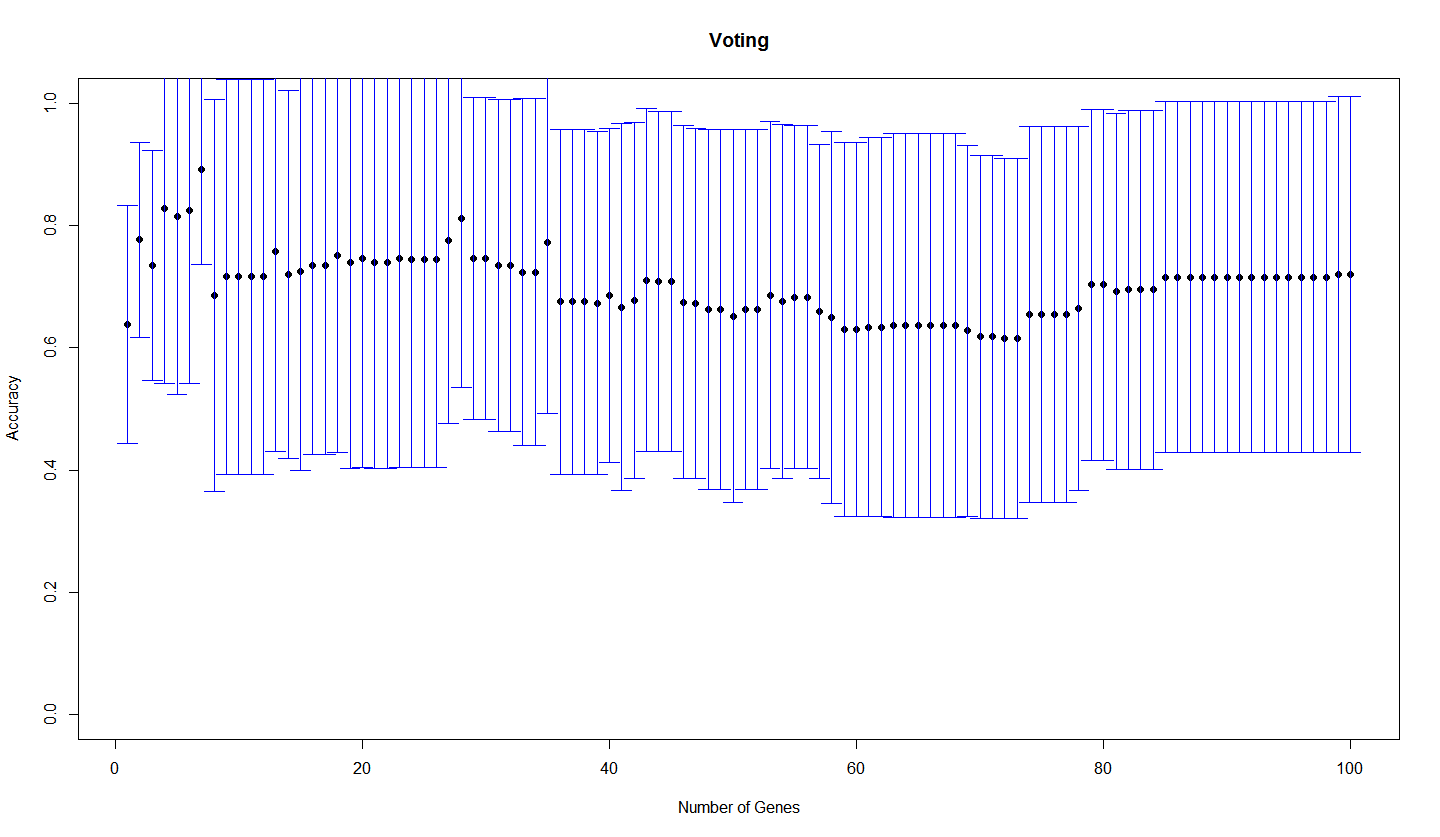


**Supplementary Figure 27:** Mean accuracy rate, with error bars, of the sequential gene selection the top 100 re-ranked genes with the ensemble Voting method, using hold out validation with train set the TCGA expression values and test set the expression values from 6 GEO independent datasets for Triple Negative subtype of breast cancer.


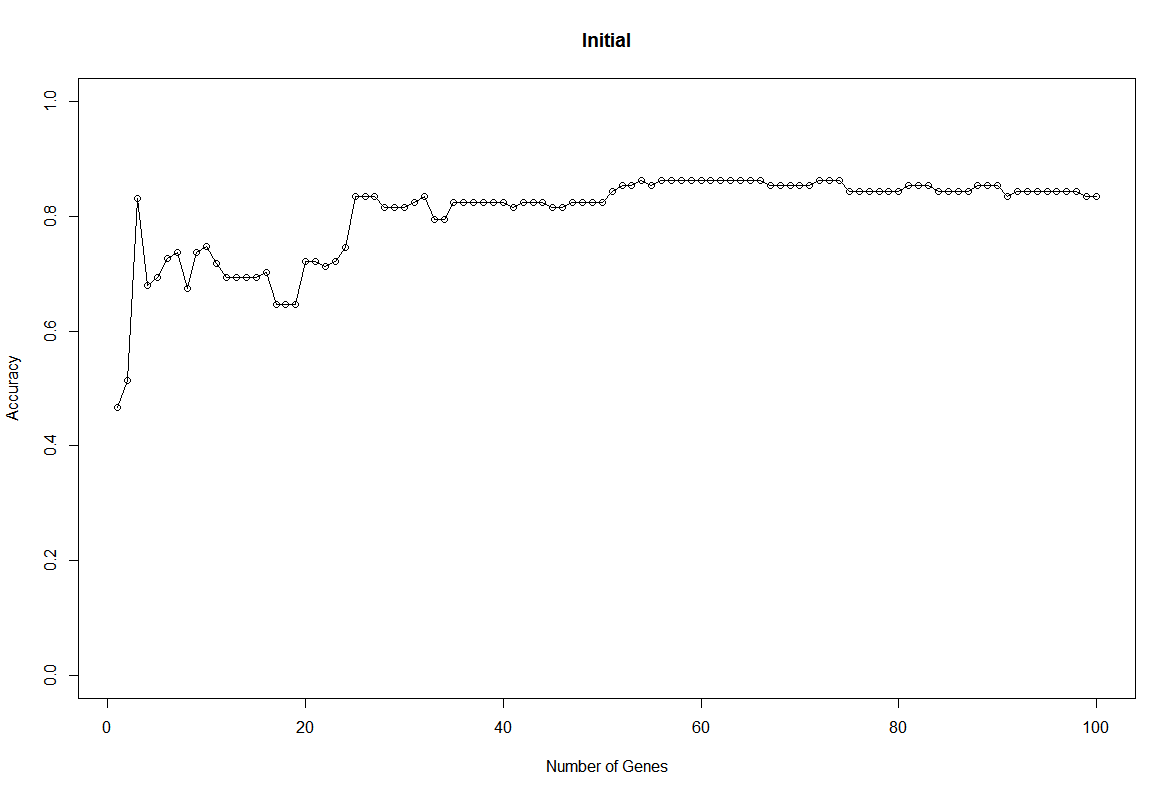


**Supplementary Figure 28:** Mean accuracy rate of the sequential gene selection from the top 100 re-ranked genes with the ensemble Voting method, using hold out validation with train set the TCGA expression values and test set the expression values from 4 GEO independent datasets for Luminal A subtype of breast cancer.


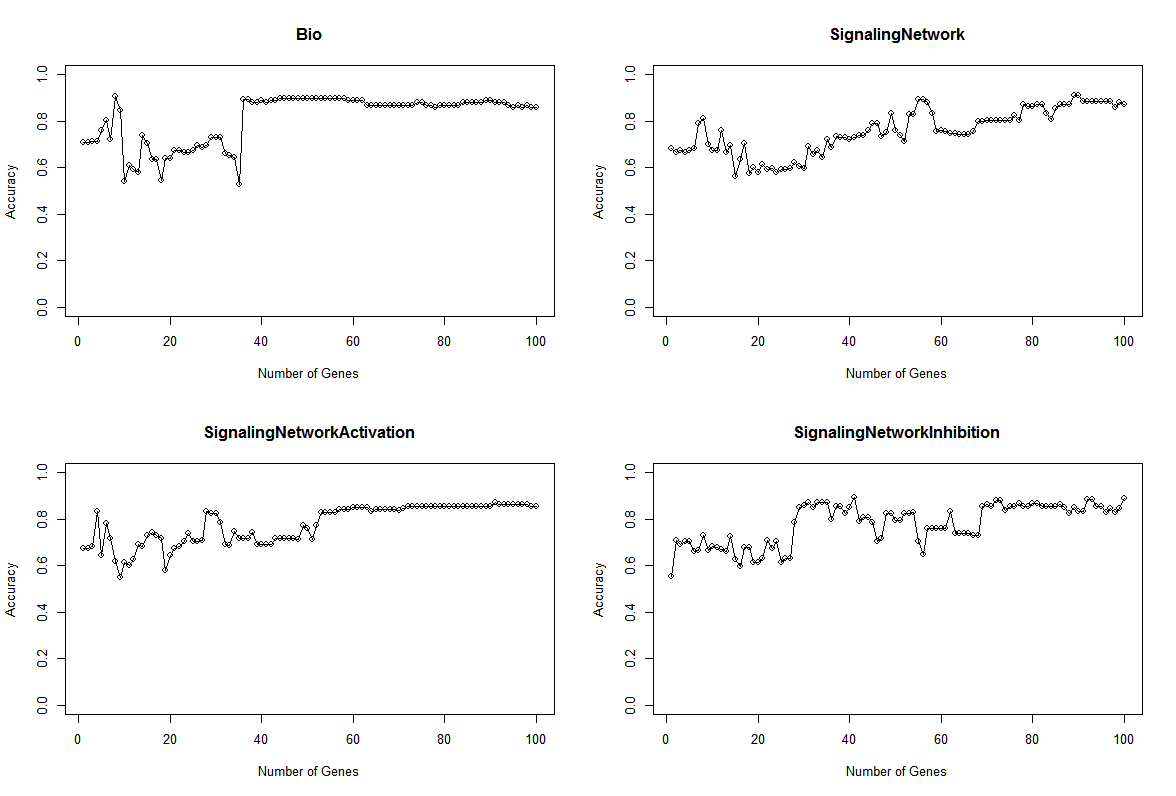


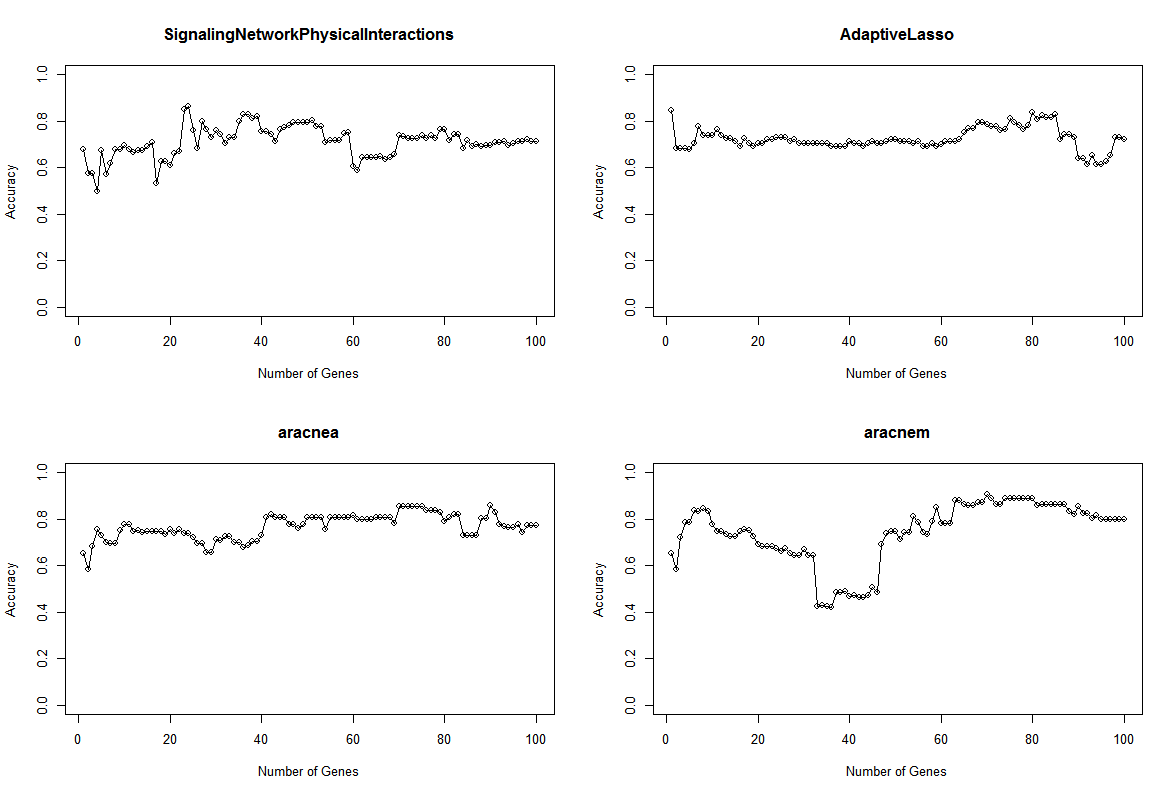


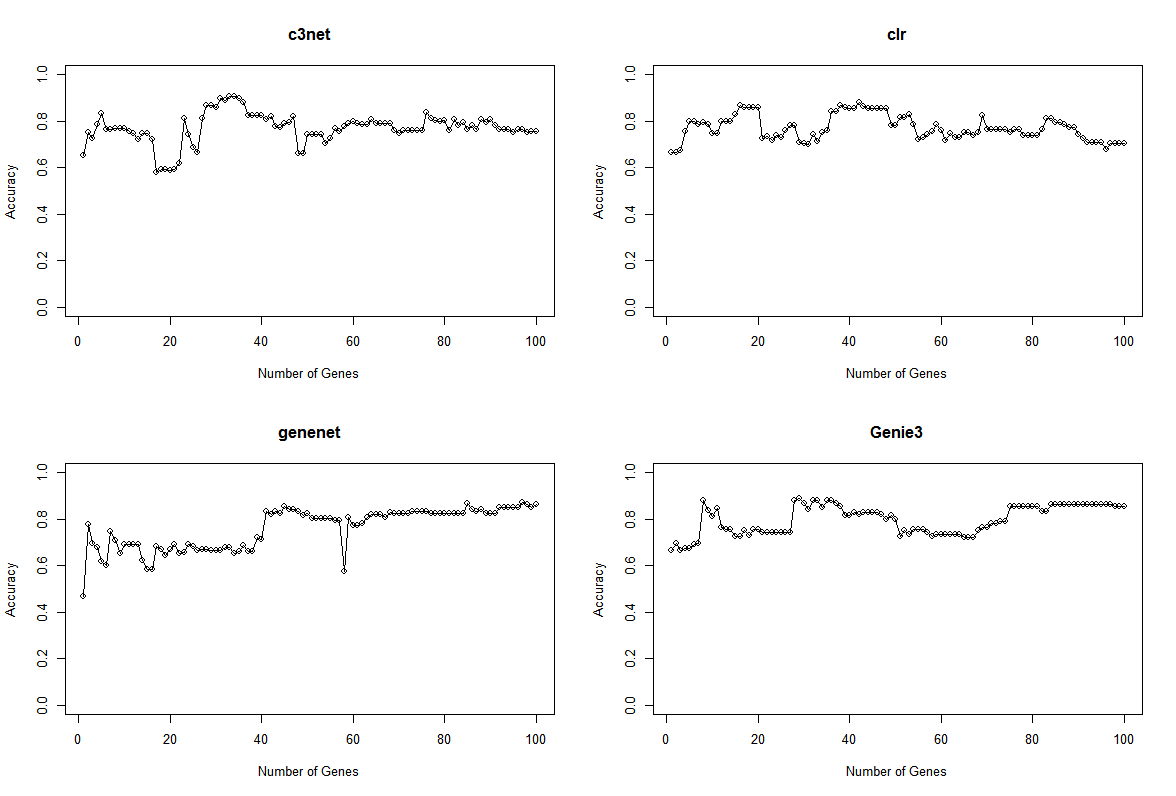


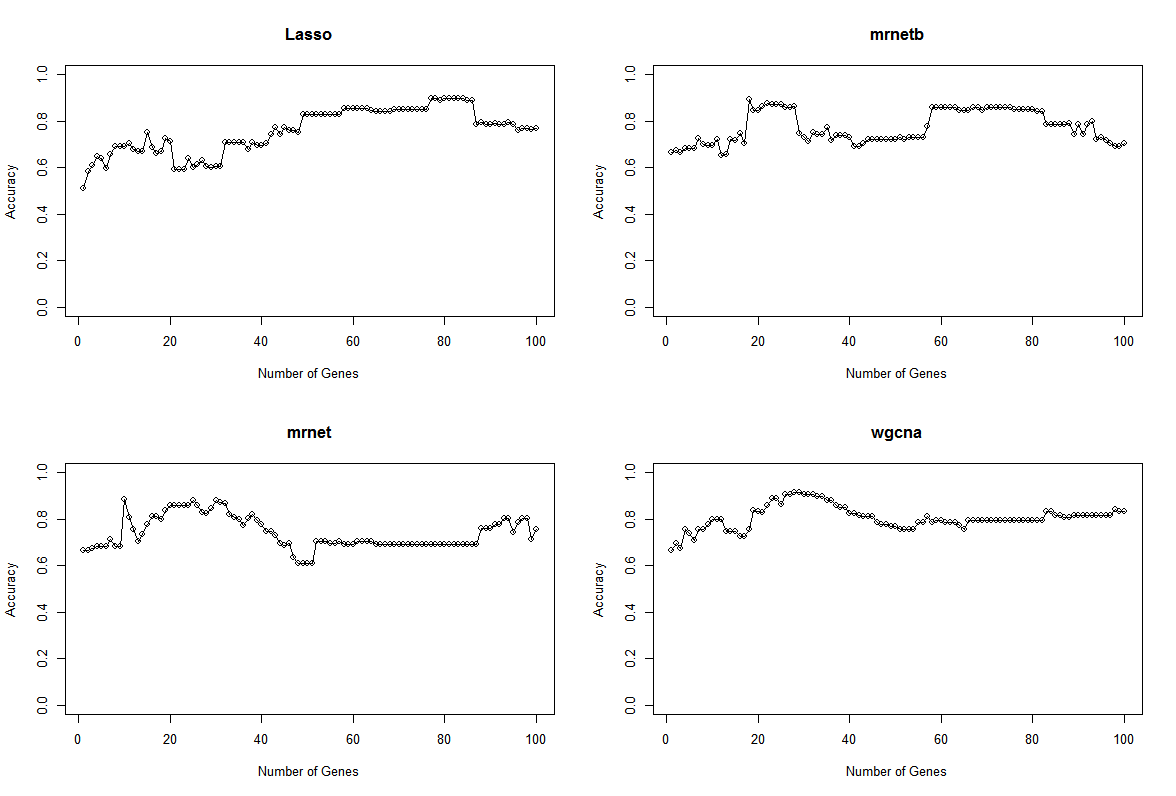


**Supplementary Figure 29:** Mean accuracy rates of the top 100 sequential genes from all ranked and re-ranked gene lists from each method in combination with PageRank reconciling method, using hold out validation with train set the TCGA expression values and test set the expression values from 4 GEO independent datasets for Luminal A subtype of breast cancer.


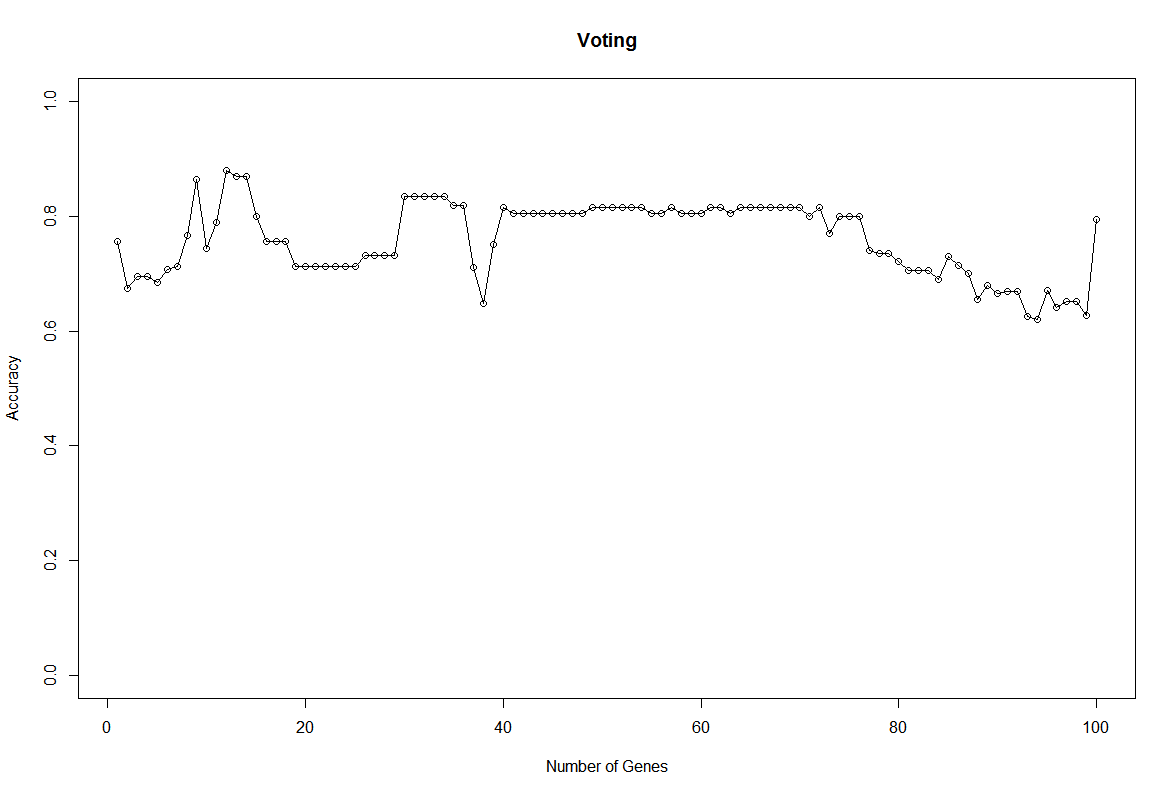


**Supplementary Figure 30:** Mean accuracy rate of the sequential gene selection from the top 100 re-ranked genes with the ensemble Voting method, using hold out validation with train set the TCGA expression values and test set the expression values from 4 GEO independent datasets for Luminal A subtype of breast cancer.


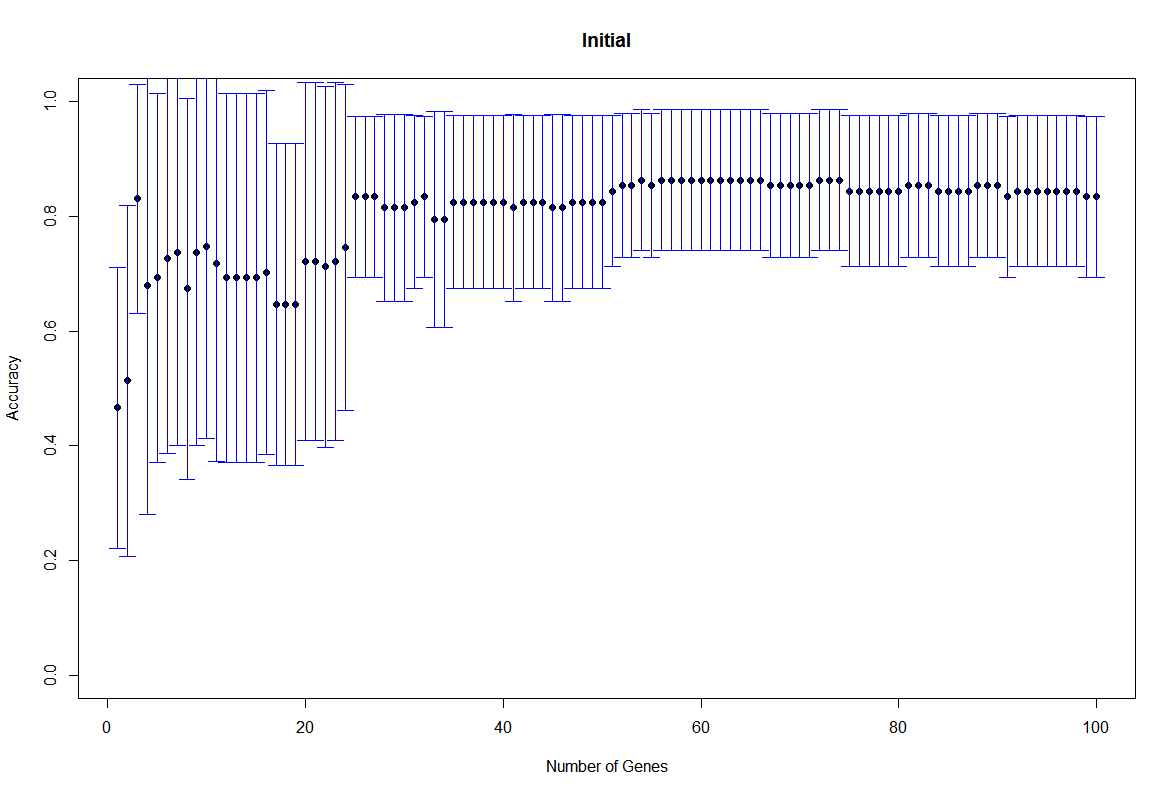


**Supplementary Figure 31:** Mean accuracy rate, with error bars, of the sequential gene selection from the top 100 Initial ranked genes (from Limma), using hold out validation with train set the TCGA expression values and test set the expression values from 4 GEO independent datasets for Luminal A subtype of breast cancer.


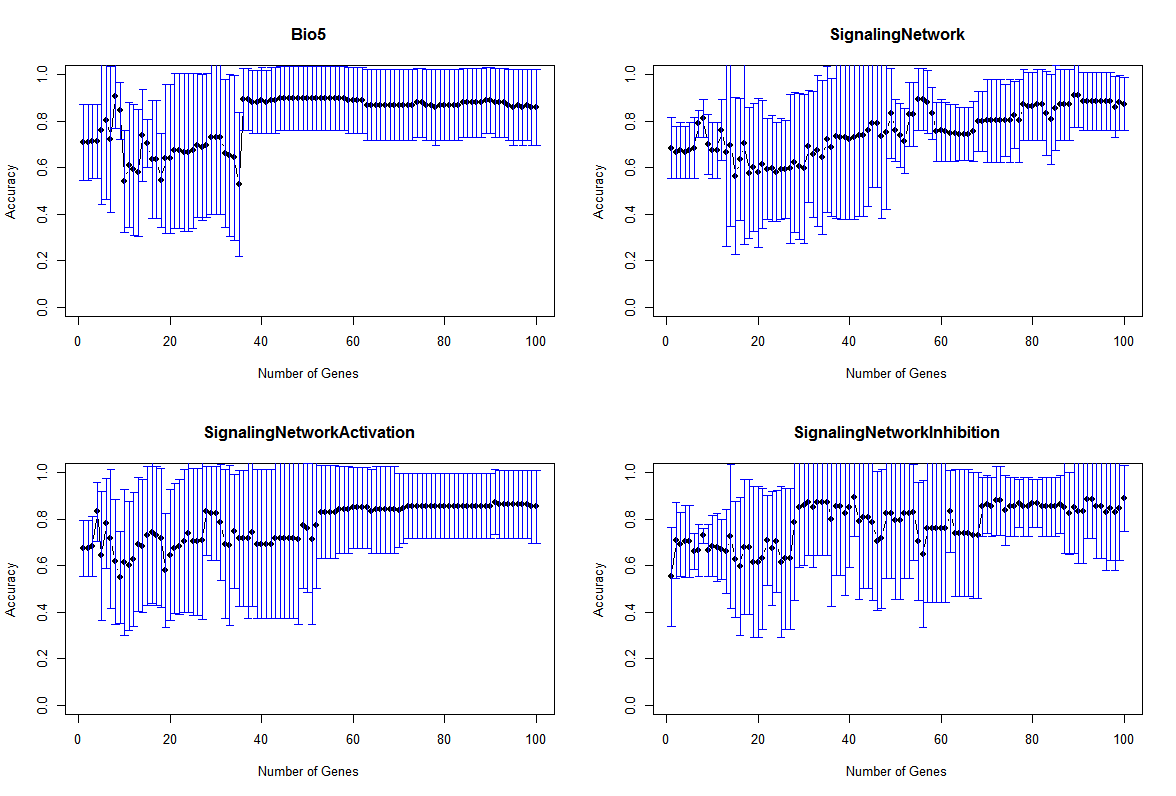


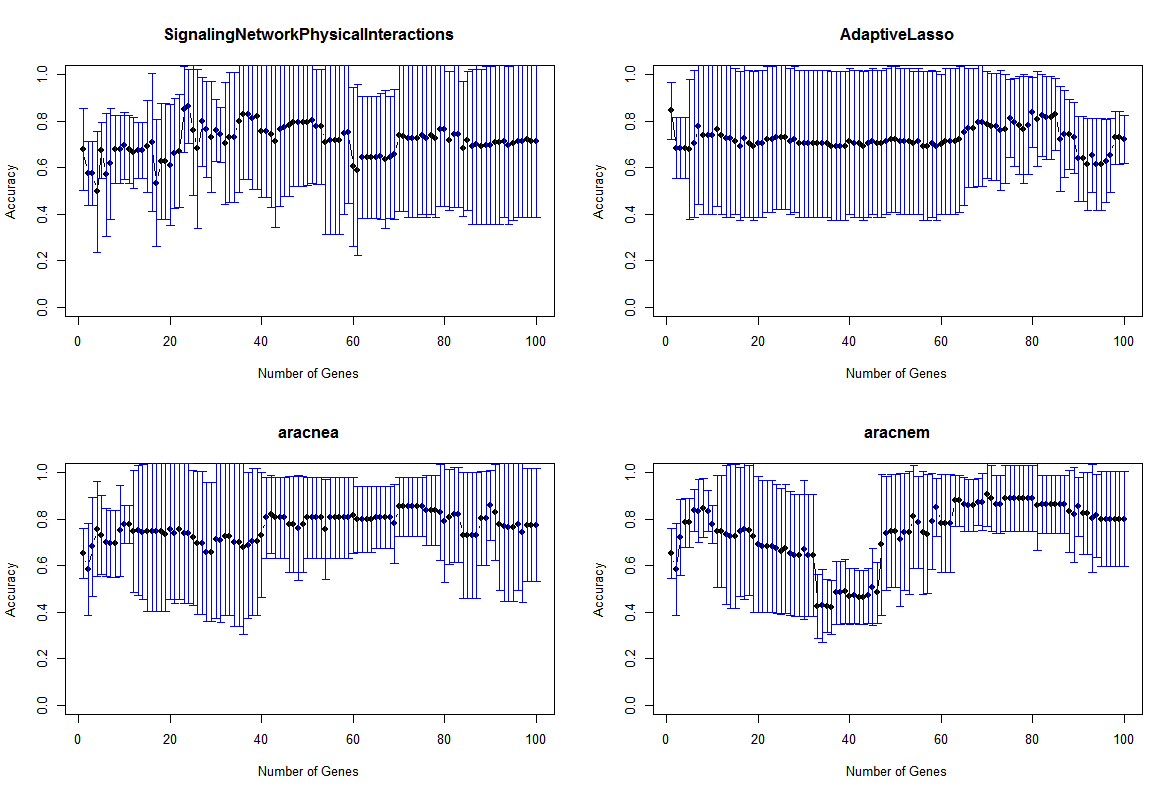


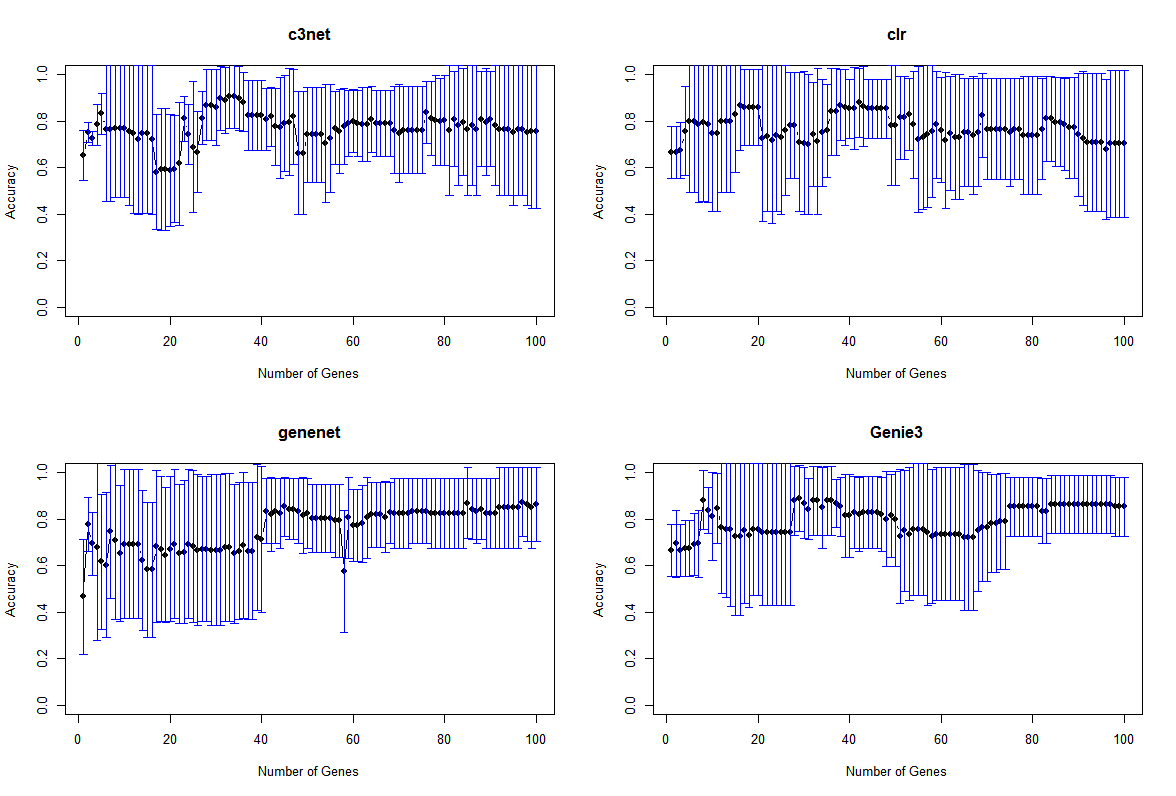


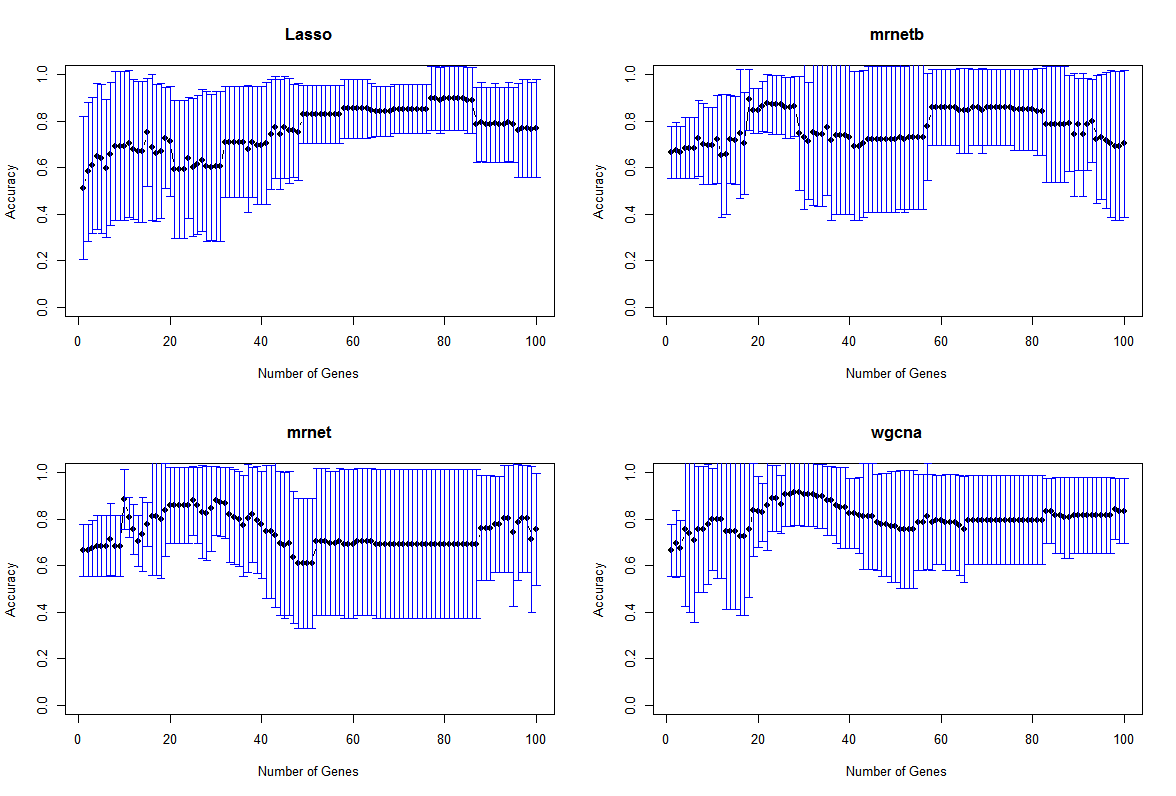


**Supplementary Figure 32:** Mean accuracy rates, with error bars, of the top 100 sequential genes from all ranked and re-ranked gene lists from each method in combination with PageRank reconciling method, using hold out validation with train set the TCGA expression values and test set the expression values from 4 GEO independent datasets Luminal A subtype of breast cancer.


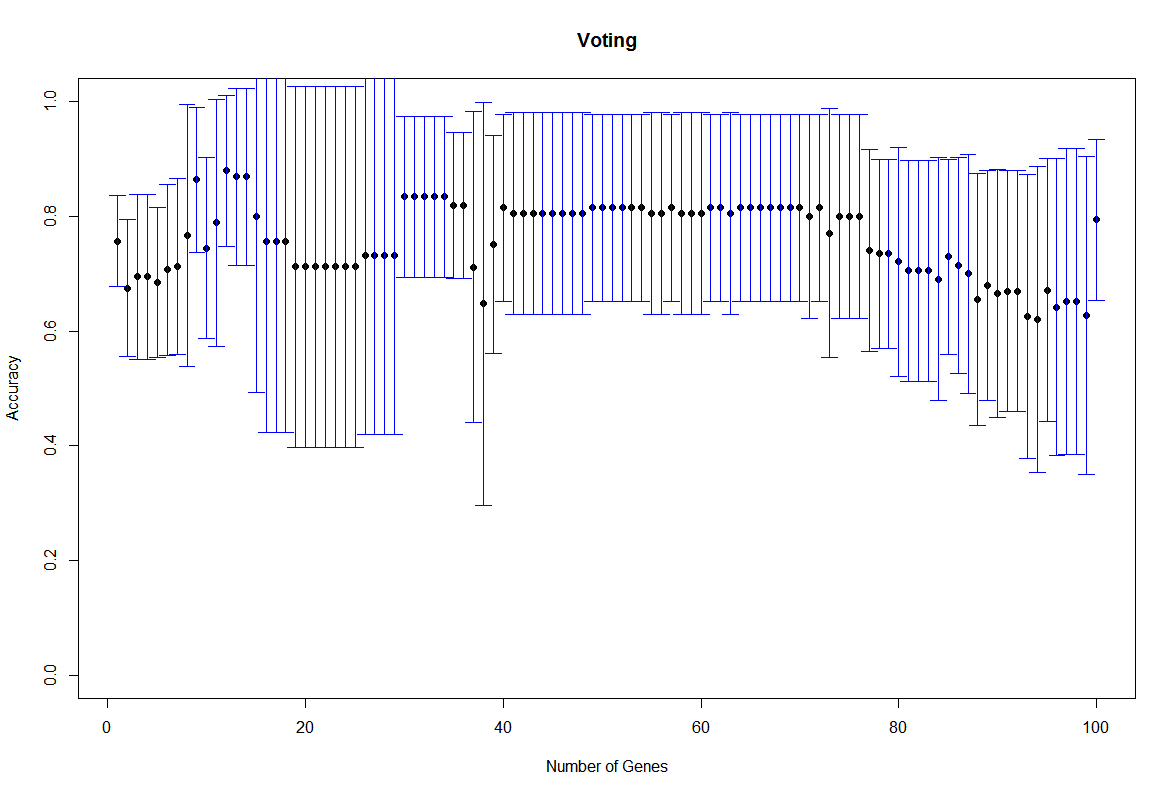


**Supplementary Figure 33:** Mean accuracy rate, with error bars, of the sequential gene selection the top 100 re-ranked genes with the ensemble Voting method, using hold out validation with train set the TCGA expression values and test set the expression values from 4 GEO independent datasets for Luminal A subtype of breast cancer.

**
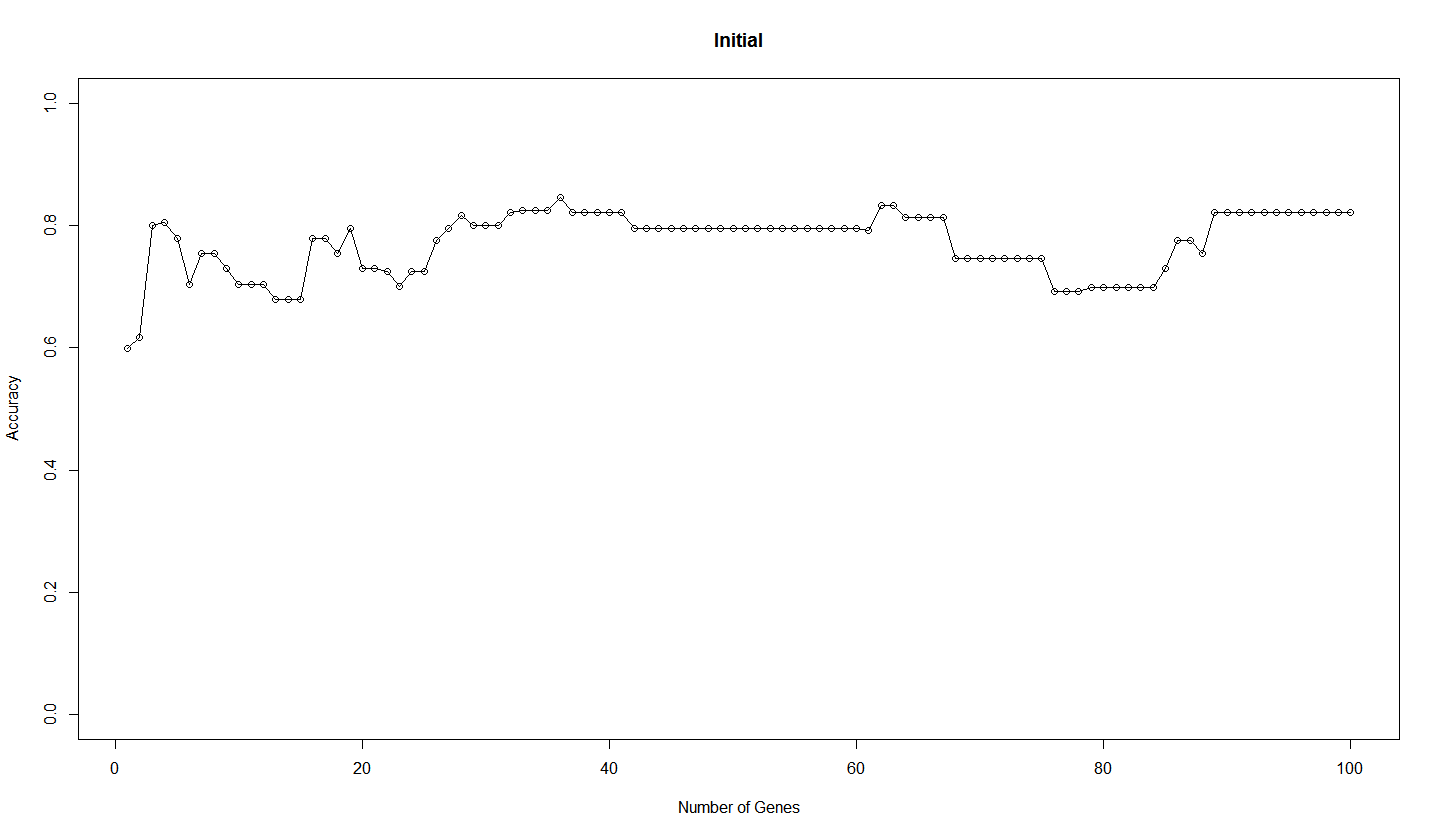
**

**Supplementary Figure 34:** Mean accuracy rate of the sequential gene selection from the top 100 re-ranked genes with the ensemble Voting method, using hold out validation with train set the TCGA expression values and test set the expression values from 4 GEO independent datasets for Luminal B subtype of breast cancer.

**
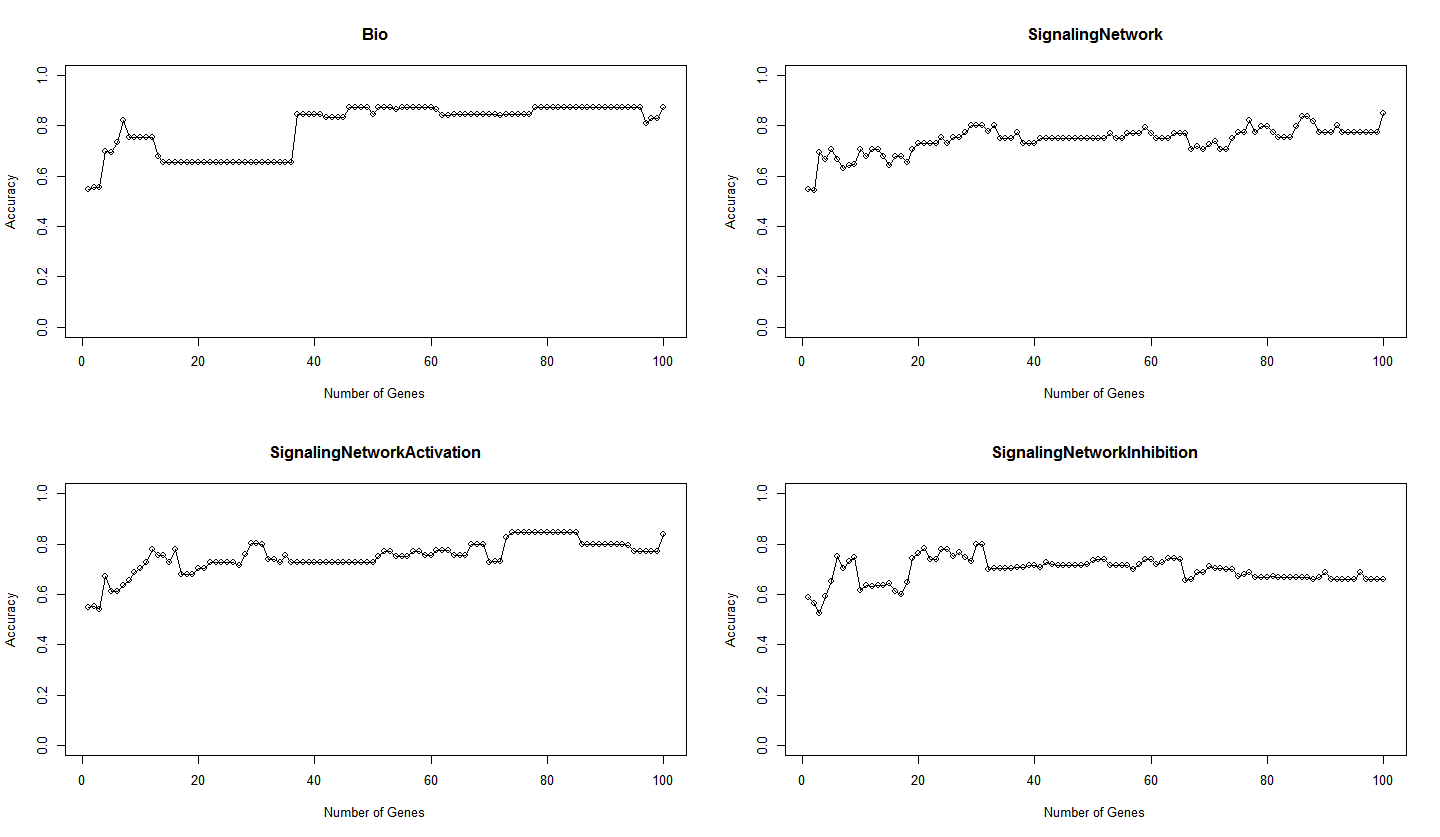
**

**
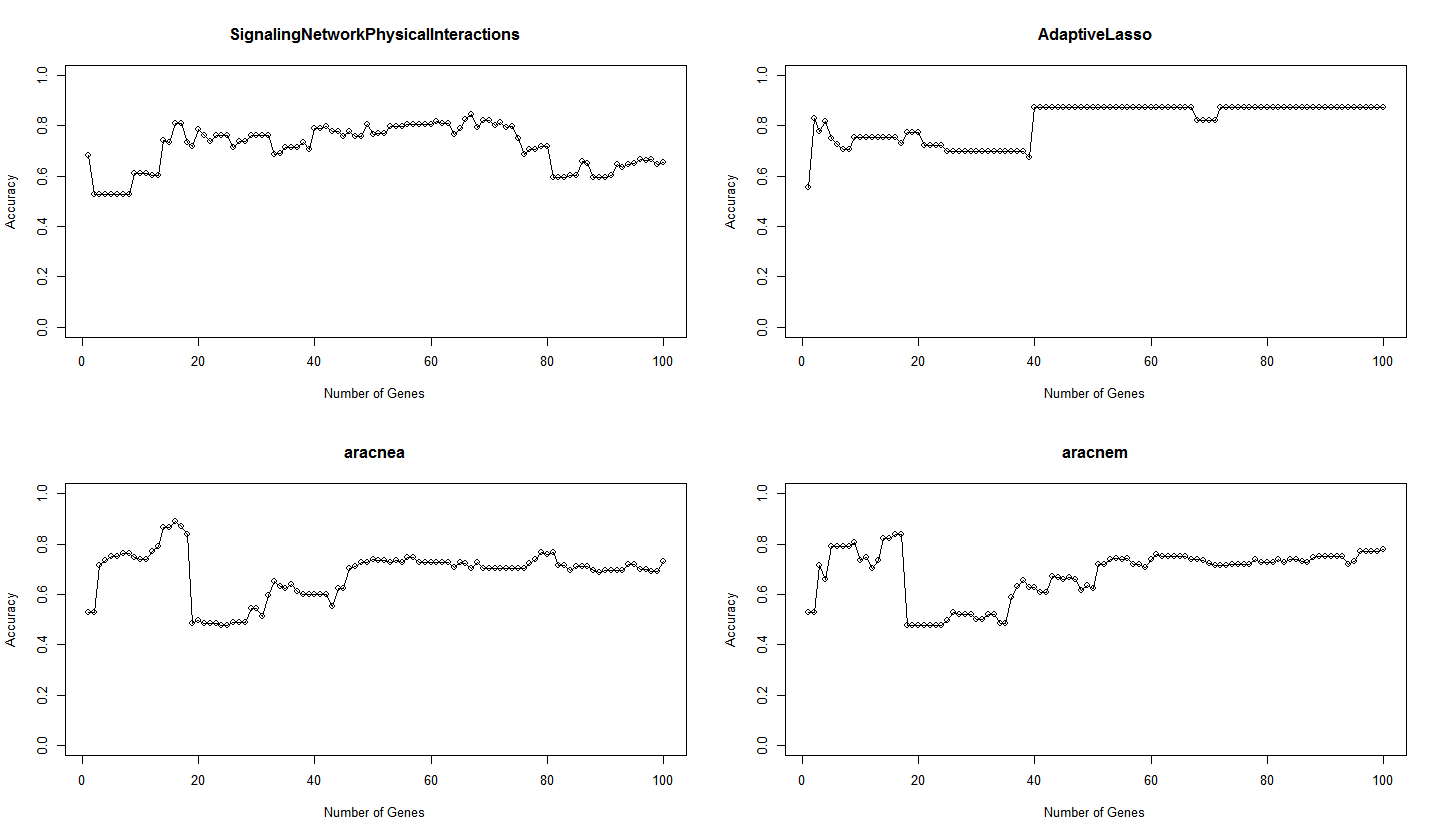
**

**
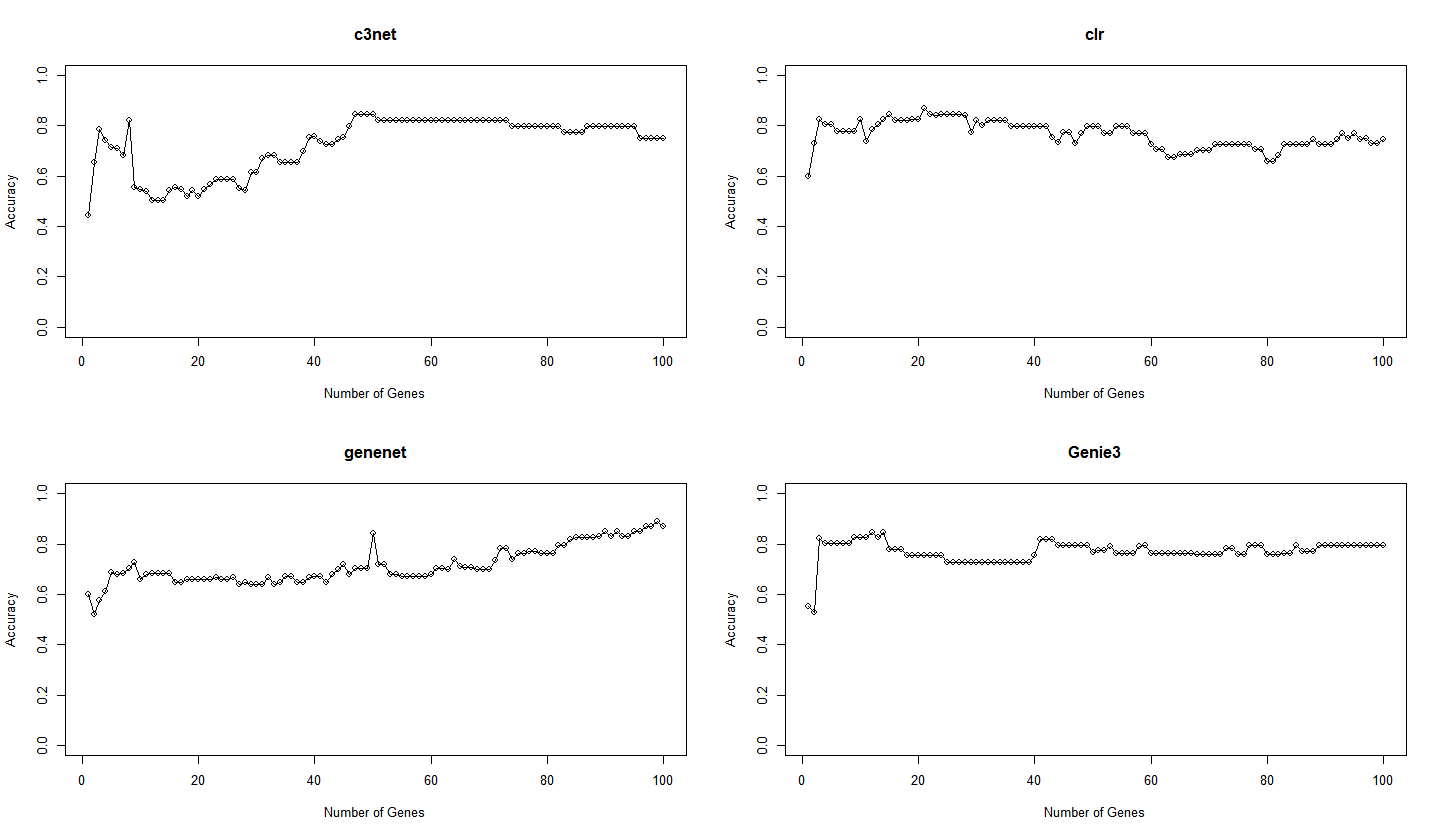
**

**
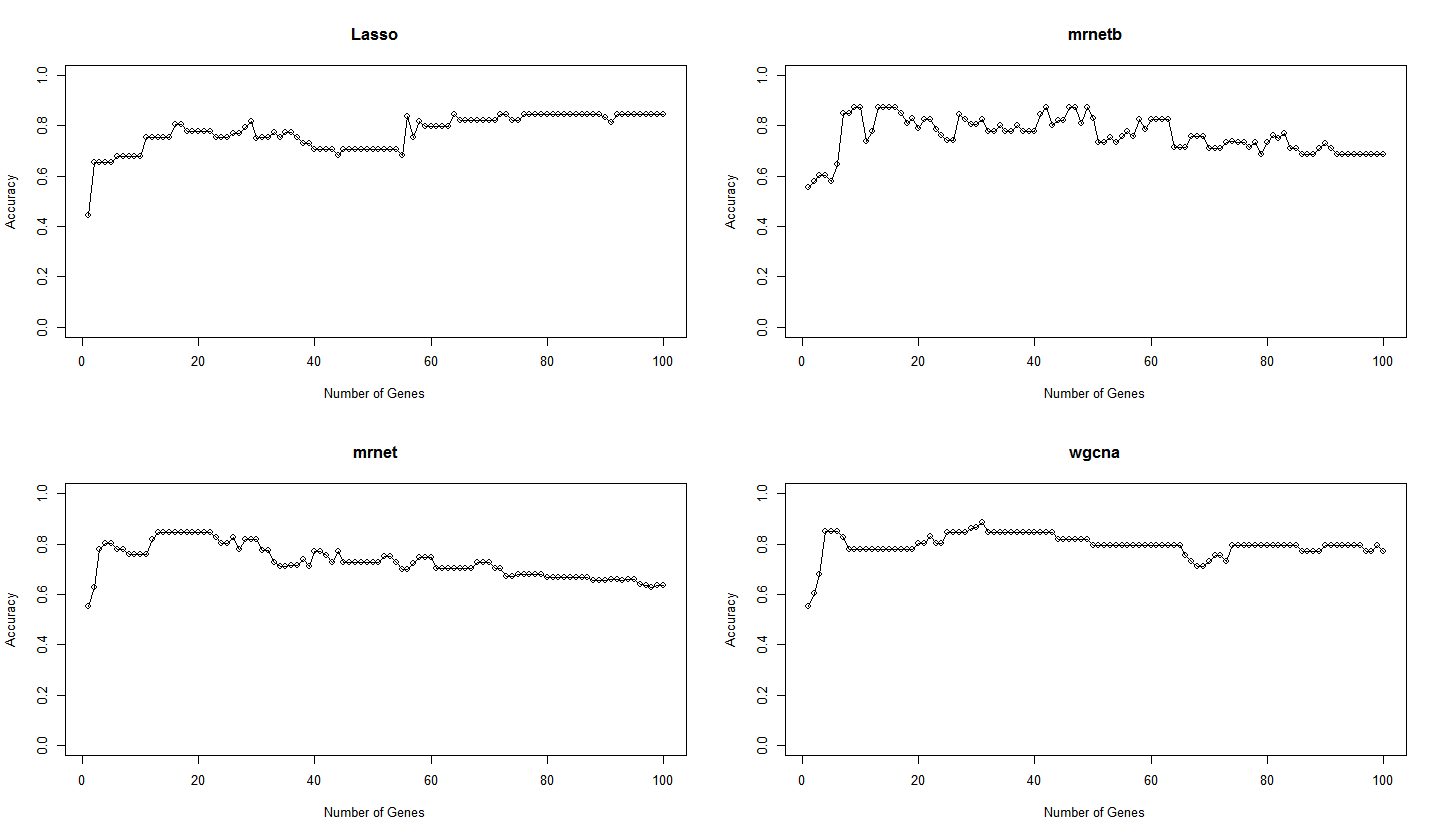
**

**Supplementary Figure 35:** Mean accuracy rates of the top 100 sequential genes from all ranked and re-ranked gene lists from each method in combination with PageRank reconciling method, using hold out validation with train set the TCGA expression values and test set the expression values from 4 GEO independent datasets for Luminal B subtype of breast cancer.

**
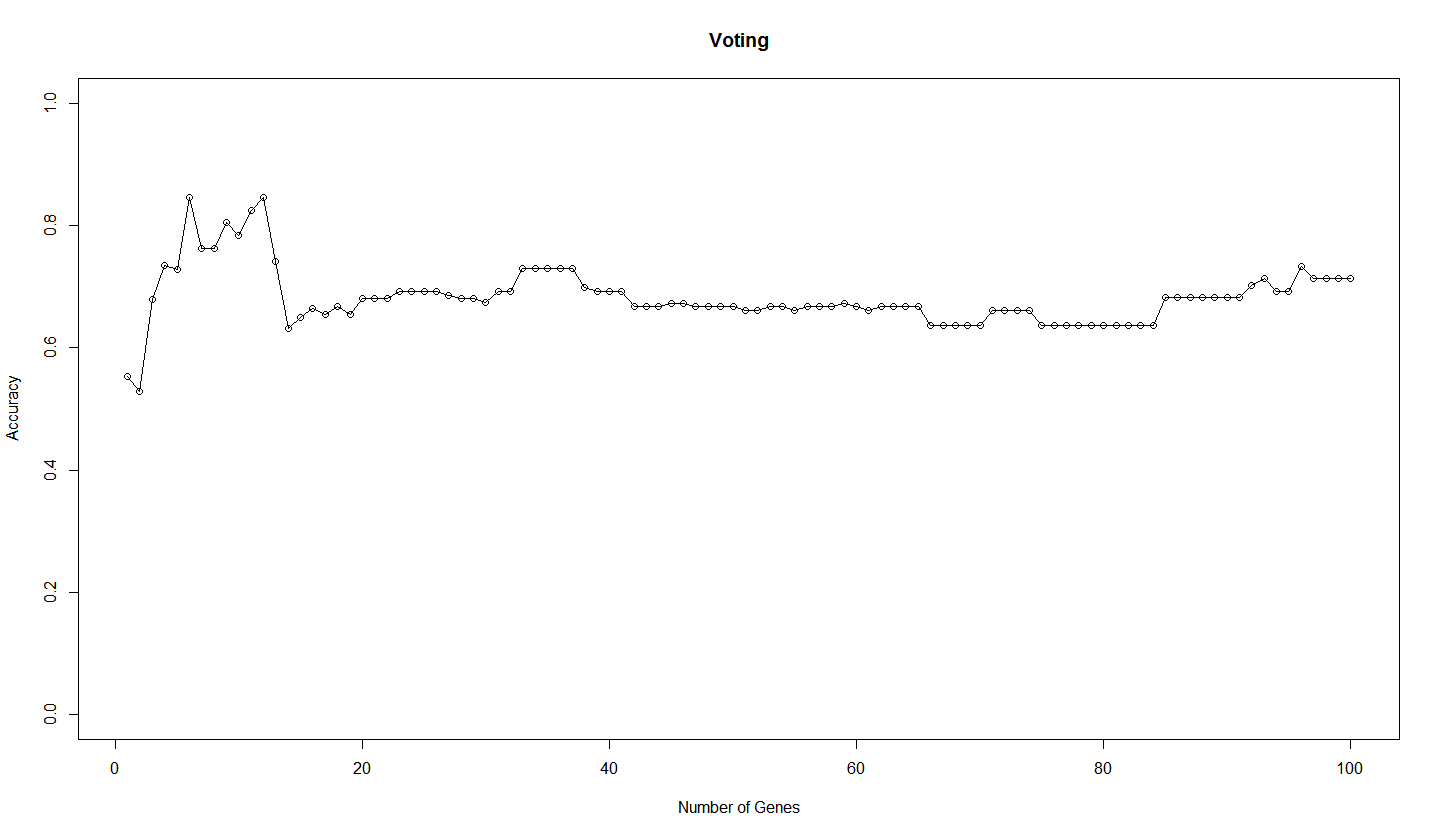
**

**Supplementary Figure 36:** Mean accuracy rate of the sequential gene selection from the top 100 re-ranked genes with the ensemble Voting method, using hold out validation with train set the TCGA expression values and test set the expression values from 4 GEO independent datasets for Luminal B subtype of breast cancer.

**
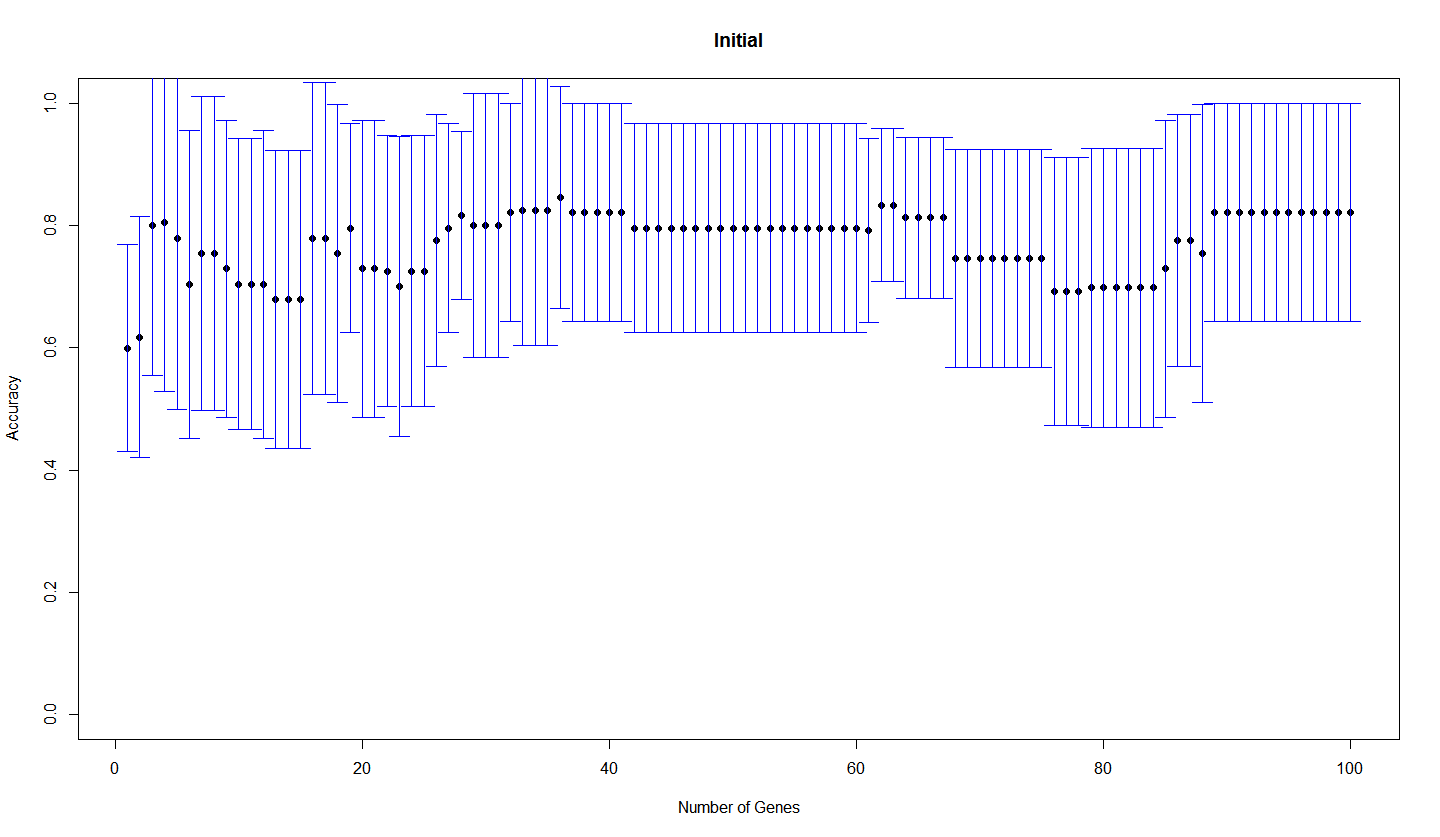
**

**Supplementary Figure 37:** Mean accuracy rate, with error bars, of the sequential gene selection from the top 100 Initial ranked genes (from Limma), using hold out validation with train set the TCGA expression values and test set the expression values from 4 GEO independent datasets for Luminal B subtype of breast cancer.

**
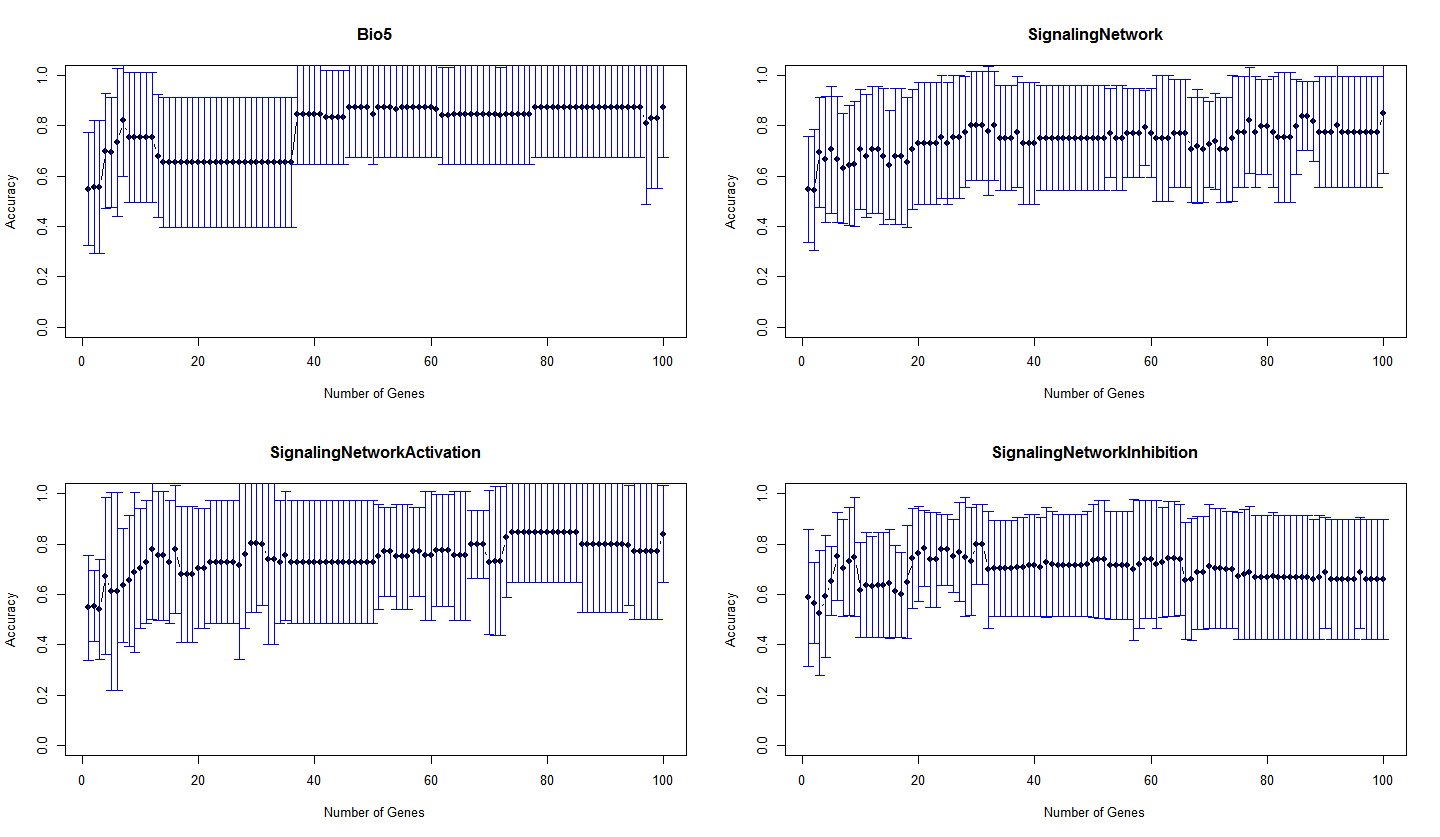
**

**
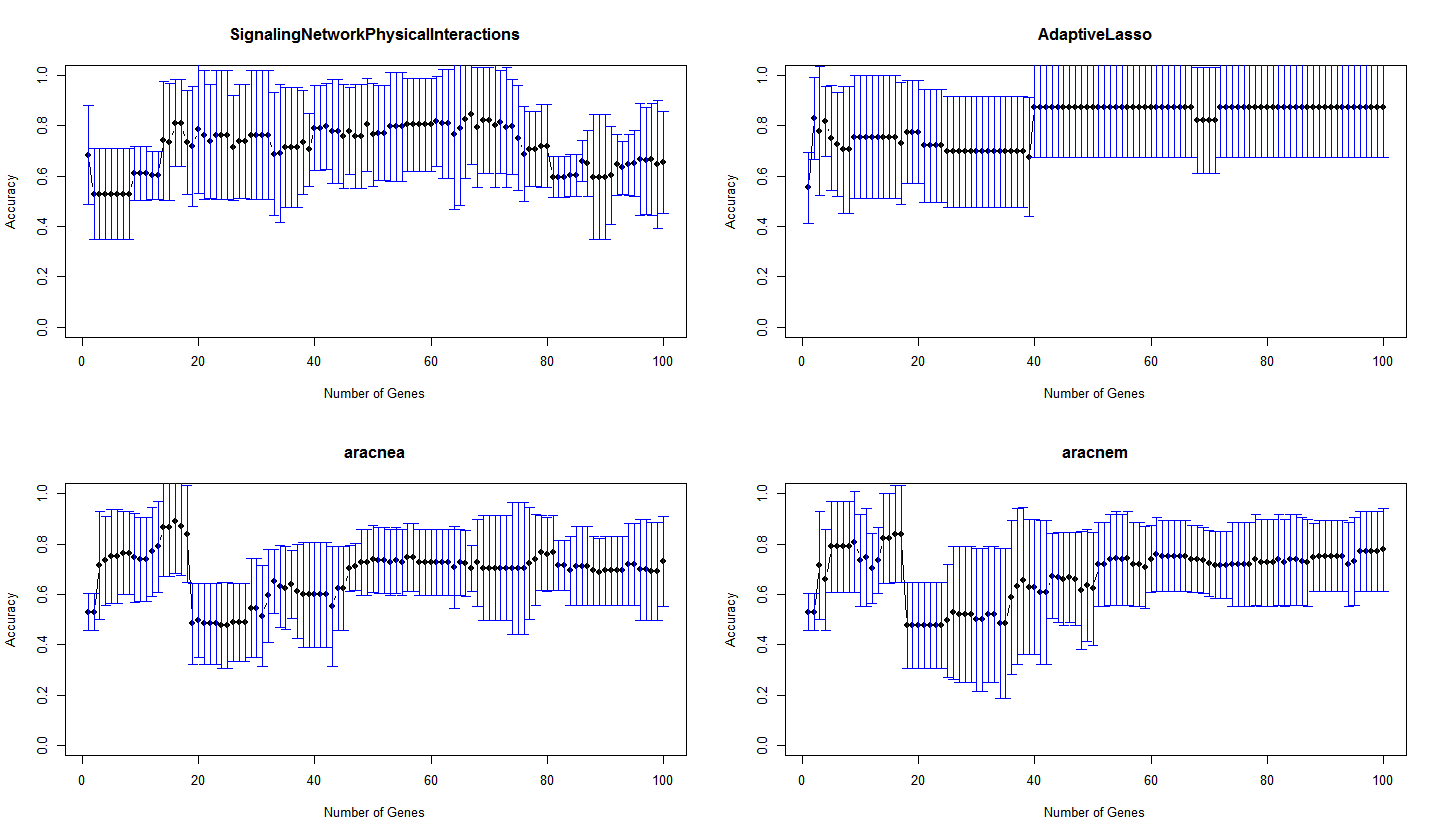
**

**
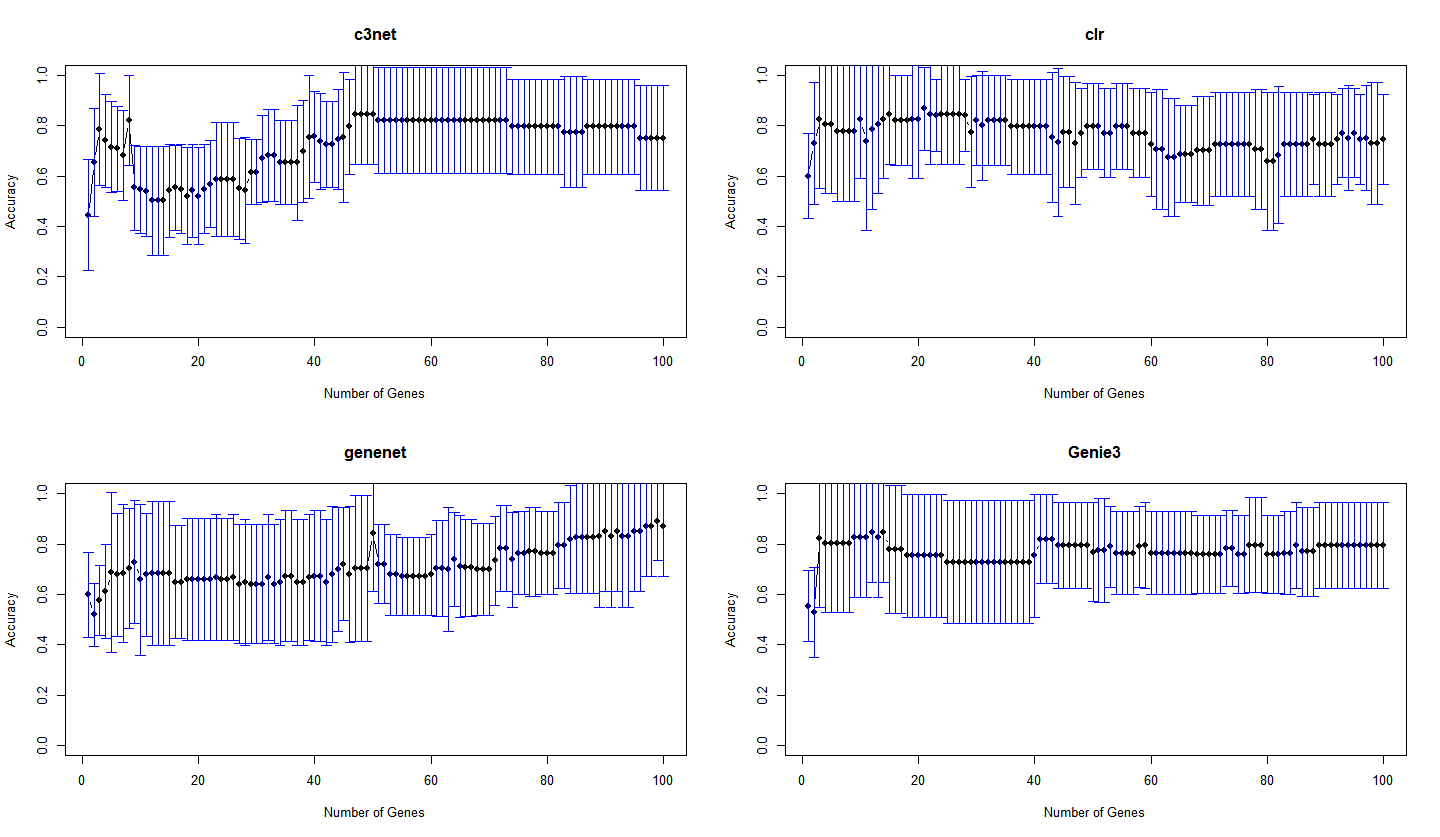
**

**
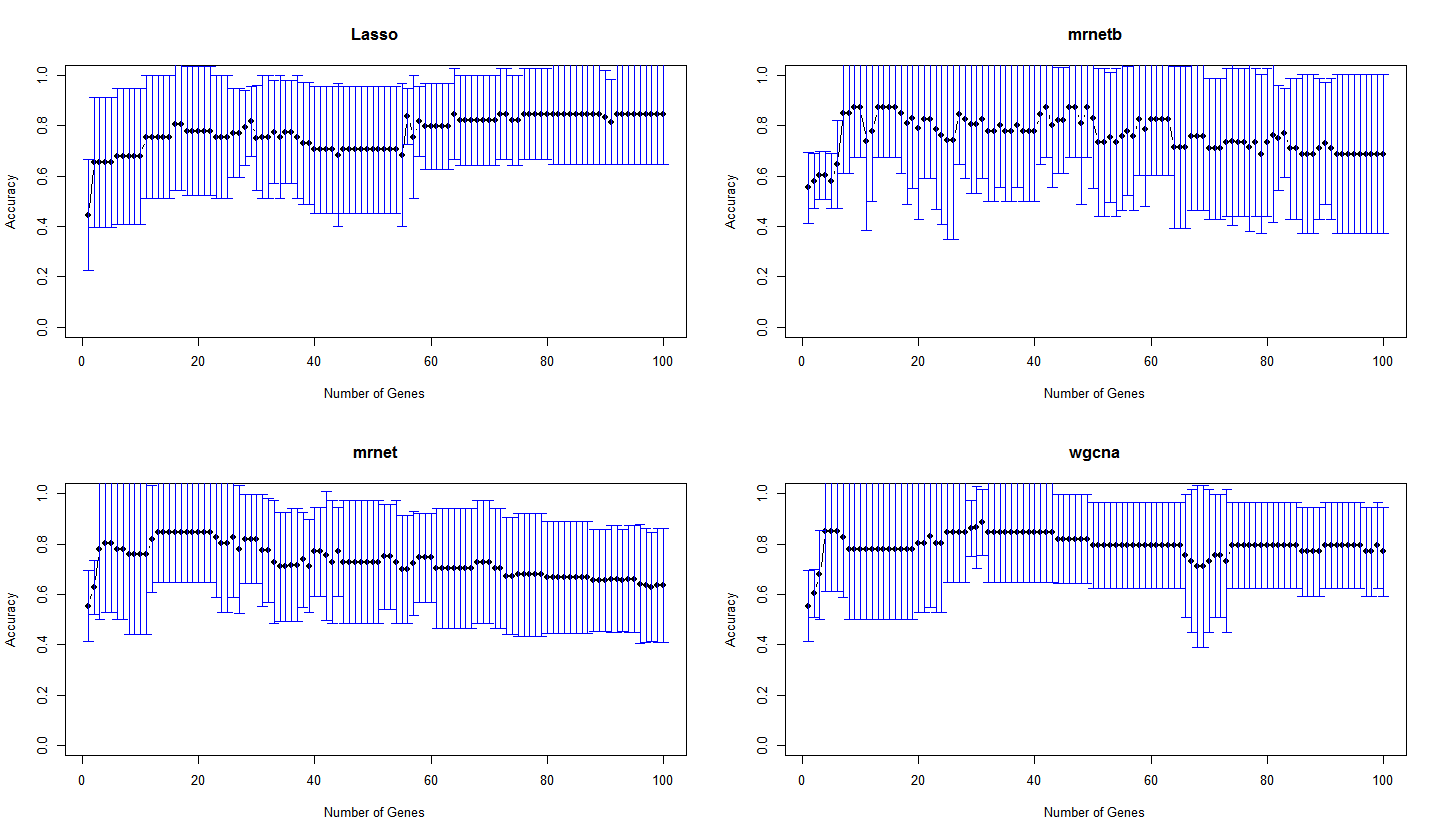
**

**Supplementary Figure 38:** Mean accuracy rates, with error bars, of the top 100 sequential genes from all ranked and re-ranked gene lists from each method in combination with PageRank reconciling method, using hold out validation with train set the TCGA expression values and test set the expression values from 4 GEO independent datasets Luminal B subtype of breast cancer.

**
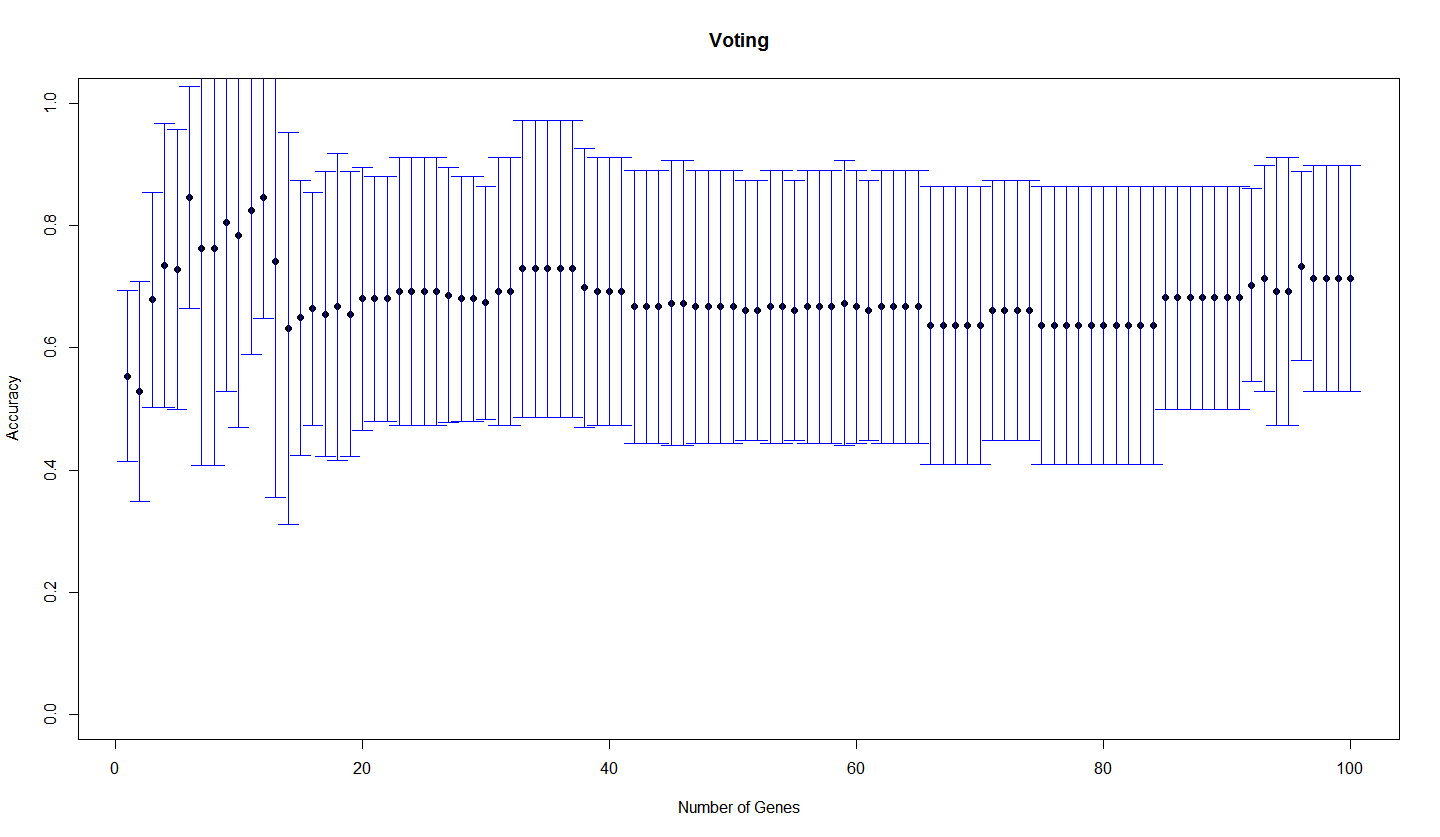
**

**Supplementary Figure 39:** Mean accuracy rate, with error bars, of the sequential gene selection the top 100 re-ranked genes with the ensemble Voting method, using hold out validation with train set the TCGA expression values and test set the expression values from 4 GEO independent datasets for Luminal B subtype of breast cancer.

**
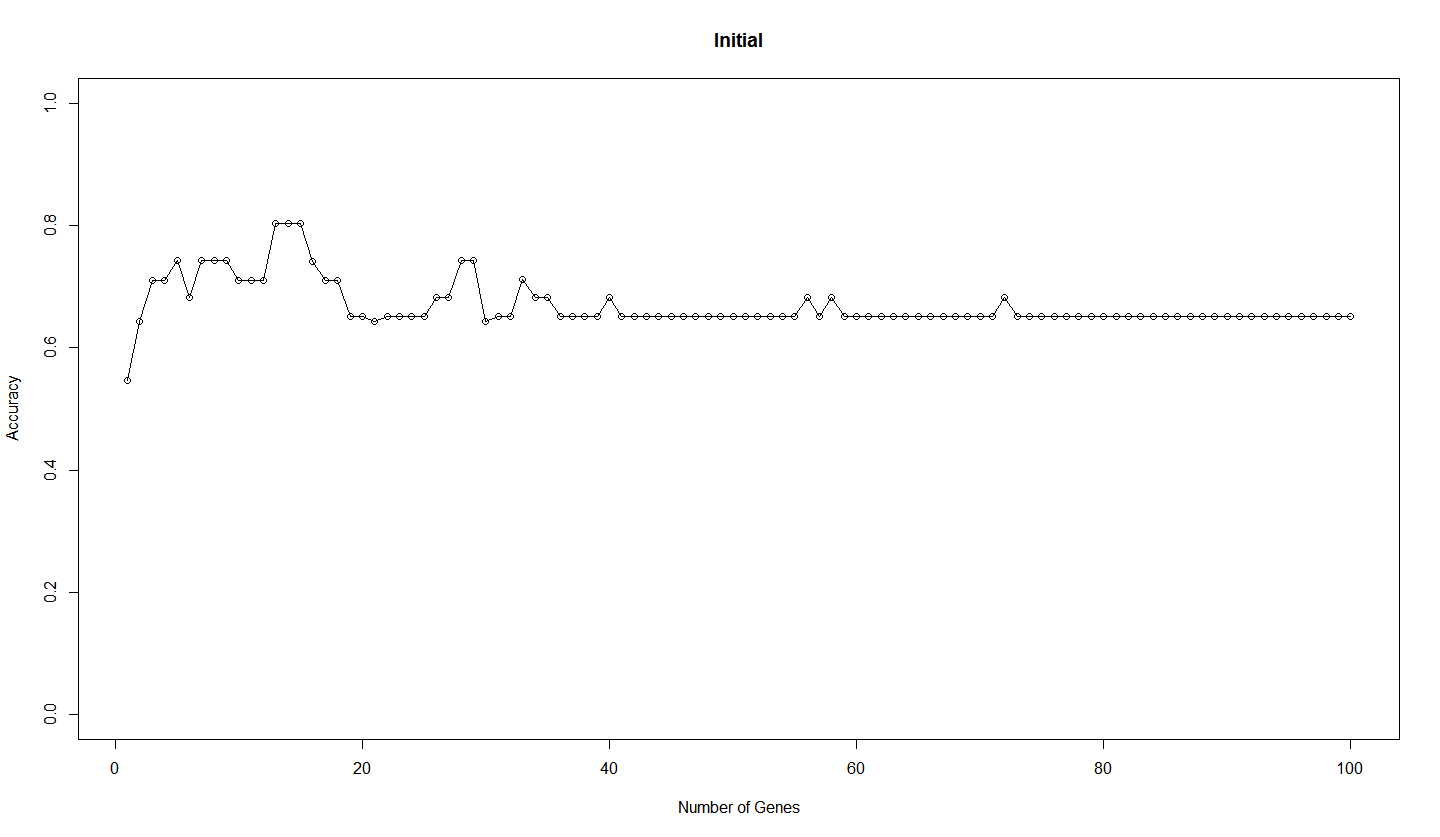
**

**Supplementary Figure 40:** Mean accuracy rate of the sequential gene selection from the top 100 re-ranked genes with the ensemble Voting method, using hold out validation with train set the TCGA expression values and test set the expression values from 3 GEO independent datasets for HER2 subtype of breast cancer.

**
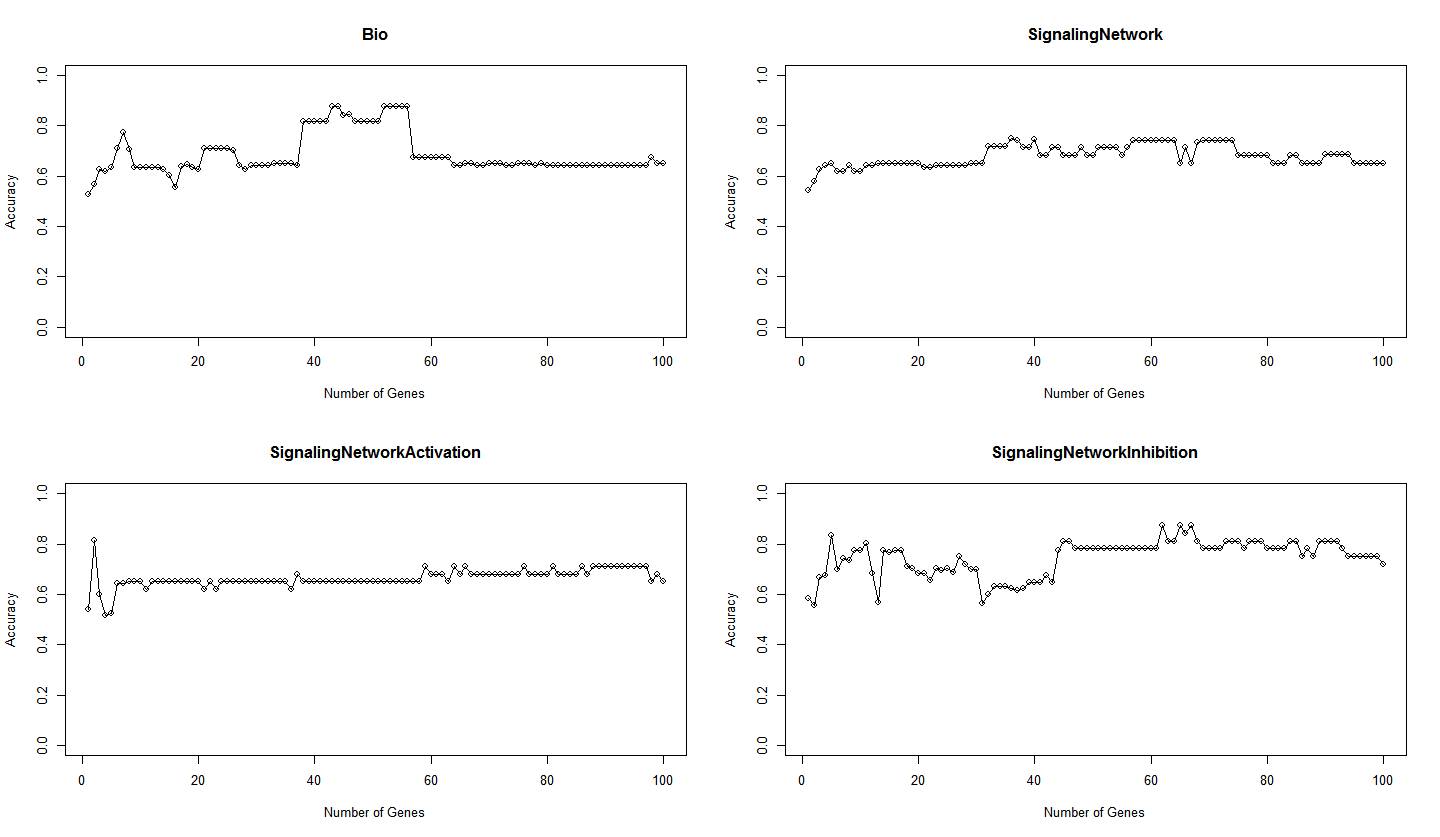
**

**
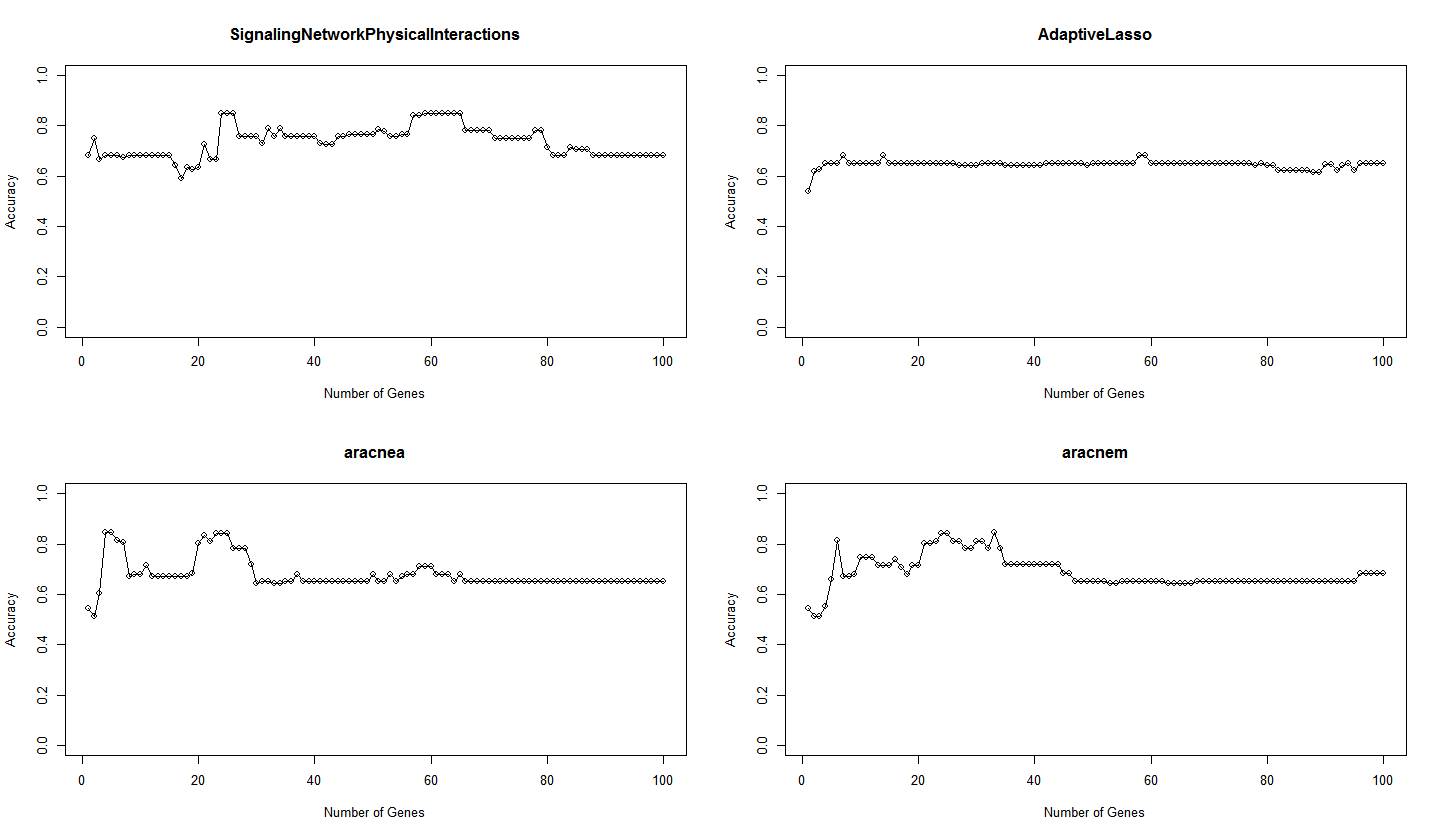
**

**
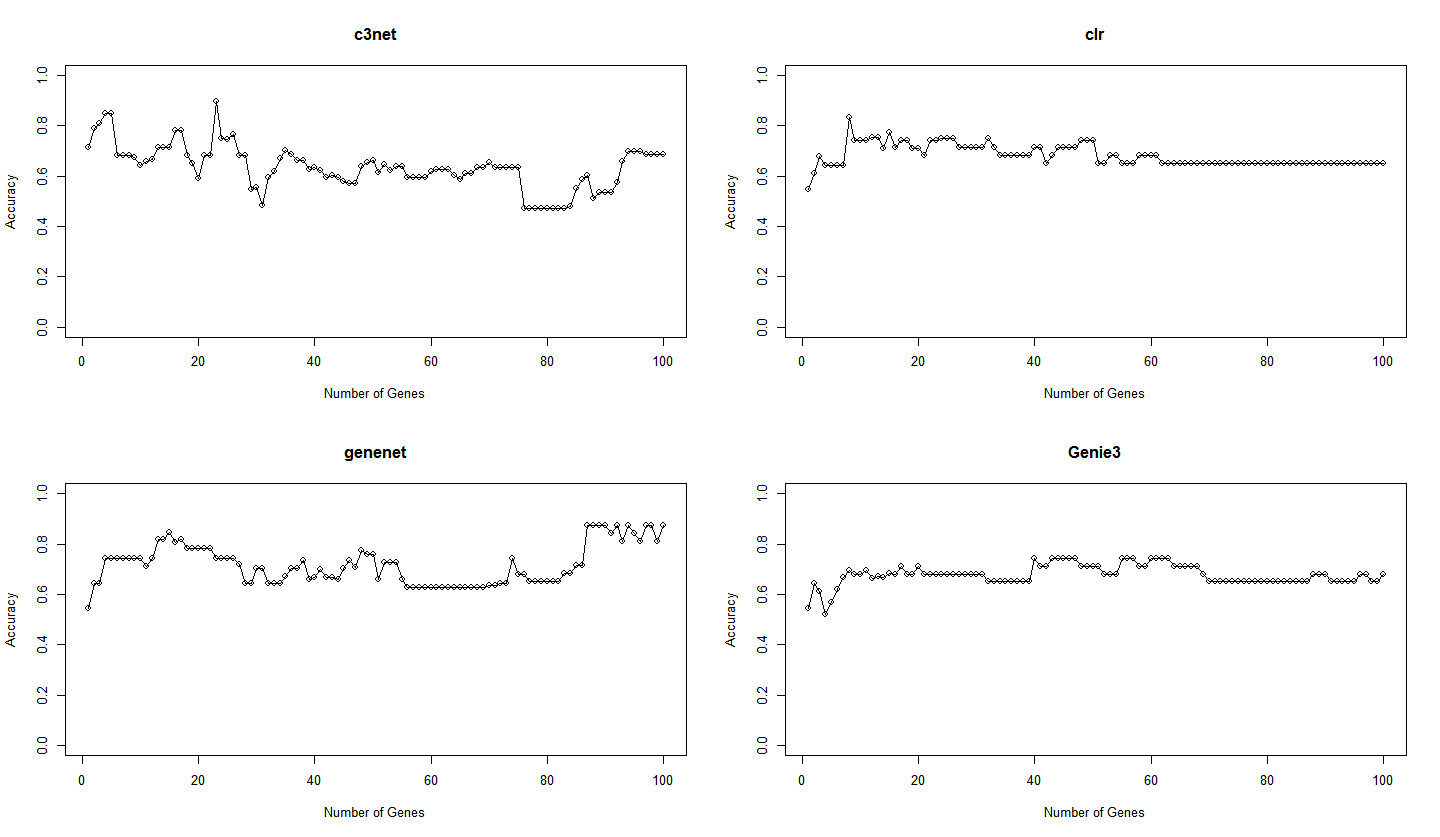
**

**
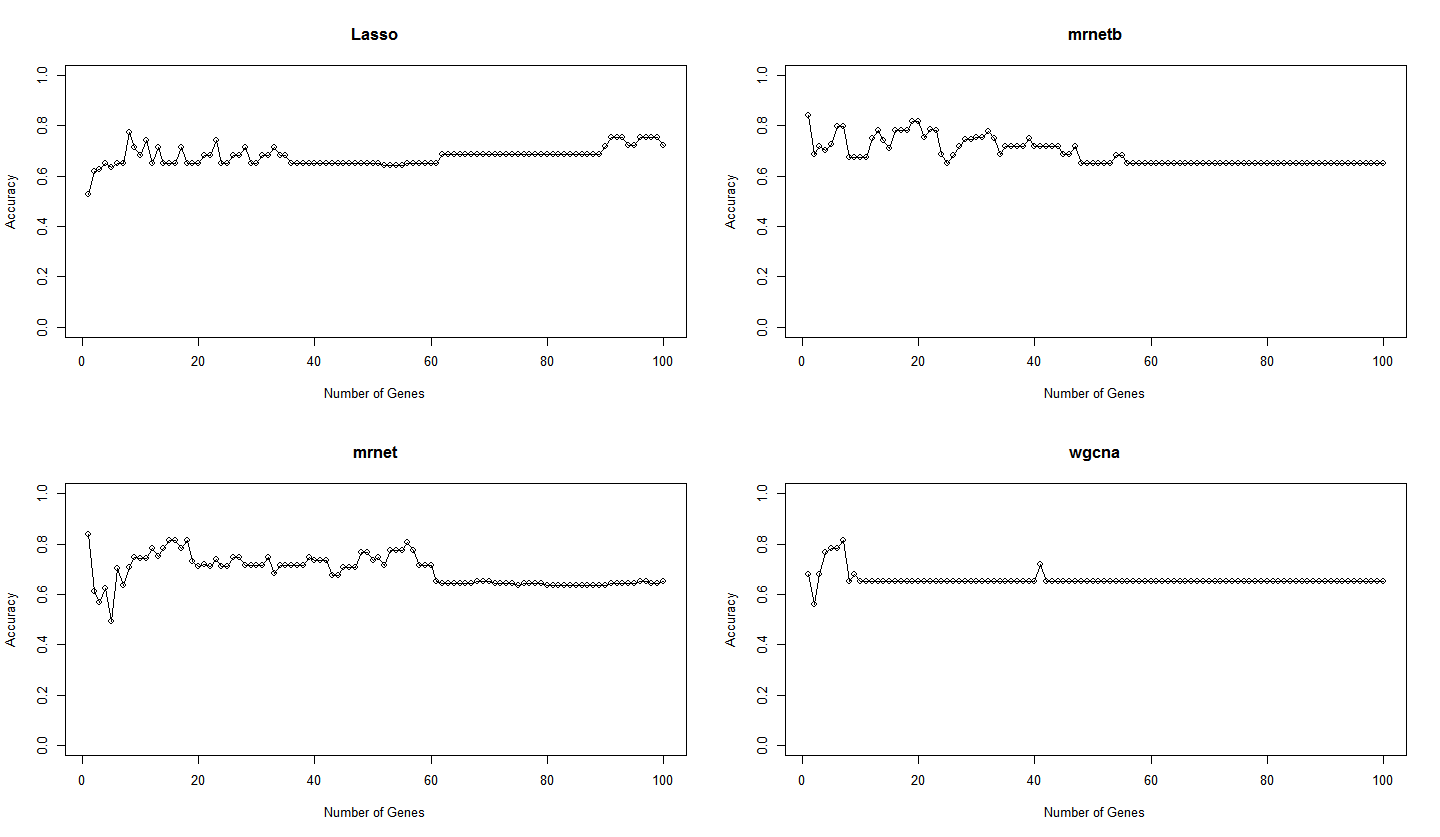
**

**Supplementary Figure 41:** Mean accuracy rates of the top 100 sequential genes from all ranked and re-ranked gene lists from each method in combination with PageRank reconciling method, using hold out validation with train set the TCGA expression values and test set the expression values from 3 GEO independent datasets for HER2 subtype of breast cancer.

**
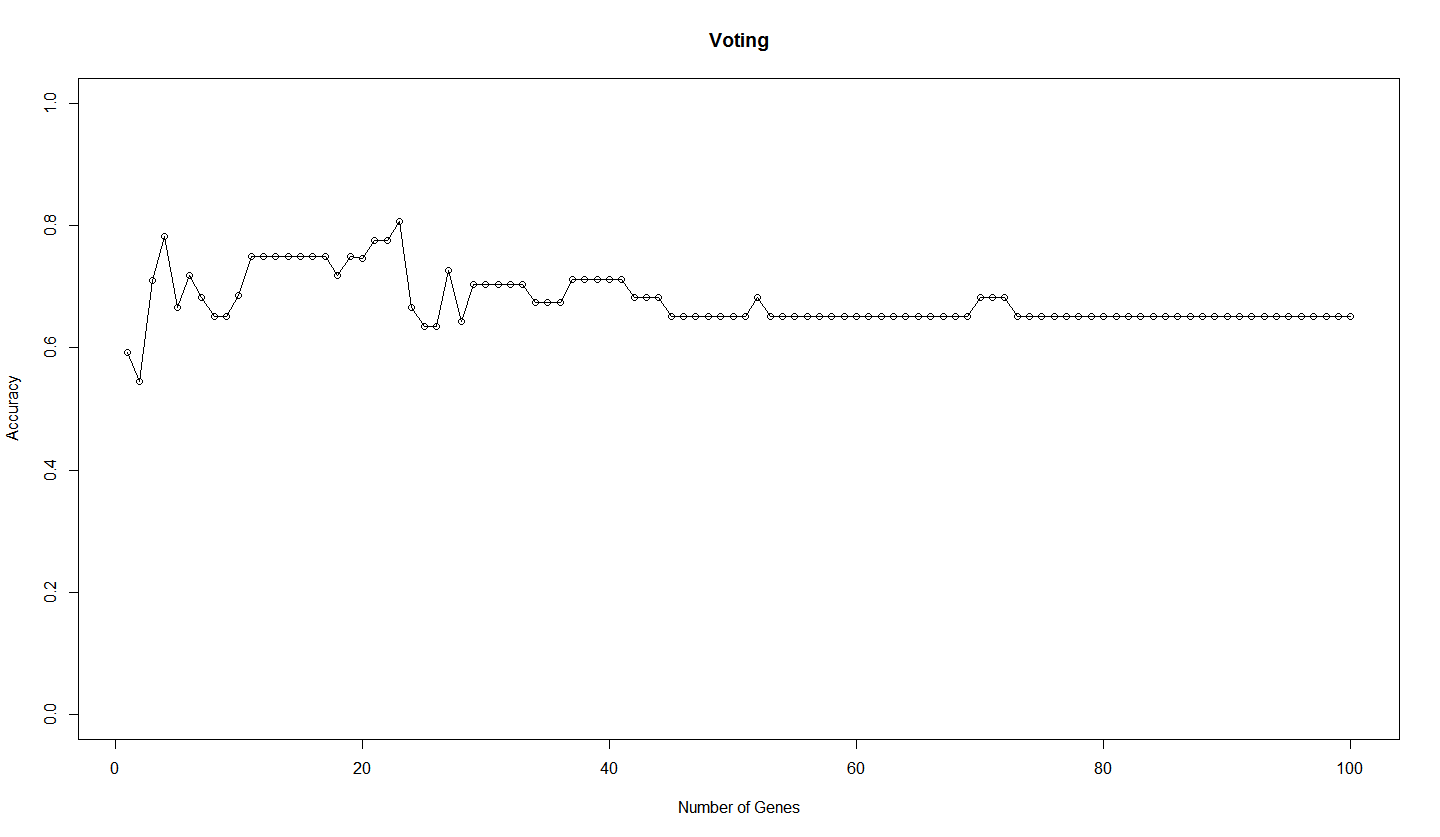
**

**Supplementary Figure 42:** Mean accuracy rate of the sequential gene selection from the top 100 re-ranked genes with the ensemble Voting method, using hold out validation with train set the TCGA expression values and test set the expression values from 3 GEO independent datasets for HER2 subtype of breast cancer.

**
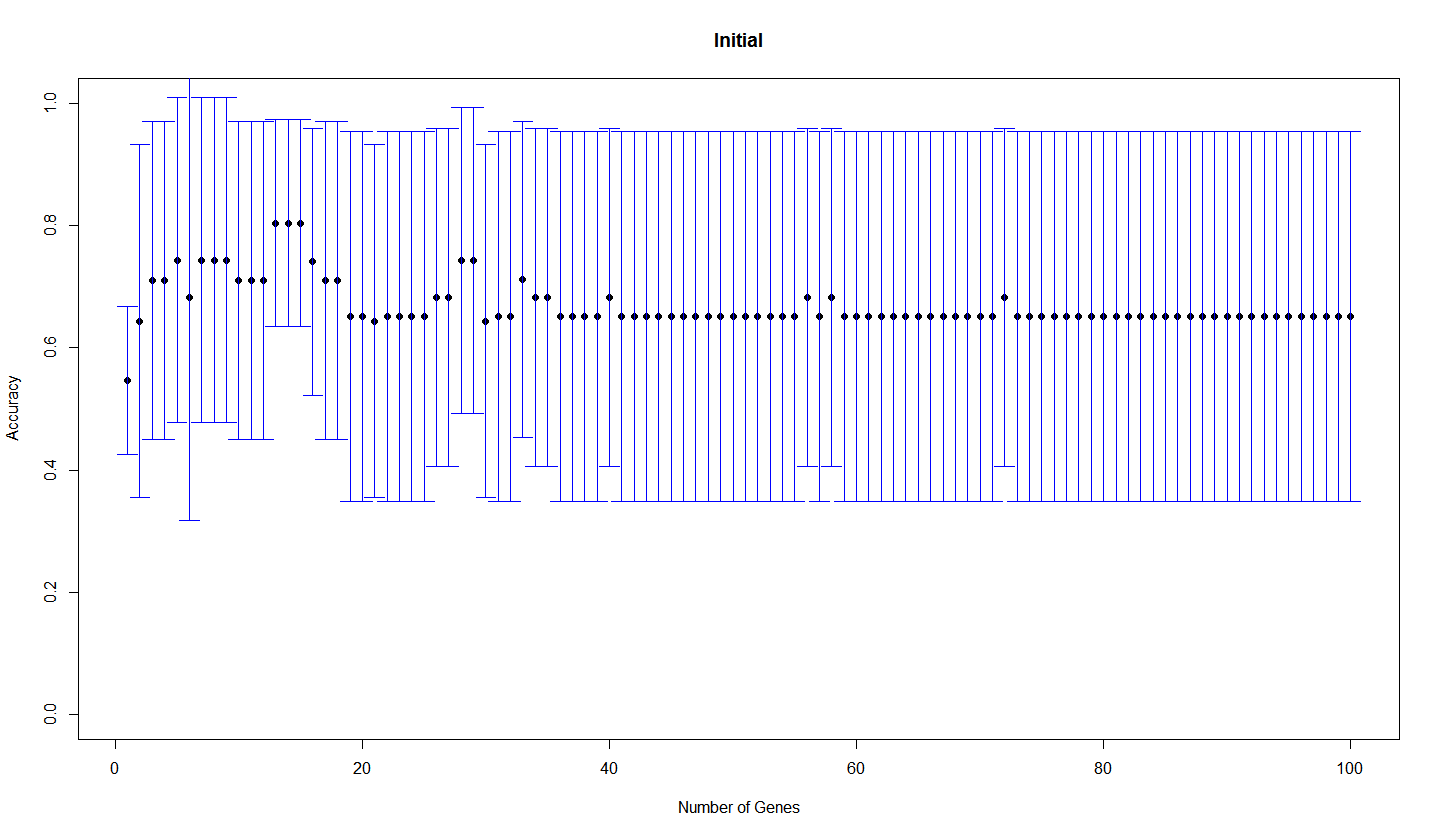
**

**Supplementary Figure 43:** Mean accuracy rate, with error bars, of the sequential gene selection from the top 100 Initial ranked genes (from Limma), using hold out validation with train set the TCGA expression values and test set the expression values from 3 GEO independent datasets for HER2 subtype of breast cancer.

**
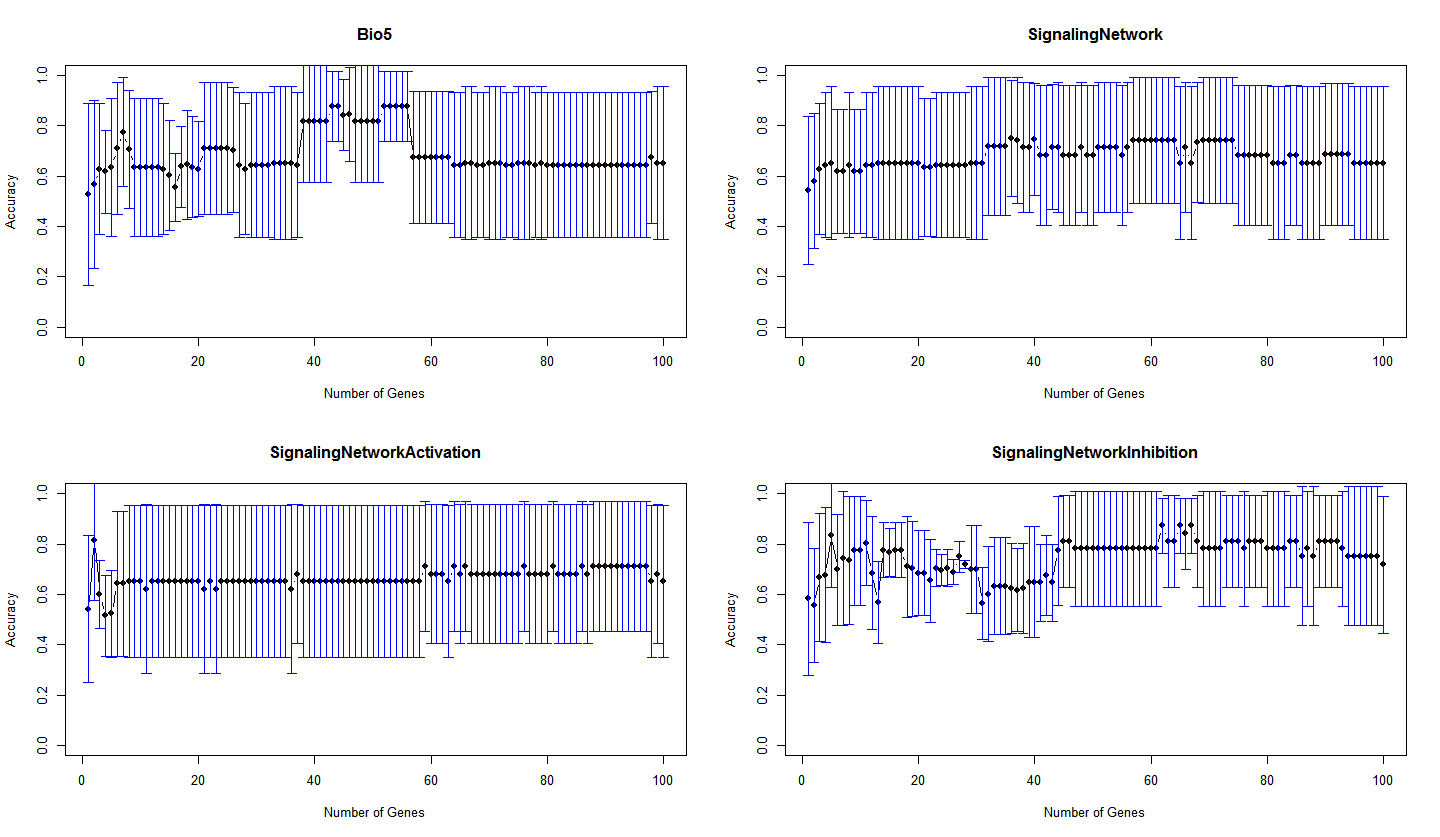
**

**
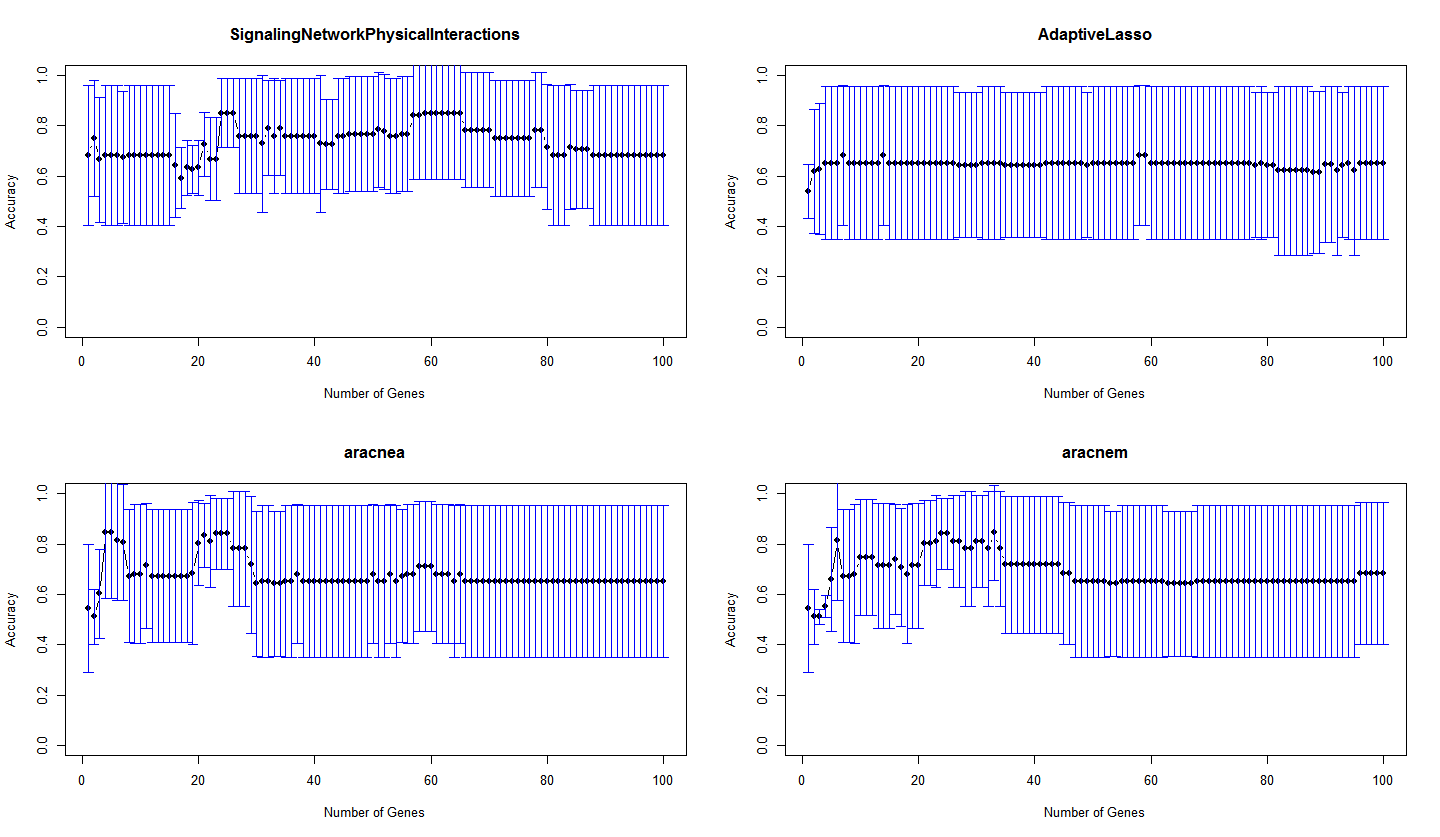
**

**
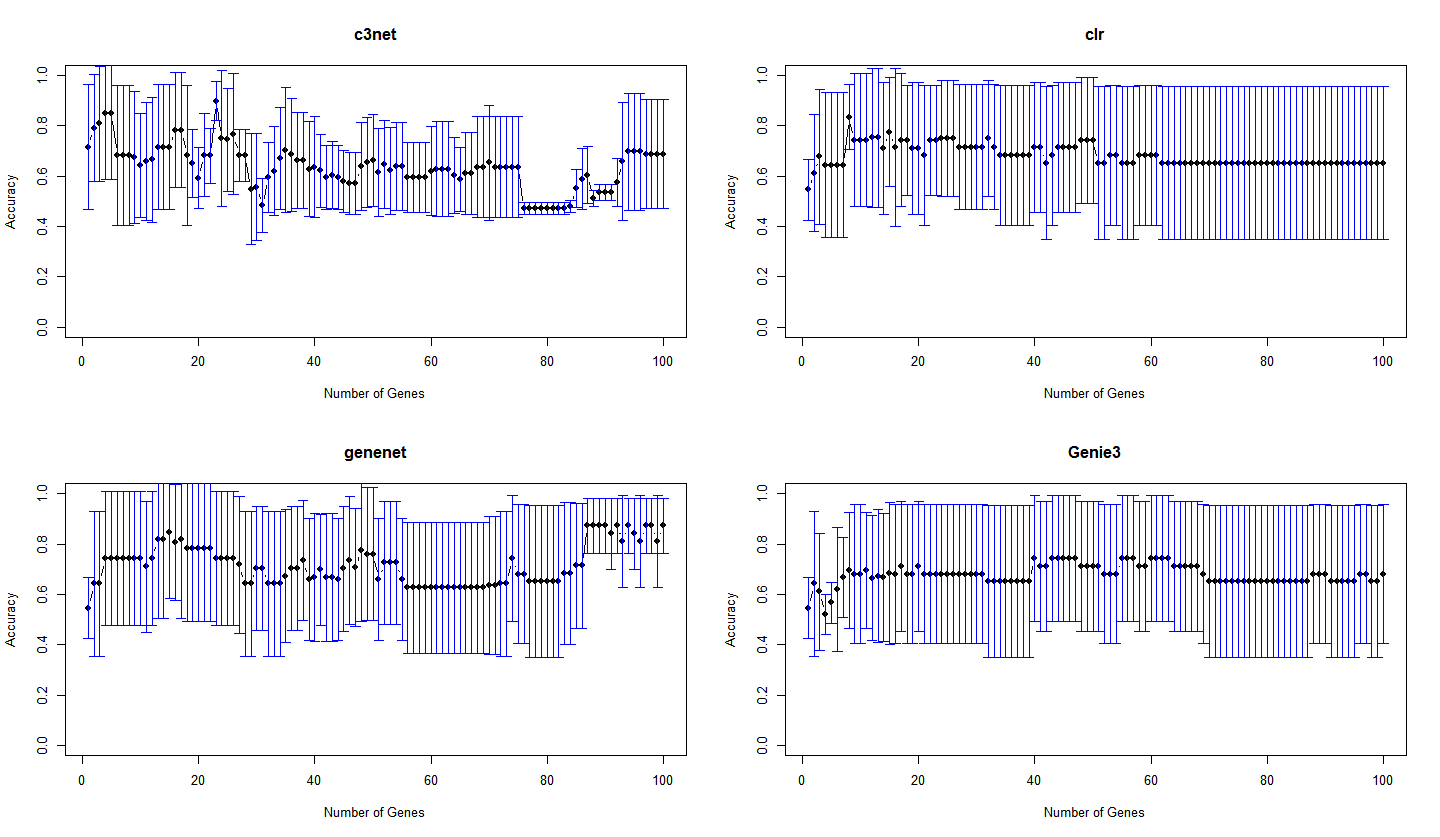
**

**
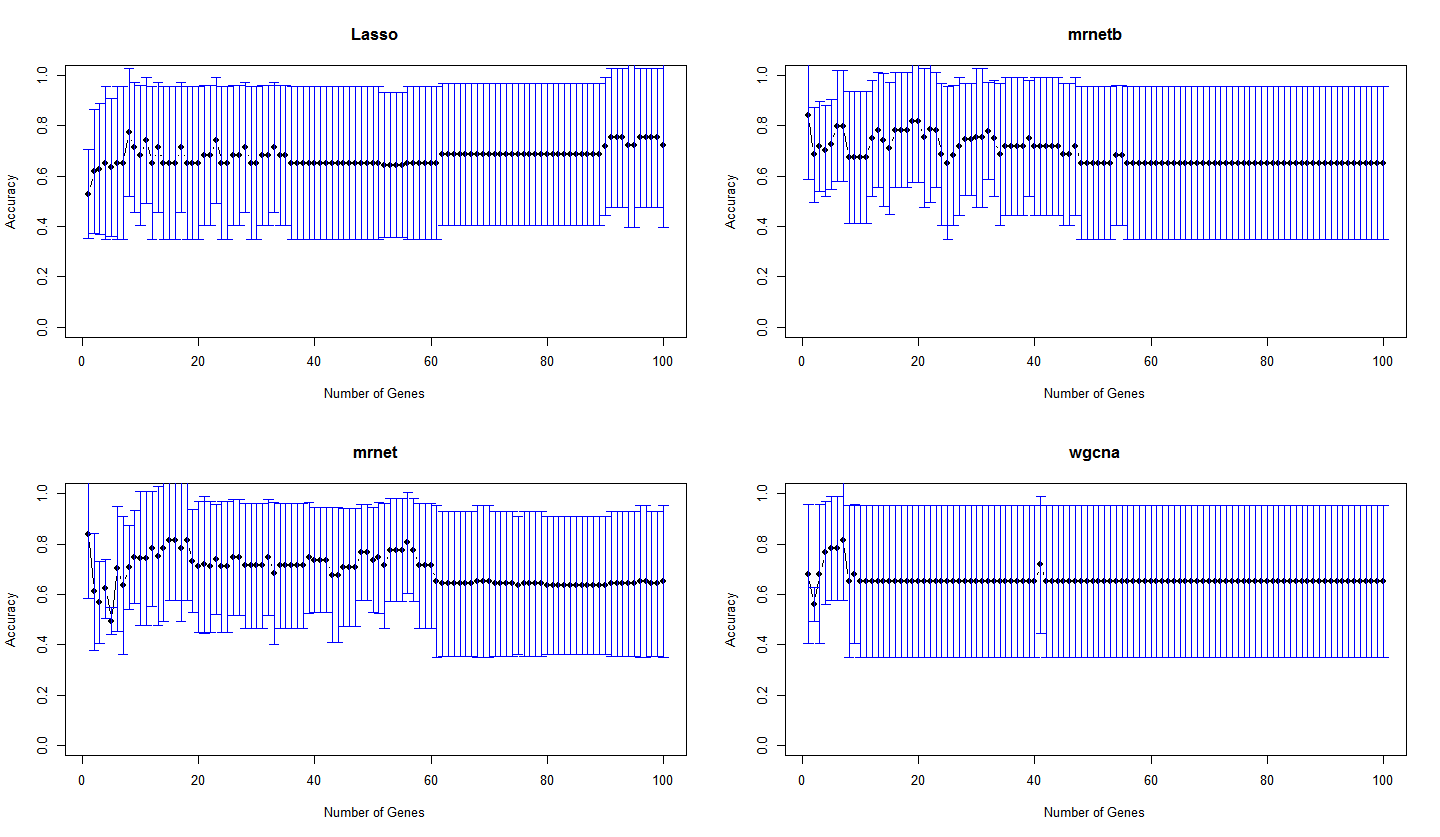
**

**Supplementary Figure 44:** Mean accuracy rates, with error bars, of the top 100 sequential genes from all ranked and re-ranked gene lists from each method in combination with PageRank reconciling method, using hold out validation with train set the TCGA expression values and test set the expression values from 3 GEO independent datasets HER2 subtype of breast

**
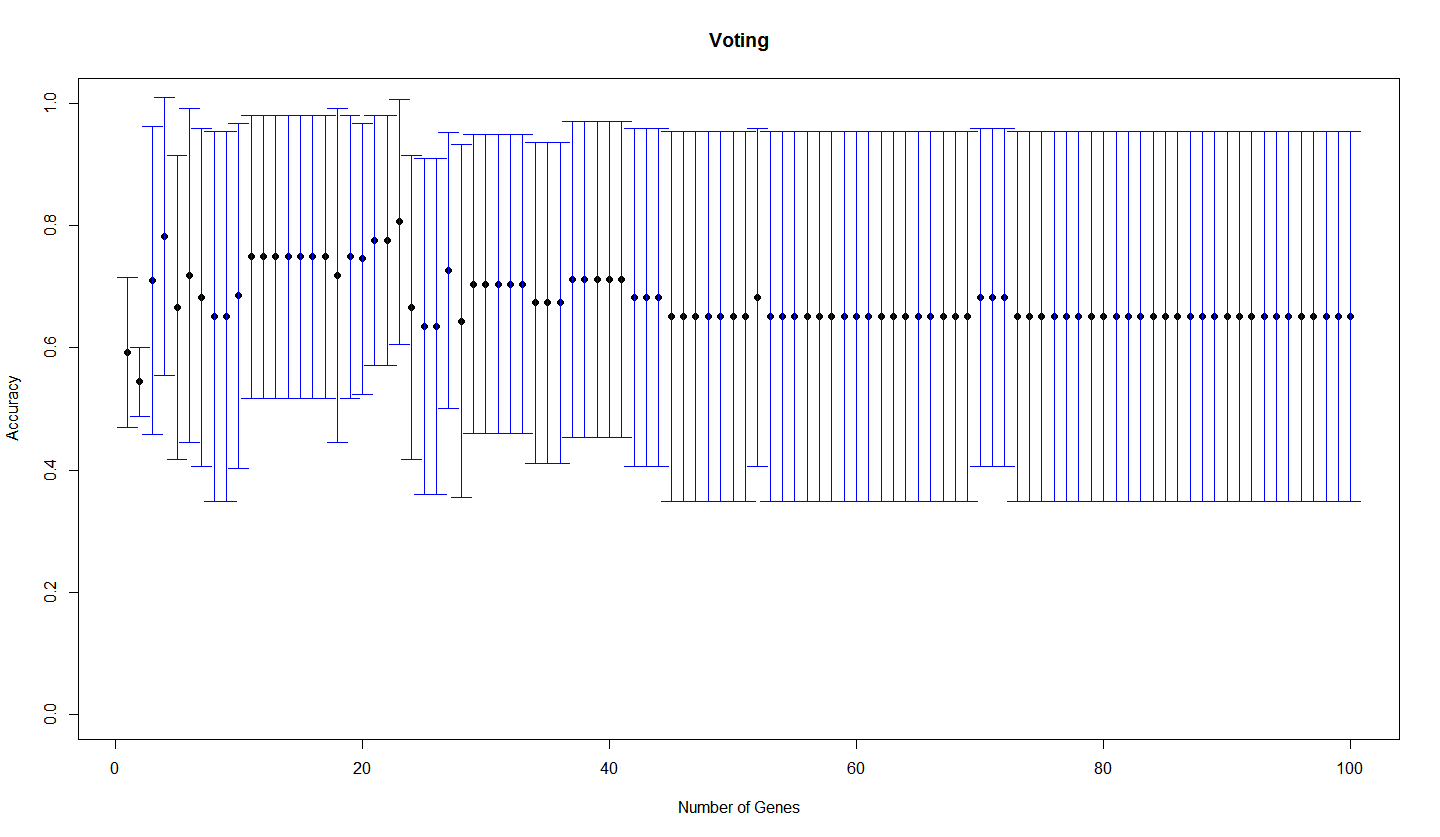
**

**Supplementary Figure 45:** Mean accuracy rate, with error bars, of the sequential gene selection the top 100 re-ranked genes with the ensemble Voting method, using hold out validation with train set the TCGA expression values and test set the expression values from 3 GEO independent datasets for HER2 subtype of breast cancer.

**
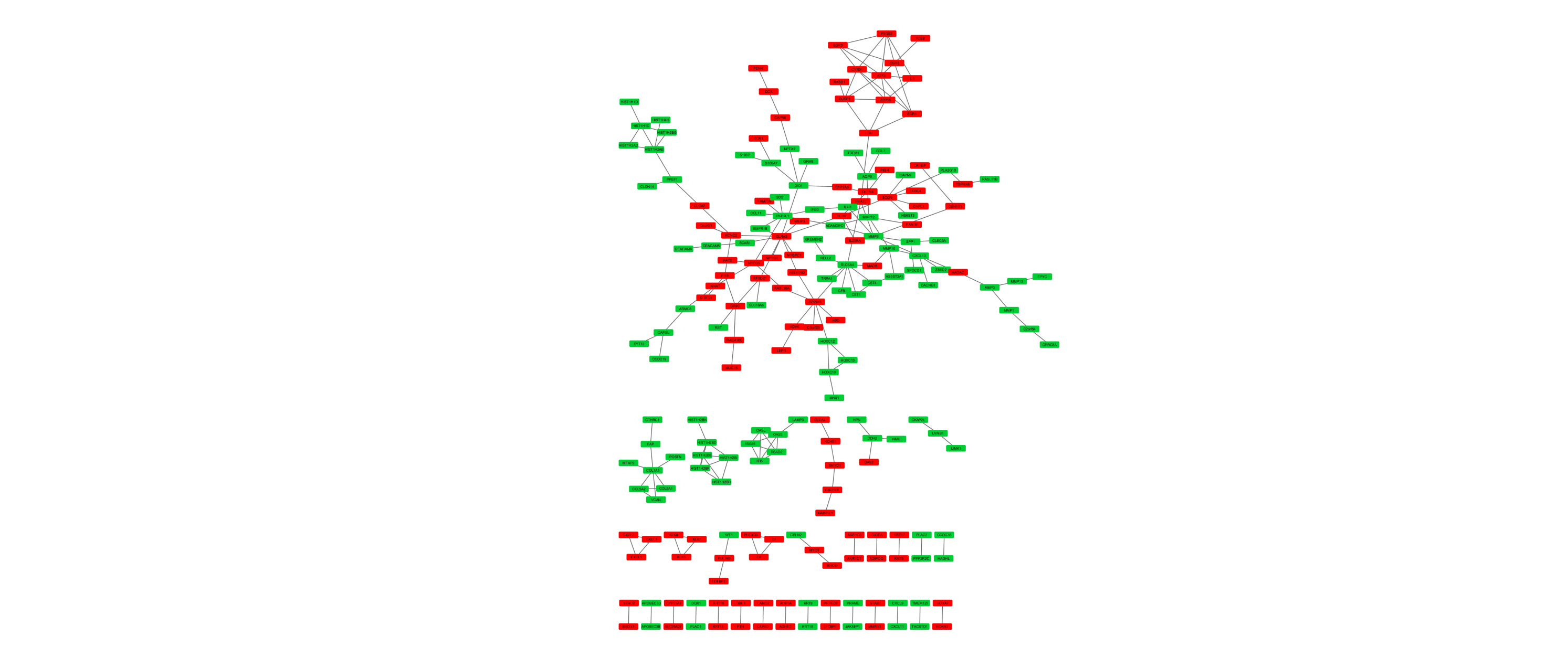

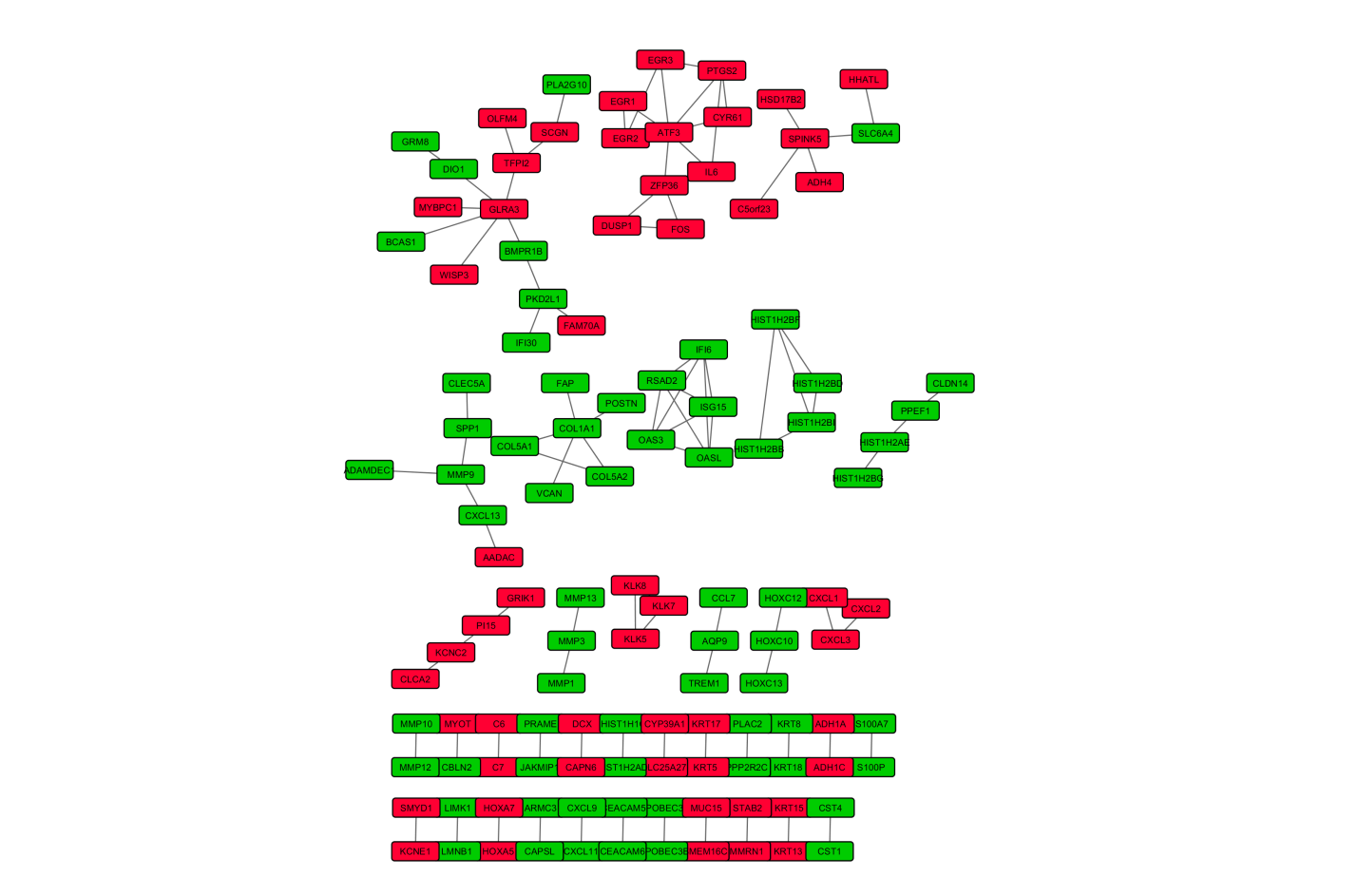
**

(a) (b)

**Supplementary Figure 46:** (a) Genenet pattern of Breast cancer Stage I (b) Top 100 interactions based on their weights.

**
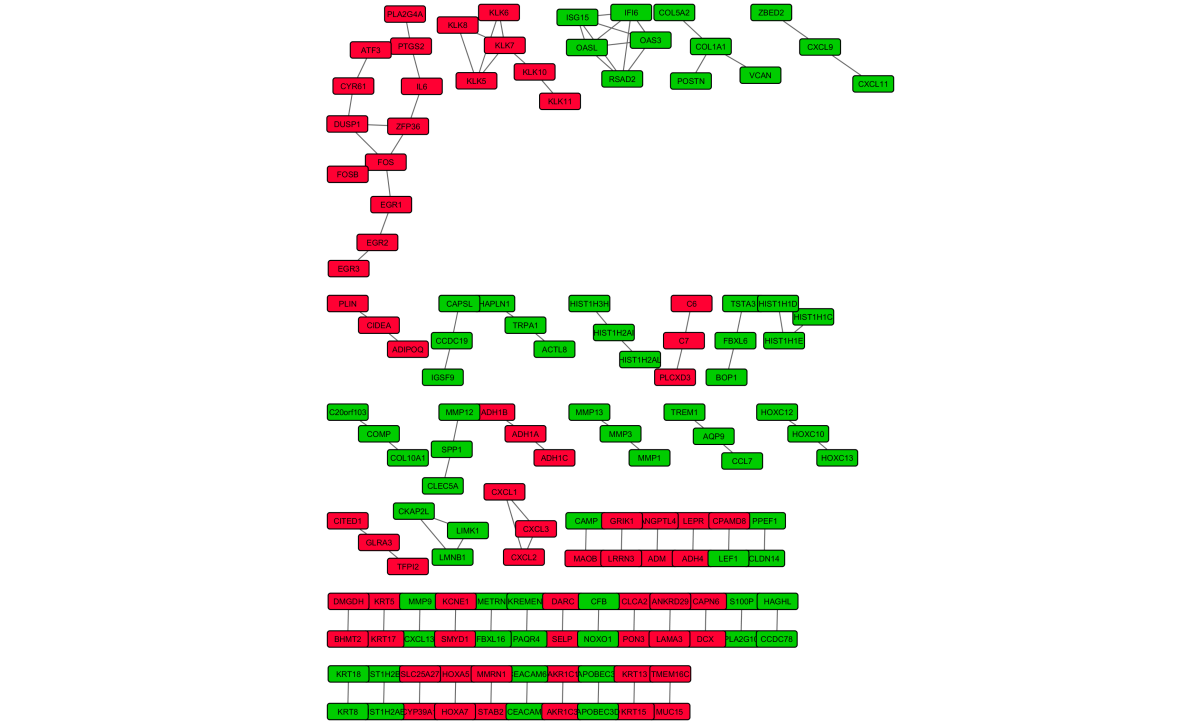

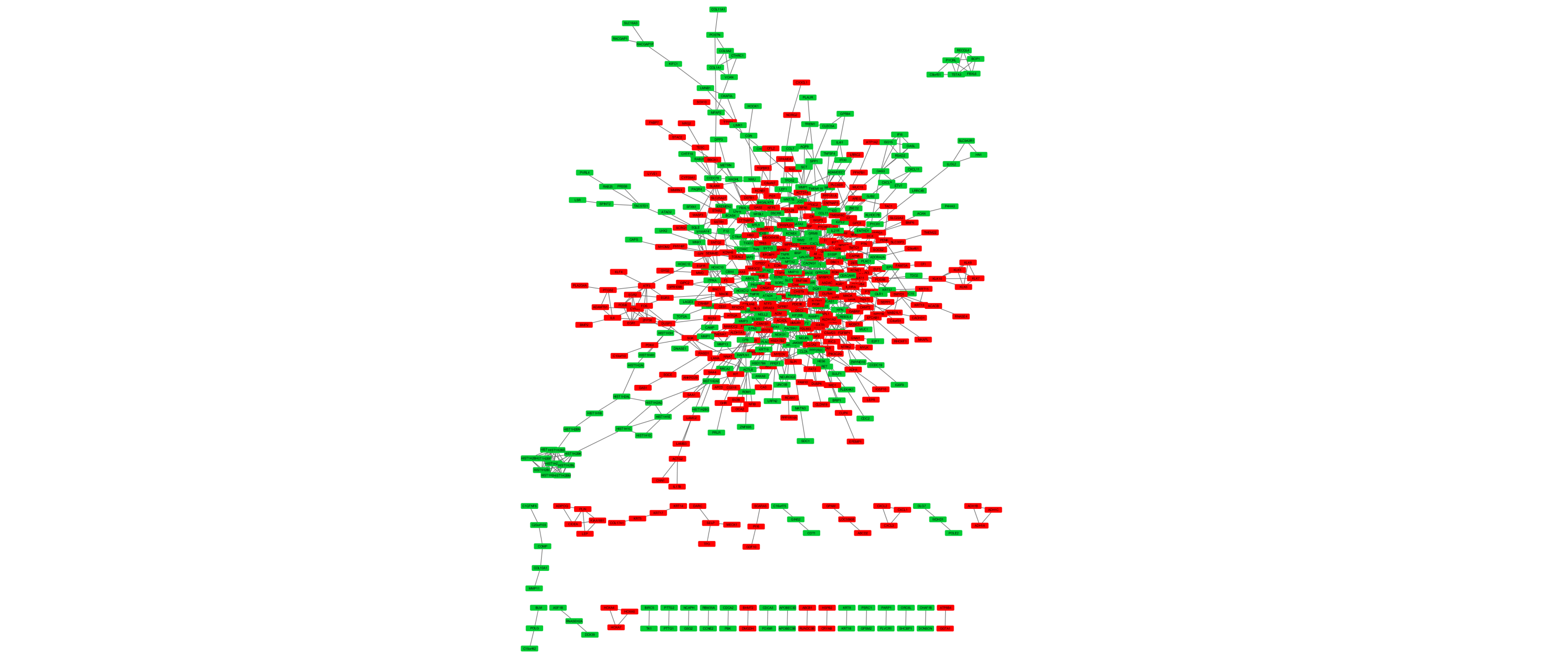
**

(a) (b)

**Supplementary Figure 47:** (a) Genenet pattern of Breast cancer Stage II (b) Top 100 interactions based on their weights.

**
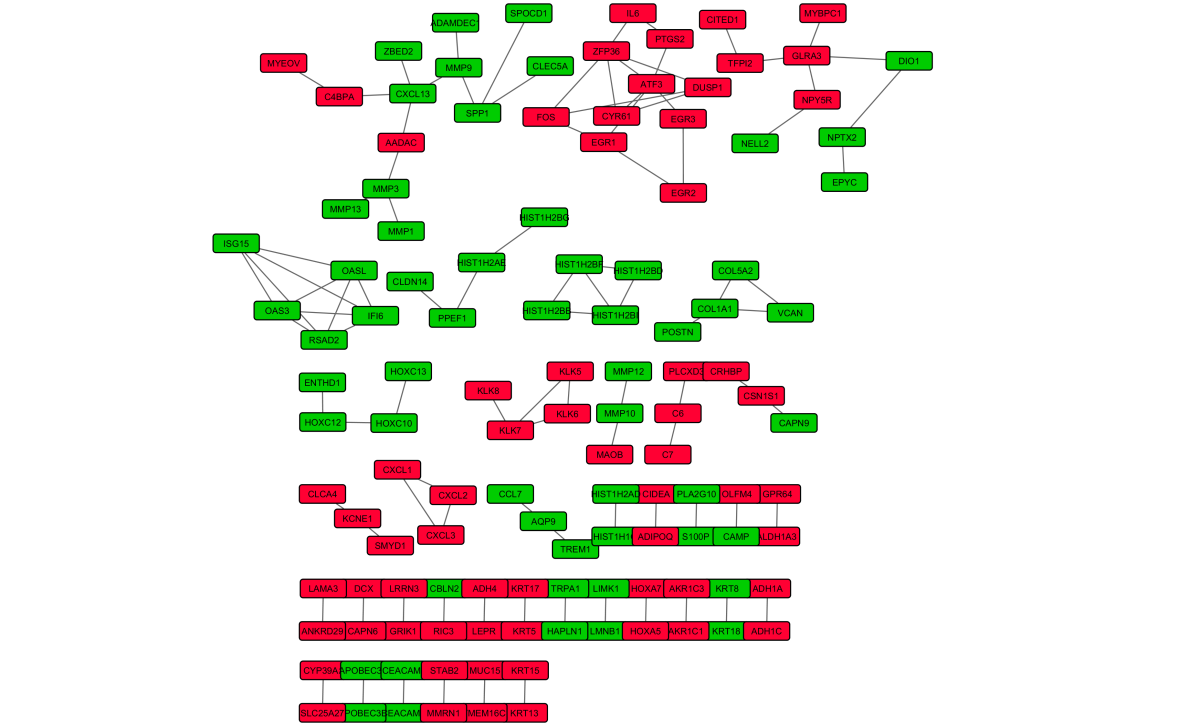

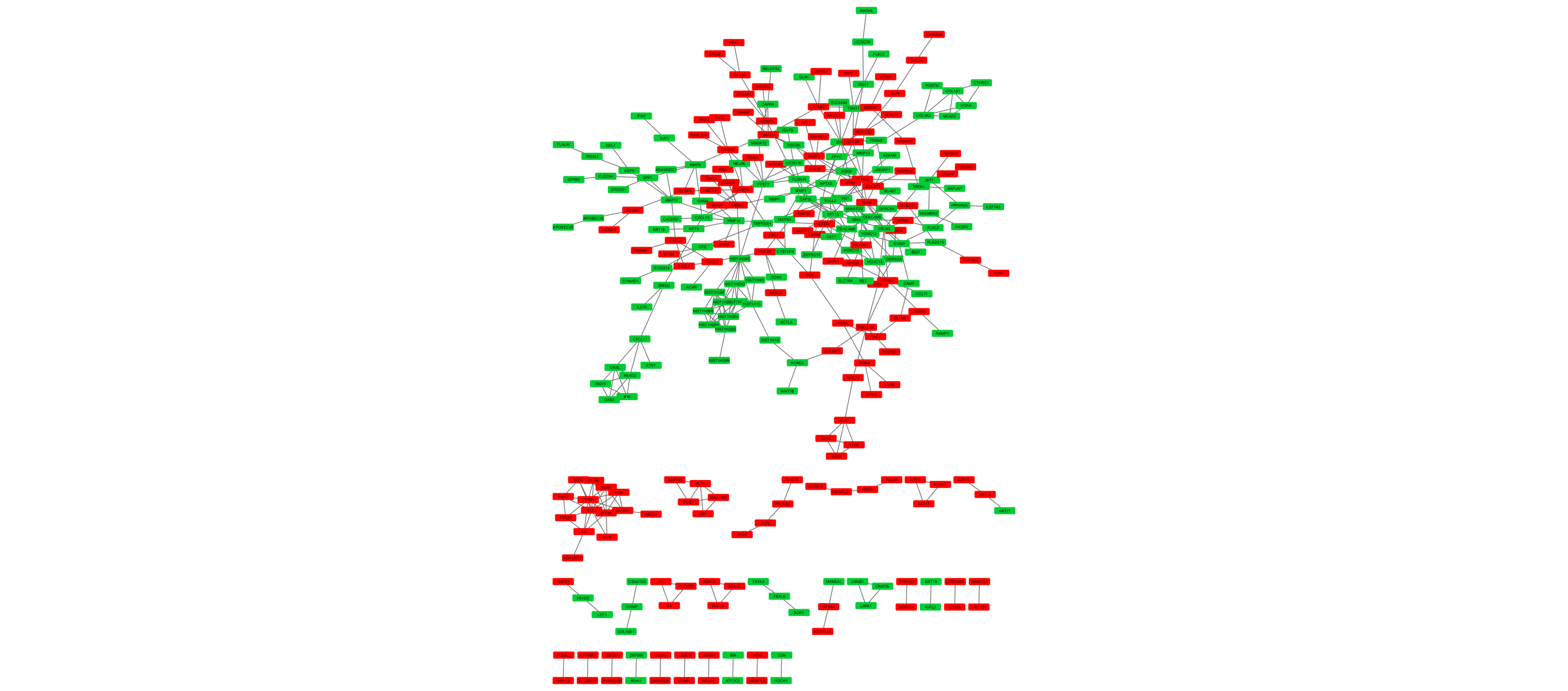
**

(a) (b)

**Supplementary Figure 48:** (a) Genenet pattern of Breast cancer Stage III (b) Top 100 interactions based on their weights.

**
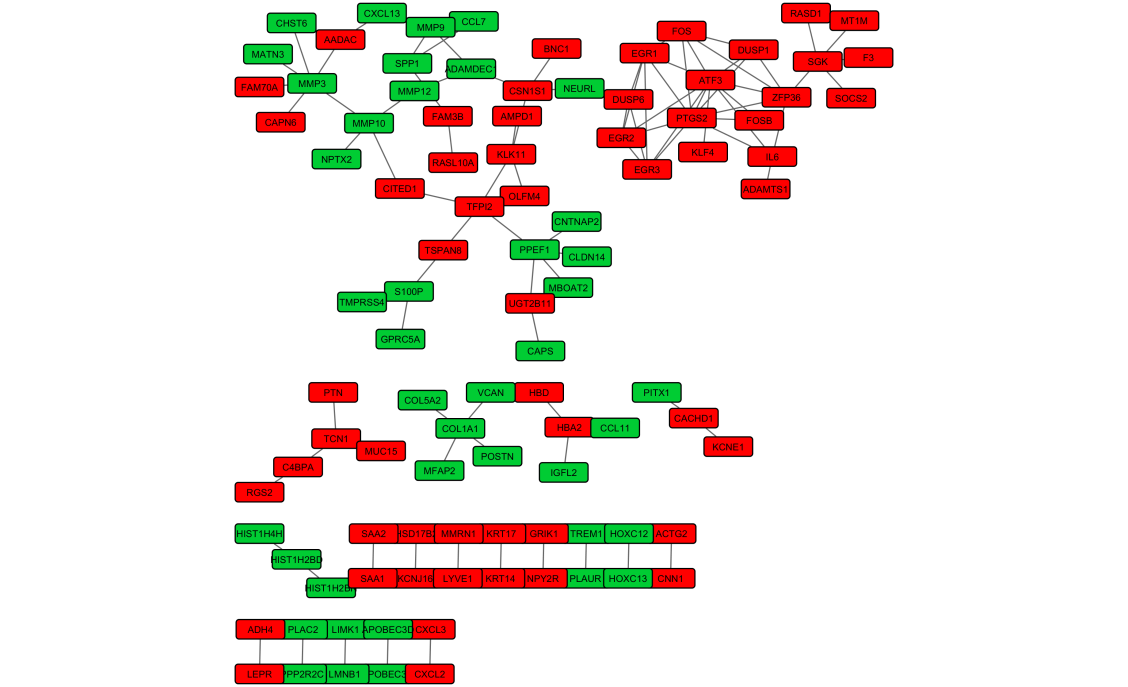

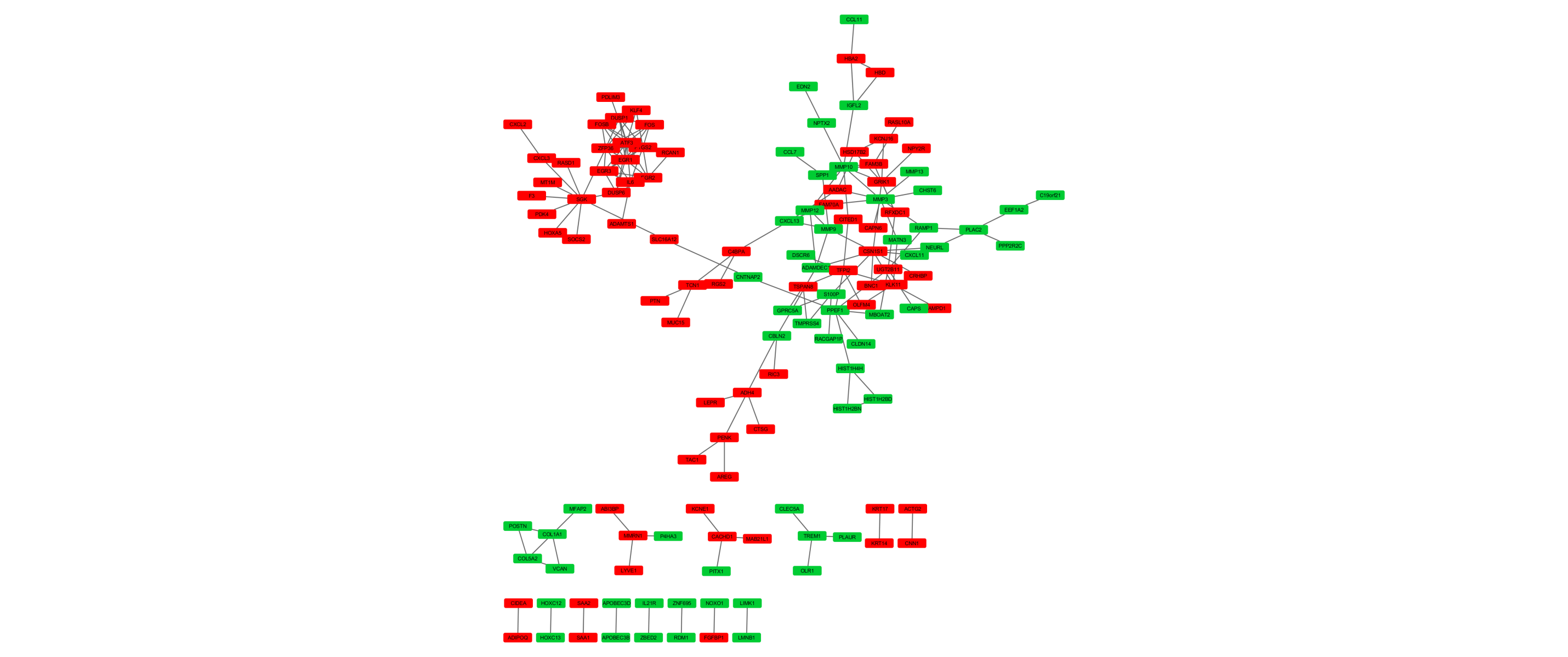
**

(a) (b)

**Supplementary Figure 49:** (a) Genenet pattern of Breast cancer Stage IV (b) Top 100 interactions based on their weights.

**
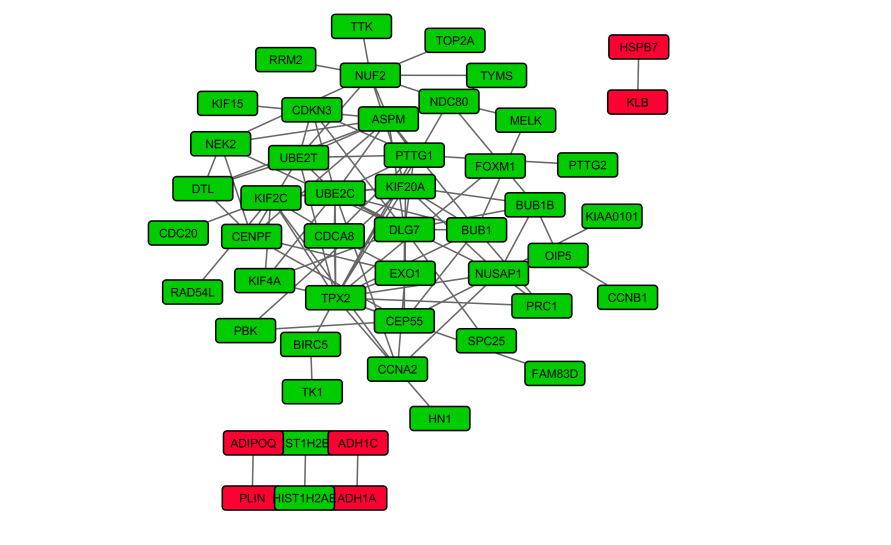

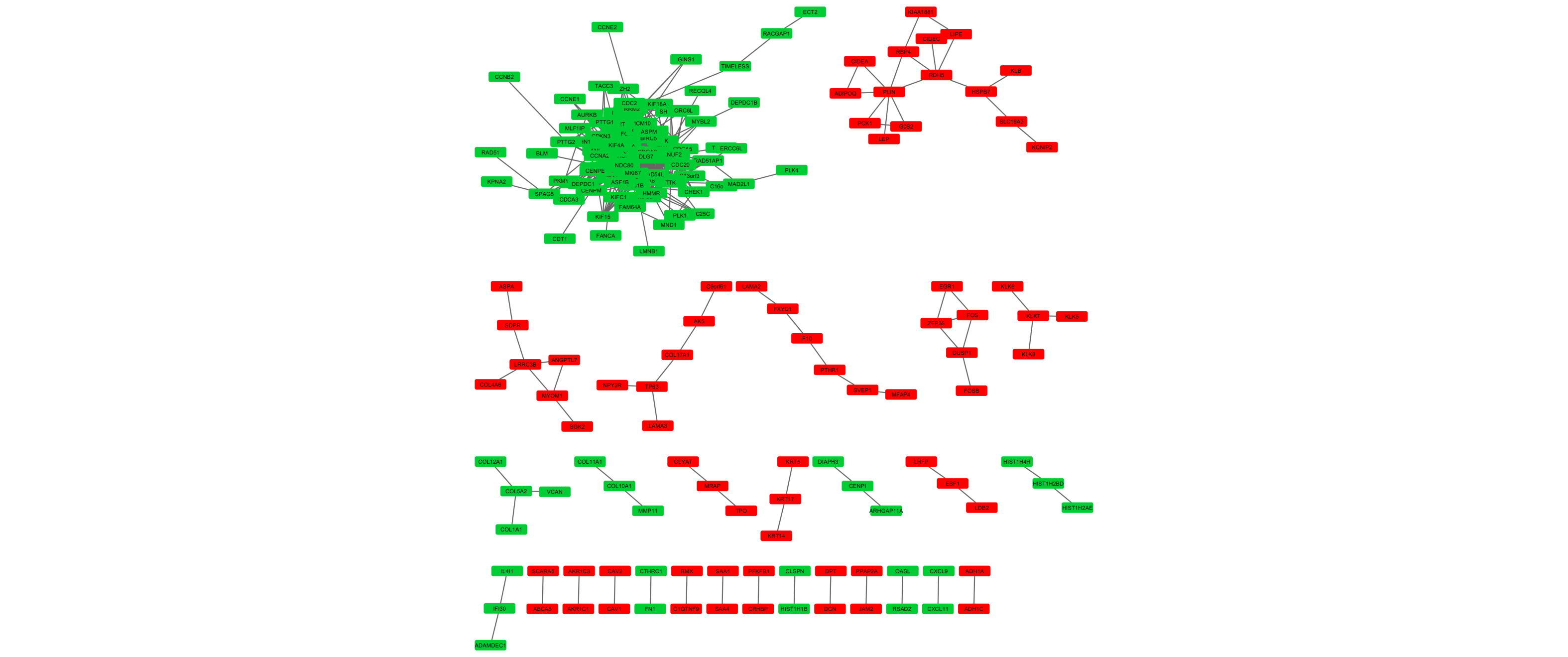
**

(a) (b)

**Supplementary Figure 50:** (a) MRNETB pattern of Triple Negative Breast cancer subtype (b) Top 100 interactions based on their weights.

(a) (b)

**Supplementary Figure 51:** (a) MRNETB pattern of Luminal A Breast cancer subtype (b) Top 100 interactions based on their weights.

(a) (b)

**Supplementary Figure 52:** (a) MRNETB pattern of Luminal B Breast cancer subtype (b) Top 100 interactions based on their weights.

(a) (b)

**Supplementary Figure 53:** (a) MRNETB pattern of HER2 Breast cancer subtype (b) Top 100 interactions based on their weights.

**Supplementary Figure 54:** Stage I - Hierarchical clustering of the top 20 proposed Stage I specific and the 25 already used breast cancer drugs using Tanimito similarity and Ward distance

**Supplementary Figure 55:** Stage II - Hierarchical clustering of the top 20 proposed Stage II specific and the 25 already used breast cancer drugs using Tanimito similarity and Ward distance

**Supplementary Figure 56:** Stage III - Hierarchical clustering of the top 20 proposed Stage III specific and the 25 already used breast cancer drugs using Tanimito similarity and Ward distance

**Supplementary Figure 57:** Stage IV - Hierarchical clustering of the top 20 proposed Stage IV specific and the 25 already used breast cancer drugs using Tanimito similarity and Ward distance

**Supplementary Figure 58:** Triple Negative - Hierarchical clustering of the top 20 proposed Triple Negative specific and the 25 already used breast cancer drugs using Tanimito similarity and Ward distance

**Supplementary Figure 59:** Luminal A - Hierarchical clustering of the top 20 proposed Luminal A specific and the 25 already used breast cancer drugs using Tanimito similarity and Ward distance

**Supplementary Figure 60:** Luminal B - Hierarchical clustering of the top 20 proposed Luminal B specific and the 25 already used breast cancer drugs using Tanimito similarity and Ward distance

**Supplementary Figure 61:** HER2 - Hierarchical clustering of the top 20 proposed HER2 specific and the 25 already used breast cancer drugs using Tanimito similarity and Ward distance

**Supplementary Table 1:** LINCS Drug Names with their corresponding ChemSpider ids.

| **LINCS Drug Names** | **ChemSpider IDs** |
| --- | --- |
| WYE-125132 | CSID:24652498 |
| Wortmannin | CSID:276037 |
| Withaferin-a | CSID:233064 |
| WAY-170523 | CSID:8006126 |
| Trichostatin-a | CSID:4901264 |
| Teniposide | CSID:31930 |
| SIB-1893 | CSID:4470920 |
| Pyrvinium-pamoate | CSID:10152945 |
| PLX-4720 | CSID:22376238 |
| K784-3187 | CSID:2921673 |
| Cytarabine | CSID:6017 |
| Cyclosporin-a | CSID:4447449 |
| Clobetasol | CSID:4470588 |
| Cladribine | CSID:19105 |
| Chrysenequinone | CSID:15483 |
| CD-437 | CSID:119278 |
| 5-[(Benzyloxy)methyl]-7-(1-pyrrolidinylmethyl)-8-quinolinol | CSID:2670336 |
| (4E)-2-(4-Methoxyphenyl)-4-[(4-methoxyphenyl)imino]-4H-chromen-6-ol | CSID:1007249 |
| Idarubicin -LINCS | CSID:39117 |
| Etoposide - LINCS | CSID:33510 |
| WZ-4002 | CSID:24608638 |
| Vorinostat | CSID:5120 |
| Tubastatin-a | CSID:28295021 |
| Triciribine | CSID:58865 |
| Purvalanol-a | CSID:401707 |
| Palbociclib | CSID:4487437 |
| KIN001-055 | CSID:5036104 |
| Fluticasone | CSID:4470631 |
| Entinostat | CSID:4111 |
| Dasatinib | CSID:2323020 |
| Barasertib | CSID:13137620 |
| Aminopurvalanol-a | CSID:5037187 |
| Gemcitabine | CSID:54753 |
| ZM-241385 | CSID:153646 |
| Serdemetan | CSID:9784341 |
| RO-28-1675 | CSID:8061759 |
| Quinoclamine | CSID:16770 |
| Obatoclax | CSID:21430401 |
| NVP-BEZ235 | CSID:10151099 |
| NM-PP1 | CSID:4327766 |
| N-[(5-Fluoro-8-hydroxy-7-quinolinyl)(2-thienyl)methyl]acetamide | CSID:26371306 |
| Methylene-blue | CSID:5874 |
| Menadione | CSID:3915 |
| L-690488 | CSID:4306665 |
| Ingenol | CSID:10282511 |
| Diphenyleneiodonium | CSID:2990 |
| Chlorambucil | CSID:2607 |
| Benzydamine | CSID:12036 |
| Benzamide | CSID:2241 |
| 4-(keto-methyl-oxido-sulfuraniumyl)-3-nitro-benzoic acid [2-(2-adamantylamino)-2-keto-1-methyl-ethyl] ester | CSID:26371450 |
| 5-[(Benzyloxy)methyl]-7-(1-piperidinylmethyl)-8-quinolinol | CSID:2489394 |
| Tunicamycin | CSID:17347361 |
| PAC-1 | CSID:12272376 |
| N-[(5-Bromo-8-hydroxy-7-quinolinyl)(2-thienyl)methyl]acetamide | CSID:26402631 |
| lonidamine | CSID:36170 |
| Heliomycin | CSID:4445287 |
| 6-(2,3-Dihydro-1,4-benzodioxin-6-yl)-N-(3-methylbenzyl)-4-quinazolinamine | CSID:24631074 |
| 4-[4-(4-Fluorobenzyl)-1-piperidinyl]thieno[2,3-d]pyrimidine | CSID:26392812 |
| TPCA-1 | CSID:8079440 |
| Selamectin | CSID:16738655 |
| Rhodomyrtoxin-b | CSID:2520167 |
| Quizartinib | CSID:24640357 |
| Niclosamide | CSID:4322 |
| Methyl-2,5-dihydroxycinnamate | CSID:4510151 |
| KM-03949SC | CSID:2098610 |
| Irinotecan | CSID:54825 |
| Homoharringtonine | CSID:251215 |
| Gemcitabine | CSID:54753 |
| EMF-sumo1-12 | CSID:1952299 |
| Calmidazolium | CSID:2435 |
| BAS-02859604 | CSID:969569 |
| Ampicillin | CSID:6013 |
| 4-{[5-(1-Naphthyl)-1,3,4-oxadiazol-2-yl]sulfanyl}-2-butyn-1-yl 4-(dimethylamino)benzoate | CSID:26363708 |
| 2-Dichloromethyl-4-ethylsulfanyl-6-phenyl-[1,3,5]triazine | CSID:683375 |
| SKF-83959 | CSID:117797 |
| SCH-79797 | CSID:21475061 |
| SA-1478088 | CSID:9467665 |
| Ruxolitinib | CSID:25027389 |
| Paroxetine | CSID:39888 |
| N-[5-(4-Morpholinylsulfonyl)-2-(1-pyrrolidinyl)phenyl]-4,5,6,7-tetrahydro-1-benzothiophene-2-carboxamide | CSID:11081330 |
| N'-[(E)-(2,3-Dihydroxyphenyl)methylene]-2-hydroxybenzohydrazide | CSID:12551687 |
| Milrinone | CSID:4052 |
| Mifepristone | CSID:49889 |
| Ixazomib | CSID:25027391 |
| INCA-6 | CSID:201000 |
| BIBR-1532 | CSID:8103164 |
| 6-(1,3-Benzodioxol-5-yl)-N-(cyclopentylmethyl)-4-quinazolinamine | CSID:26356474 |
| 4-(4-Methoxyphenoxy)-2-(4-methylphenyl)-5-(2-thienyl)-3(2H)-pyridazinone | CSID:26394722 |
| Clofarabine - LINCS | CSID:106472 |
| Pevonedistat | CSID:17625129 |
| IKK-2-inhibitor-V | CSID:4258263 |
| Artesunate | CSID:5293084 |
| AG-592 | CSID:4578420 |
| 3-(3-Benzoyl-6-chloro-4,5-dihydroxy-1-benzofuran-7-yl)-2,4-pentanedione | CSID:578565 |
| Sepantronium bromide | CSID:9353328 |
| SCH 79797 dihydrochloride | CSID:21475061 |
| SA-792541 | CSID:230135 |
| N-[2-(allyloxy)benzyl]-N-1,3-benzodioxol-5-yl-2-chloroacetamide | CSID:1828443 |
| L755507 | CSID:8005571 |
| Kinetin-riboside | CSID:19164 |
| HBEG | CSID:140808 |
| 4-{2-[(6-Chloro-4-quinazolinyl)amino]ethyl}phenol | CSID:24704847 |
| 2-Chlor-N-(1-phenyl-3-propyl-1H-pyrazol-5-yl)acetamid | CSID:1589461 |
| 1-benzhydryl-4-[(5-methyl-4-nitroisoxazol-3-yl)carbonyl]piperazine | CSID:13087408 |
| Vanoxerine | CSID:3337 |

**Supplementary Table 2:** Scoring the Ranked Gene Lists for Breast Cancer Stage I

|  | **min** | **Num**  **genes** | **max** | **Num**  **genes** | **mean** | **sd** | [**numgenes @ max_penalty**](mailto:numgenes@max_penalty) | **max-min** | **cv** | **cv_weight** | **Score** |
| --- | --- | --- | --- | --- | --- | --- | --- | --- | --- | --- | --- |
| **AdLasso** | 0.836 | 95 | 1 | 2 | 0.927 | 0.050 | 1 | 0.164 | 0.054 | 0.9 | 0.900 |
| **Aracnea** | 0.575 | 1 | 0.944 | 66 | 0.834 | 0.104 | 0.4 | 0.369 | 0.125 | 0.8 | 0.302 |
| **Aracnem** | 0.628 | 1 | 0.944 | 66 | 0.822 | 0.089 | 0.4 | 0.317 | 0.108 | 0.8 | 0.302 |
| **Bio5** | 0.740 | 11 | 1 | 39 | 0.949 | 0.052 | 0.7 | 0.260 | 0.055 | 0.9 | 0.630 |
| **C3net** | 0.587 | 1 | 1 | 20 | 0.884 | 0.074 | 0.8 | 0.413 | 0.084 | 0.9 | 0.720 |
| **CLR** | 0.641 | 2 | 1 | 12 | 0.920 | 0.059 | 0.9 | 0.359 | 0.064 | 0.9 | 0.810 |
| **Genenet** | 0.820 | 20 | 1 | 4 | 0.933 | 0.061 | 1 | 0.180 | 0.066 | 0.9 | 0.900 |
| **Genie3** | 0.737 | 3 | 1 | 15 | 0.936 | 0.065 | 0.9 | 0.263 | 0.069 | 0.9 | 0.810 |
| **Initial** | 0.931 | 12 | 1 | 4 | 0.961 | 0.026 | 1 | 0.069 | 0.027 | 1 | 1.000 |
| **Lasso** | 0.616 | 1 | 1 | 2 | 0.937 | 0.085 | 1 | 0.384 | 0.091 | 0.9 | 0.900 |
| **MRNET** | 0.778 | 5 | 1 | 28 | 0.890 | 0.037 | 0.8 | 0.222 | 0.042 | 1 | 0.800 |
| **MRNETB** | 0.670 | 2 | 1.000 | 53 | 0.913 | 0.090 | 0.5 | 0.330 | 0.099 | 0.9 | 0.450 |
| **SN** | 0.724 | 1 | 1 | 9 | 0.949 | 0.052 | 1 | 0.276 | 0.055 | 0.9 | 0.900 |
| **SN_A** | 0.697 | 5 | 1 | 11 | 0.955 | 0.074 | 0.9 | 0.303 | 0.077 | 0.9 | 0.810 |
| **SN_I** | 0.793 | 1 | 1 | 9 | 0.968 | 0.045 | 1 | 0.207 | 0.047 | 1 | 1.000 |
| **SN_PI** | 0.480 | 33 | 0.986 | 75 | 0.741 | 0.130 | 0.3 | 0.506 | 0.175 | 0.7 | 0.207 |
| **Voting** | 0.628 | 1 | 1 | 14 | 0.914 | 0.090 | 0.9 | 0.372 | 0.099 | 0.9 | 0.810 |
| **WGCNA** | 0.764 | 4 | 1 | 6 | 0.960 | 0.054 | 1 | 0.236 | 0.056 | 0.9 | 0.900 |

**Supplementary Table 3:** Scoring the Ranked Gene Lists for Breast Cancer Stage II

|  | **min** | **Num**  **genes** | **max** | **Num**  **genes** | **mean** | **sd** | [**numgenes @ max_penalty**](mailto:numgenes@max_penalty) | **max-min** | **cv** | **cv_weight** | **Score** |
| --- | --- | --- | --- | --- | --- | --- | --- | --- | --- | --- | --- |
| **AdLasso** | 0.692 | 1 | 1 | 5 | 0.980 | 0.033 | 1 | 0.308 | 0.033 | 1 | 1 |
| **Aracnea** | 0.498 | 12 | 1 | 45 | 0.894 | 0.147 | 0.6 | 0.502 | 0.164 | 0.7 | 0.42 |
| **Aracnem** | 0.555 | 19 | 1 | 29 | 0.905 | 0.126 | 0.8 | 0.445 | 0.139 | 0.8 | 0.64 |
| **Bio5** | 0.861 | 12 | 1 | 32 | 0.972 | 0.033 | 0.7 | 0.139 | 0.034 | 1 | 0.7 |
| **C3net** | 0.529 | 12 | 1 | 42 | 0.934 | 0.119 | 0.6 | 0.471 | 0.128 | 0.8 | 0.48 |
| **CLR** | 0.564 | 6 | 1 | 21 | 0.967 | 0.080 | 0.8 | 0.436 | 0.083 | 0.9 | 0.72 |
| **Genenet** | 0.934 | 13 | 1 | 4 | 0.986 | 0.016 | 1 | 0.066 | 0.016 | 1 | 1 |
| **Genie3** | 0.827 | 1 | 1 | 16 | 0.979 | 0.028 | 0.9 | 0.173 | 0.028 | 1 | 0.9 |
| **Initial** | 0.954 | 87 | 1 | 4 | 0.985 | 0.011 | 1 | 0.046 | 0.011 | 1 | 1 |
| **Lasso** | 0.827 | 27 | 0.98 | 4 | 0.939 | 0.033 | 1 | 0.153 | 0.035 | 1 | 0.98 |
| **MRNET** | 0.739 | 12 | 1 | 22 | 0.974 | 0.040 | 0.8 | 0.261 | 0.041 | 1 | 0.8 |
| **MRNETB** | 0.624 | 27 | 1 | 76 | 0.932 | 0.095 | 0.3 | 0.376 | 0.102 | 0.8 | 0.24 |
| **SN** | 0.722 | 1 | 1 | 22 | 0.967 | 0.034 | 0.8 | 0.278 | 0.035 | 1 | 0.8 |
| **SN_A** | 0.847 | 6 | 1 | 15 | 0.945 | 0.028 | 0.9 | 0.153 | 0.030 | 1 | 0.9 |
| **SN_I** | 0.874 | 1 | 1 | 11 | 0.974 | 0.025 | 0.9 | 0.126 | 0.025 | 1 | 0.9 |
| **SN_PI** | 0.707 | 37 | 0.98 | 79 | 0.897 | 0.077 | 0.3 | 0.273 | 0.086 | 0.9 | 0.265 |
| **Voting** | 0.532 | 14 | 1 | 21 | 0.938 | 0.112 | 0.8 | 0.468 | 0.120 | 0.8 | 0.64 |
| **WGCNA** | 0.72 | 14 | 0.99 | 19 | 0.968 | 0.051 | 0.9 | 0.27 | 0.053 | 0.9 | 0.802 |

**Supplementary Table 4:** Scoring the Ranked Gene Lists for Breast Cancer Stage III

|  | **min** | **Num**  **genes** | **max** | **Num**  **genes** | **mean** | **sd** | [**numgenes @ max_penalty**](mailto:numgenes@max_penalty) | **max-min** | **cv** | **cv_weight** | **Score** |
| --- | --- | --- | --- | --- | --- | --- | --- | --- | --- | --- | --- |
| **AdLasso** | 0.715 | 1 | 1 | 11 | 0.956 | 0.043 | 0.9 | 0.285 | 0.045 | 1 | 0.9 |
| **Aracnea** | 0.461 | 39 | 0.986 | 73 | 0.784 | 0.192 | 0.3 | 0.525 | 0.245 | 0.6 | 0.177 |
| **Aracnem** | 0.511 | 6 | 0.986 | 61 | 0.794 | 0.153 | 0.4 | 0.475 | 0.193 | 0.7 | 0.276 |
| **Bio5** | 0.836 | 1 | 0.986 | 6 | 0.971 | 0.032 | 1 | 0.150 | 0.033 | 1 | 0.986 |
| **C3net** | 0.516 | 8 | 1 | 62 | 0.796 | 0.165 | 0.4 | 0.484 | 0.207 | 0.6 | 0.24 |
| **CLR** | 0.424 | 6 | 0.986 | 47 | 0.904 | 0.109 | 0.6 | 0.562 | 0.120 | 0.8 | 0.473 |
| **Genenet** | 0.745 | 19 | 0.986 | 6 | 0.923 | 0.061 | 1 | 0.241 | 0.066 | 0.9 | 0.887 |
| **Genie3** | 0.827 | 11 | 1 | 86 | 0.966 | 0.029 | 0.2 | 0.173 | 0.031 | 1 | 0.2 |
| **Initial** | 0.895 | 15 | 1 | 4 | 0.979 | 0.018 | 1 | 0.105 | 0.018 | 1 | 1 |
| **Lasso** | 0.715 | 1 | 0.986 | 4 | 0.973 | 0.029 | 1 | 0.271 | 0.029 | 1 | 0.986 |
| **MRNET** | 0.736 | 14 | 1 | 20 | 0.942 | 0.046 | 0.8 | 0.264 | 0.049 | 1 | 0.8 |
| **MRNETB** | 0.600 | 40 | 0.986 | 53 | 0.883 | 0.120 | 0.5 | 0.386 | 0.135 | 0.8 | 0.394 |
| **SN** | 0.621 | 1 | 1 | 15 | 0.953 | 0.056 | 0.9 | 0.379 | 0.059 | 0.9 | 0.81 |
| **SN_A** | 0.826 | 1 | 1 | 16 | 0.979 | 0.038 | 0.9 | 0.174 | 0.039 | 1 | 0.9 |
| **SN_I** | 0.818 | 7 | 1 | 11 | 0.976 | 0.041 | 0.9 | 0.182 | 0.042 | 1 | 0.9 |
| **SN_PI** | 0.538 | 1 | 0.972 | 83 | 0.830 | 0.117 | 0.2 | 0.434 | 0.141 | 0.8 | 0.156 |
| **Voting** | 0.466 | 5 | 0.972 | 64 | 0.871 | 0.123 | 0.4 | 0.506 | 0.141 | 0.8 | 0.311 |
| **WGCNA** | 0.782 | 33 | 0.986 | 7 | 0.951 | 0.038 | 1 | 0.205 | 0.040 | 1 | 0.986 |

**Supplementary Table 5:** Scoring the Ranked Gene Lists for Breast Cancer Stage IV

|  | **min** | **Num**  **genes** | **max** | **Num**  **genes** | **mean** | **sd** | [**numgenes @ max_penalty**](mailto:numgenes@max_penalty) | **max-min** | **cv** | **cv_weight** | **Score** |
| --- | --- | --- | --- | --- | --- | --- | --- | --- | --- | --- | --- |
| **AdLasso** | 0.964 | 1 | 1 | 11 | 0.995 | 0.012 | 0.9 | 0.036 | 0.013 | 1 | 0.9 |
| **Aracnea** | 0.893 | 29 | 1 | 16 | 0.979 | 0.033 | 0.9 | 0.107 | 0.034 | 1 | 0.9 |
| **Aracnem** | 0.893 | 29 | 1 | 2 | 0.977 | 0.029 | 1 | 0.107 | 0.030 | 1 | 1 |
| **Bio5** | 0.893 | 7 | 1 | 1 | 0.970 | 0.046 | 1 | 0.107 | 0.047 | 1 | 1 |
| **C3net** | 0.929 | 20 | 1 | 7 | 0.976 | 0.020 | 1 | 0.071 | 0.020 | 1 | 1 |
| **CLR** | 0.929 | 10 | 1 | 2 | 0.984 | 0.020 | 1 | 0.071 | 0.020 | 1 | 1 |
| **Genenet** | 0.964 | 11 | 1 | 1 | 0.989 | 0.016 | 1 | 0.036 | 0.017 | 1 | 1 |
| **Genie3** | 0.893 | 6 | 1 | 7 | 0.991 | 0.019 | 1 | 0.107 | 0.019 | 1 | 1 |
| **Initial** | 1.000 | 1 | 1 | 1 | 1 | 0 | 1 | 0 | 0 | 1 | 1 |
| **Lasso** | 0.893 | 1 | 1 | 10 | 0.996 | 0.015 | 0.9 | 0.107 | 0.016 | 1 | 0.9 |
| **MRNET** | 0.964 | 1 | 1 | 3 | 0.999 | 0.007 | 1 | 0.036 | 0.007 | 1 | 1 |
| **MRNETB** | 0.929 | 1 | 1 | 7 | 0.991 | 0.019 | 1 | 0.071 | 0.019 | 1 | 1 |
| **SN** | 0.786 | 4 | 1 | 6 | 0.987 | 0.034 | 1 | 0.214 | 0.034 | 1 | 1 |
| **SN_A** | 0.607 | 6 | 1 | 8 | 0.988 | 0.057 | 1 | 0.393 | 0.057 | 0.9 | 0.9 |
| **SN_I** | 0.964 | 1 | 1 | 6 | 0.991 | 0.016 | 1 | 0.036 | 0.016 | 1 | 1 |
| **SN_PI** | 0.929 | 1 | 1 | 69 | 0.965 | 0.008 | 0.4 | 0.071 | 0.008 | 1 | 0.4 |
| **Voting** | 0.964 | 1 | 1 | 2 | 0.994 | 0.013 | 1 | 0.036 | 0.013 | 1 | 1 |
| **WGCNA** | 0.929 | 4 | 1 | 5 | 0.975 | 0.017 | 1 | 0.071 | 0.018 | 1 | 1 |

**Supplementary Table 6:** Scoring the Ranked Gene Lists for Triple Negative Breast Cancer

|  | **min** | **Num**  **genes** | **max** | **Num**  **genes** | **mean** | **sd** | [**numgenes @ max_penalty**](mailto:numgenes@max_penalty) | **max-min** | **cv** | **cv_weight** | **Score** |
| --- | --- | --- | --- | --- | --- | --- | --- | --- | --- | --- | --- |
| **AdLasso** | 0.617 | 69 | 0.733 | 45 | 0.665 | 0.029 | 0.6 | 0.116 | 0.043 | 1 | 0.440 |
| **Aracnea** | 0.532 | 48 | 0.868 | 10 | 0.621 | 0.092 | 0.9 | 0.336 | 0.149 | 0.8 | 0.625 |
| **Aracnem** | 0.554 | 29 | 0.858 | 5 | 0.622 | 0.081 | 1 | 0.304 | 0.130 | 0.8 | 0.687 |
| **Bio5** | 0.540 | 12 | 0.854 | 43 | 0.720 | 0.092 | 0.6 | 0.313 | 0.128 | 0.8 | 0.410 |
| **C3net** | 0.527 | 31 | 0.749 | 4 | 0.616 | 0.034 | 1 | 0.222 | 0.055 | 0.9 | 0.674 |
| **CLR** | 0.680 | 96 | 0.895 | 13 | 0.744 | 0.064 | 0.9 | 0.216 | 0.086 | 0.9 | 0.725 |
| **Genenet** | 0.539 | 1 | 0.875 | 62 | 0.728 | 0.105 | 0.4 | 0.336 | 0.144 | 0.8 | 0.280 |
| **Genie3** | 0.637 | 28 | 0.875 | 10 | 0.733 | 0.049 | 0.9 | 0.237 | 0.067 | 0.9 | 0.708 |
| **Initial** | 0.539 | 1 | 0.740 | 6 | 0.692 | 0.037 | 1 | 0.201 | 0.053 | 0.9 | 0.666 |
| **Lasso** | 0.607 | 1 | 0.821 | 60 | 0.682 | 0.042 | 0.4 | 0.214 | 0.061 | 0.9 | 0.296 |
| **MRNET** | 0.595 | 67 | 0.880 | 13 | 0.718 | 0.073 | 0.9 | 0.285 | 0.102 | 0.8 | 0.633 |
| **MRNETB** | 0.657 | 97 | 0.895 | 21 | 0.765 | 0.068 | 0.8 | 0.238 | 0.088 | 0.9 | 0.645 |
| **SN** | 0.577 | 62 | 0.781 | 75 | 0.699 | 0.053 | 0.3 | 0.204 | 0.076 | 0.9 | 0.211 |
| **SN_A** | 0.603 | 18 | 0.811 | 67 | 0.734 | 0.057 | 0.4 | 0.208 | 0.078 | 0.9 | 0.292 |
| **SN_I** | 0.553 | 15 | 0.893 | 42 | 0.710 | 0.083 | 0.6 | 0.340 | 0.116 | 0.8 | 0.429 |
| **SN_PI** | 0.544 | 93 | 0.815 | 13 | 0.682 | 0.070 | 0.9 | 0.272 | 0.102 | 0.8 | 0.587 |
| **Voting** | 0.615 | 72 | 0.891 | 7 | 0.701 | 0.051 | 1 | 0.276 | 0.073 | 0.9 | 0.802 |
| **WGCNA** | 0.710 | 4 | 0.809 | 17 | 0.767 | 0.021 | 0.9 | 0.098 | 0.028 | 1 | 0.728 |

**Supplementary Table 7:** Scoring the Ranked Gene Lists for Luminal A Breast Cancer

|  | **min** | **Num**  **genes** | **max** | **Num**  **genes** | **mean** | **sd** | [**numgenes @ max_penalty**](mailto:numgenes@max_penalty) | **max-min** | **cv** | **cv_weight** | **Score** |
| --- | --- | --- | --- | --- | --- | --- | --- | --- | --- | --- | --- |
| **AdLasso** | 0.614 | 92 | 0.844 | 1 | 0.724 | 0.048 | 1 | 0.231 | 0.066 | 0.9 | 0.760 |
| **Aracnea** | 0.584 | 2 | 0.858 | 90 | 0.767 | 0.054 | 0.1 | 0.274 | 0.070 | 0.9 | 0.077 |
| **Aracnem** | 0.421 | 36 | 0.907 | 70 | 0.740 | 0.135 | 0.3 | 0.487 | 0.182 | 0.7 | 0.191 |
| **Bio5** | 0.529 | 35 | 0.908 | 8 | 0.811 | 0.106 | 1 | 0.379 | 0.131 | 0.8 | 0.726 |
| **C3net** | 0.582 | 17 | 0.907 | 33 | 0.770 | 0.066 | 0.7 | 0.325 | 0.086 | 0.9 | 0.572 |
| **CLR** | 0.665 | 1 | 0.878 | 42 | 0.771 | 0.054 | 0.6 | 0.213 | 0.070 | 0.9 | 0.474 |
| **Genenet** | 0.466 | 1 | 0.874 | 97 | 0.760 | 0.088 | 0.1 | 0.408 | 0.116 | 0.8 | 0.070 |
| **Genie3** | 0.665 | 1 | 0.888 | 29 | 0.797 | 0.062 | 0.8 | 0.223 | 0.078 | 0.9 | 0.639 |
| **Initial** | 0.466 | 1 | 0.863 | 54 | 0.805 | 0.076 | 0.5 | 0.397 | 0.094 | 0.9 | 0.388 |
| **Lasso** | 0.513 | 1 | 0.898 | 77 | 0.757 | 0.096 | 0.3 | 0.385 | 0.127 | 0.8 | 0.215 |
| **MRNET** | 0.612 | 48 | 0.885 | 10 | 0.741 | 0.069 | 0.9 | 0.274 | 0.094 | 0.9 | 0.717 |
| **MRNETB** | 0.651 | 12 | 0.892 | 18 | 0.773 | 0.069 | 0.9 | 0.240 | 0.089 | 0.9 | 0.722 |
| **SN** | 0.563 | 15 | 0.911 | 89 | 0.756 | 0.097 | 0.2 | 0.348 | 0.128 | 0.8 | 0.146 |
| **SN_A** | 0.550 | 9 | 0.872 | 91 | 0.777 | 0.084 | 0.1 | 0.322 | 0.108 | 0.8 | 0.070 |
| **SN_I** | 0.553 | 1 | 0.893 | 41 | 0.779 | 0.087 | 0.6 | 0.340 | 0.111 | 0.8 | 0.428 |
| **SN_PI** | 0.496 | 4 | 0.865 | 24 | 0.711 | 0.067 | 0.8 | 0.369 | 0.094 | 0.9 | 0.623 |
| **Voting** | 0.620 | 94 | 0.879 | 12 | 0.760 | 0.064 | 0.9 | 0.259 | 0.084 | 0.9 | 0.712 |
| **WGCNA** | 0.665 | 1 | 0.917 | 28 | 0.806 | 0.051 | 0.8 | 0.251 | 0.063 | 0.9 | 0.660 |

**Supplementary Table 8:** Scoring the Ranked Gene Lists for Luminal B Breast Cancer

|  | **min** | **Num**  **genes** | **max** | **Num**  **genes** | **mean** | **sd** | [**numgenes @ max_penalty**](mailto:numgenes@max_penalty) | **max-min** | **cv** | **cv_weight** | **Score** |
| --- | --- | --- | --- | --- | --- | --- | --- | --- | --- | --- | --- |
| **AdLasso** | 0.554 | 1 | 0.871 | 40 | 0.812 | 0.076 | 0.6 | 0.317 | 0.094 | 0.9 | 0.470 |
| **Aracnea** | 0.477 | 24 | 0.890 | 16 | 0.682 | 0.095 | 0.9 | 0.412 | 0.140 | 0.8 | 0.641 |
| **Aracnem** | 0.477 | 18 | 0.840 | 16 | 0.681 | 0.103 | 0.9 | 0.362 | 0.151 | 0.7 | 0.529 |
| **Bio5** | 0.549 | 1 | 0.871 | 46 | 0.790 | 0.097 | 0.6 | 0.322 | 0.123 | 0.8 | 0.418 |
| **C3net** | 0.445 | 1 | 0.846 | 47 | 0.725 | 0.109 | 0.6 | 0.400 | 0.151 | 0.7 | 0.355 |
| **CLR** | 0.599 | 1 | 0.867 | 21 | 0.764 | 0.053 | 0.8 | 0.267 | 0.069 | 0.9 | 0.624 |
| **Genenet** | 0.520 | 2 | 0.892 | 99 | 0.716 | 0.075 | 0.1 | 0.372 | 0.104 | 0.8 | 0.071 |
| **Genie3** | 0.529 | 2 | 0.846 | 12 | 0.771 | 0.044 | 0.9 | 0.317 | 0.057 | 0.9 | 0.685 |
| **Initial** | 0.599 | 1 | 0.846 | 36 | 0.771 | 0.052 | 0.7 | 0.247 | 0.067 | 0.9 | 0.533 |
| **Lasso** | 0.445 | 1 | 0.846 | 64 | 0.773 | 0.069 | 0.4 | 0.400 | 0.090 | 0.9 | 0.304 |
| **MRNET** | 0.554 | 1 | 0.846 | 13 | 0.731 | 0.065 | 0.9 | 0.292 | 0.089 | 0.9 | 0.685 |
| **MRNETB** | 0.554 | 1 | 0.871 | 9 | 0.761 | 0.072 | 1 | 0.317 | 0.094 | 0.9 | 0.784 |
| **SN** | 0.545 | 2 | 0.85 | 100 | 0.744 | 0.052 | 0.1 | 0.305 | 0.070 | 0.9 | 0.077 |
| **SN_A** | 0.541 | 3 | 0.846 | 74 | 0.752 | 0.063 | 0.3 | 0.305 | 0.084 | 0.9 | 0.228 |
| **SN_I** | 0.526 | 3 | 0.800 | 30 | 0.695 | 0.049 | 0.7 | 0.274 | 0.070 | 0.9 | 0.504 |
| **SN_PI** | 0.529 | 2 | 0.846 | 67 | 0.714 | 0.087 | 0.4 | 0.317 | 0.122 | 0.8 | 0.271 |
| **Voting** | 0.529 | 2 | 0.846 | 6 | 0.682 | 0.048 | 1 | 0.318 | 0.070 | 0.9 | 0.762 |
| **WGCNA** | 0.554 | 1 | 0.888 | 31 | 0.796 | 0.047 | 0.7 | 0.334 | 0.059 | 0.9 | 0.559 |

**Supplementary Table 9:** Scoring the Ranked Gene Lists for HER2 Breast Cancer

|  | **min** | **Num**  **genes** | **max** | **Num**  **genes** | **mean** | **sd** | [**numgenes @ max_penalty**](mailto:numgenes@max_penalty) | **max-min** | **cv** | **cv_weight** | **Score** |
| --- | --- | --- | --- | --- | --- | --- | --- | --- | --- | --- | --- |
| **AdLasso** | 0.538 | 1 | 0.682 | 7 | 0.646 | 0.017 | 1 | 0.144 | 0.026 | 1 | 0.682 |
| **Aracnea** | 0.512 | 2 | 0.848 | 4 | 0.679 | 0.061 | 1 | 0.337 | 0.089 | 0.9 | 0.764 |
| **Aracnem** | 0.511 | 3 | 0.845 | 33 | 0.687 | 0.066 | 0.7 | 0.334 | 0.095 | 0.9 | 0.533 |
| **Bio5** | 0.528 | 1 | 0.876 | 43 | 0.686 | 0.082 | 0.6 | 0.348 | 0.119 | 0.8 | 0.420 |
| **C3net** | 0.473 | 76 | 0.898 | 23 | 0.635 | 0.087 | 0.8 | 0.425 | 0.136 | 0.8 | 0.575 |
| **CLR** | 0.546 | 1 | 0.834 | 8 | 0.682 | 0.043 | 1 | 0.288 | 0.063 | 0.9 | 0.751 |
| **Genenet** | 0.546 | 1 | 0.873 | 87 | 0.718 | 0.080 | 0.2 | 0.327 | 0.111 | 0.8 | 0.140 |
| **Genie3** | 0.521 | 4 | 0.742 | 40 | 0.678 | 0.040 | 0.6 | 0.221 | 0.059 | 0.9 | 0.401 |
| **Initial** | 0.546 | 1 | 0.804 | 13 | 0.669 | 0.038 | 0.9 | 0.258 | 0.057 | 0.9 | 0.651 |
| **Lasso** | 0.529 | 1 | 0.773 | 8 | 0.677 | 0.036 | 1 | 0.244 | 0.053 | 0.9 | 0.695 |
| **MRNET** | 0.495 | 5 | 0.840 | 1 | 0.693 | 0.061 | 1 | 0.346 | 0.087 | 0.9 | 0.756 |
| **MRNETB** | 0.652 | 25 | 0.840 | 1 | 0.691 | 0.051 | 1 | 0.189 | 0.074 | 0.9 | 0.756 |
| **SN** | 0.542 | 1 | 0.748 | 36 | 0.679 | 0.042 | 0.7 | 0.206 | 0.062 | 0.9 | 0.472 |
| **SN_A** | 0.516 | 4 | 0.815 | 2 | 0.665 | 0.038 | 1 | 0.300 | 0.057 | 0.9 | 0.734 |
| **SN_I** | 0.556 | 2 | 0.873 | 62 | 0.746 | 0.072 | 0.4 | 0.317 | 0.096 | 0.9 | 0.314 |
| **SN_PI** | 0.592 | 17 | 0.850 | 24 | 0.736 | 0.060 | 0.8 | 0.258 | 0.082 | 0.9 | 0.612 |
| **Voting** | 0.544 | 2 | 0.806 | 23 | 0.676 | 0.042 | 0.8 | 0.262 | 0.062 | 0.9 | 0.580 |
| **WGCNA** | 0.560 | 2 | 0.816 | 7 | 0.658 | 0.030 | 1 | 0.256 | 0.045 | 1 | 0.816 |
